# Saliva Microbiota Carry Caries-Specific Functional Gene Signatures

**DOI:** 10.1371/journal.pone.0076458

**Published:** 2014-02-12

**Authors:** Fang Yang, Kang Ning, Xingzhi Chang, Xiao Yuan, Qichao Tu, Tong Yuan, Ye Deng, Christopher L. Hemme, Joy Van Nostrand, Xinping Cui, Zhili He, Zhenggang Chen, Dawei Guo, Jiangbo Yu, Yue Zhang, Jizhong Zhou, Jian Xu

**Affiliations:** 1 Oral Research Center, Qingdao Municipal Hospital, Qingdao, Shandong, China; 2 Shandong Key Laboratory of Energy Genetics, CAS Key Laboratory of Biofuels and BioEnergy Genome Center, Qingdao Institute of Bioenergy and Bioprocess Technology, Chinese Academy of Sciences, Qingdao, Shandong, China; 3 Institute for Environmental Genomics and Department of Botany and Microbiology, University of Oklahoma, Norman, Oklahoma, United States of America; 4 Department of Statistics, University of California, Riverside, California, United States of America; Wadsworth Center, United States of America

## Abstract

Human saliva microbiota is phylogenetically divergent among host individuals yet their roles in health and disease are poorly appreciated. We employed a microbial functional gene microarray, HuMiChip 1.0, to reconstruct the global functional profiles of human saliva microbiota from ten healthy and ten caries-active adults. Saliva microbiota in the pilot population featured a vast diversity of functional genes. No significant distinction in gene number or diversity indices was observed between healthy and caries-active microbiota. However, co-presence network analysis of functional genes revealed that caries-active microbiota was more divergent in non-core genes than healthy microbiota, despite both groups exhibited a similar degree of conservation at their respective core genes. Furthermore, functional gene structure of saliva microbiota could potentially distinguish caries-active patients from healthy hosts. Microbial functions such as *Diaminopimelate epimerase*, *Prephenate dehydrogenase*, *Pyruvate-formate lyase* and *N-acetylmuramoyl-L-alanine amidase* were significantly linked to caries. Therefore, saliva microbiota carried disease-associated functional signatures, which could be potentially exploited for caries diagnosis.

## Introduction

Caries is the most common infectious disease throughout the world [1]. Lesions and cavities on tooth surfaces, caused by caries activity, result in infection and pain and can lead to decay and even the loss of tooth structure. Furthermore, once started, the destruction process is usually irreversible. Therefore, preventive measures against caries, as well as the prognosis and early diagnosis, are of particular clinical significance.

Human saliva is home to numerous microorganisms [2], [3], [4], [5], [6]. Evidences have recently emerged from our group and others that the organismal structure of saliva microbiota is highly individualized among human hosts [3], [4], [5], [6], [7], [8] and that changes in organismal structure are linked to caries [9], gingivitis [10] and periodontitis [11]. However, the functional characteristics of saliva microbiota are not well understood [12] and the potential roles of saliva microbiota in health and diseases remain elusive, as (i) organismal lineages do not necessarily correlate with functional activities; (ii) many organisms in a given microbiota are either novel or uncultured; (iii) the degree of microbial functional divergence among host individuals is presently unknown.

Here we reported the global functional profiles of human saliva microbiota associated with dental caries and health. Saliva samples from ten healthy (“H”) and ten caries-active (“C”) hosts were analyzed using HuMiChip 1.0, a new generation of Geochip targeting microbial metabolism in human and mouse microbiota, based on a modified pipeline in the well validated GeoChip3.0 [13]. Our results showed that the functional gene structure of saliva microbiota is able to distinguish caries-active patients from healthy hosts, suggesting that the structure and selected microbial functional gene markers can be potentially exploited for caries diagnosis and perturbation. Thus saliva can serve as a sensitive and non-invasive venue for simultaneously tracking the host, microbial and environmental attributes whose interactions underlie health and disease.

## Materials and Methods

### Study design

All human host volunteers (nearly 700 individuals) were from an oral health census on the undergraduates from the east campus of Sun Yat-sen University, Guangzhou, China, in September, 2009 [9]. After oral health survey, “healthy” individuals (DMFT = 0) and “caries-active” subjects (DMFT≧6) were chosen for saliva sample collection (**Materials S1**). All volunteers provided written informed consent in accordance with the sampling protocol with approval of the ethical committee of the Guanghua Stomatological Hospital, Sun Yat-sen University. They were all unrelated individuals of both genders, aged between 18 and 23 years and shared a relatively homogeneous college-campus living environment. All reported no antibiotics intake for the preceding at least six months and no smoking or tobacco used. All were asked to avoid eating or drinking for 1 h before oral sampling. Those with other oral (for example, periodontitis or halitosis) or systematic diseases were excluded. To decipher the functional landscape of saliva microbiota, 20 saliva samples (including ten from the “healthy” group and ten from the “caries-active” group) were randomly selected for HuMiChip analysis (**Table 1**).

**Table 1:** Background information and microbial diversity of the healthy and caries-active saliva samples.

Sample ID	Group	DMFT index	Gender	Age	Shannon Index	Simpson Index	Gene number
H102	Healthy	0	Male	19	7.59	1659.47	2,361
H106	Healthy	0	Male	18	7.36	798.19	2,573
H107	Healthy	0	Female	22	7.59	1604.16	2,481
H111	Healthy	0	Male	20	7.54	1374.78	2,502
H112	Healthy	0	Male	19	7.65	1411.50	2,757
H116	Healthy	0	Female	19	7.56	1502.65	2,362
H117	Healthy	0	Female	19	7.52	1269.82	2,423
H118	Healthy	0	Female	21	7.45	1101.35	2,360
H121	Healthy	0	Female	19	7.49	1139.90	2,433
H122	Healthy	0	Female	23	7.54	1288.32	2,492
C204	Caries-active	8	Male	21	7.63	1307.07	2,856
C206	Caries-active	6	Female	19	7.44	888.29	2,714
C207	Caries-active	10	Female	19	7.62	1195.71	2,880
C211	Caries-active	7	Male	22	7.42	991.67	2,377
C212	Caries-active	7	Male	22	7.66	1551.77	2,707
C217	Caries-active	8	Male	19	7.50	1053.03	2,616
C219	Caries-active	7	Male	20	7.54	1183.21	2,604
C220	Caries-active	7	Male	21	7.58	1296.09	2,660
C221	Caries-active	7	Male	19	7.36	934.46	2,246
C222	Caries-active	7	Male	19	7.45	925.66	2,614

### Sample collection and processing

Two milliliters of saliva were collected from each human-host individual into a tube containing an equal volume of lysis buffer (50 mM Tris, pH 8.0, 50 mM EDTA, 50 mM sucrose, 100 mM NaCl and 1% SDS) [10]. Samples were stored at −80°C before high-salt DNA extraction [14]. Thirty microliters of proteinase K (20 mg/mL, Sigma, USA) and 150 µL of 10% SDS were added to 2 mL of the saliva extraction buffer mixture, which was then incubated overnight at 53°C in a shaking water bath. After addition of 400 µL 5 M NaCl and 10 min incubation on ice, the mixture was equally distributed into two 2-mL centrifuge tubes and centrifuged for 10 min at 13,000 rpm in an Eppendorf 5415D centrifuge. The supernatant from each tube was transferred to a new tube, where 800 µL isopropanol was added. The tubes were then incubated for 10 min at room temperature and centrifuged for 15 min at 13,000 rpm. The supernatants were discarded and then the DNA pellets were washed once with 500 µL 70% ethanol, dried and dissolved in 30 µL double-distilled water. Concentrations of the resulted total DNA were measured by Nanovue (GE, USA). DNA purity was determined by A_260_/A_280_, with the inclusion criteria of above 1.8. DNA integrity was verified via agarose gel electrophoresis after ethidium bromide staining under ultraviolet light. DNA Samples were stored at −20°C before further processing.

### HuMiChip analysis of saliva microbiota function

A functional gene microarray (HuMiChip1.0) was developed to interrogate microbial metabolism in human and mouse microbiota (details in **Materials S1**). The design of HuMiChip employed a modified pipeline as that in the well validated GeoChip 3.0 [15], [16], [17]. In total, 36,056 probes targeting 139 functional genes families were included in HuMiChip 1.0, covering 50,007 coding sequences from 322 draft/finished bacterial genomes and 27 shotgun metagenome datasets from various human body sites. The microarrays were synthesized and manufactured by NimbleGen.

HuMiChip analysis was performed for totally 20 saliva microbiota that include ten healthy and ten caries-active ones (**Table 1**). Microarray sample preparation, hybridization, and scaling were performed as previously described [16]. We used minimal signal intensity of 1000 and SNR (Signal to Noise Ratio) cutoff of 2 for positive callings of the presence of a protein. Raw data obtained from microarray image analysis was uploaded to microarray data manager for preprocessing and analysis (http://ieg2.ou.edu/NimbleGen). Functional gene diversity (e.g., Shannon-Weaver index), detrended correspondence analysis (DCA) and permutation *t-*tests were performed using R (version 2.9.1). Permutation *t*-tests were performed based on host dental health-state. All statistical tests were two-sided, with asterisks denoting statistical significance (NS: not significant; *: *p*<0.1; **: *p*<0.05; ***: *p*<0.01). Array data were deposited at the Gene Expression Omnibus with accession numbers GSE49875.

### Statistical analysis in network reconstruction and biomarker detection

The 3,656 functional genes with hybridization signals on HuMiChip were grouped into “complete-presence proteins” (“core”, i.e., those present in all the 20 saliva microbiota) and “partial-presence proteins” (“non-core”, i.e., those missing in at least one saliva microbiota). The “complete-presence proteins” were represented as normalized values according to their signal intensity, while the “partial-presence proteins” as binary values (either 1 or 0). The network of core functional genes was built based on the normalized values of “all-presence genes” for the H and C Groups respectively. The network of non-core functional genes was based on the binary values of “partial-presence genes” on specific metabolic pathways that include Carbon-associated Pathway (including ‘*Complex carbohydrates*’ and ‘*Feeder pathways to glycolysis*’ and ‘*Respiration*’), AA-associated Pathway (including “*Amino acid transport and metabolism*” and “*Amino acid synthesis*”) and Nitrogen-associated Pathway (“*Nitrogen Metabolism”*).

To identify those markers that reliably distinguish caries microbiota, the ten healthy samples were grouped into training (seven samples) and testing (three samples) by all possible combinations (thus 120 different groupings). Based on the binary presence profiles of non-core genes, bootstrapping method was used to randomly select a grouping and then used two steps (“feature selection” and “classification”) to identify biomarkers based on triplet feature selection. Features with the highest discrimination power on the training data were selected and then employed to “predict” the presence profiles of the testing data (**Figure S1; Materials S1**). The biomarkers (each represented as a triplet-feature set of microbial genes) identified after each of the two steps were then subjected to manual inspections before retrieving the final list of biomarkers.

## Results

### Functional gene diversity in healthy and caries-active saliva microbiota

**Figure 1:**
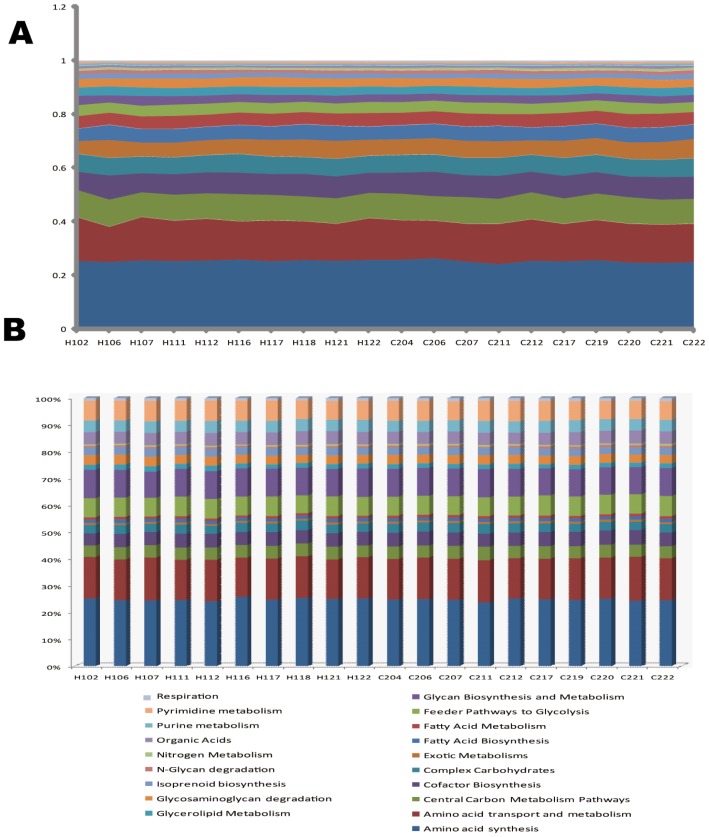
Functional patterns of the ten healthy and ten caries-active human saliva microbiota. (**A**) Relative abundance of the functional genes among the 19 gene categories on HuMiChip 1.0. (**B**) Relative diversity of the functional genes among the 19 gene categories on HuMiChip 1.0.

To interrogate microbial metabolisms in human and mouse microbiota, we developed a functional gene microarray (HuMiChip1.0) based on our well validated GeoChip 3.0 platform [15], [16], [17]. HuMiChip 1.0 contains 36,056 oligonucleotide probes targeting 139 functional genes families and covering 50,007 coding sequences from 322 draft/finished bacterial genomes and 27 shotgun metagenome datasets from various human body sites (**Table S1**; **Materials S1**). For a pilot-population of 20 human adults (whose organismal structure were decoded [9]) that included ten healthy (H Group) and ten caries-active (C Group) (**Table 1**), metabolic functions of saliva microbiota were analyzed via hybridizing the saliva DNA to the microarray. In total, 3,685 genes distributed in 19 gene categories were identified within the collection of 20 saliva microbiota. For each microbiota, the number of detected genes was 2,246∼2,880 (**Table 1**). In terms of signal intensity, gene categories such as “*Amino acid synthesis*” (accounting for ∼25% of all detected genes), “*Amino acid transport and metabolism*” (∼15%), “*Central Carbon Metabolism Pathways*” (∼10%), “*Cofactor Biosynthesis*” (∼8%) and “*Complex Carbohydrates*” (∼7%) were the most prominent across all samples (**Figure 1A**). The results indicated that the overall functional gene diversity was similar among the 20 samples (**Figure 1B**), and no significant difference in gene number or diversity indices was observed between the two groups (*p*>0.05) (**Table 1**).

**Figure 2:**
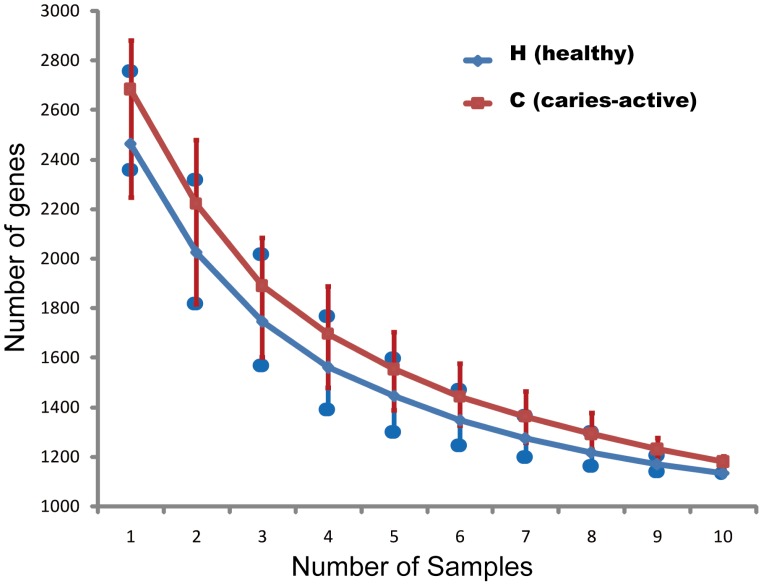
Conservation of function genes encoded in saliva microbiota among human hosts. The *x*-axis stands for the number of saliva microbiota (i.e. hosts) included. The *y*-axis is the number of shared functional genes among the hosts, representing the means of 100 iterations. Error bars represent standard deviations.

Among the detected 3,685 genes, averagely 70.86% genes (62.68%∼79.45%) were shared between any two of the ten H microbiota, and 69.47% genes (63.37%∼76.04%) were shared between any pair of the ten C microbiota (**Table S2**). The functional core, of either the H or C group, mainly consisted of “*Amino acid synthesis*” (∼25%), “*Amino acid transport and metabolism*” (∼15%) and “*Central carbon metabolism pathways*” (∼5%). The top 20 most abundant shared genes in the functional core of all 20 samples were shown in **Table S3**. The most varied functions (measured by standard deviation of signal intensity) in the functional core was “*Cytidylate kinase*” (“*Pyrimidine metabolism*”), while the most conserved (least varied in signal intensity) functions in the functional core was *“Acetyl-CoA acyltransferase anaerobic” (“Fatty Acid Metabolism”)* (**Table S4**). Inside either the H or the C group, a gradual decrease and eventual saturation of shared genes with individual additions of hosts were apparent (**Figure 2**), where 35.6% (1,134/3,187) and 35.0% (1,179/3,366) of the genes were shared for individuals within H and those within C group respectively (in contrast, only 0.08% (for C group) and 0.53% (for H group) of species-level OTUs were shared for these hosts [9]). Four functional genes were found with an “exclusive pattern” (found in either H or C triplet-features but not both): *Diaminopimelate epimerase* (“*Amino Acid Synthesis*”; C-exclusive), *Prephenate dehydrogenase* (“*Amino Acid Synthesis*”; C-exclusive), *Pyruvate-formate lyase* (“*Respiration*”; H-exclusive) and *N-acetylmuramoyl-L-alanine amidase* (“*Glycan Biosynthesis and Metabolism*”; C-exclusive).

### Distinctions in functional structure between the healthy and caries-active saliva microbiota

**Figure 3:**
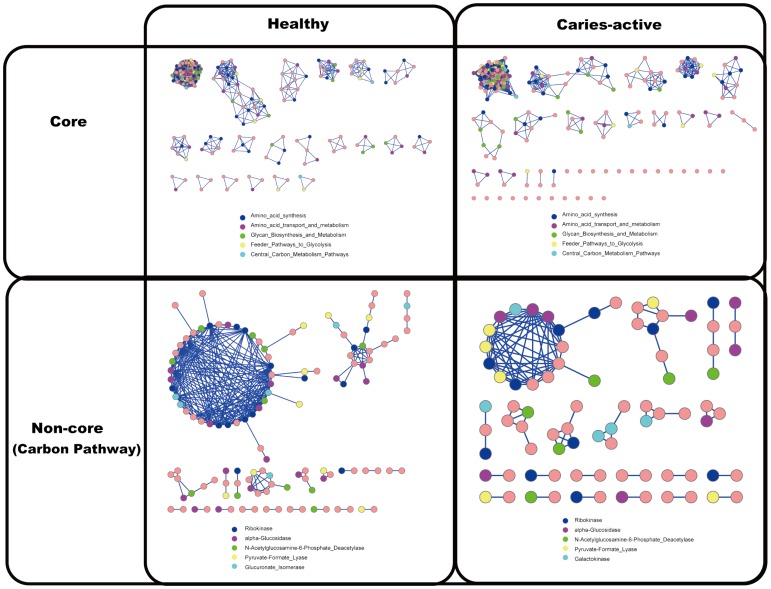
Co-presence networks of core and non-core functional genes in the healthy and caries-active host groups. A node (squares: core genes; circles: non-core genes) represents a functional gene. A solid line linking two nodes stands for positive correlation between the two. The sub-networks of Carbon-associated pathway (including ‘*Complex Carbohydrates’* , ‘*Feeder Pathways to Glycolysis’ and ‘Respiration*’) were shown. The top five most abundant gene categories (the core-genes network) or genes (the non-core-genes network) in each network were labeled with different colors.

Interestingly, functional modularity in saliva microbiota was apparent, as unveiled by the co-presence network of functional genes (i.e. among the 3,685 positive genes; **Materials and Methods**). *First*, the network of core functional gene (genes shared by every microbiota) in the H Group (the core H-network) consisted of 21 modules, with 348 nodes and 22,964 edges (links), while the core C-network consisted of 19 modules, with 363 nodes and 22,031 links (**Figure 3A**). Sizes of these modules were quite similar between the two core networks. The largest module in the core H-network consisted of 222 nodes and 22,658 links, while that in the core C-network consisted of 230 nodes and 21,800 links; furthermore, between the two modules, there was a large overlap of functional genes (96.9%∼98.3% of the genes). In fact, the nodes in the two networks displayed a similar functional pattern (**Figure 3A** and **Table S5**).

**Figure 4:**
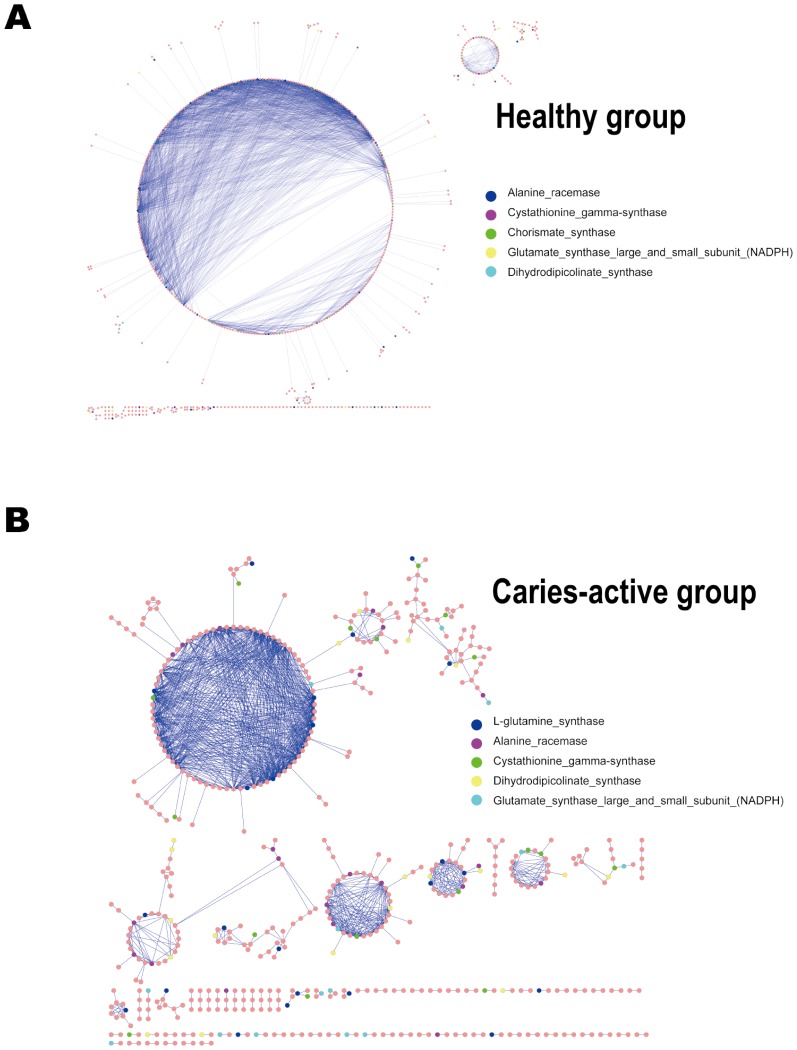
Amino acid (AA) associated gene co-presence sub-networks in the healthy and caries-active host groups. Genes in the AA-associated pathway include AA transport and metabolism and AA synthesis. The sub-networks in the H (**A**) and C groups (**B**) were shown and compared, where the largest module in the H Group consists of 337 genes yet only 74 genes were found in the largest module in the C Group. The top five most abundant genes in each network were labeled with different colors.

*Second*, sub-networks of non-core genes (genes without signals in at least one microbiota) were constructed based on specific metabolic pathways, such as the Carbon-associated, AA (amino acid)-associated and Nitrogen-associated pathways. The largest module in the Carbon-associated non-core sub-networks for H Group featured 35 genes, while that for C Group features only 14 genes (**Figure 3B**). The Amino-acids-associated non-core sub-networks exhibited a similar trend, with 337 genes in the biggest module in H Group while 74 in that of the C Group (**Figure 4**). The largest Nitrogen-metabolism-associated module was consisted of seven genes in H Group (with most encoding *Spermidine synthase*), yet was absent in C Group. Therefore, healthy saliva microbiota exhibited more conservation in non-core genes than caries-active ones. Interestingly, healthy saliva microbiota was also more conserved in organismal structure than caries-active ones [9].

### Functional gene markers of saliva microbiota that were linked to caries

**Figure 5:**
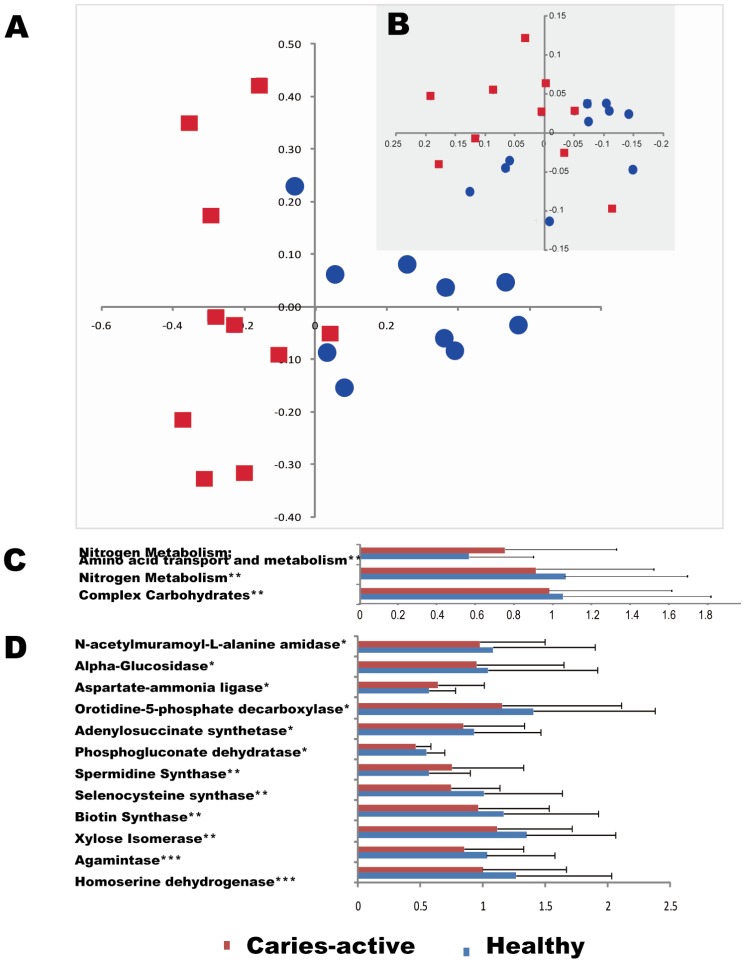
Functional-gene structure of saliva microbiota distinguishes caries-active patients from healthy hosts. (**A**) Detrended correspondence analysis (DCA) of the functional-gene structures of the 20 saliva microbiota (10 from the H and 10 from C host-groups) as defined. Normalized signal intensity data for 3,685 functional gene sequences detected in at least one of the 20 saliva microbiota were used. (**B**) Principal Coordinate Analysis (PCoA) of the organismal structures of the 20 saliva microbiota. The organismal structures were based on 16S-amplicon sequencing (adapted from [9]). Thus in this study functional-gene structure of saliva microbiota can be more sensitive than organismal structure in distinguishing caries state from healthy state. (**C**) Categories of functional genes with significant differences between the two groups. (**D**) Functional genes that were of significant differences between the two groups. Means of signal intensity for each gene (or gene category) between the two groups were compared (*p<0.1; **p<0.05, ***p<0.01; mean±s.e.m.).

Although the overall functional gene diversity of saliva microbial communities remained unchanged between the C and H groups, their composition and structure were significantly different as demonstrated by dissimilarity analysis (MRPP with *p*<0.01) and detrended correspondence analysis (DCA) from all 3,685 detected genes on HuMiChip 1.0 (**Figure 5A**), indicating a significant link between the host disease state and saliva microbiota functioning. We have previously demonstrated a high degree of divergence in organismal structure and a minimal organismal core in human saliva microbiota among host individuals [9]. Our data here showed that functional-gene structure of saliva microbiota was able to distinguish the caries state from the healthy state of human hosts. Thus a function-based strategy via HuMiChip appears to be more effective than an organism-based strategy via 16S amplicon sequencing in our case (**Figure 5B**). Therefore, functional gene structure of saliva microbiota can potentially be a more reliable predictor of caries than established risk factors such as *Streptococcus mutans* [18].

To understand the link between functional-gene structure of saliva microbiota to caries-state, signal intensities of genes and gene categories detected by HuMiChip were compared between the two groups of hosts. Significant differences were detected for gene categories of *Complex carbohydrates*, *Nitrogen metabolisms* and *Amino acid transport and metabolism* (**Figure 5C**), and for functional genes such as *Xylose isomerase*, *N-acetylmuramoyl-L-alanine amidase*, *Alpha-glucosidase*, etc (**Figure 5D**). Through a “feature selection” strategy (**Materials and Methods**) based on the 2,822 non-core functional genes, 1,247 triplet features were selected whose accuracy was at least 80% each among all possible permutations (**Table S6**). Among them, eight triplet-features were identified with high predictive power for H Group, and nine triplet-features for C Group (**Table 2**). These 17 triplet-feature sets thus represented salivary microbial gene markers that were of value in dissecting and diagnosing caries etiology.

**Table 2:** Microbial functional markers that could potentially distinguish caries-active saliva microbiota from healthy ones.

	Gene name	Gene category	Genbank ID
**Healthy**			
1	*Pyruvate-Formate Lyase*	*Respiration*	fig_4440944.3.peg.10065
	*Cytosine deaminase*	*Pyrimidine metabolism*	259506366
	*Glutamate synthase large and small subunit (NADPH)*	*Amino acid synthesis*	fig_4440823.3.peg.83433
2	*Phosphoribosyl glycinamide synthetase phosphoribosylamine-glycine ligase*	*Purine metabolism*	258543886
	*Pyruvate-Formate Lyase*	*Respiration*	fig_4440944.3.peg.10065
	*Beta-N-acetyl-D-hexosaminide N-acetylhexosaminohydrolase*	*Glycosaminoglycan degradation*	160942077
3	*Cytosine deaminase*	*Pyrimidine metabolism*	fig_4440823.3.peg.212847
	*Branched-chain-amino-acid transaminase*	*Amino acid transport and metabolism*	255323153
	*Mannanase (beta-mannosidase)*	*Complex Carbohydrates*	fig_4440943.3.peg.37808
4	*Agamintase*	*Nitrogen Metabolism*	fig_4440824.3.peg.113687
	*3-isopropylmalate dehydrogenase*	*Amino acid synthesis*	fig_4440452.7.peg.65716
	*Diaminopimelate epimerase*	*Amino acid synthesis*	fig_4440824.3.peg.204352
5	*Diaminopimelate epimerase*	*Amino acid synthesis*	fig_4440824.3.peg.204352
	*Methylmalonyl-CaA decarboxylase*	*Organic Acids*	fig_4440825.3.peg.10215
	*Alpha-Glucosidase*	*Complex Carbohydrates*	225570901
6	*Diaminopimelate epimerase*	*Amino acid synthesis*	fig_4440824.3.peg.204352
	*Agamintase*	*Nitrogen Metabolism*	fig_4440824.3.peg.4961
	*Cytosine deaminase*	*Pyrimidine metabolism*	229828779
7	*Dihydrodipicolinate synthase*	*Amino acid synthesis*	fig_4440613.3.peg.149199
	*Cystathionine gamma-synthase*	*Amino acid synthesis*	fig_4440461.5.peg.1268
	*Uridine phosphorylase*	*Pyrimidine metabolism*	154495787
8	*Beta-D-glucuronidase*	*Glycan structures - degradation;Exotic Metabolisms*	fig_4440946.3.peg.10812
	*Pyruvate-Formate Lyase*	*Respiration*	fig_4440944.3.peg.10065
	*Phosphoribosyl glycinamide synthetase phosphoribosylamine-glycine ligase*	*Purine metabolism*	258543886
***Caries-active***			
1	*3-demethylubiquinone-9 3-methyltransferase*	*Cofactor Biosynthesis*	241759137
	*Prephenate dehydrogenase*	*Amino acid synthesis*	fig_4440943.3.peg.33989
	*Cysteine synthase A*	*Amino acid synthesis*	241760414
2	*Pyruvate-Formate Lyase*	*Respiration*	fig_4440944.3.peg.10065
	*N-acetylmuramoyl-L-alanine amidase*	*Glycan Biosynthesis and Metabolism*	218128624
	*Beta-D-glucuronidase*	*Glycan structures - degradation;Exotic Metabolisms*	218258198
3	*Geranyltranstransferase*	*Isoprenoid biosynthesis*	237749332
	*Cytosine deaminase*	*Pyrimidine metabolism*	229828779
	*Diaminopimelate epimerase*	*Amino acid synthesis*	fig_4440824.3.peg.204352
4	*Carbon Monoxide Dehydrogenase*	*Central Carbon Metabolism Pathways*	167748998
	*Prephenate dehydrogenase*	*Amino acid synthesis*	fig_4440943.3.peg.33989
	*Beta-ketoacyl-acyl-carrier-protein synthase III*	*Fatty Acid Biosynthesis*	227895174
5	*Prephenate dehydrogenase*	*Amino acid synthesis*	fig_4440943.3.peg.33989
	*Cysteine synthase A*	*Amino acid synthesis*	241760414
	*Cysteine synthase A*	*Amino acid synthesis*	209907778
6	*Branched-chain-amino-acid transaminase*	*Amino acid transport and metabolism*	fig_4440610.3.peg.10420
	*Pyruvate-Formate Lyase*	*Respiration*	fig_4440944.3.peg.10065
	*Adenylosuccinate synthetase*	*Purine metabolism*	fig_4440949.3.peg.18491
7	*Dihydrodipicolinate synthase*	*Amino acid synthesis*	229496628
	*Prephenate dehydrogenase*	*Amino acid synthesis*	fig_4440943.3.peg.33989
	*Arginosuccinate synthase*	*Amino acid synthesis*	fig_4440939.3.peg.8046
8	*N-acetylmuramoyl-L-alanine amidase*	*Glycan Biosynthesis and Metabolism*	218128624
	*Quinolinate Synthase*	*Cofactor Biosynthesis*	227547856
	*Pyruvate-Formate Lyase*	*Respiration*	fig_4440944.3.peg.10065
9	*Pyridoxal Kinase*	*Cofactor Biosynthesis*	fig_4440942.3.peg.3331
	*Serine hydroxymethyltransferase*	*Amino acid synthesis*	225574091
	*Prephenate dehydrogenase*	*Amino acid synthesis*	fig_4440943.3.peg.33989

Each biomarker is a triplet-feature set of microbial genes.

Interestingly, those genes presented with the highest frequency in the 17 triplet-features were those that exhibited an “exclusive pattern” (found in either H or C triplet-features but not in both): *Diaminopimelate epimerase* (“*Amino Acid Synthesis*”; C-exclusive), *Prephenate dehydrogenase* (“*Amino Acid Synthesis*”; C-exclusive), *Pyruvate-formate lyase* (“*Respiration*”; H-exclusive) and *N-acetylmuramoyl-L-alanine amidase* (“*Glycan Biosynthesis and Metabolism*”; C-exclusive). In contrast, for these 20 saliva microbiota, not a single taxon, from the phylogenetic level of phylum to that of OTUs (totally 9,628 OTU found), was identified with such an “exclusive pattern” of distribution in either the H or C Group [9], suggesting function-based strategies can potentially be more effective than organism-based ones in diagnosis and treatment of oral infectious diseases.

## Discussion

There has been a long history in sialometry and sialochemistry diagnosis of both oral and systemic diseases, such as caries [19], primary Sjogren's Syndrome [20], oral squamous cell carcinoma [21] and pancreatic diseases [22]. For caries, previous works in saliva have mainly focused on human-host attributes such as *Glucosyltransferase B* [23], antimicrobial peptides [24], past caries experience [25], soluble CD14 [26] and trace elements [27], while only a few have exploited individual microbial features, such as specific microbiological counts [28] and microbial nitrate reductase activities [29]. Few global functional analysis and comparison of saliva microbiota function was available, due to (i) the organismal complexity of the microbiota [9] and (ii) the observations that metagenome-sequencing based functional comparison of microbiota can be hampered by sequencing biases [30], the paucity of reference genomes and the small percentage of annotatable reads [31], [32].

Microarray-based technologies are generally robust for community comparisons and more resistant to contaminants [33]. Therefore, we developed a functional gene microarray (HuMiChip1.0) to interrogate microbial metabolism in human and mouse microbiota. This comprehensive survey of saliva microbiota functions on the 10 healthy and 10 caries-active adults suggested that saliva microbiota carried disease-associated functional signatures.

The global functional landscapes of saliva microbiota in healthy and diseased hosts revealed a series of microbial functional markers strongly linked to caries in the pilot populations. Most of these microbial markers were novel and could lead to new clinical applications once validated in larger cohorts. One class of them was affiliated with *Amino acid synthesis*, suggesting the close link between the microbial activity and caries. *Diaminopimelate epimerase* is central to the biosynthesis of both lysine and cell-wall peptidoglycan in many bacteria. It catalyzes the stereoinversion of LL-DAP to meso-DAP, a precursor of L-lysine and an essential component of bacterial peptidoglycans [34]. *Prephenate dehydrogenase* (PDH) is a bacterial enzyme that converts prephenate to 4-hydroxyphenylpyruvate through the oxidative decarboxylation pathway for tyrosine biosynthesis [34]. *Aspartate–ammonia ligase* (*Asparagine synthetase*) catalyses the conversion of L-aspartate to L-asparagine in the presence of ATP and ammonia. These findings were consistent with previous works linking compounds with amine functional groups to caries (such as association of free salivary proline and glycine with caries [35]) and reporting higher levels of free salivary arginine and lysine in caries-free adults than those with caries history [36]). Microbial catabolism of dibasic amino acids might contribute to neutralization of plaque acids and thus partially accounted for the higher resting plaque pH in caries-free hosts [37].

Another class of candidate caries biomarkers we identified was consisted of those involved in carbohydrate hydrolysis. *Pyruvate formate-lyase* (PFL), exclusively existent in the H Group, converts sugar into volatile compounds (formate, acetate and ethanol) and serves in ATP synthesis and NAD+/NADH recycling [38]. This enzyme is extremely sensitive to oxygen and can be key to anaerobic fermentation in dental plaques [39]. *N-acetylmuramoyl-L-alanine amidase* is an autolytic enzyme bound to the surface of bacterial cell walls. It hydrolyzes the link between N-acetylmuramoyl residues and L-amino acid residues in certain cell wall glycopeptides (particularly peptidoglycan). It was reported that mutanolysin, one of the petidoglycan-degradative enzymes, exhibited lytic activity against the “etiologic agents” of dental caries, e.g. *Streptococcus mutans*, *Streptococcus salivarius*, *Streptococcus sanguis*, *Lactobacillus acidophilus* and *Actinomyces viscosus* [40]. *Alpha-glucosidase* is hypothesized to participate in the induction of dental caries [41]. *Alpha-glucosidase* and *Glucosyltransferases* (Gtfs) are both from GH13 family (Glycoside Hydrolase Family 13; http://www.cazy.org); *Gtfs* are a major virulence factor in caries-pathogens in that *Gtfs* adsorb to enamel and synthesize extracellular glucans *in situ*, providing sites for colonization by microbes and an insoluble matrix for plaque [42]. *Xylose isomerase* is a key enzyme in xylose to xylitol conversion, which is carried out by bacteria. Xylitol has been recommended for its positive caries-prevention effect, demonstrated in various clinical trials using xylitol-containing chewing gum [43].

Microarray-based technology has served as useful tools for sensitive, specific, and quantitative analysis of microbial communities, yet their limitations in dissecting the functional composition of complex microbial communities still remain. For example, functional features that can be revealed were dependent on the defined probe sets with known functions. With the development of high-throughput sequencing, the number of functional gene sequences of interest has been increasing rapidly, thus the probes must be continuously updated and improved for comprehensive analysis [44].

In summary, our work unveiled the global functional features of human saliva microbiota. The sensitivity to host disease state, links to systematic body functions (due to circulation; [45], [46]), easy accessibility and non-invasiveness in sampling, susceptibility for *in situ* analysis, feasibility of genotyping microbiota, as well as the extensive clinical knowledge base [47] and accumulating saliva-omics data (e.g. the salivary proteome [48]) suggest saliva as an advantageous venue and valuable research model for tracking intricate interactions that underlie oral and even systemic diseases.

## Supporting Information

Figure S1 Computational strategy for selecting the functional-gene markers associated with caries in saliva microbiota.
(TIF)

Table S1 Probes and coding sequences on HuMiChip 1.0.
(DOCX)

Table S2 The percentages of microbial genes detected by HuMiChip 1.0 that are shared between any pair of microbiota from the 20 saliva microbiota.
(DOCX)

Table S3 The top 20 most abundant genes in the functional core of the 20 saliva microbiota.
(DOCX)

Table S4 The most conserved and variable genes (in signal intensity) in the functional cores of the 20 saliva microbiota.
(DOCX)

Table S5 Distribution of the functional-core genes in the healthy and caries-active microbiota. Genes in both H and C groups were shown.
(DOCX)

Table S6 Triplet feature set with high prediction power for healthy and caries states of the hosts.
(DOCX)

Materials S1 Supplementary materials.
(DOC)

## References

[1] Qi X (2008) The Third National Sampling Epidemiological Survey on Oral Health.

[2] Kanasi E, Johansson I, Lu SC, Kressin NR, Nunn ME, et al (2010) Microbial risk markers for childhood caries in pediatricians' offices. J Dent Res 89: 378–383. 20164496 10.1177/0022034509360010 PMC2880172

[3] Keijser BJ, Zaura E, Huse SM, van der Vossen JM, Schuren FH, et al (2008) Pyrosequencing analysis of the oral microflora of healthy adults. J Dent Res 87: 1016–1020. 18946007 10.1177/154405910808701104

[4] Lazarevic V, Whiteson K, Huse S, Hernandez D, Farinelli L, et al (2009) Metagenomic study of the oral microbiota by Illumina high-throughput sequencing. J Microbiol Methods 79: 266–271. 19796657 10.1016/j.mimet.2009.09.012 PMC3568755

[5] Nasidze I, Quinque D, Li J, Li M, Tang K, et al (2009) Comparative analysis of human saliva microbiome diversity by barcoded pyrosequencing and cloning approaches. Anal Biochem 391: 64–68. 19406095 10.1016/j.ab.2009.04.034

[6] Zaura E, Keijser BJ, Huse SM, Crielaard W (2009) Defining the healthy “core microbiome” of oral microbial communities. BMC Microbiol 9: 259. 20003481 10.1186/1471-2180-9-259 PMC2805672

[7] Lazarevic V, Whiteson K, Hernandez D, Francois P, Schrenzel J (2010) Study of inter- and intra-individual variations in the salivary microbiota. BMC Genomics 11: 523. 20920195 10.1186/1471-2164-11-523 PMC2997015

[8] Ling Z, Kong J, Jia P, Wei C, Wang Y, et al (2010) Analysis of oral microbiota in children with dental caries by PCR-DGGE and barcoded pyrosequencing. Microb Ecol 60: 677–690. 20614117 10.1007/s00248-010-9712-8

[9] Yang F, Zeng X, Ning K, Liu KL, Lo CC, et al (2012) Saliva microbiomes distinguish caries-active from healthy human populations. ISME J 6: 1–10. 21716312 10.1038/ismej.2011.71 PMC3246229

[10] Huang S, Yang F, Zeng X, Chen J, Li R, et al (2011) Preliminary characterization of the oral microbiota of Chinese adults with and without gingivitis. BMC Oral Health 11: 33. 22152152 10.1186/1472-6831-11-33 PMC3254127

[11] Griffen AL, Beall CJ, Campbell JH, Firestone ND, Kumar PS, et al (2011) Distinct and complex bacterial profiles in human periodontitis and health revealed by 16S pyrosequencing. ISME J 6.1176–1185. 10.1038/ismej.2011.191 PMC3358035 22170420

[12] Structure, function and diversity of the healthy human microbiome. Nature 486: 207–214. 22699609 10.1038/nature11234 PMC3564958

[13] He Z, Deng Y, Van Nostrand JD, Tu Q, Xu M, et al (2010) GeoChip 3.0 as a high-throughput tool for analyzing microbial community composition, structure and functional activity. ISME J 4: 1167–1179. 20428223 10.1038/ismej.2010.46

[14] Quinque D, Kittler R, Kayser M, Stoneking M, Nasidze I (2006) Evaluation of saliva as a source of human DNA for population and association studies. Anal Biochem 353: 272–277. 16620753 10.1016/j.ab.2006.03.021

[15] He Z, Deng Y, Van Nostrand JD, Tu Q, Xu M, et al (2010) GeoChip 3.0 as a high-throughput tool for analyzing microbial community composition, structure and functional activity. ISME J 4: 1167–1179. 20428223 10.1038/ismej.2010.46

[16] Hazen TC, Dubinsky EA, DeSantis TZ, Andersen GL, Piceno YM, et al (2010) Deep-sea oil plume enriches indigenous oil-degrading bacteria. Science 330: 204–208. 20736401 10.1126/science.1195979

[17] Lu Z, Deng Y, Van Nostrand JD, He Z, Voordeckers J, et al (2012) Microbial gene functions enriched in the Deepwater Horizon deep-sea oil plume. ISME J 6: 451–460. 21814288 10.1038/ismej.2011.91 PMC3260509

[18] Jiang Q, Yu M, Min Z, Yi A, Chen D, et al (2012) AP-PCR detection of Streptococcus mutans and Streptococcus sobrinus in caries-free and caries-active subjects. Mol Cell Biochem 10.1007/s11010-012-1255-5 22407567

[19] Thomadaki K, Helmerhorst EJ, Tian N, Sun X, Siqueira WL, et al (2011) Whole-saliva proteolysis and its impact on salivary diagnostics. J Dent Res 90: 1325–1330. 21917601 10.1177/0022034511420721 PMC3188460

[20] Baldini C, Giusti L, Ciregia F, Da Valle Y, Giacomelli C, et al (2011) Proteomic analysis of saliva: a unique tool to distinguish primary Sjogren's syndrome from secondary Sjogren's syndrome and other sicca syndromes. Arthritis Res Ther 13: R194. 22117835 10.1186/ar3523 PMC3334644

[21] Brinkmann O, Kastratovic DA, Dimitrijevic MV, Konstantinovic VS, Jelovac DB, et al (2011) Oral squamous cell carcinoma detection by salivary biomarkers in a Serbian population. Oral Oncol 47: 51–55. 21109482 10.1016/j.oraloncology.2010.10.009 PMC3032819

[22] Farrell JJ, Zhang L, Zhou H, Chia D, Elashoff D, et al (2011) Variations of oral microbiota are associated with pancreatic diseases including pancreatic cancer. Gut 10.1136/gutjnl-2011-300784 PMC3705763 21994333

[23] Vacca Smith AM, Scott-Anne KM, Whelehan MT, Berkowitz RJ, Feng C, et al (2007) Salivary glucosyltransferase B as a possible marker for caries activity. Caries Res 41: 445–450. 17827962 10.1159/000107930 PMC2820324

[24] Tao R, Jurevic RJ, Coulton KK, Tsutsui MT, Roberts MC, et al (2005) Salivary antimicrobial peptide expression and dental caries experience in children. Antimicrob Agents Chemother 49: 3883–3888. 16127066 10.1128/AAC.49.9.3883-3888.2005 PMC1195389

[25] Zhang Q, Bian Z, Fan M, van Palenstein Helderman WH (2007) Salivary mutans streptococci counts as indicators in caries risk assessment in 6–7-year-old Chinese children. J Dent 35: 177–180. 16949192 10.1016/j.jdent.2006.07.004

[26] Bergandi L, Defabianis P, Re F, Preti G, Aldieri E, et al (2007) Absence of soluble CD14 in saliva of young patients with dental caries. Eur J Oral Sci 115: 93–96. 17451497 10.1111/j.1600-0722.2007.00437.x

[27] Zahir S, Sarkar S (2006) Study of trace elements in mixed saliva of caries free and caries active children. J Indian Soc Pedod Prev Dent 24: 27–29. 16582528 10.4103/0970-4388.22832

[28] Martinez-Pabon MC, Ramirez-Puerta BS, Escobar-Paucar GM, Franco-Cortes AM (2010) Physicochemical salivary properties, Lactobacillus, mutans streptococci counts and early childhood caries in preschool children of Colombia. Acta Odontol Latinoam 23: 249–256. 21638968

[29] Doel JJ, Hector MP, Amirtham CV, Al-Anzan LA, Benjamin N, et al (2004) Protective effect of salivary nitrate and microbial nitrate reductase activity against caries. Eur J Oral Sci 112: 424–428. 15458501 10.1111/j.1600-0722.2004.00153.x

[30] Morgan JL, Darling AE, Eisen JA (2010) Metagenomic sequencing of an in vitro-simulated microbial community. PLoS One 5: e10209. 20419134 10.1371/journal.pone.0010209 PMC2855710

[31] Fodor AA, DeSantis TZ, Wylie KM, Badger JH, Ye Y, et al (2012) The “most wanted” taxa from the human microbiome for whole genome sequencing. PLoS One 7: e41294. 22848458 10.1371/journal.pone.0041294 PMC3406062

[32] Xie G, Chain PS, Lo CC, Liu KL, Gans J, et al (2010) Community and gene composition of a human dental plaque microbiota obtained by metagenomic sequencing. Mol Oral Microbiol 25: 391–405. 21040513 10.1111/j.2041-1014.2010.00587.x PMC2975940

[33] He Z, Van Nostrand JD, Zhou J (2012) Applications of functional gene microarrays for profiling microbial communities. Curr Opin Biotechnol 10.1016/j.copbio.2011.12.021 22226464

[34] Ku HK, Do NH, Song JS, Choi S, Yeon SH, et al (2011) Crystal structure of prephenate dehydrogenase from Streptococcus mutans. Int J Biol Macromol 49: 761–766. 21798280 10.1016/j.ijbiomac.2011.07.009

[35] Fonteles CS, Guerra MH, Ribeiro TR, Mendonca DN, de Carvalho CB, et al (2009) Association of free amino acids with caries experience and mutans streptococci levels in whole saliva of children with early childhood caries. Arch Oral Biol 54: 80–85. 18774124 10.1016/j.archoralbio.2008.07.011

[36] Van Nieuw Amerongen A, Bolscher JG, Veerman EC (2004) Salivary proteins: protective and diagnostic value in cariology? Caries Res 38: 247–253. 15153696 10.1159/000077762

[37] Van Wuyckhuyse BC, Perinpanayagam HE, Bevacqua D, Raubertas RF, Billings RJ, et al (1995) Association of free arginine and lysine concentrations in human parotid saliva with caries experience. J Dent Res 74: 686–690. 7722066 10.1177/00220345950740021001

[38] Yamamoto Y, Sato Y, Takahashi-Abbe S, Takahashi N, Kizaki H (2000) Characterization of the Streptococcus mutans pyruvate formate-lyase (PFL)-activating enzyme gene by complementary reconstitution of the In vitro PFL-reactivating system. Infect Immun 68: 4773–4777. 10899886 10.1128/iai.68.8.4773-4777.2000 PMC98435

[39] Takahashi-Abbe S, Abe K, Takahashi N (2003) Biochemical and functional properties of a pyruvate formate-lyase (PFL)-activating system in Streptococcus mutans. Oral Microbiol Immunol 18: 293–297. 12930520 10.1034/j.1399-302x.2003.00081.x

[40] Thanyasrisung P, Komatsuzawa H, Yoshimura G, Fujiwara T, Yamada S, et al (2009) Automutanolysin disrupts clinical isolates of cariogenic streptococci in biofilms and planktonic cells. Oral Microbiol Immunol 24: 451–455. 19832796 10.1111/j.1399-302X.2009.00536.x

[41] Mormann JE, Schmid R, Muhlemann HR (1983) Effect of alpha-amylase and alpha-glucosidase inhibitors on caries incidence and plaque accumulation in rats. Caries Res 17: 353–356. 6191863 10.1159/000260687

[42] Bowen WH, Koo H (2011) Biology of Streptococcus mutans-derived glucosyltransferases: role in extracellular matrix formation of cariogenic biofilms. Caries Res 45: 69–86. 10.1159/000324598 PMC3068567 21346355

[43] Lif Holgerson P, Stecksen-Blicks C, Sjostrom I, Twetman S (2005) Effect of xylitol-containing chewing gums on interdental plaque-pH in habitual xylitol consumers. Acta Odontol Scand 63: 233–238. 16040446 10.1080/00016350510019883

[44] He Z, Deng Y, Zhou J (2012) Development of functional gene microarrays for microbial community analysis. Curr Opin Biotechnol 23: 49–55. 22100036 10.1016/j.copbio.2011.11.001

[45] Mirzaii-Dizgah I, Riahi E (2011) Serum and saliva levels of cathepsin L in patients with acute coronary syndrome. J Contemp Dent Pract 12: 114–119. 22186754 10.5005/jp-journals-10024-1019

[46] Xiao H, Zhang L, Zhou H, Lee JM, Garon EB, et al (2011) Proteomic analysis of human saliva from lung cancer patients using two-dimensional difference gel electrophoresis and mass spectrometry. Mol Cell Proteomics 10.1074/mcp.M111.012112 PMC3277759 22096114

[47] Baum BJ, Yates JR 3rd, Srivastava S, Wong DT, Melvin JE (2011) Scientific frontiers: emerging technologies for salivary diagnostics. Adv Dent Res 23: 360–368. 21917746 10.1177/0022034511420433 PMC3172997

[48] Denny P, Hagen FK, Hardt M, Liao L, Yan W, et al (2008) The proteomes of human parotid and submandibular/sublingual gland salivas collected as the ductal secretions. J Proteome Res 7: 1994–2006. 18361515 10.1021/pr700764j PMC2839126

