# Supplementary figures and images for: Saliva Microbiota Carry Caries-Specific Functional Gene Signatures
Source: PLoS One. 2014 Feb 12;9(2):e76458. doi: 10.1371/journal.pone.0076458 (PMC3922703; doi:10.1371/journal.pone.0076458)

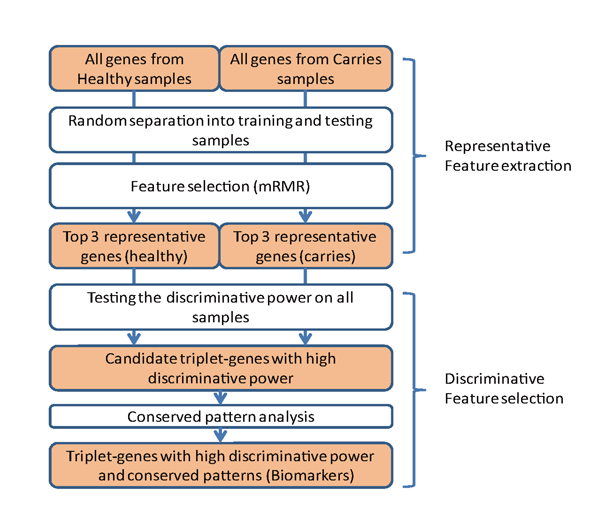

Supplement: Figure S1 — Computational strategy for selecting the functional-gene markers associated with caries in saliva microbiota. (TIF) [file pone.0076458.s001.tif]
