# Supplementary material for: Saliva Microbiota Carry Caries-Specific Functional Gene Signatures
Source: PLoS One. 2014 Feb 12;9(2):e76458. doi: 10.1371/journal.pone.0076458 (PMC3922703; doi:10.1371/journal.pone.0076458)
Supplement: Table S1 — Probes and coding sequences on HuMiChip 1.0. (DOCX) [file pone.0076458.s002.docx]

**Table S1. Probes and coding sequences on HuMiChip 1.0.**

|  | **Num. genes** | **Num. probes** | **Num.**  **sequence-specific probes** | **Num.**  **group-specific**  **probes** | **Num.**  **covered CDS** |
| --- | --- | --- | --- | --- | --- |
| **Amino acid metabolism** | 83 | 14,783 | 6,835 | 21,681 | 29,838 |
| **Carbohydrate metabolism** | 36 | 6,539 | 3,256 | 9,795 | 12,544 |
| **Energy metabolism** | 14 | 3,292 | 1,700 | 4,992 | 6,359 |
| **Glycan biosynthesis and metabolism** | 15 | 4,682 | 2,234 | 6,916 | 8,346 |
| **Lipid metabolism** | 6 | 1,585 | 830 | 2,415 | 2,905 |
| **Metabolism of cofactors and vitamins** | 17 | 2,680 | 1,217 | 3,897 | 4,999 |
| **Metabolism of terpenoids and polyketides** | 5 | 1,247 | 594 | 1,841 | 2,517 |
| **Nucleotide metabolism** | 13 | 3,013 | 1,424 | 4,437 | 6,421 |
| **Total*** | 139 | 36,056 | 24,188 | 11,868 | 50,007 |

*Gene families targeting human microbiome were selected from KEGG pathway database, and may participate in multiple pathways. The total number of probes and covered coding sequences were based on non-redundant genes included in all pathways, i.e. they were not the sum of all sub-categories.
