# Supplementary material for: Saliva Microbiota Carry Caries-Specific Functional Gene Signatures
Source: PLoS One. 2014 Feb 12;9(2):e76458. doi: 10.1371/journal.pone.0076458 (PMC3922703; doi:10.1371/journal.pone.0076458)
Supplement: Table S2 — The percentages of microbial genes detected by HuMiChip 1.0 that are shared between any pair of microbiota from the 20 saliva microbiota. (DOCX) [file pone.0076458.s003.docx]

**Table S2. The percentages of microbial genes detected by HuMiChip 1.0 that are shared between any pair of microbiota from the 20 saliva microbiota.**
