# Supplementary material for: Saliva Microbiota Carry Caries-Specific Functional Gene Signatures
Source: PLoS One. 2014 Feb 12;9(2):e76458. doi: 10.1371/journal.pone.0076458 (PMC3922703; doi:10.1371/journal.pone.0076458)
Supplement: Table S3 — The top 20 most abundant genes in the functional core of the 20 saliva microbiota. (DOCX) [file pone.0076458.s004.docx]

**Table S3. The top 20 most abundant genes in the functional core of the all 20 saliva microbiota.**

| **Gene name** | **Gene category** |
| --- | --- |
| *Beta-D-galactosidase* | *Amino acid synthesis* |
| *Transketolase* | *Glycan Biosynthesis and Metabolism* |
| *Ribokinase* | *Amino acid synthesis* |
| *Alanine racemase* | *Glycan structures - degradation;Complex Carbohydrates* |
| *Dihydrodipicolinate synthase* | *Amino acid synthesis* |
| *N-Acetylglucosamine-6-Phosphate Deacetylase* | *Amino acid synthesis* |
| *UDP-N-acetylmuramateL-alanine ligase* | *Amino acid synthesis* |
| *Alpha-Glucosidase* | *Feeder Pathways to Glycolysis* |
| *Beta-N-acetyl-D-hexosaminide N-acetylhexosaminohydrolase* | *Glycan Biosynthesis and Metabolism* |
| *Glycerol Kinase* | *Amino acid transport and metabolism* |
| *UDP-N-acetylmuramoyl-L-alanineD-glutamate ligase* | *Pyrimidine metabolism* |
| *UDP-N-acetylmuramoyl-L-alanyl-D-glutamatemeso-diaminopimelate ligase* | *Amino acid synthesis* |
| *Beta-ketoacyl-acyl-carrier-protein synthase III* | *Fatty Acid Biosynthesis* |
| *Serine O-acetyltransferase* | *Pyrimidine metabolism* |
| *3-deoxy-7-phosphoheptulonate synthase* | *Amino acid transport and metabolism* |
| *4-diphosphocytidyl-2C-methyl-D-erythritol synthase* | *Isoprenoid biosynthesis* |
| *Aspartate kinase* | *Amino acid synthesis* |
| *Diaminopimelate epimerase* | *Amino acid synthesis* |
| *Methylmalonyl-CaA decarboxylase* | *Glycerolipid Metabolism* |
| *Thioredoxin reductase FAD-NADP-binding* | *Amino acid synthesis* |
