# Supplementary material for: Saliva Microbiota Carry Caries-Specific Functional Gene Signatures
Source: PLoS One. 2014 Feb 12;9(2):e76458. doi: 10.1371/journal.pone.0076458 (PMC3922703; doi:10.1371/journal.pone.0076458)
Supplement: Table S4 — The most conserved and variable genes (in signal intensity) in the functional cores of the 20 saliva microbiota. (DOCX) [file pone.0076458.s005.docx]

**Table S4. The most conserved and variable genes (in signal intensity) in the functional cores of all the 20 saliva microbiota.**

|  | **Gene name** | **Gene category** |
| --- | --- | --- |
| **Most conversed** | *Acetyl-CoA acyltransferase anaerobic* | *Fatty Acid Metabolism* |
|  | *UDP-N-acetylmuramateL-alanine ligase* | *Glycan Biosynthesis and Metabolism* |
|  | *Dihydrodipicolinate synthase* | *Amino acid synthesis* |
|  | *Selenocysteine lyase PLP-dependent* | *Amino acid synthesis* |
|  | *Acetyl-CoA acyltransferase anaerobic* | *Fatty Acid Metabolism* |
|  | *Thioredoxin reductase FAD-NADP-binding* | *Pyrimidine metabolism* |
|  | *Serine hydroxymethyltransferase* | *Amino acid synthesis* |
|  | *Arabinose Isomerase* | *Feeder Pathways to Glycolysis* |
|  | *Ribokinase* | *Feeder Pathways to Glycolysis* |
|  | *Mannanase (beta-mannosidase)* | *Complex Carbohydrates* |
| **Most variable** | *Cytidylate kinase* | *Pyrimidine metabolism* |
|  | *Beta-D-galactosidase* | *Glycan structures - degradation;Complex Carbohydrates* |
|  | *Methylmalonyl-CaA decarboxylase* | *Organic Acids* |
|  | *Thioredoxin reductase FAD-NADP-binding* | *Pyrimidine metabolism* |
|  | *Alanine racemase* | *Amino acid synthesis* |
|  | *L-threonine synthase* | *Amino acid synthesis* |
|  | *Selenocysteine lyase PLP-dependent* | *Amino acid synthesis* |
|  | *Uridine phosphorylase* | *Pyrimidine metabolism* |
|  | *Purine-nucleoside phosphorylase* | *Purine metabolism* |
|  | *2-isopropylmalate synthase* | *Amino acid synthesis* |
