# Supplementary material for: Saliva Microbiota Carry Caries-Specific Functional Gene Signatures
Source: PLoS One. 2014 Feb 12;9(2):e76458. doi: 10.1371/journal.pone.0076458 (PMC3922703; doi:10.1371/journal.pone.0076458)
Supplement: Table S5 — Distribution of the functional-core genes in the healthy and caries-active microbiota. Genes in both H and C groups were shown. (DOCX) [file pone.0076458.s006.docx]

**Table S5. Distribution of the functional-core genes in the healthy and caries-active microbiota.** Genes in both H and C groups were shown.

| **Gene category** | **Health** | **Caries-active** |
| --- | --- | --- |
| *Purine metabolism* | 45 | 45 |
| *Amino acid synthesis* | 281 | 282 |
| *Amino acid transport and metabolism* | 169 | 176 |
| *Pyrimidine metabolism* | 70 | 76 |
| *Glycan Biosynthesis and Metabolism* | 107 | 110 |
| *Feeder Pathways to Glycolysis* | 85 | 87 |
| *Cofactor Biosynthesis* | 44 | 55 |
| *N-Glycan degradation* | 9 | 5 |
| *Glycosaminoglycan degradation* | 34 | 32 |
| *Central Carbon Metabolism Pathways* | 53 | 62 |
| *Nitrogen Metabolism* | 7 | 8 |
| *Complex Carbohydrates* | 44 | 40 |
| *Isoprenoid biosynthesis* | 31 | 33 |
| *Organic Acids* | 52 | 55 |
| *Glycan structures - degradation;Complex Carbohydrates* | 28 | 33 |
| *Glycerolipid Metabolism* | 19 | 18 |
| *Respiration* | 11 | 9 |
| *Fatty Acid Metabolism* | 9 | 10 |
| *Exotic Metabolisms* | 9 | 9 |
| *Fatty Acid Biosynthesis* | 13 | 19 |
| *Glycan structures - degradation;Exotic Metabolisms* | 9 | 11 |
| *Amino acid synthesis;amino acid synthesis* | 2 | 2 |
| *Feeder Pathways to Glycolysis;Central Carbon Metabolism Pathways* | 3 | 2 |
| *Nitrogen Metabolism;Amino acid transport and metabolism* | 0 | 0 |
