# Supplementary material for: Saliva Microbiota Carry Caries-Specific Functional Gene Signatures
Source: PLoS One. 2014 Feb 12;9(2):e76458. doi: 10.1371/journal.pone.0076458 (PMC3922703; doi:10.1371/journal.pone.0076458)
Supplement: Table S6 — Triplet feature set with high prediction power for healthy and caries states of the hosts. (DOCX) [file pone.0076458.s007.docx]

**Table S6. Triplet feature set with high prediction power for healthy and caries states of the hosts.**

|  | | **Gene name** | | **Gene category** | |
| --- | --- | --- | --- | --- | --- |
| 1 | | *UDP-N-acetylglucosamine acyltransferase* | | *Glycan Biosynthesis and Metabolism* | |
|  | | *N-acetylmuramoyl-L-alanine amidase* | | *Glycan Biosynthesis and Metabolism* | |
|  | | *Alanine racemase* | | *Amino acid synthesis* | |
| 2 | | *Pyruvate-Formate Lyase* | | *Respiration* | |
|  | | *Beta-D-glucuronidase* | | *Glycan structures - degradation;Exotic Metabolisms* | |
|  | | *Alpha-Glucosidase* | | *Complex Carbohydrates* | |
| 3 | | *IMP dehydrogenase* | | *Purine metabolism* | |
|  | | *Prephenate dehydrogenase* | | *Amino acid synthesis* | |
|  | | *Pyridoxal Kinase* | | *Cofactor Biosynthesis* | |
| 4 | | *Anthranilate synthase* | | *Amino acid transport and metabolism* | |
|  | | *3-demethylubiquinone-9 3-methyltransferase* | | *Cofactor Biosynthesis* | |
|  | | *Prephenate dehydrogenase* | | *Amino acid synthesis* | |
| 5 | | *Alpha-Glucosidase* | | *Complex Carbohydrates* | |
|  | | *Pyruvate-Formate Lyase* | | *Respiration* | |
|  | | *Cysteine synthase A* | | *Amino acid synthesis* | |
| 6 | | *Homoserine dehydrogenase* | | *Amino acid transport and metabolism* | |
|  | | *N-acetylmuramoyl-L-alanine amidase* | | *Glycan Biosynthesis and Metabolism* | |
|  | | *2-isopropylmalate synthase* | | *Amino acid synthesis* | |
| 7 | | *Carbamoyl phosphate synthetase small subunit glutamine amidotransferase* | | *Pyrimidine metabolism* | |
|  | | *Pyruvate-Formate Lyase* | | *Respiration* | |
|  | | *Argininosuccinate lyase* | | *Amino acid transport and metabolism* | |
| 8 | | *N-acetylmuramoyl-L-alanine amidase* | | *Glycan Biosynthesis and Metabolism* | |
|  | | *3-isopropylmalate dehydrogenase* | | *Amino acid synthesis* | |
|  | | *Argininosuccinate lyase* | | *Amino acid transport and metabolism* | |
| 9 | | *N-acetylmuramoyl-L-alanine amidase* | | *Glycan Biosynthesis and Metabolism* | |
|  | | *Ornithine carbamoyltransferase 1* | | *Amino acid transport and metabolism* | |
|  | | *Alpha-Glucosidase* | | *Complex Carbohydrates* | |
| 10 | | *Histidinol dehydrogenase* | | *Amino acid transport and metabolism* | |
|  | | *Anaerobic ribonucleoside-triphosphate reductase* | | *Pyrimidine metabolism* | |
|  | | *Diaminopimelate epimerase* | | *Amino acid synthesis* | |
| 11 | | *Alpha-Glucosidase* | | *Complex Carbohydrates* | |
|  | | *Diaminopimelate epimerase* | | *Amino acid synthesis* | |
|  | | *Formyltetrahydrofolate synthetase* | | *Organic Acids* | |
| 12 | | *Prephenate dehydrogenase* | | *Amino acid synthesis* | |
|  | | *Cysteine synthase A* | | *Amino acid synthesis* | |
|  | | *Butyrate Kinase* | | *Organic Acids* | |
| 13 | | *Beta-D-galactosidase* | | *Glycan structures - degradation;Complex Carbohydrates* | |
|  | | *Prephenate dehydrogenase* | | *Amino acid synthesis* | |
|  | | *Cystathionine gamma-synthase* | | *Amino acid synthesis* | |
| 14 | | *Alpha-Glucosidase* | | *Complex Carbohydrates* | |
|  | | *Anaerobic ribonucleoside-triphosphate reductase* | | *Pyrimidine metabolism* | |
|  | | *N-acetylmuramoyl-L-alanine amidase* | | *Glycan Biosynthesis and Metabolism* | |
| 15 | | *4-diphosphocytidyl-2C-methyl-D-erythritol synthase* | | *Isoprenoid biosynthesis* | |
|  | | *N-acetylmuramoyl-L-alanine amidase* | | *Glycan Biosynthesis and Metabolism* | |
|  | | *UDP-N-acetylglucosamine acyltransferase* | | *Glycan Biosynthesis and Metabolism* | |
| 16 | | *Diaminopimelate epimerase* | | *Amino acid synthesis* | |
|  | | *Beta-ketoacyl-acyl-carrier-protein synthase III* | | *Fatty Acid Biosynthesis* | |
|  | | *Agamintase* | | *Nitrogen Metabolism* | |
| 17 | | *Prephenate dehydrogenase* | | *Amino acid synthesis* | |
|  | | *L-threonine aldolase* | | *Amino acid synthesis* | |
|  | | *Thymidine phosphorylase* | | *Pyrimidine metabolism* | |
| 18 | | *Alanine racemase* | | *Amino acid synthesis* | |
|  | | *4-diphosphocytidyl-2C-methyl-D-erythritol synthase* | | *Isoprenoid biosynthesis* | |
|  | | *Diaminopimelate epimerase* | | *Amino acid synthesis* | |
| 19 | | *N-Acetylglucosamine-6-Phosphate Deacetylase* | | *Feeder Pathways to Glycolysis* | |
|  | | *Diaminopimelate epimerase* | | *Amino acid synthesis* | |
|  | | *Formyltetrahydrofolate synthetase* | | *Organic Acids* | |
| 20 | | *Galactokinase* | | *Feeder Pathways to Glycolysis* | |
|  | | *Diaminopimelate epimerase* | | *Amino acid synthesis* | |
|  | | *UDP-N-acetylmuramateL-alanine ligase* | | *Glycan Biosynthesis and Metabolism* | |
| 21 | | *Ribokinase* | | *Feeder Pathways to Glycolysis* | |
|  | | *Pyruvate-Formate Lyase* | | *Respiration* | |
|  | | *Transketolase* | | *Central Carbon Metabolism Pathways* | |
| 22 | | *Alanine racemase* | | *Amino acid synthesis* | |
|  | | *Pyruvate-Formate Lyase* | | *Respiration* | |
|  | | *Phosphoribosylglycinamide synthetase phosphoribosylamine-glycine ligase* | | *Purine metabolism* | |
| 23 | | *Glycerol Kinase* | | *Glycerolipid Metabolism* | |
|  | | *ATP phosphoribosyltransferase* | | *Amino acid transport and metabolism* | |
|  | | *Prephenate dehydrogenase* | | *Amino acid synthesis* | |
| 24 | | *Alpha-Glucosidase* | | *Complex Carbohydrates* | |
|  | | *L-alanine dehydrogenase* | | *Amino acid synthesis* | |
|  | | *Pyruvate-Formate Lyase* | | *Respiration* | |
| 25 | | *Pyruvate-Formate Lyase* | | *Respiration* | |
|  | | *UDP-N-acetylmuramoyl-L-alanyl-D-glutamatemeso-diaminopimelate ligase* | | *Glycan Biosynthesis and Metabolism* | |
|  | | *Selenocysteine synthase* | | *Amino acid synthesis;Amino acid synthesis* | |
| 26 | | *N-acetylmuramoyl-L-alanine amidase* | | *Glycan Biosynthesis and Metabolism* | |
|  | | *Pyridoxal Kinase* | | *Cofactor Biosynthesis* | |
|  | | *Acetyl-CoA acyltransferase anaerobic* | | *Fatty Acid Metabolism* | |
| 27 | | *Thymidine phosphorylase* | | *Pyrimidine metabolism* | |
|  | | *Diaminopimelate epimerase* | | *Amino acid synthesis* | |
|  | | *Arginosuccinate synthase* | | *Amino acid synthesis* | |
| 28 | | *Pyruvate-Formate Lyase* | | *Respiration* | |
|  | | *Formyltetrahydrofolate synthetase* | | *Organic Acids* | |
|  | | *Ornithine carbamoyltransferase 1* | | *Amino acid transport and metabolism* | |
| 29 | | *L-glutamine synthase* | | *Amino acid synthesis* | |
|  | | *Gamma-glutamyl kinase* | | *Amino acid transport and metabolism* | |
|  | | *Pyruvate-Formate Lyase* | | *Respiration* | |
| 30 | | *Butyrate Kinase* | | *Organic Acids* | |
|  | | *Diaminopimelate epimerase* | | *Amino acid synthesis* | |
|  | | *N-acetylmuramoyl-L-alanine amidase* | | *Glycan Biosynthesis and Metabolism* | |
| 31 | | *Dihydrodipicolinate reductase* | | *Amino acid transport and metabolism* | |
|  | | *Pyruvate-Formate Lyase* | | *Respiration* | |
|  | | *Beta-N-acetyl-D-hexosaminide N-acetylhexosaminohydrolase* | | *Glycosaminoglycan degradation* | |
| 32 | | *Transketolase* | | *Central Carbon Metabolism Pathways* | |
|  | | *Pyruvate-Formate Lyase* | | *Respiration* | |
|  | | *Alanine racemase* | | *Amino acid synthesis* | |
| 33 | | *N-acetylmuramoyl-L-alanine amidase* | | *Glycan Biosynthesis and Metabolism* | |
|  | | *N-acetylmuramoyl-L-alanine amidase* | | *Glycan Biosynthesis and Metabolism* | |
|  | | *Folylpolyglutamate Synthase* | | *Cofactor Biosynthesis* | |
| 34 | | *Transketolase* | | *Central Carbon Metabolism Pathways* | |
|  | | *Prephenate dehydrogenase* | | *Amino acid synthesis* | |
|  | | *Phosphoribosylglycinamide synthetase phosphoribosylamine-glycine ligase* | | *Purine metabolism* | |
| 35 | | *Diaminopimelate epimerase* | | *Amino acid synthesis* | |
|  | | *3-isopropylmalate dehydrogenase* | | *Amino acid synthesis* | |
|  | | *Thymidine phosphorylase* | | *Pyrimidine metabolism* | |
| 36 | | *Glycerol Kinase* | | *Glycerolipid Metabolism* | |
|  | | *Diaminopimelate epimerase* | | *Amino acid synthesis* | |
|  | | *Ribokinase* | | *Feeder Pathways to Glycolysis* | |
| 37 | | *Quinolinate Synthase* | | *Cofactor Biosynthesis* | |
|  | | *Pyruvate-Formate Lyase* | | *Respiration* | |
|  | | *Si-Citrate Synthase* | | *Central Carbon Metabolism Pathways* | |
| 38 | | *Beta-ketoacyl-acyl-carrier-protein synthase III* | | *Fatty Acid Biosynthesis* | |
|  | | *Transketolase* | | *Central Carbon Metabolism Pathways* | |
|  | | *Pyruvate-Formate Lyase* | | *Respiration* | |
| 39 | | *Pyruvate-Formate Lyase* | | *Respiration* | |
|  | | *Beta-ketoacyl-acyl-carrier-protein synthase III* | | *Fatty Acid Biosynthesis* | |
|  | | *Glutamate racemase* | | *Amino acid synthesis* | |
| 40 | | *Cytidylate kinase* | | *Pyrimidine metabolism* | |
|  | | *Diaminopimelate epimerase* | | *Amino acid synthesis* | |
|  | | *Pantothenate Synthetase* | | *Cofactor Biosynthesis* | |
| 41 | | *Uridine phosphorylase* | | *Pyrimidine metabolism* | |
|  | | *Beta-D-galactosidase* | | *Glycan structures - degradation;Complex Carbohydrates* | |
|  | | *Prephenate dehydrogenase* | | *Amino acid synthesis* | |
| 42 | | *Pyruvate-Formate Lyase* | | *Respiration* | |
|  | | *UDP-N-acetylmuramateL-alanine ligase* | | *Glycan Biosynthesis and Metabolism* | |
|  | | *Pyruvate-Formate Lyase* | | *Respiration* | |
| 43 | | *Carbon Monoxide Dehydrogenase* | | *Central Carbon Metabolism Pathways* | |
|  | | *Glutamate synthase large and small subunit (NADPH)* | | *Amino acid synthesis* | |
|  | | *Pyruvate-Formate Lyase* | | *Respiration* | |
| 44 | | *Diaminopimelate epimerase* | | *Amino acid synthesis* | |
|  | | *Si-Citrate Synthase* | | *Central Carbon Metabolism Pathways* | |
|  | | *3-isopropylmalate dehydrogenase* | | *Amino acid synthesis* | |
| 45 | | *Thioredoxin reductase FAD-NADP-binding* | | *Pyrimidine metabolism* | |
|  | | *N-acetyl-D-galactosamine-4-sulfate 4-sulfohydrolase* | | *Glycosaminoglycan degradation* | |
|  | | *Prephenate dehydrogenase* | | *Amino acid synthesis* | |
| 46 | | *Diaminopimelate epimerase* | | *Amino acid synthesis* | |
|  | | *Chorismate synthase* | | *Amino acid transport and metabolism* | |
|  | | *Anthranilate synthase* | | *Amino acid transport and metabolism* |  |
| 47 | | *Prephenate dehydrogenase* | | *Amino acid synthesis* |  |
|  | | *Thymidine phosphorylase* | | *Pyrimidine metabolism* |  |
|  | | *Anaerobic ribonucleoside-triphosphate reductase* | | *Pyrimidine metabolism* |  |
| 48 | | *L-alanine dehydrogenase* | | *Amino acid synthesis* |  |
|  | | *Prephenate dehydrogenase* | | *Amino acid synthesis* |  |
|  | | *3-dehydroquinate dehydratase* | | *Amino acid transport and metabolism* |  |
| 49 | | *Beta-ketoacyl-acyl-carrier-protein synthase III* | | *Fatty Acid Biosynthesis* |  |
|  | | *Methionyl-tRNA synthetase* | | *Amino acid synthesis* |  |
|  | | *Diaminopimelate epimerase* | | *Amino acid synthesis* |  |
| 50 | | *Glycerol Kinase* | | *Glycerolipid Metabolism* |  |
|  | | *Pyruvate-Formate Lyase* | | *Respiration* |  |
|  | | *Pyridoxal Kinase* | | *Cofactor Biosynthesis* |  |
| 51 | | *Prephenate dehydrogenase* | | *Amino acid synthesis* |  |
|  | | *Cytidylate kinase* | | *Pyrimidine metabolism* |  |
|  | | *Aspartate-ammonia ligase* | | *Amino acid synthesis* |  |
| 52 | | *Beta-N-acetyl-D-hexosaminide N-acetylhexosaminohydrolase* | | *Glycosaminoglycan degradation* |  |
|  | | *Prephenate dehydrogenase* | | *Amino acid synthesis* |  |
|  | | *Pyruvate-Formate Lyase* | | *Respiration* |  |
| 53 | | *N-acetylmuramoyl-L-alanine amidase* | | *Glycan Biosynthesis and Metabolism* |  |
|  | | *1-Phosphofructokinase* | | *Feeder Pathways to Glycolysis* |  |
|  | | *Cysteine synthase A* | | *Amino acid synthesis* |  |
| 54 | | *3-demethylubiquinone-9 3-methyltransferase* | | *Cofactor Biosynthesis* |  |
|  | | *Prephenate dehydrogenase* | | *Amino acid synthesis* |  |
|  | | *Cysteine synthase A* | | *Amino acid synthesis* |  |
| 55 | | *Ribokinase* | | *Feeder Pathways to Glycolysis* |  |
|  | | *Diaminopimelate epimerase* | | *Amino acid synthesis* |  |
|  | | *Glycerol Kinase* | | *Glycerolipid Metabolism* |  |
| 56 | | *D-alanineD-alanine ligase* | | *Glycan Biosynthesis and Metabolism* |  |
|  | | *Diaminopimelate epimerase* | | *Amino acid synthesis* |  |
|  | | *Chorismate synthase* | | *Amino acid transport and metabolism* |  |
| 57 | | *Prephenate dehydrogenase* | | *Amino acid synthesis* |  |
|  | | *Beta-ketoacyl-acyl-carrier-protein synthase III* | | *Fatty Acid Biosynthesis* |  |
|  | | *Biotin Synthase* | | *Cofactor Biosynthesis* |  |
| 58 | | *Anthranilate synthase* | | *Amino acid transport and metabolism* |  |
|  | | *Prephenate dehydrogenase* | | *Amino acid synthesis* |  |
|  | | *Butyryl CoA Acetate CoA Transferase* | | *Organic Acids* |  |
| 59 | | *Prephenate dehydrogenase* | | *Amino acid synthesis* |  |
|  | | *3-dehydroquinate dehydratase* | | *Amino acid transport and metabolism* |  |
|  | | *N-Acetylglucosamine-6-Phosphate Deacetylase* | | *Feeder Pathways to Glycolysis* |  |
| 60 | | *Uridine phosphorylase* | | *Pyrimidine metabolism* |  |
|  | | *N-acetylmuramoyl-L-alanine amidase* | | *Glycan Biosynthesis and Metabolism* |  |
|  | | *N-acetyl-D-glucosamine-6-sulfate 6-sulfohydrolase* | | *Glycosaminoglycan degradation* |  |
| 61 | | *Cysteine synthase A* | | *Amino acid synthesis* |  |
|  | | *N-acetylmuramoyl-L-alanine amidase* | | *Glycan Biosynthesis and Metabolism* |  |
|  | | *Purine-nucleoside phosphorylase* | | *Purine metabolism* |  |
| 62 | | *Prephenate dehydrogenase* | | *Amino acid synthesis* |  |
|  | | *Phosphoribosylglycinamide synthetase phosphoribosylamine-glycine ligase* | | *Purine metabolism* |  |
|  | | *Carbamoyl phosphate synthetase small subunit glutamine amidotransferase* | | *Pyrimidine metabolism* |  |
| 63 | | *Pyridoxal Kinase* | | *Cofactor Biosynthesis* |  |
|  | | *N-acetylmuramoyl-L-alanine amidase* | | *Glycan Biosynthesis and Metabolism* |  |
|  | | *Dihydrodipicolinate synthase* | | *Amino acid synthesis* |  |
| 64 | | *Pyruvate-Formate Lyase* | | *Respiration* |  |
|  | | *Folylpolyglutamate Synthase* | | *Cofactor Biosynthesis* |  |
|  | | *Fucose Isomerase* | | *Feeder Pathways to Glycolysis* |  |
| 65 | | *Glucuronate Isomerase* | | *Feeder Pathways to Glycolysis* |  |
|  | | *Prephenate dehydrogenase* | | *Amino acid synthesis* |  |
|  | | *D-alanineD-alanine ligase* | | *Glycan Biosynthesis and Metabolism* |  |
| 66 | | *D-alanineD-alanine ligase* | | *Glycan Biosynthesis and Metabolism* |  |
|  | | *N-acetylmuramoyl-L-alanine amidase* | | *Glycan Biosynthesis and Metabolism* |  |
|  | | *Glycerol Kinase* | | *Glycerolipid Metabolism* |  |
| 67 | | *Diaminopimelate epimerase* | | *Amino acid synthesis* |  |
|  | | *Dihydrodipicolinate synthase* | | *Amino acid synthesis* |  |
|  | | *Adenylosuccinate synthetase* | | *Purine metabolism* |  |
| 68 | | *IMP dehydrogenase* | | *Purine metabolism* |  |
|  | | *Diaminopimelate epimerase* | | *Amino acid synthesis* |  |
|  | | *Dihydrodipicolinate synthase* | | *Amino acid synthesis* |  |
| 69 | | *2-isopropylmalate synthase* | | *Amino acid synthesis* |  |
|  | | *N-acetylmuramoyl-L-alanine amidase* | | *Glycan Biosynthesis and Metabolism* |  |
|  | | *Folylpolyglutamate Synthase* | | *Cofactor Biosynthesis* |  |
| 70 | | *N-acetylmuramoyl-L-alanine amidase* | | *Glycan Biosynthesis and Metabolism* |  |
|  | | *Pyruvate-Formate Lyase* | | *Respiration* |  |
|  | | *Alanine racemase* | | *Amino acid synthesis* |  |
| 71 | | *Alanine racemase* | | *Amino acid synthesis* |  |
|  | | *Prephenate dehydrogenase* | | *Amino acid synthesis* |  |
|  | | *1-hydroxy-2-methyl-2-E-butenyl 4-diphosphate reductase 4Fe-4S protein* | | *Isoprenoid biosynthesis* |  |
| 72 | | *3-demethylubiquinone-9 3-methyltransferase* | | *Cofactor Biosynthesis* |  |
|  | | *N-acetyl-D-glucosamine-6-sulfate 6-sulfohydrolase* | | *Glycosaminoglycan degradation* |  |
|  | | *N-acetylmuramoyl-L-alanine amidase* | | *Glycan Biosynthesis and Metabolism* |  |
| 73 | | *Prephenate dehydrogenase* | | *Amino acid synthesis* |  |
|  | | *Cysteine synthase A* | | *Amino acid synthesis* |  |
|  | | *Threonine ammonia-lyase* | | *Amino acid synthesis* |  |
| 74 | | *Prephenate dehydrogenase* | | *Amino acid synthesis* |  |
|  | | *Alpha-Glucosidase* | | *Complex Carbohydrates* |  |
|  | | *Ornithine carbamoyltransferase 1* | | *Amino acid transport and metabolism* |  |
| 75 | | *UDP-N-acetylmuramoyl-L-alanineD-glutamate ligase* | | *Glycan Biosynthesis and Metabolism* |  |
|  | | *Transketolase* | | *Central Carbon Metabolism Pathways* |  |
|  | | *Diaminopimelate epimerase* | | *Amino acid synthesis* |  |
| 76 | | *Pyruvate-Formate Lyase* | | *Respiration* |  |
|  | | *4-diphosphocytidyl-2C-methyl-D-erythritol synthase* | | *Isoprenoid biosynthesis* |  |
|  | | *Alpha-mannosidase* | | *N-Glycan degradation* |  |
| 77 | | *Purine-nucleoside phosphorylase* | | *Purine metabolism* |  |
|  | | *Alpha-Glucosidase* | | *Complex Carbohydrates* |  |
|  | | *Prephenate dehydrogenase* | | *Amino acid synthesis* |  |
| 78 | | *Transketolase* | | *Central Carbon Metabolism Pathways* |  |
|  | | *Prephenate dehydrogenase* | | *Amino acid synthesis* |  |
|  | | *Thioredoxin reductase FAD-NADP-binding* | | *Pyrimidine metabolism* |  |
| 79 | | *Pyruvate-Formate Lyase* | | *Respiration* |  |
|  | | *Formyltetrahydrofolate synthetase* | | *Organic Acids* |  |
|  | | *Pyridoxal Kinase* | | *Cofactor Biosynthesis* |  |
| 80 | | *Aspartate kinase* | | *Amino acid synthesis* |  |
|  | | *Diaminopimelate epimerase* | | *Amino acid synthesis* |  |
|  | | *UDP-N-acetylmuramateL-alanine ligase* | | *Glycan Biosynthesis and Metabolism* |  |
| 81 | | *Carbamoyl phosphate synthetase small subunit glutamine amidotransferase* | | *Pyrimidine metabolism* |  |
|  | | *Shikimate kinase I II* | | *Amino acid transport and metabolism* |  |
|  | | *Pyruvate-Formate Lyase* | | *Respiration* |  |
| 82 | | *Pyruvate-Formate Lyase* | | *Respiration* |  |
|  | | *Cytosine deaminase* | | *Pyrimidine metabolism* |  |
|  | | *Glutamate synthase large and small subunit (NADPH)* | | *Amino acid synthesis* |  |
| 83 | | *N-acetylmuramoyl-L-alanine amidase* | | *Glycan Biosynthesis and Metabolism* |  |
|  | | *Threonine ammonia-lyase* | | *Amino acid synthesis* |  |
|  | | *Beta-N-acetyl-D-hexosaminide N-acetylhexosaminohydrolase* | | *Glycosaminoglycan degradation* |  |
| 84 | | *Diaminopimelate epimerase* | | *Amino acid synthesis* |  |
|  | | *Pantothenate Synthetase* | | *Cofactor Biosynthesis* |  |
|  | | *Formyltetrahydrofolate synthetase* | | *Organic Acids* |  |
| 85 | | *Alpha-Glucosidase* | | *Complex Carbohydrates* |  |
|  | | *Diaminopimelate epimerase* | | *Amino acid synthesis* |  |
|  | | *Asparaginase* | | *Amino acid synthesis* |  |
| 86 | | *UDP-N-acetylmuramateL-alanine ligase* | | *Glycan Biosynthesis and Metabolism* |  |
|  | | *Pyruvate-Formate Lyase* | | *Respiration* |  |
|  | | *Alpha-Glucosidase* | | *Complex Carbohydrates* |  |
| 87 | | *Formyltetrahydrofolate synthetase* | | *Organic Acids* |  |
|  | | *Prephenate dehydrogenase* | | *Amino acid synthesis* |  |
|  | | *Aspartate kinase* | | *Amino acid synthesis* |  |
| 88 | | *Formyltetrahydrofolate synthetase* | | *Organic Acids* |  |
|  | | *Diaminopimelate epimerase* | | *Amino acid synthesis* |  |
|  | | *Glutamate racemase* | | *Amino acid synthesis* |  |
| 89 | | *Galactokinase* | | *Feeder Pathways to Glycolysis* |  |
|  | | *Diaminopimelate epimerase* | | *Amino acid synthesis* |  |
|  | | *Butyrate Kinase* | | *Organic Acids* |  |
| 90 | | *Shikimate kinase I II* | | *Amino acid transport and metabolism* |  |
|  | | *Conjugated Bile Salt Hydrolase* | | *Exotic Metabolisms* |  |
|  | | *Pyruvate-Formate Lyase* | | *Respiration* |  |
| 91 | | *UDP-N-acetylmuramoyl-L-alanyl-D-glutamatemeso-diaminopimelate ligase* | | *Glycan Biosynthesis and Metabolism* |  |
|  | | *Diaminopimelate epimerase* | | *Amino acid synthesis* |  |
|  | | *Beta-ketoacyl-acyl-carrier-protein synthase III* | | *Fatty Acid Biosynthesis* |  |
| 92 | | *Diaminopimelate epimerase* | | *Amino acid synthesis* |  |
|  | | *Ornithine carbamoyltransferase 1* | | *Amino acid transport and metabolism* |  |
|  | | *Beta-D-galactosidase* | | *Glycan structures - degradation;Complex Carbohydrates* |  |
| 93 | | *3-isopropylmalate dehydrogenase* | | *Amino acid synthesis* |  |
|  | | *N-Acetylglucosamine-6-Phosphate Deacetylase* | | *Feeder Pathways to Glycolysis* |  |
|  | | *Diaminopimelate epimerase* | | *Amino acid synthesis* |  |
| 94 | | *Prephenate dehydrogenase* | | *Amino acid synthesis* |  |
|  | | *IMP dehydrogenase* | | *Purine metabolism* |  |
|  | | *Conjugated Bile Salt Hydrolase* | | *Exotic Metabolisms* |  |
| 95 | | *Glutamate synthase large and small subunit (NADPH)* | | *Amino acid synthesis* |  |
|  | | *Anthranilate synthase* | | *Amino acid transport and metabolism* |  |
|  | | *Pyruvate-Formate Lyase* | | *Respiration* |  |
| 96 | | *Pyruvate-Formate Lyase* | | *Respiration* |  |
|  | | *Anaerobic ribonucleoside-triphosphate reductase* | | *Pyrimidine metabolism* |  |
|  | | *Acetyl-CoA acyltransferase anaerobic* | | *Fatty Acid Metabolism* |  |
| 97 | | *Xylose Isomerase* | | *Feeder Pathways to Glycolysis* |  |
|  | | *Cytidylate kinase* | | *Pyrimidine metabolism* |  |
|  | | *Pyruvate-Formate Lyase* | | *Respiration* |  |
| 98 | | *Butyrate Kinase* | | *Organic Acids* |  |
|  | | *N-acetylmuramoyl-L-alanine amidase* | | *Glycan Biosynthesis and Metabolism* |  |
|  | | *UDP-N-acetylmuramateL-alanine ligase* | | *Glycan Biosynthesis and Metabolism* |  |
| 99 | | *N-acetylmuramoyl-L-alanine amidase* | | *Glycan Biosynthesis and Metabolism* |  |
|  | | *Beta-ketoacyl-acyl-carrier-protein synthase III* | | *Fatty Acid Biosynthesis* |  |
|  | | *L-threonine aldolase* | | *Amino acid synthesis* |  |
| 100 | | *Carbamoyl phosphate synthetase small subunit glutamine amidotransferase* | | *Pyrimidine metabolism* |  |
|  | | *N-acetylmuramoyl-L-alanine amidase* | | *Glycan Biosynthesis and Metabolism* |  |
|  | | *Butyrate Kinase* | | *Organic Acids* |  |
| 101 | | *L-glutamine synthase* | | *Amino acid synthesis* |  |
|  | | *ADP-L-glycero-D-mannoheptose-6-epimerase NAD(P)-binding* | | *Glycan Biosynthesis and Metabolism* |  |
|  | | *Pyruvate-Formate Lyase* | | *Respiration* |  |
| 102 | | *Diaminopimelate epimerase* | | *Amino acid synthesis* |  |
|  | | *2-isopropylmalate synthase* | | *Amino acid synthesis* |  |
|  | | *Selenocysteine synthase* | | *Amino acid synthesis;Amino acid synthesis* |  |
| 103 | | *Diaminopimelate epimerase* | | *Amino acid synthesis* |  |
|  | | *Cytosine deaminase* | | *Pyrimidine metabolism* |  |
|  | | *UDP-N-acetylmuramateL-alanine ligase* | | *Glycan Biosynthesis and Metabolism* |  |
| 104 | | *Acetyl-CoA acyltransferase anaerobic* | | *Fatty Acid Metabolism* |  |
|  | | *Beta-ketoacyl-acyl-carrier-protein synthase III* | | *Fatty Acid Biosynthesis* |  |
|  | | *Prephenate dehydrogenase* | | *Amino acid synthesis* |  |
| 105 | | *Beta-D-galactosidase* | | *Glycan structures - degradation;Complex Carbohydrates* |  |
|  | | *N-acetylmuramoyl-L-alanine amidase* | | *Glycan Biosynthesis and Metabolism* |  |
|  | | *Alpha-Glucosidase* | | *Complex Carbohydrates* |  |
| 106 | | *N-acetylmuramoyl-L-alanine amidase* | | *Glycan Biosynthesis and Metabolism* |  |
|  | | *Asparaginase* | | *Amino acid synthesis* |  |
|  | | *3-isopropylmalate dehydrogenase* | | *Amino acid synthesis* |  |
| 107 | | *L-glutamine synthase* | | *Amino acid synthesis* |  |
|  | | *Diaminopimelate epimerase* | | *Amino acid synthesis* |  |
|  | | *Cytosine deaminase* | | *Pyrimidine metabolism* |  |
| 108 | | *Argininosuccinate lyase* | | *Amino acid transport and metabolism* |  |
|  | | *Acetate Kinase* | | *Organic Acids* |  |
|  | | *Pyruvate-Formate Lyase* | | *Respiration* |  |
| 109 | | *Pyruvate-Formate Lyase* | | *Respiration* |  |
|  | | *Beta-ketoacyl-acyl-carrier-protein synthase III* | | *Fatty Acid Biosynthesis* |  |
|  | | *Shikimate kinase I II* | | *Amino acid transport and metabolism* |  |
| 110 | | *Diaminopimelate epimerase* | | *Amino acid synthesis* |  |
|  | | *L-glutamine synthase* | | *Amino acid synthesis* |  |
|  | | *N-acetylmuramoyl-L-alanine amidase* | | *Glycan Biosynthesis and Metabolism* |  |
| 111 | | *Carbon Monoxide Dehydrogenase* | | *Central Carbon Metabolism Pathways* |  |
|  | | *N-acetylmuramoyl-L-alanine amidase* | | *Glycan Biosynthesis and Metabolism* |  |
|  | | *Asparaginase* | | *Amino acid synthesis* |  |
| 112 | | *3-isopropylmalate dehydrogenase* | | *Amino acid synthesis* |  |
|  | | *Alanine racemase* | | *Amino acid synthesis* |  |
|  | | *Diaminopimelate epimerase* | | *Amino acid synthesis* |  |
| 113 | | *Pyruvate-Formate Lyase* | | *Respiration* |  |
|  | | *Glutamate synthase large and small subunit (NADPH)* | | *Amino acid synthesis* |  |
|  | | *Uridine phosphorylase* | | *Pyrimidine metabolism* |  |
| 114 | | *Folylpolyglutamate Synthase* | | *Cofactor Biosynthesis* |  |
|  | | *Diaminopimelate epimerase* | | *Amino acid synthesis* |  |
|  | | *Beta-N-acetyl-D-hexosaminide N-acetylhexosaminohydrolase* | | *Glycosaminoglycan degradation* |  |
| 115 | | *Biosynthetic arginine decarboxylase PLP-binding* | | *Amino acid transport and metabolism* |  |
|  | | *Pyridoxal Kinase* | | *Cofactor Biosynthesis* |  |
|  | | *N-acetylmuramoyl-L-alanine amidase* | | *Glycan Biosynthesis and Metabolism* |  |
| 116 | | *Fucose Isomerase* | | *Feeder Pathways to Glycolysis* |  |
|  | | *Beta-D-galactosidase* | | *Glycan structures - degradation;Complex Carbohydrates* |  |
|  | | *Diaminopimelate epimerase* | | *Amino acid synthesis* |  |
| 117 | | *Cystathionine gamma-synthase* | | *Amino acid synthesis* |  |
|  | | *Orotidine-5-phosphate decarboxylase* | | *Pyrimidine metabolism* |  |
|  | | *Diaminopimelate epimerase* | | *Amino acid synthesis* |  |
| 118 | | *Prephenate dehydrogenase* | | *Amino acid synthesis* |  |
|  | | *Geranyltranstransferase* | | *Isoprenoid biosynthesis* |  |
|  | | *Pyridoxal Kinase* | | *Cofactor Biosynthesis* |  |
| 119 | | *Ribokinase* | | *Feeder Pathways to Glycolysis* |  |
|  | | *Pyruvate-Formate Lyase* | | *Respiration* |  |
|  | | *Xylose Isomerase* | | *Feeder Pathways to Glycolysis* |  |
| 120 | | *UDP-N-acetylmuramoyl-L-alanineD-glutamate ligase* | | *Glycan Biosynthesis and Metabolism* |  |
|  | | *Threonine ammonia-lyase* | | *Amino acid synthesis* |  |
|  | | *Pyruvate-Formate Lyase* | | *Respiration* |  |
| 121 | | *Cysteine synthase A* | | *Amino acid synthesis* |  |
|  | | *Alpha-Glucosidase* | | *Complex Carbohydrates* |  |
|  | | *Prephenate dehydrogenase* | | *Amino acid synthesis* |  |
| 122 | | *Glutamate racemase* | | *Amino acid synthesis* |  |
|  | | *Transketolase* | | *Central Carbon Metabolism Pathways* |  |
|  | | *Prephenate dehydrogenase* | | *Amino acid synthesis* |  |
| 123 | | *Dihydrodipicolinate reductase* | | *Amino acid transport and metabolism* |  |
|  | | *Prephenate dehydrogenase* | | *Amino acid synthesis* |  |
|  | | *Riboflavin Synthase α Subunit* | | *Cofactor Biosynthesis* |  |
| 124 | | *Phosphoribosylglycinamide synthetase phosphoribosylamine-glycine ligase* | | *Purine metabolism* |  |
|  | | *2-isopropylmalate synthase* | | *Amino acid synthesis* |  |
|  | | *N-acetylmuramoyl-L-alanine amidase* | | *Glycan Biosynthesis and Metabolism* |  |
| 125 | | *Pyruvate-Formate Lyase* | | *Respiration* |  |
|  | | *Threonine ammonia-lyase* | | *Amino acid synthesis* |  |
|  | | *Xylose Isomerase* | | *Feeder Pathways to Glycolysis* |  |
| 126 | | *Transaldolase* | | *Central Carbon Metabolism Pathways* |  |
|  | | *Prephenate dehydrogenase* | | *Amino acid synthesis* |  |
|  | | *Adenylosuccinate synthetase* | | *Purine metabolism* |  |
| 127 | | *3-isopropylmalate dehydrogenase* | | *Amino acid synthesis* |  |
|  | | *Diaminopimelate epimerase* | | *Amino acid synthesis* |  |
|  | | *UDP-N-acetylmuramoyl-L-alanineD-glutamate ligase* | | *Glycan Biosynthesis and Metabolism* |  |
| 128 | | *Thioredoxin reductase FAD-NADP-binding* | | *Pyrimidine metabolism* |  |
|  | | *Prephenate dehydrogenase* | | *Amino acid synthesis* |  |
|  | | *L-Lactate Dehydrogenase* | | *Organic Acids* |  |
| 129 | | *N-acetylmuramoyl-L-alanine amidase* | | *Glycan Biosynthesis and Metabolism* |  |
|  | | *3-demethylubiquinone-9 3-methyltransferase* | | *Cofactor Biosynthesis* |  |
|  | | *Ribokinase* | | *Feeder Pathways to Glycolysis* |  |
| 130 | | *Lysine decarboxylase 1* | | *Amino acid transport and metabolism* |  |
|  | | *Histidinol dehydrogenase* | | *Amino acid transport and metabolism* |  |
|  | | *Prephenate dehydrogenase* | | *Amino acid synthesis* |  |
| 131 | | *Cytidylate kinase* | | *Pyrimidine metabolism* |  |
|  | | *Pyruvate-Formate Lyase* | | *Respiration* |  |
|  | | *Diaminopimelate epimerase* | | *Amino acid synthesis* |  |
| 132 | | *Aspartate-ammonia ligase* | | *Amino acid synthesis* |  |
|  | | *L-threonine aldolase* | | *Amino acid synthesis* |  |
|  | | *Pyruvate-Formate Lyase* | | *Respiration* |  |
| 133 | | *Diaminopimelate epimerase* | | *Amino acid synthesis* |  |
|  | | *3-isopropylmalate dehydrogenase* | | *Amino acid synthesis* |  |
|  | | *N-acetylglutamate synthase* | | *Amino acid transport and metabolism* |  |
| 134 | | *Diaminopimelate epimerase* | | *Amino acid synthesis* |  |
|  | | *Pyridoxal Kinase* | | *Cofactor Biosynthesis* |  |
|  | | *Butyrate Kinase* | | *Organic Acids* |  |
| 135 | | *Alpha-mannosidase* | | *N-Glycan degradation* |  |
|  | | *Acetyl-CoA acyltransferase anaerobic* | | *Fatty Acid Metabolism* |  |
|  | | *Prephenate dehydrogenase* | | *Amino acid synthesis* |  |
| 136 | | *UDP-N-acetylmuramateL-alanine ligase* | | *Glycan Biosynthesis and Metabolism* |  |
|  | | *Prephenate dehydrogenase* | | *Amino acid synthesis* |  |
|  | | *Beta-D-glucuronidase* | | *Glycan structures - degradation;Exotic Metabolisms* |  |
| 137 | | *Prephenate dehydrogenase* | | *Amino acid synthesis* |  |
|  | | *Cysteine synthase A* | | *Amino acid synthesis* |  |
|  | | *UDP-N-acetylmuramoyl-L-alanineD-glutamate ligase* | | *Glycan Biosynthesis and Metabolism* |  |
| 138 | | *Pyruvate-Formate Lyase* | | *Respiration* |  |
|  | | *Beta-D-glucuronidase* | | *Glycan structures - degradation;Exotic Metabolisms* |  |
|  | | *3-dehydroquinate dehydratase* | | *Amino acid transport and metabolism* |  |
| 139 | | *Diaminopimelate epimerase* | | *Amino acid synthesis* |  |
|  | | *Shikimate kinase I II* | | *Amino acid transport and metabolism* |  |
|  | | *Alpha-Glucosidase* | | *Complex Carbohydrates* |  |
| 140 | | *Butyrate Kinase* | | *Organic Acids* |  |
|  | | *Phosphoribosylglycinamide synthetase phosphoribosylamine-glycine ligase* | | *Purine metabolism* |  |
|  | | *N-acetylmuramoyl-L-alanine amidase* | | *Glycan Biosynthesis and Metabolism* |  |
| 141 | | *Anthranilate synthase* | | *Amino acid transport and metabolism* |  |
|  | | *Folylpolyglutamate Synthase* | | *Cofactor Biosynthesis* |  |
|  | | *Pyruvate-Formate Lyase* | | *Respiration* |  |
| 142 | | *Anthranilate synthase* | | *Amino acid transport and metabolism* |  |
|  | | *Chorismate synthase* | | *Amino acid transport and metabolism* |  |
|  | | *Pyruvate-Formate Lyase* | | *Respiration* |  |
| 143 | | *N-acetyl-D-galactosamine-4-sulfate 4-sulfohydrolase* | | *Glycosaminoglycan degradation* |  |
|  | | *Glycerol Kinase* | | *Glycerolipid Metabolism* |  |
|  | | *Diaminopimelate epimerase* | | *Amino acid synthesis* |  |
| 144 | | *4-diphosphocytidyl-2C-methyl-D-erythritol synthase* | | *Isoprenoid biosynthesis* |  |
|  | | *ATP phosphoribosyltransferase* | | *Amino acid transport and metabolism* |  |
|  | | *Diaminopimelate epimerase* | | *Amino acid synthesis* |  |
| 145 | | *L-glutamine synthase* | | *Amino acid synthesis* |  |
|  | | *L-threonine aldolase* | | *Amino acid synthesis* |  |
|  | | *Prephenate dehydrogenase* | | *Amino acid synthesis* |  |
| 146 | | *Beta-D-glucuronidase* | | *Glycan structures - degradation;Exotic Metabolisms* |  |
|  | | *Folylpolyglutamate Synthase* | | *Cofactor Biosynthesis* |  |
|  | | *Diaminopimelate epimerase* | | *Amino acid synthesis* |  |
| 147 | | *Beta-D-galactosidase* | | *Glycan structures - degradation;Complex Carbohydrates* |  |
|  | | *N-acetylmuramoyl-L-alanine amidase* | | *Glycan Biosynthesis and Metabolism* |  |
|  | | *Glycerol Kinase* | | *Glycerolipid Metabolism* |  |
| 148 | | *Threonine ammonia-lyase* | | *Amino acid synthesis* |  |
|  | | *Dihydrodipicolinate synthase* | | *Amino acid synthesis* |  |
|  | | *Diaminopimelate epimerase* | | *Amino acid synthesis* |  |
| 149 | | *Pyruvate-Formate Lyase* | | *Respiration* |  |
|  | | *N-acetylmuramoyl-L-alanine amidase* | | *Glycan Biosynthesis and Metabolism* |  |
|  | | *Beta-D-glucuronidase* | | *Glycan structures - degradation;Exotic Metabolisms* |  |
| 150 | | *Indole-3-glycerol-phosphate synthase* | | *Amino acid synthesis* |  |
|  | | *Ornithine carbamoyltransferase 1* | | *Amino acid transport and metabolism* |  |
|  | | *N-acetylmuramoyl-L-alanine amidase* | | *Glycan Biosynthesis and Metabolism* |  |
| 151 | | *Ribokinase* | | *Feeder Pathways to Glycolysis* |  |
|  | | *Ribokinase* | | *Feeder Pathways to Glycolysis* |  |
|  | | *N-acetylmuramoyl-L-alanine amidase* | | *Glycan Biosynthesis and Metabolism* |  |
| 152 | | *Acetyl-CoA acyltransferase anaerobic* | | *Fatty Acid Metabolism* |  |
|  | | *UDP-N-acetylmuramateL-alanine ligase* | | *Glycan Biosynthesis and Metabolism* |  |
|  | | *Diaminopimelate epimerase* | | *Amino acid synthesis* |  |
| 153 | | *Glutamate synthase large and small subunit (NADPH)* | | *Amino acid synthesis* |  |
|  | | *Butyrate Kinase* | | *Organic Acids* |  |
|  | | *Pyruvate-Formate Lyase* | | *Respiration* |  |
| 154 | | *1-hydroxy-2-methyl-2-E-butenyl 4-diphosphate reductase 4Fe-4S protein* | | *Isoprenoid biosynthesis* |  |
|  | | *Prephenate dehydrogenase* | | *Amino acid synthesis* |  |
|  | | *D-alanineD-alanine ligase* | | *Glycan Biosynthesis and Metabolism* |  |
| 155 | | *Anthranilate synthase* | | *Amino acid transport and metabolism* |  |
|  | | *Diaminopimelate epimerase* | | *Amino acid synthesis* |  |
|  | | *Alanine racemase* | | *Amino acid synthesis* |  |
| 156 | | *Folylpolyglutamate Synthase* | | *Cofactor Biosynthesis* |  |
|  | | *N-acetylmuramoyl-L-alanine amidase* | | *Glycan Biosynthesis and Metabolism* |  |
|  | | *Anaerobic ribonucleoside-triphosphate reductase* | | *Pyrimidine metabolism* |  |
| 157 | | *Argininosuccinate lyase* | | *Amino acid transport and metabolism* |  |
|  | | *Threonine ammonia-lyase* | | *Amino acid synthesis* |  |
|  | | *Prephenate dehydrogenase* | | *Amino acid synthesis* |  |
| 158 | | *Prephenate dehydrogenase* | | *Amino acid synthesis* |  |
|  | | *Alanine racemase* | | *Amino acid synthesis* |  |
|  | | *Folylpolyglutamate Synthase* | | *Cofactor Biosynthesis* |  |
| 159 | | *Beta-N-acetyl-D-hexosaminide N-acetylhexosaminohydrolase* | | *Glycosaminoglycan degradation* |  |
|  | | *N-Acetylglucosamine-6-Phosphate Deacetylase* | | *Feeder Pathways to Glycolysis* |  |
|  | | *N-acetylmuramoyl-L-alanine amidase* | | *Glycan Biosynthesis and Metabolism* |  |
| 160 | | *Adenylosuccinate synthetase* | | *Purine metabolism* |  |
|  | | *L-alanine dehydrogenase* | | *Amino acid synthesis* |  |
|  | | *Pyruvate-Formate Lyase* | | *Respiration* |  |
| 161 | | *Geranyltranstransferase* | | *Isoprenoid biosynthesis* |  |
|  | | *Cytosine deaminase* | | *Pyrimidine metabolism* |  |
|  | | *Diaminopimelate epimerase* | | *Amino acid synthesis* |  |
| 162 | | *UDP-N-acetylmuramoyl-L-alanyl-D-glutamatemeso-diaminopimelate ligase* | | *Glycan Biosynthesis and Metabolism* |  |
|  | | *Prephenate dehydrogenase* | | *Amino acid synthesis* |  |
|  | | *Mannose-6-Phosphate Isomerase* | | *Feeder Pathways to Glycolysis* |  |
| 163 | | *Prephenate dehydrogenase* | | *Amino acid synthesis* |  |
|  | | *Shikimate kinase I II* | | *Amino acid transport and metabolism* |  |
|  | | *Cysteine synthase A* | | *Amino acid synthesis* |  |
| 164 | | *Histidinol dehydrogenase* | | *Amino acid transport and metabolism* |  |
|  | | *Uridine phosphorylase* | | *Pyrimidine metabolism* |  |
|  | | *Diaminopimelate epimerase* | | *Amino acid synthesis* |  |
| 165 | | *N-acetylmuramoyl-L-alanine amidase* | | *Glycan Biosynthesis and Metabolism* |  |
|  | | *Pyruvate-Formate Lyase* | | *Respiration* |  |
|  | | *Ornithine carbamoyltransferase 1* | | *Amino acid transport and metabolism* |  |
| 166 | | *Diaminopimelate epimerase* | | *Amino acid synthesis* |  |
|  | | *3-isopropylmalate dehydrogenase* | | *Amino acid synthesis* |  |
|  | | *UDP-N-acetylmuramateL-alanine ligase* | | *Glycan Biosynthesis and Metabolism* |  |
| 167 | | *Aspartate-ammonia ligase* | | *Amino acid synthesis* |  |
|  | | *Thioredoxin reductase FAD-NADP-binding* | | *Pyrimidine metabolism* |  |
|  | | *Pyruvate-Formate Lyase* | | *Respiration* |  |
| 168 | | *Pyruvate-Formate Lyase* | | *Respiration* |  |
|  | | *Uridine phosphorylase* | | *Pyrimidine metabolism* |  |
|  | | *Beta-D-glucuronidase* | | *Glycan structures - degradation;Exotic Metabolisms* |  |
| 169 | | *Dihydrodipicolinate synthase* | | *Amino acid synthesis* |  |
|  | | *Prephenate dehydrogenase* | | *Amino acid synthesis* |  |
|  | | *Homoserine dehydrogenase* | | *Amino acid transport and metabolism* |  |
| 170 | | *Pyruvate-Formate Lyase* | | *Respiration* |  |
|  | | *Glutamate synthase large and small subunit (NADPH)* | | *Amino acid synthesis* |  |
|  | | *Beta-D-glucuronidase* | | *Glycan structures - degradation;Exotic Metabolisms* |  |
| 171 | | *Thymidine phosphorylase* | | *Pyrimidine metabolism* |  |
|  | | *Pyruvate-Formate Lyase* | | *Respiration* |  |
|  | | *N-acetylglutamate synthase* | | *Amino acid transport and metabolism* |  |
| 172 | | *Indole-3-glycerol-phosphate synthase* | | *Amino acid synthesis* |  |
|  | | *N-Acetylglucosamine-6-Phosphate Deacetylase* | | *Feeder Pathways to Glycolysis* |  |
|  | | *Diaminopimelate epimerase* | | *Amino acid synthesis* |  |
| 173 | | *Prephenate dehydrogenase* | | *Amino acid synthesis* |  |
|  | | *Purine-nucleoside phosphorylase* | | *Purine metabolism* |  |
|  | | *Pyruvate-Formate Lyase* | | *Respiration* |  |
| 174 | | *Pyruvate-Formate Lyase* | | *Respiration* |  |
|  | | *IMP dehydrogenase* | | *Purine metabolism* |  |
|  | | *ADP-ribose pyrophosphatase* | | *Purine metabolism* |  |
| 175 | | *Transketolase* | | *Central Carbon Metabolism Pathways* |  |
|  | | *Prephenate dehydrogenase* | | *Amino acid synthesis* |  |
|  | | *Cysteine synthase A* | | *Amino acid synthesis* |  |
| 176 | | *Beta-D-galactosidase* | | *Glycan structures - degradation;Complex Carbohydrates* |  |
|  | | *Prephenate dehydrogenase* | | *Amino acid synthesis* |  |
|  | | *L-alanine dehydrogenase* | | *Amino acid synthesis* |  |
| 177 | | *Ornithine carbamoyltransferase 1* | | *Amino acid transport and metabolism* |  |
|  | | *Diaminopimelate epimerase* | | *Amino acid synthesis* |  |
|  | | *N-acetyl-D-galactosamine-4-sulfate 4-sulfohydrolase* | | *Glycosaminoglycan degradation* |  |
| 178 | | *Anthranilate synthase* | | *Amino acid transport and metabolism* |  |
|  | | *UDP-N-acetylmuramateL-alanine ligase* | | *Glycan Biosynthesis and Metabolism* |  |
|  | | *Pyruvate-Formate Lyase* | | *Respiration* |  |
| 179 | | *UDP-N-acetylmuramateL-alanine ligase* | | *Glycan Biosynthesis and Metabolism* |  |
|  | | *Pyruvate-Formate Lyase* | | *Respiration* |  |
|  | | *Beta-ketoacyl-acyl-carrier-protein synthase III* | | *Fatty Acid Biosynthesis* |  |
| 180 | | *UDP-N-acetylglucosamine acyltransferase* | | *Glycan Biosynthesis and Metabolism* |  |
|  | | *Folylpolyglutamate Synthase* | | *Cofactor Biosynthesis* |  |
|  | | *Diaminopimelate epimerase* | | *Amino acid synthesis* |  |
| 181 | | *Diaminopimelate epimerase* | | *Amino acid synthesis* |  |
|  | | *Beta-N-acetyl-D-hexosaminide N-acetylhexosaminohydrolase* | | *Glycosaminoglycan degradation* |  |
|  | | *2-isopropylmalate synthase* | | *Amino acid synthesis* |  |
| 182 | | *N-acetyl-D-galactosamine-4-sulfate 4-sulfohydrolase* | | *Glycosaminoglycan degradation* |  |
|  | | *Cobalt Chelatase* | | *Cofactor Biosynthesis* |  |
|  | | *Prephenate dehydrogenase* | | *Amino acid synthesis* |  |
| 183 | | *Cytidylate kinase* | | *Pyrimidine metabolism* |  |
|  | | *L-threonine synthase* | | *Amino acid synthesis* |  |
|  | | *Pyruvate-Formate Lyase* | | *Respiration* |  |
| 184 | | *Mannose-6-Phosphate Isomerase* | | *Feeder Pathways to Glycolysis* |  |
|  | | *Pyruvate-Formate Lyase* | | *Respiration* |  |
|  | | *Alanine racemase* | | *Amino acid synthesis* |  |
| 185 | | *Butyrate Kinase* | | *Organic Acids* |  |
|  | | *Diaminopimelate epimerase* | | *Amino acid synthesis* |  |
|  | | *Carbamoyl phosphate synthetase small subunit glutamine amidotransferase* | | *Pyrimidine metabolism* |  |
| 186 | | *Glycerol Kinase* | | *Glycerolipid Metabolism* |  |
|  | | *N-acetylmuramoyl-L-alanine amidase* | | *Glycan Biosynthesis and Metabolism* |  |
|  | | *N-acetylmuramoyl-L-alanine amidase* | | *Glycan Biosynthesis and Metabolism* |  |
| 187 | | *N-acetyl-D-galactosamine-4-sulfate 4-sulfohydrolase* | | *Glycosaminoglycan degradation* |  |
|  | | *L-threonine aldolase* | | *Amino acid synthesis* |  |
|  | | *Diaminopimelate epimerase* | | *Amino acid synthesis* |  |
| 188 | | *L-Lactate Dehydrogenase* | | *Organic Acids* |  |
|  | | *Threonine ammonia-lyase* | | *Amino acid synthesis* |  |
|  | | *Diaminopimelate epimerase* | | *Amino acid synthesis* |  |
| 189 | | *Glucuronate Isomerase* | | *Feeder Pathways to Glycolysis* |  |
|  | | *N-acetylmuramoyl-L-alanine amidase* | | *Glycan Biosynthesis and Metabolism* |  |
|  | | *Anaerobic ribonucleoside-triphosphate reductase* | | *Pyrimidine metabolism* |  |
| 190 | | *Pyruvate-Formate Lyase* | | *Respiration* |  |
|  | | *UDP-N-acetylmuramoyl-L-alanyl-D-glutamatemeso-diaminopimelate ligase* | | *Glycan Biosynthesis and Metabolism* |  |
|  | | *Spermidine Synthase* | | *Nitrogen Metabolism;Amino acid transport and metabolism* |  |
| 191 | | *Dihydrodipicolinate synthase* | | *Amino acid synthesis* |  |
|  | | *Prephenate dehydrogenase* | | *Amino acid synthesis* |  |
|  | | *Glutamate synthase large and small subunit (NADPH)* | | *Amino acid synthesis* |  |
| 192 | | *Orotidine-5-phosphate decarboxylase* | | *Pyrimidine metabolism* |  |
|  | | *Prephenate dehydrogenase* | | *Amino acid synthesis* |  |
|  | | *Alanine racemase* | | *Amino acid synthesis* |  |
| 193 | | *Formyltetrahydrofolate synthetase* | | *Organic Acids* |  |
|  | | *Formyltetrahydrofolate synthetase* | | *Organic Acids* |  |
|  | | *Prephenate dehydrogenase* | | *Amino acid synthesis* |  |
| 194 | | *Beta-D-galactosidase* | | *Glycan structures - degradation;Complex Carbohydrates* |  |
|  | | *Dihydrodipicolinate synthase* | | *Amino acid synthesis* |  |
|  | | *Diaminopimelate epimerase* | | *Amino acid synthesis* |  |
| 195 | | *L-alanine dehydrogenase* | | *Amino acid synthesis* |  |
|  | | *N-acetylmuramoyl-L-alanine amidase* | | *Glycan Biosynthesis and Metabolism* |  |
|  | | *Butyrate Kinase* | | *Organic Acids* |  |
| 196 | | *Quinolinate Synthase* | | *Cofactor Biosynthesis* |  |
|  | | *Pectinase (Pectinesterase)* | | *Complex Carbohydrates* |  |
|  | | *Diaminopimelate epimerase* | | *Amino acid synthesis* |  |
| 197 | | *Diaminopimelate epimerase* | | *Amino acid synthesis* |  |
|  | | *IMP dehydrogenase* | | *Purine metabolism* |  |
|  | | *Histidinol dehydrogenase* | | *Amino acid transport and metabolism* |  |
| 198 | | *Carbon Monoxide Dehydrogenase* | | *Central Carbon Metabolism Pathways* |  |
|  | | *Prephenate dehydrogenase* | | *Amino acid synthesis* |  |
|  | | *Beta-ketoacyl-acyl-carrier-protein synthase III* | | *Fatty Acid Biosynthesis* |  |
| 199 | | *Ribokinase* | | *Feeder Pathways to Glycolysis* |  |
|  | | *Pyruvate-Formate Lyase* | | *Respiration* |  |
|  | | *L-glutaminase* | | *Amino acid synthesis* |  |
| 200 | | *Transketolase* | | *Central Carbon Metabolism Pathways* |  |
|  | | *Prephenate dehydrogenase* | | *Amino acid synthesis* |  |
|  | | *Glycerol Kinase* | | *Glycerolipid Metabolism* |  |
| 201 | | *Diaminopimelate epimerase* | | *Amino acid synthesis* |  |
|  | | *Cystathionine gamma-synthase* | | *Amino acid synthesis* |  |
|  | | *Formyltetrahydrofolate synthetase* | | *Organic Acids* |  |
| 202 | | *Aspartate-ammonia ligase* | | *Amino acid synthesis* |  |
|  | | *L-Lactate Dehydrogenase* | | *Organic Acids* |  |
|  | | *N-acetylmuramoyl-L-alanine amidase* | | *Glycan Biosynthesis and Metabolism* |  |
| 203 | | *Pyruvate-Formate Lyase* | | *Respiration* |  |
|  | | *Anthranilate synthase* | | *Amino acid transport and metabolism* |  |
|  | | *Asparaginase* | | *Amino acid synthesis* |  |
| 204 | | *Serine hydroxymethyltransferase* | | *Amino acid synthesis* |  |
|  | | *Alpha-Glucosidase* | | *Complex Carbohydrates* |  |
|  | | *Prephenate dehydrogenase* | | *Amino acid synthesis* |  |
| 205 | | *Prephenate dehydrogenase* | | *Amino acid synthesis* |  |
|  | | *Serine-tRNA ligase* | | *Amino acid synthesis* |  |
|  | | *Beta-D-galactosidase* | | *Glycan structures - degradation;Complex Carbohydrates* |  |
| 206 | | *Argininosuccinate lyase* | | *Amino acid transport and metabolism* |  |
|  | | *N-acetylmuramoyl-L-alanine amidase* | | *Glycan Biosynthesis and Metabolism* |  |
|  | | *Asparaginase* | | *Amino acid synthesis* |  |
| 207 | | *Threonine ammonia-lyase* | | *Amino acid synthesis* |  |
|  | | *N-acetyl-D-galactosamine-4-sulfate 4-sulfohydrolase* | | *Glycosaminoglycan degradation* |  |
|  | | *N-acetylmuramoyl-L-alanine amidase* | | *Glycan Biosynthesis and Metabolism* |  |
| 208 | | *Galactokinase* | | *Feeder Pathways to Glycolysis* |  |
|  | | *Diaminopimelate epimerase* | | *Amino acid synthesis* |  |
|  | | *Alpha-Glucosidase* | | *Complex Carbohydrates* |  |
| 209 | | *Transketolase* | | *Central Carbon Metabolism Pathways* |  |
|  | | *N-acetylmuramoyl-L-alanine amidase* | | *Glycan Biosynthesis and Metabolism* |  |
|  | | *Beta-D-galactosidase* | | *Glycan structures - degradation;Complex Carbohydrates* |  |
| 210 | | *4-diphosphocytidyl-2C-methyl-D-erythritol synthase* | | *Isoprenoid biosynthesis* |  |
|  | | *Pyruvate-Formate Lyase* | | *Respiration* |  |
|  | | *Xylose Isomerase* | | *Feeder Pathways to Glycolysis* |  |
| 211 | | *Beta-D-galactosidase* | | *Glycan structures - degradation;Complex Carbohydrates* |  |
|  | | *Cystathionine gamma-synthase* | | *Amino acid synthesis* |  |
|  | | *Prephenate dehydrogenase* | | *Amino acid synthesis* |  |
| 212 | | *Diaminopimelate epimerase* | | *Amino acid synthesis* |  |
|  | | *Acetyl-CoA acyltransferase anaerobic* | | *Fatty Acid Metabolism* |  |
|  | | *Galactokinase* | | *Feeder Pathways to Glycolysis* |  |
| 213 | | *Purine-nucleoside phosphorylase* | | *Purine metabolism* |  |
|  | | *Dihydrodipicolinate synthase* | | *Amino acid synthesis* |  |
|  | | *Pyruvate-Formate Lyase* | | *Respiration* |  |
| 214 | | *Butyrate Kinase* | | *Organic Acids* |  |
|  | | *Argininosuccinate lyase* | | *Amino acid transport and metabolism* |  |
|  | | *N-acetylmuramoyl-L-alanine amidase* | | *Glycan Biosynthesis and Metabolism* |  |
| 215 | | *Pyruvate-Formate Lyase* | | *Respiration* |  |
|  | | *Cysteine synthase A* | | *Amino acid synthesis* |  |
|  | | *Glucuronate Isomerase* | | *Feeder Pathways to Glycolysis* |  |
| 216 | | *Carbamoyl phosphate synthetase small subunit glutamine amidotransferase* | | *Pyrimidine metabolism* |  |
|  | | *Pyruvate-Formate Lyase* | | *Respiration* |  |
|  | | *Ribokinase* | | *Feeder Pathways to Glycolysis* |  |
| 217 | | *3-demethylubiquinone-9 3-methyltransferase* | | *Cofactor Biosynthesis* |  |
|  | | *N-acetylmuramoyl-L-alanine amidase* | | *Glycan Biosynthesis and Metabolism* |  |
|  | | *Biosynthetic arginine decarboxylase PLP-binding* | | *Amino acid transport and metabolism* |  |
| 218 | | *Prephenate dehydrogenase* | | *Amino acid synthesis* |  |
|  | | *Cysteine synthase A* | | *Amino acid synthesis* |  |
|  | | *Beta-D-galactosidase* | | *Glycan structures - degradation;Complex Carbohydrates* |  |
| 219 | | *Cytosine deaminase* | | *Pyrimidine metabolism* |  |
|  | | *UDP-N-acetylmuramoyl-L-alanineD-glutamate ligase* | | *Glycan Biosynthesis and Metabolism* |  |
|  | | *Pyruvate-Formate Lyase* | | *Respiration* |  |
| 220 | | *N-acetyl-D-galactosamine-4-sulfate 4-sulfohydrolase* | | *Glycosaminoglycan degradation* |  |
|  | | *D-alanineD-alanine ligase* | | *Glycan Biosynthesis and Metabolism* |  |
|  | | *Prephenate dehydrogenase* | | *Amino acid synthesis* |  |
| 221 | | *Aspartate kinase* | | *Amino acid synthesis* |  |
|  | | *Diaminopimelate epimerase* | | *Amino acid synthesis* |  |
|  | | *Formyltetrahydrofolate synthetase* | | *Organic Acids* |  |
| 222 | | *3-demethylubiquinone-9 3-methyltransferase* | | *Cofactor Biosynthesis* |  |
|  | | *Prephenate dehydrogenase* | | *Amino acid synthesis* |  |
|  | | *Acetyl-CoA acyltransferase anaerobic* | | *Fatty Acid Metabolism* |  |
| 223 | | *Cysteine synthase A* | | *Amino acid synthesis* |  |
|  | | *Argininosuccinate lyase* | | *Amino acid transport and metabolism* |  |
|  | | *Diaminopimelate epimerase* | | *Amino acid synthesis* |  |
| 224 | | *Prephenate dehydrogenase* | | *Amino acid synthesis* |  |
|  | | *Beta-D-galactosidase* | | *Glycan structures - degradation;Complex Carbohydrates* |  |
|  | | *Adenylosuccinate synthetase* | | *Purine metabolism* |  |
| 225 | | *Glycerol Kinase* | | *Glycerolipid Metabolism* |  |
|  | | *Arginosuccinate synthase* | | *Amino acid synthesis* |  |
|  | | *Diaminopimelate epimerase* | | *Amino acid synthesis* |  |
| 226 | | *Branched-chain-amino-acid transaminase* | | *Amino acid transport and metabolism* |  |
|  | | *Diaminopimelate epimerase* | | *Amino acid synthesis* |  |
|  | | *Cystathionine gamma-synthase* | | *Amino acid synthesis* |  |
| 227 | | *3-demethylubiquinone-9 3-methyltransferase* | | *Cofactor Biosynthesis* |  |
|  | | *N-acetylmuramoyl-L-alanine amidase* | | *Glycan Biosynthesis and Metabolism* |  |
|  | | *Diaminopimelate epimerase* | | *Amino acid synthesis* |  |
| 228 | | *Prephenate dehydrogenase* | | *Amino acid synthesis* |  |
|  | | *Cysteine synthase A* | | *Amino acid synthesis* |  |
|  | | *Anaerobic ribonucleoside-triphosphate reductase* | | *Pyrimidine metabolism* |  |
| 229 | | *Acetyl-CoA acyltransferase anaerobic* | | *Fatty Acid Metabolism* |  |
|  | | *Dihydrodipicolinate reductase* | | *Amino acid transport and metabolism* |  |
|  | | *Diaminopimelate epimerase* | | *Amino acid synthesis* |  |
| 230 | | *N-acetylmuramoyl-L-alanine amidase* | | *Glycan Biosynthesis and Metabolism* |  |
|  | | *D-alanineD-alanine ligase* | | *Glycan Biosynthesis and Metabolism* |  |
|  | | *Alpha-Glucosidase* | | *Complex Carbohydrates* |  |
| 231 | | *N-acetylmuramoyl-L-alanine amidase* | | *Glycan Biosynthesis and Metabolism* |  |
|  | | *1-Phosphofructokinase* | | *Feeder Pathways to Glycolysis* |  |
|  | | *3-deoxy-7-phosphoheptulonate synthase* | | *Amino acid transport and metabolism* |  |
| 232 | | *Diaminopimelate epimerase* | | *Amino acid synthesis* |  |
|  | | *Glutamate racemase* | | *Amino acid synthesis* |  |
|  | | *Ornithine carbamoyltransferase 1* | | *Amino acid transport and metabolism* |  |
| 233 | | *2-isopropylmalate synthase* | | *Amino acid synthesis* |  |
|  | | *Uridine phosphorylase* | | *Pyrimidine metabolism* |  |
|  | | *Diaminopimelate epimerase* | | *Amino acid synthesis* |  |
| 234 | | *Phosphoribosylglycinamide synthetase phosphoribosylamine-glycine ligase* | | *Purine metabolism* |  |
|  | | *Pyruvate-Formate Lyase* | | *Respiration* |  |
|  | | *Beta-N-acetyl-D-hexosaminide N-acetylhexosaminohydrolase* | | *Glycosaminoglycan degradation* |  |
| 235 | | *Cystathionine gamma-synthase* | | *Amino acid synthesis* |  |
|  | | *Pyruvate-Formate Lyase* | | *Respiration* |  |
|  | | *L-glutamine synthase* | | *Amino acid synthesis* |  |
| 236 | | *3-deoxy-7-phosphoheptulonate synthase* | | *Amino acid transport and metabolism* |  |
|  | | *Cysteine synthase A* | | *Amino acid synthesis* |  |
|  | | *Prephenate dehydrogenase* | | *Amino acid synthesis* |  |
| 237 | | *3-demethylubiquinone-9 3-methyltransferase* | | *Cofactor Biosynthesis* |  |
|  | | *Pyruvate-Formate Lyase* | | *Respiration* |  |
|  | | *Beta-D-galactosidase* | | *Glycan structures - degradation;Complex Carbohydrates* |  |
| 238 | | *L-alanine dehydrogenase* | | *Amino acid synthesis* |  |
|  | | *Conjugated Bile Salt Hydrolase* | | *Exotic Metabolisms* |  |
|  | | *Diaminopimelate epimerase* | | *Amino acid synthesis* |  |
| 239 | | *Dihydrodipicolinate synthase* | | *Amino acid synthesis* |  |
|  | | *Beta-N-acetyl-D-hexosaminide N-acetylhexosaminohydrolase* | | *Glycosaminoglycan degradation* |  |
|  | | *Prephenate dehydrogenase* | | *Amino acid synthesis* |  |
| 240 | | *Prephenate dehydrogenase* | | *Amino acid synthesis* |  |
|  | | *Cysteine synthase A* | | *Amino acid synthesis* |  |
|  | | *Cysteine synthase A* | | *Amino acid synthesis* |  |
| 241 | | *Prephenate dehydrogenase* | | *Amino acid synthesis* |  |
|  | | *Cysteine synthase A* | | *Amino acid synthesis* |  |
|  | | *Cytidylate kinase* | | *Pyrimidine metabolism* |  |
| 242 | | *Alpha-Glucosidase* | | *Complex Carbohydrates* |  |
|  | | *Pyruvate-Formate Lyase* | | *Respiration* |  |
|  | | *Diaminopimelate epimerase* | | *Amino acid synthesis* |  |
| 243 | | *Beta-N-acetyl-D-hexosaminide N-acetylhexosaminohydrolase* | | *Glycosaminoglycan degradation* |  |
|  | | *UDP-N-acetylmuramoyl-L-alanyl-D-glutamatemeso-diaminopimelate ligase* | | *Glycan Biosynthesis and Metabolism* |  |
|  | | *Prephenate dehydrogenase* | | *Amino acid synthesis* |  |
| 244 | | *L-threonine aldolase* | | *Amino acid synthesis* |  |
|  | | *Diaminopimelate epimerase* | | *Amino acid synthesis* |  |
|  | | *Formyltetrahydrofolate synthetase* | | *Organic Acids* |  |
| 245 | | *Biosynthetic arginine decarboxylase PLP-binding* | | *Amino acid transport and metabolism* |  |
|  | | *Anaerobic ribonucleoside-triphosphate reductase* | | *Pyrimidine metabolism* |  |
|  | | *N-acetylmuramoyl-L-alanine amidase* | | *Glycan Biosynthesis and Metabolism* |  |
| 246 | | *Purine-nucleoside phosphorylase* | | *Purine metabolism* |  |
|  | | *N-acetylmuramoyl-L-alanine amidase* | | *Glycan Biosynthesis and Metabolism* |  |
|  | | *Aspartate kinase* | | *Amino acid synthesis* |  |
| 247 | | *UDP-N-acetylmuramateL-alanine ligase* | | *Glycan Biosynthesis and Metabolism* |  |
|  | | *UDP-N-acetylglucosamine acyltransferase* | | *Glycan Biosynthesis and Metabolism* |  |
|  | | *N-acetylmuramoyl-L-alanine amidase* | | *Glycan Biosynthesis and Metabolism* |  |
| 248 | | *Agamintase* | | *Nitrogen Metabolism* |  |
|  | | *Pyridoxal Kinase* | | *Cofactor Biosynthesis* |  |
|  | | *Diaminopimelate epimerase* | | *Amino acid synthesis* |  |
| 249 | | *UDP-N-acetylmuramateL-alanine ligase* | | *Glycan Biosynthesis and Metabolism* |  |
|  | | *Diaminopimelate epimerase* | | *Amino acid synthesis* |  |
|  | | *Quinolinate Synthase* | | *Cofactor Biosynthesis* |  |
| 250 | | *Cysteine synthase A* | | *Amino acid synthesis* |  |
|  | | *Prephenate dehydrogenase* | | *Amino acid synthesis* |  |
|  | | *3-deoxy-7-phosphoheptulonate synthase* | | *Amino acid transport and metabolism* |  |
| 251 | | *D-alanineD-alanine ligase* | | *Glycan Biosynthesis and Metabolism* |  |
|  | | *Phosphoribosylglycinamide synthetase phosphoribosylamine-glycine ligase* | | *Purine metabolism* |  |
|  | | *Diaminopimelate epimerase* | | *Amino acid synthesis* |  |
| 252 | | *Pyruvate-Formate Lyase* | | *Respiration* |  |
|  | | *N-acetylglutamate synthase* | | *Amino acid transport and metabolism* |  |
|  | | *Thymidine phosphorylase* | | *Pyrimidine metabolism* |  |
| 253 | | *Glycerol Kinase* | | *Glycerolipid Metabolism* |  |
|  | | *Prephenate dehydrogenase* | | *Amino acid synthesis* |  |
|  | | *Folylpolyglutamate Synthase* | | *Cofactor Biosynthesis* |  |
| 254 | | *L-alanine dehydrogenase* | | *Amino acid synthesis* |  |
|  | | *Pyruvate-Formate Lyase* | | *Respiration* |  |
|  | | *Diaminopimelate epimerase* | | *Amino acid synthesis* |  |
| 255 | | *Orotidine-5-phosphate decarboxylase* | | *Pyrimidine metabolism* |  |
|  | | *Diaminopimelate epimerase* | | *Amino acid synthesis* |  |
|  | | *3-dehydroquinate dehydratase* | | *Amino acid transport and metabolism* |  |
| 256 | | *N-acetylmuramoyl-L-alanine amidase* | | *Glycan Biosynthesis and Metabolism* |  |
|  | | *Pyruvate-Formate Lyase* | | *Respiration* |  |
|  | | *N-acetyl-D-galactosamine-4-sulfate 4-sulfohydrolase* | | *Glycosaminoglycan degradation* |  |
| 257 | | *Prephenate dehydrogenase* | | *Amino acid synthesis* |  |
|  | | *Glutamate synthase large and small subunit (NADPH)* | | *Amino acid synthesis* |  |
|  | | *Formyltetrahydrofolate synthetase* | | *Organic Acids* |  |
| 258 | | *Alpha-Glucosidase* | | *Complex Carbohydrates* |  |
|  | | *N-Acetylglucosamine-6-Phosphate Deacetylase* | | *Feeder Pathways to Glycolysis* |  |
|  | | *N-acetylmuramoyl-L-alanine amidase* | | *Glycan Biosynthesis and Metabolism* |  |
| 259 | | *Branched-chain-amino-acid transaminase* | | *Amino acid transport and metabolism* |  |
|  | | *Pyruvate-Formate Lyase* | | *Respiration* |  |
|  | | *Adenylosuccinate synthetase* | | *Purine metabolism* |  |
| 260 | | *N-acetylmuramoyl-L-alanine amidase* | | *Glycan Biosynthesis and Metabolism* |  |
|  | | *Carbon Monoxide Dehydrogenase* | | *Central Carbon Metabolism Pathways* |  |
|  | | *Beta-D-glucuronidase* | | *Glycan structures - degradation;Exotic Metabolisms* |  |
| 261 | | *Beta-N-acetyl-D-hexosaminide N-acetylhexosaminohydrolase* | | *Glycosaminoglycan degradation* |  |
|  | | *N-acetylmuramoyl-L-alanine amidase* | | *Glycan Biosynthesis and Metabolism* |  |
|  | | *UDP-N-acetylmuramateL-alanine ligase* | | *Glycan Biosynthesis and Metabolism* |  |
| 262 | | *Diaminopimelate epimerase* | | *Amino acid synthesis* |  |
|  | | *Carbamoyl phosphate synthetase small subunit glutamine amidotransferase* | | *Pyrimidine metabolism* |  |
|  | | *Beta-ketoacyl-acyl-carrier-protein synthase III* | | *Fatty Acid Biosynthesis* |  |
| 263 | | *Beta-ketoacyl-acyl-carrier-protein synthase III* | | *Fatty Acid Biosynthesis* |  |
|  | | *Diaminopimelate epimerase* | | *Amino acid synthesis* |  |
|  | | *Methylmalonyl-CaA decarboxylase* | | *Organic Acids* |  |
| 264 | | *Acetyl-CoA acyltransferase anaerobic* | | *Fatty Acid Metabolism* |  |
|  | | *Diaminopimelate epimerase* | | *Amino acid synthesis* |  |
|  | | *Anthranilate synthase* | | *Amino acid transport and metabolism* |  |
| 265 | | *Beta-ketoacyl-acyl-carrier-protein synthase III* | | *Fatty Acid Biosynthesis* |  |
|  | | *Prephenate dehydrogenase* | | *Amino acid synthesis* |  |
|  | | *Alanine racemase* | | *Amino acid synthesis* |  |
| 266 | | *Glucuronate Isomerase* | | *Feeder Pathways to Glycolysis* |  |
|  | | *Cytidylate kinase* | | *Pyrimidine metabolism* |  |
|  | | *Pyruvate-Formate Lyase* | | *Respiration* |  |
| 267 | | *Diaminopimelate epimerase* | | *Amino acid synthesis* |  |
|  | | *Aspartate-ammonia ligase* | | *Amino acid synthesis* |  |
|  | | *Beta-D-galactosidase* | | *Glycan structures - degradation;Complex Carbohydrates* |  |
| 268 | | *Ribokinase* | | *Feeder Pathways to Glycolysis* |  |
|  | | *Cysteine synthase A* | | *Amino acid synthesis* |  |
|  | | *N-acetylmuramoyl-L-alanine amidase* | | *Glycan Biosynthesis and Metabolism* |  |
| 269 | | *N-acetylmuramoyl-L-alanine amidase* | | *Glycan Biosynthesis and Metabolism* |  |
|  | | *Beta-D-glucuronidase* | | *Glycan structures - degradation;Exotic Metabolisms* |  |
|  | | *L-glutamine synthase* | | *Amino acid synthesis* |  |
| 270 | | *3-deoxy-7-phosphoheptulonate synthase* | | *Amino acid transport and metabolism* |  |
|  | | *Diaminopimelate epimerase* | | *Amino acid synthesis* |  |
|  | | *3-isopropylmalate dehydrogenase* | | *Amino acid synthesis* |  |
| 271 | | *Ribokinase* | | *Feeder Pathways to Glycolysis* |  |
|  | | *Diaminopimelate epimerase* | | *Amino acid synthesis* |  |
|  | | *Thioredoxin reductase FAD-NADP-binding* | | *Pyrimidine metabolism* |  |
| 272 | | *Pyruvate-Formate Lyase* | | *Respiration* |  |
|  | | *Prephenate dehydrogenase* | | *Amino acid synthesis* |  |
|  | | *ADP-ribose pyrophosphatase* | | *Purine metabolism* |  |
| 273 | | *Diaminopimelate epimerase* | | *Amino acid synthesis* |  |
|  | | *L-glutamine synthase* | | *Amino acid synthesis* |  |
|  | | *N-acetylmuramoyl-L-alanine amidase* | | *Glycan Biosynthesis and Metabolism* |  |
| 274 | | *L-alanine dehydrogenase* | | *Amino acid synthesis* |  |
|  | | *Pyruvate-Formate Lyase* | | *Respiration* |  |
|  | | *Alpha-mannosidase* | | *N-Glycan degradation* |  |
| 275 | | *Cysteine synthase A* | | *Amino acid synthesis* |  |
|  | | *UDP-N-acetylmuramoyl-L-alanyl-D-glutamatemeso-diaminopimelate ligase* | | *Glycan Biosynthesis and Metabolism* |  |
|  | | *Prephenate dehydrogenase* | | *Amino acid synthesis* |  |
| 276 | | *Ribokinase* | | *Feeder Pathways to Glycolysis* |  |
|  | | *Beta-N-acetyl-D-hexosaminide N-acetylhexosaminohydrolase* | | *Glycosaminoglycan degradation* |  |
|  | | *Diaminopimelate epimerase* | | *Amino acid synthesis* |  |
| 277 | | *Pyruvate-Formate Lyase* | | *Respiration* |  |
|  | | *Adenylosuccinate synthetase* | | *Purine metabolism* |  |
|  | | *Dihydrodipicolinate synthase* | | *Amino acid synthesis* |  |
| 278 | | *Branched-chain-amino-acid transaminase* | | *Amino acid transport and metabolism* |  |
|  | | *Pyruvate-Formate Lyase* | | *Respiration* |  |
|  | | *Glucuronate Isomerase* | | *Feeder Pathways to Glycolysis* |  |
| 279 | | *1-Phosphofructokinase* | | *Feeder Pathways to Glycolysis* |  |
|  | | *Biosynthetic arginine decarboxylase PLP-binding* | | *Amino acid transport and metabolism* |  |
|  | | *Prephenate dehydrogenase* | | *Amino acid synthesis* |  |
| 280 | | *N-acetylmuramoyl-L-alanine amidase* | | *Glycan Biosynthesis and Metabolism* |  |
|  | | *3-deoxy-7-phosphoheptulonate synthase* | | *Amino acid transport and metabolism* |  |
|  | | *Beta-D-galactosidase* | | *Glycan structures - degradation;Complex Carbohydrates* |  |
| 281 | | *Beta-ketoacyl-acyl-carrier-protein synthase III* | | *Fatty Acid Biosynthesis* |  |
|  | | *Pyridoxal Kinase* | | *Cofactor Biosynthesis* |  |
|  | | *Prephenate dehydrogenase* | | *Amino acid synthesis* |  |
| 282 | | *Ribokinase* | | *Feeder Pathways to Glycolysis* |  |
|  | | *Pyruvate-Formate Lyase* | | *Respiration* |  |
|  | | *ADP-ribose pyrophosphatase* | | *Purine metabolism* |  |
| 283 | | *Prephenate dehydrogenase* | | *Amino acid synthesis* |  |
|  | | *Cysteine synthase A* | | *Amino acid synthesis* |  |
|  | | *3-deoxy-7-phosphoheptulonate synthase* | | *Amino acid transport and metabolism* |  |
| 284 | | *L-alanine dehydrogenase* | | *Amino acid synthesis* |  |
|  | | *3-isopropylmalate dehydrogenase* | | *Amino acid synthesis* |  |
|  | | *Pyruvate-Formate Lyase* | | *Respiration* |  |
| 285 | | *IMP dehydrogenase* | | *Purine metabolism* |  |
|  | | *Pyruvate-Formate Lyase* | | *Respiration* |  |
|  | | *Conjugated Bile Salt Hydrolase* | | *Exotic Metabolisms* |  |
| 286 | | *N-acetylmuramoyl-L-alanine amidase* | | *Glycan Biosynthesis and Metabolism* |  |
|  | | *Beta-ketoacyl-acyl-carrier-protein synthase III* | | *Fatty Acid Biosynthesis* |  |
|  | | *Glucuronate Isomerase* | | *Feeder Pathways to Glycolysis* |  |
| 287 | | *Anaerobic ribonucleoside-triphosphate reductase* | | *Pyrimidine metabolism* |  |
|  | | *N-Acetylglucosamine-6-Phosphate Deacetylase* | | *Feeder Pathways to Glycolysis* |  |
|  | | *Pyruvate-Formate Lyase* | | *Respiration* |  |
| 288 | | *Diaminopimelate epimerase* | | *Amino acid synthesis* |  |
|  | | *N-Acetylglucosamine-6-Phosphate Deacetylase* | | *Feeder Pathways to Glycolysis* |  |
|  | | *Histidinol dehydrogenase* | | *Amino acid transport and metabolism* |  |
| 289 | | *Prephenate dehydrogenase* | | *Amino acid synthesis* |  |
|  | | *Alpha-Glucosidase* | | *Complex Carbohydrates* |  |
|  | | *Alanine racemase* | | *Amino acid synthesis* |  |
| 290 | | *Beta-D-galactosidase* | | *Glycan structures - degradation;Complex Carbohydrates* |  |
|  | | *Diaminopimelate epimerase* | | *Amino acid synthesis* |  |
|  | | *Alpha-Glucosidase* | | *Complex Carbohydrates* |  |
| 291 | | *Pyruvate-Formate Lyase* | | *Respiration* |  |
|  | | *Formyltetrahydrofolate synthetase* | | *Organic Acids* |  |
|  | | *N-acetyl-D-galactosamine-4-sulfate 4-sulfohydrolase* | | *Glycosaminoglycan degradation* |  |
| 292 | | *N-acetylmuramoyl-L-alanine amidase* | | *Glycan Biosynthesis and Metabolism* |  |
|  | | *Diaminopimelate epimerase* | | *Amino acid synthesis* |  |
|  | | *Dihydrodipicolinate synthase* | | *Amino acid synthesis* |  |
| 293 | | *Dihydrodipicolinate synthase* | | *Amino acid synthesis* |  |
|  | | *Prephenate dehydrogenase* | | *Amino acid synthesis* |  |
|  | | *Pyridoxal Kinase* | | *Cofactor Biosynthesis* |  |
| 294 | | *Beta-D-glucuronidase* | | *Glycan structures - degradation;Exotic Metabolisms* |  |
|  | | *Carbamoyl phosphate synthetase small subunit glutamine amidotransferase* | | *Pyrimidine metabolism* |  |
|  | | *Prephenate dehydrogenase* | | *Amino acid synthesis* |  |
| 295 | | *Prephenate dehydrogenase* | | *Amino acid synthesis* |  |
|  | | *N-acetylmuramoyl-L-alanine amidase* | | *Glycan Biosynthesis and Metabolism* |  |
|  | | *Alpha-Glucosidase* | | *Complex Carbohydrates* |  |
| 296 | | *Aspartate-ammonia ligase* | | *Amino acid synthesis* |  |
|  | | *N-acetylmuramoyl-L-alanine amidase* | | *Glycan Biosynthesis and Metabolism* |  |
|  | | *Dihydrodipicolinate reductase* | | *Amino acid transport and metabolism* |  |
| 297 | | *Argininosuccinate lyase* | | *Amino acid transport and metabolism* |  |
|  | | *Prephenate dehydrogenase* | | *Amino acid synthesis* |  |
|  | | *Cystathionine gamma-synthase* | | *Amino acid synthesis* |  |
| 298 | | *N-acetylmuramoyl-L-alanine amidase* | | *Glycan Biosynthesis and Metabolism* |  |
|  | | *Alanine racemase* | | *Amino acid synthesis* |  |
|  | | *Acetyl-CoA acyltransferase anaerobic* | | *Fatty Acid Metabolism* |  |
| 299 | | *Adenylosuccinate synthetase* | | *Purine metabolism* |  |
|  | | *Butyrate Kinase* | | *Organic Acids* |  |
|  | | *N-acetylmuramoyl-L-alanine amidase* | | *Glycan Biosynthesis and Metabolism* |  |
| 300 | | *D-alanineD-alanine ligase* | | *Glycan Biosynthesis and Metabolism* |  |
|  | | *Alanine racemase* | | *Amino acid synthesis* |  |
|  | | *Diaminopimelate epimerase* | | *Amino acid synthesis* |  |
| 301 | | *Prephenate dehydrogenase* | | *Amino acid synthesis* |  |
|  | | *3-demethylubiquinone-9 3-methyltransferase* | | *Cofactor Biosynthesis* |  |
|  | | *Cystathionine gamma-synthase* | | *Amino acid synthesis* |  |
| 302 | | *N-acetylmuramoyl-L-alanine amidase* | | *Glycan Biosynthesis and Metabolism* |  |
|  | | *Glutamate racemase* | | *Amino acid synthesis* |  |
|  | | *Alpha-Glucosidase* | | *Complex Carbohydrates* |  |
| 303 | | *Ribokinase* | | *Feeder Pathways to Glycolysis* |  |
|  | | *Prephenate dehydrogenase* | | *Amino acid synthesis* |  |
|  | | *Beta-D-glucuronidase* | | *Glycan structures - degradation;Exotic Metabolisms* |  |
| 304 | | *N-acetylmuramoyl-L-alanine amidase* | | *Glycan Biosynthesis and Metabolism* |  |
|  | | *Riboflavin Synthase α Subunit* | | *Cofactor Biosynthesis* |  |
|  | | *Diaminopimelate epimerase* | | *Amino acid synthesis* |  |
| 305 | | *N-acetylmuramoyl-L-alanine amidase* | | *Glycan Biosynthesis and Metabolism* |  |
|  | | *Cysteine synthase A* | | *Amino acid synthesis* |  |
|  | | *Diaminopimelate epimerase* | | *Amino acid synthesis* |  |
| 306 | | *L-glutamine synthase* | | *Amino acid synthesis* |  |
|  | | *Prephenate dehydrogenase* | | *Amino acid synthesis* |  |
|  | | *1-Phosphofructokinase* | | *Feeder Pathways to Glycolysis* |  |
| 307 | | *Beta-D-glucuronidase* | | *Glycan structures - degradation;Exotic Metabolisms* |  |
|  | | *Ornithine carbamoyltransferase 1* | | *Amino acid transport and metabolism* |  |
|  | | *N-acetylmuramoyl-L-alanine amidase* | | *Glycan Biosynthesis and Metabolism* |  |
| 308 | | *Diaminopimelate epimerase* | | *Amino acid synthesis* |  |
|  | | *Biosynthetic arginine decarboxylase PLP-binding* | | *Amino acid transport and metabolism* |  |
|  | | *Orotidine-5-phosphate decarboxylase* | | *Pyrimidine metabolism* |  |
| 309 | | *Carbon Monoxide Dehydrogenase* | | *Central Carbon Metabolism Pathways* |  |
|  | | *N-acetylmuramoyl-L-alanine amidase* | | *Glycan Biosynthesis and Metabolism* |  |
|  | | *Agamintase* | | *Nitrogen Metabolism* |  |
| 310 | | *N-acetylmuramoyl-L-alanine amidase* | | *Glycan Biosynthesis and Metabolism* |  |
|  | | *Uridine phosphorylase* | | *Pyrimidine metabolism* |  |
|  | | *Dihydrodipicolinate synthase* | | *Amino acid synthesis* |  |
| 311 | | *Diaminopimelate epimerase* | | *Amino acid synthesis* |  |
|  | | *Phosphoribosylglycinamide synthetase phosphoribosylamine-glycine ligase* | | *Purine metabolism* |  |
|  | | *Glutamate synthase large and small subunit (NADPH)* | | *Amino acid synthesis* |  |
| 312 | | *Aspartate-ammonia ligase* | | *Amino acid synthesis* |  |
|  | | *Diaminopimelate epimerase* | | *Amino acid synthesis* |  |
|  | | *Dihydrodipicolinate synthase* | | *Amino acid synthesis* |  |
| 313 | | *L-alanine dehydrogenase* | | *Amino acid synthesis* |  |
|  | | *Pyruvate-Formate Lyase* | | *Respiration* |  |
|  | | *Diaminopimelate epimerase* | | *Amino acid synthesis* |  |
| 314 | | *Dihydrodipicolinate synthase* | | *Amino acid synthesis* |  |
|  | | *Pyruvate-Formate Lyase* | | *Respiration* |  |
|  | | *Diaminopimelate epimerase* | | *Amino acid synthesis* |  |
| 315 | | *Thymidine phosphorylase* | | *Pyrimidine metabolism* |  |
|  | | *Histidinol dehydrogenase* | | *Amino acid transport and metabolism* |  |
|  | | *Prephenate dehydrogenase* | | *Amino acid synthesis* |  |
| 316 | | *Anaerobic ribonucleoside-triphosphate reductase* | | *Pyrimidine metabolism* |  |
|  | | *Anthranilate synthase* | | *Amino acid transport and metabolism* |  |
|  | | *Diaminopimelate epimerase* | | *Amino acid synthesis* |  |
| 317 | | *Beta-N-acetyl-D-hexosaminide N-acetylhexosaminohydrolase* | | *Glycosaminoglycan degradation* |  |
|  | | *Pyruvate-Formate Lyase* | | *Respiration* |  |
|  | | *D-alanineD-alanine ligase* | | *Glycan Biosynthesis and Metabolism* |  |
| 318 | | *Formyltetrahydrofolate synthetase* | | *Organic Acids* |  |
|  | | *Formyltetrahydrofolate synthetase* | | *Organic Acids* |  |
|  | | *Prephenate dehydrogenase* | | *Amino acid synthesis* |  |
| 319 | | *Adenylosuccinate synthetase* | | *Purine metabolism* |  |
|  | | *Prephenate dehydrogenase* | | *Amino acid synthesis* |  |
|  | | *Phosphoribosylglycinamide synthetase phosphoribosylamine-glycine ligase* | | *Purine metabolism* |  |
| 320 | | *Diaminopimelate epimerase* | | *Amino acid synthesis* |  |
|  | | *Carbamoyl phosphate synthetase small subunit glutamine amidotransferase* | | *Pyrimidine metabolism* |  |
|  | | *Adenylosuccinate synthetase* | | *Purine metabolism* |  |
| 321 | | *L-threonine synthase* | | *Amino acid synthesis* |  |
|  | | *Pyruvate-Formate Lyase* | | *Respiration* |  |
|  | | *Folylpolyglutamate Synthase* | | *Cofactor Biosynthesis* |  |
| 322 | | *Beta-ketoacyl-acyl-carrier-protein synthase III* | | *Fatty Acid Biosynthesis* |  |
|  | | *Folylpolyglutamate Synthase* | | *Cofactor Biosynthesis* |  |
|  | | *Pyruvate-Formate Lyase* | | *Respiration* |  |
| 323 | | *3-deoxy-7-phosphoheptulonate synthase* | | *Amino acid transport and metabolism* |  |
|  | | *Diaminopimelate epimerase* | | *Amino acid synthesis* |  |
|  | | *Glutamate synthase large and small subunit (NADPH)* | | *Amino acid synthesis* |  |
| 324 | | *Glutamate synthase large and small subunit (NADPH)* | | *Amino acid synthesis* |  |
|  | | *UDP-N-acetylmuramoyl-L-alanyl-D-glutamatemeso-diaminopimelate ligase* | | *Glycan Biosynthesis and Metabolism* |  |
|  | | *N-acetylmuramoyl-L-alanine amidase* | | *Glycan Biosynthesis and Metabolism* |  |
| 325 | | *Agamintase* | | *Nitrogen Metabolism* |  |
|  | | *Diaminopimelate epimerase* | | *Amino acid synthesis* |  |
|  | | *Thioredoxin reductase FAD-NADP-binding* | | *Pyrimidine metabolism* |  |
| 326 | | *Anthranilate synthase* | | *Amino acid transport and metabolism* |  |
|  | | *UDP-N-acetylmuramateL-alanine ligase* | | *Glycan Biosynthesis and Metabolism* |  |
|  | | *Prephenate dehydrogenase* | | *Amino acid synthesis* |  |
| 327 | | *Glutamate synthase large and small subunit (NADPH)* | | *Amino acid synthesis* |  |
|  | | *Glutamate synthase large and small subunit (NADPH)* | | *Amino acid synthesis* |  |
|  | | *Pyruvate-Formate Lyase* | | *Respiration* |  |
| 328 | | *N-acetyl-D-galactosamine-4-sulfate 4-sulfohydrolase* | | *Glycosaminoglycan degradation* |  |
|  | | *Pyruvate-Formate Lyase* | | *Respiration* |  |
|  | | *Histidinol dehydrogenase* | | *Amino acid transport and metabolism* |  |
| 329 | | *Pyrroline-5-carboxylate reductase* | | *Amino acid transport and metabolism* |  |
|  | | *Prephenate dehydrogenase* | | *Amino acid synthesis* |  |
|  | | *Cytidylate kinase* | | *Pyrimidine metabolism* |  |
| 330 | | *Homoserine dehydrogenase* | | *Amino acid transport and metabolism* |  |
|  | | *Glutamate racemase* | | *Amino acid synthesis* |  |
|  | | *Pyruvate-Formate Lyase* | | *Respiration* |  |
| 331 | | *Cytosine deaminase* | | *Pyrimidine metabolism* |  |
|  | | *Branched-chain-amino-acid transaminase* | | *Amino acid transport and metabolism* |  |
|  | | *Mannanase (beta-mannosidase)* | | *Complex Carbohydrates* |  |
| 332 | | *Pyruvate-Formate Lyase* | | *Respiration* |  |
|  | | *Histidinol dehydrogenase* | | *Amino acid transport and metabolism* |  |
|  | | *Thioredoxin reductase FAD-NADP-binding* | | *Pyrimidine metabolism* |  |
| 333 | | *Alpha-Glucosidase* | | *Complex Carbohydrates* |  |
|  | | *1-Phosphofructokinase* | | *Feeder Pathways to Glycolysis* |  |
|  | | *N-acetylmuramoyl-L-alanine amidase* | | *Glycan Biosynthesis and Metabolism* |  |
| 334 | | *Arginosuccinate synthase* | | *Amino acid synthesis* |  |
|  | | *Spermidine Synthase* | | *Nitrogen Metabolism;Amino acid transport and metabolism* |  |
|  | | *Prephenate dehydrogenase* | | *Amino acid synthesis* |  |
| 335 | | *Dihydrodipicolinate synthase* | | *Amino acid synthesis* |  |
|  | | *Carbon Monoxide Dehydrogenase* | | *Central Carbon Metabolism Pathways* |  |
|  | | *Prephenate dehydrogenase* | | *Amino acid synthesis* |  |
| 336 | | *L-glutamine synthase* | | *Amino acid synthesis* |  |
|  | | *Cytidylate kinase* | | *Pyrimidine metabolism* |  |
|  | | *Pyruvate-Formate Lyase* | | *Respiration* |  |
| 337 | | *Pyruvate-Formate Lyase* | | *Respiration* |  |
|  | | *Ribokinase* | | *Feeder Pathways to Glycolysis* |  |
|  | | *Histidinol dehydrogenase* | | *Amino acid transport and metabolism* |  |
| 338 | | *Prephenate dehydrogenase* | | *Amino acid synthesis* |  |
|  | | *Formyltetrahydrofolate synthetase* | | *Organic Acids* |  |
|  | | *Selenocysteine synthase* | | *Amino acid synthesis;Amino acid synthesis* |  |
| 339 | | *Glutamate synthase large and small subunit (NADPH)* | | *Amino acid synthesis* |  |
|  | | *Methylmalonyl-CaA decarboxylase* | | *Organic Acids* |  |
|  | | *Pyruvate-Formate Lyase* | | *Respiration* |  |
| 340 | | *Prephenate dehydrogenase* | | *Amino acid synthesis* |  |
|  | | *Geranyltranstransferase* | | *Isoprenoid biosynthesis* |  |
|  | | *Dihydrodipicolinate synthase* | | *Amino acid synthesis* |  |
| 341 | | *KDPG Aldolase* | | *Central Carbon Metabolism Pathways* |  |
|  | | *Cytosine deaminase* | | *Pyrimidine metabolism* |  |
|  | | *Prephenate dehydrogenase* | | *Amino acid synthesis* |  |
| 342 | | *Acetyl-CoA acyltransferase anaerobic* | | *Fatty Acid Metabolism* |  |
|  | | *Thioredoxin reductase FAD-NADP-binding* | | *Pyrimidine metabolism* |  |
|  | | *Diaminopimelate epimerase* | | *Amino acid synthesis* |  |
| 343 | | *Homoserine dehydrogenase* | | *Amino acid transport and metabolism* |  |
|  | | *Acetylglutamate kinase* | | *Amino acid transport and metabolism* |  |
|  | | *N-acetylmuramoyl-L-alanine amidase* | | *Glycan Biosynthesis and Metabolism* |  |
| 344 | | *Beta-D-glucuronidase* | | *Glycan structures - degradation;Exotic Metabolisms* |  |
|  | | *Beta-N-acetyl-D-hexosaminide N-acetylhexosaminohydrolase* | | *Glycosaminoglycan degradation* |  |
|  | | *N-acetylmuramoyl-L-alanine amidase* | | *Glycan Biosynthesis and Metabolism* |  |
| 345 | | *Prephenate dehydrogenase* | | *Amino acid synthesis* |  |
|  | | *Alpha-Glucosidase* | | *Complex Carbohydrates* |  |
|  | | *N-acetyl-D-galactosamine-4-sulfate 4-sulfohydrolase* | | *Glycosaminoglycan degradation* |  |
| 346 | | *Ribokinase* | | *Feeder Pathways to Glycolysis* |  |
|  | | *Thioredoxin reductase FAD-NADP-binding* | | *Pyrimidine metabolism* |  |
|  | | *Prephenate dehydrogenase* | | *Amino acid synthesis* |  |
| 347 | | *Prephenate dehydrogenase* | | *Amino acid synthesis* |  |
|  | | *Cysteine synthase A* | | *Amino acid synthesis* |  |
|  | | *Alanine racemase* | | *Amino acid synthesis* |  |
| 348 | | *Transketolase* | | *Central Carbon Metabolism Pathways* |  |
|  | | *Ribokinase* | | *Feeder Pathways to Glycolysis* |  |
|  | | *N-acetylmuramoyl-L-alanine amidase* | | *Glycan Biosynthesis and Metabolism* |  |
| 349 | | *Histidinol dehydrogenase* | | *Amino acid transport and metabolism* |  |
|  | | *N-acetyl-D-galactosamine-4-sulfate 4-sulfohydrolase* | | *Glycosaminoglycan degradation* |  |
|  | | *Pyruvate-Formate Lyase* | | *Respiration* |  |
| 350 | | *N-acetylmuramoyl-L-alanine amidase* | | *Glycan Biosynthesis and Metabolism* |  |
|  | | *N-acetylmuramoyl-L-alanine amidase* | | *Glycan Biosynthesis and Metabolism* |  |
|  | | *Beta-D-galactosidase* | | *Glycan structures - degradation;Complex Carbohydrates* |  |
| 351 | | *ketol-acid reductoisomerase* | | *Amino acid synthesis* |  |
|  | | *Pyruvate-Formate Lyase* | | *Respiration* |  |
|  | | *Homoserine dehydrogenase* | | *Amino acid transport and metabolism* |  |
| 352 | | *Diaminopimelate epimerase* | | *Amino acid synthesis* |  |
|  | | *Adenylosuccinate synthetase* | | *Purine metabolism* |  |
|  | | *Histidinol dehydrogenase* | | *Amino acid transport and metabolism* |  |
| 353 | | *Histidinol dehydrogenase* | | *Amino acid transport and metabolism* |  |
|  | | *Purine-nucleoside phosphorylase* | | *Purine metabolism* |  |
|  | | *Pyruvate-Formate Lyase* | | *Respiration* |  |
| 354 | | *Adenylosuccinate synthetase* | | *Purine metabolism* |  |
|  | | *Glucuronate Isomerase* | | *Feeder Pathways to Glycolysis* |  |
|  | | *Prephenate dehydrogenase* | | *Amino acid synthesis* |  |
| 355 | | *3-isopropylmalate dehydrogenase* | | *Amino acid synthesis* |  |
|  | | *UDP-N-acetylmuramateL-alanine ligase* | | *Glycan Biosynthesis and Metabolism* |  |
|  | | *Prephenate dehydrogenase* | | *Amino acid synthesis* |  |
| 356 | | *Prephenate dehydrogenase* | | *Amino acid synthesis* |  |
|  | | *N-acetyl-D-galactosamine-4-sulfate 4-sulfohydrolase* | | *Glycosaminoglycan degradation* |  |
|  | | *Cysteine synthase A* | | *Amino acid synthesis* |  |
| 357 | | *Cytidylate kinase* | | *Pyrimidine metabolism* |  |
|  | | *Prephenate dehydrogenase* | | *Amino acid synthesis* |  |
|  | | *L-glutamine synthase* | | *Amino acid synthesis* |  |
| 358 | | *Pyrroline-5-carboxylate reductase* | | *Amino acid transport and metabolism* |  |
|  | | *Histidinol dehydrogenase* | | *Amino acid transport and metabolism* |  |
|  | | *N-acetylmuramoyl-L-alanine amidase* | | *Glycan Biosynthesis and Metabolism* |  |
| 359 | | *N-acetylmuramoyl-L-alanine amidase* | | *Glycan Biosynthesis and Metabolism* |  |
|  | | *UDP-N-acetylmuramateL-alanine ligase* | | *Glycan Biosynthesis and Metabolism* |  |
|  | | *Homoserine kinase* | | *Amino acid transport and metabolism* |  |
| 360 | | *Diaminopimelate epimerase* | | *Amino acid synthesis* |  |
|  | | *Cystathionine gamma-synthase* | | *Amino acid synthesis* |  |
|  | | *Alpha-Glucosidase* | | *Complex Carbohydrates* |  |
| 361 | | *N-acetyl-D-glucosamine-6-sulfate 6-sulfohydrolase* | | *Glycosaminoglycan degradation* |  |
|  | | *Diaminopimelate epimerase* | | *Amino acid synthesis* |  |
|  | | *Aspartate kinase* | | *Amino acid synthesis* |  |
| 362 | | *Galactokinase* | | *Feeder Pathways to Glycolysis* |  |
|  | | *Si-Citrate Synthase* | | *Central Carbon Metabolism Pathways* |  |
|  | | *Diaminopimelate epimerase* | | *Amino acid synthesis* |  |
| 363 | | *Diaminopimelate epimerase* | | *Amino acid synthesis* |  |
|  | | *Adenylosuccinate synthetase* | | *Purine metabolism* |  |
|  | | *N-Acetylglucosamine-6-Phosphate Deacetylase* | | *Feeder Pathways to Glycolysis* |  |
| 364 | | *Diaminopimelate epimerase* | | *Amino acid synthesis* |  |
|  | | *Cystathionine gamma-synthase* | | *Amino acid synthesis* |  |
|  | | *Thymidine phosphorylase* | | *Pyrimidine metabolism* |  |
| 365 | | *Galactokinase* | | *Feeder Pathways to Glycolysis* |  |
|  | | *Diaminopimelate epimerase* | | *Amino acid synthesis* |  |
|  | | *Beta-N-acetyl-D-hexosaminide N-acetylhexosaminohydrolase* | | *Glycosaminoglycan degradation* |  |
| 366 | | *Prephenate dehydrogenase* | | *Amino acid synthesis* |  |
|  | | *IMP dehydrogenase* | | *Purine metabolism* |  |
|  | | *Glutamate racemase* | | *Amino acid synthesis* |  |
| 367 | | *3-demethylubiquinone-9 3-methyltransferase* | | *Cofactor Biosynthesis* |  |
|  | | *Pyruvate-Formate Lyase* | | *Respiration* |  |
|  | | *Ribokinase* | | *Feeder Pathways to Glycolysis* |  |
| 368 | | *Riboflavin Synthase α Subunit* | | *Cofactor Biosynthesis* |  |
|  | | *N-acetylmuramoyl-L-alanine amidase* | | *Glycan Biosynthesis and Metabolism* |  |
|  | | *Transketolase* | | *Central Carbon Metabolism Pathways* |  |
| 369 | | *Pyruvate-Formate Lyase* | | *Respiration* |  |
|  | | *N-Acetylglucosamine-6-Phosphate Deacetylase* | | *Feeder Pathways to Glycolysis* |  |
|  | | *Threonine ammonia-lyase* | | *Amino acid synthesis* |  |
| 370 | | *N-acetylmuramoyl-L-alanine amidase* | | *Glycan Biosynthesis and Metabolism* |  |
|  | | *Glutamate racemase* | | *Amino acid synthesis* |  |
|  | | *Beta-N-acetyl-D-hexosaminide N-acetylhexosaminohydrolase* | | *Glycosaminoglycan degradation* |  |
| 371 | | *L-alanine dehydrogenase* | | *Amino acid synthesis* |  |
|  | | *N-acetylmuramoyl-L-alanine amidase* | | *Glycan Biosynthesis and Metabolism* |  |
|  | | *Cobalamin Synthase* | | *Cofactor Biosynthesis* |  |
| 372 | | *Adenylosuccinate synthetase* | | *Purine metabolism* |  |
|  | | *Ornithine carbamoyltransferase 1* | | *Amino acid transport and metabolism* |  |
|  | | *Diaminopimelate epimerase* | | *Amino acid synthesis* |  |
| 373 | | *Cytidylate kinase* | | *Pyrimidine metabolism* |  |
|  | | *Butyrate Kinase* | | *Organic Acids* |  |
|  | | *Diaminopimelate epimerase* | | *Amino acid synthesis* |  |
| 374 | | *Pyruvate-Formate Lyase* | | *Respiration* |  |
|  | | *Homoserine dehydrogenase* | | *Amino acid transport and metabolism* |  |
|  | | *UDP-N-acetylmuramateL-alanine ligase* | | *Glycan Biosynthesis and Metabolism* |  |
| 375 | | *UDP-N-acetylmuramateL-alanine ligase* | | *Glycan Biosynthesis and Metabolism* |  |
|  | | *Glutamate synthase large and small subunit (NADPH)* | | *Amino acid synthesis* |  |
|  | | *Diaminopimelate epimerase* | | *Amino acid synthesis* |  |
| 376 | | *Alpha-Glucosidase* | | *Complex Carbohydrates* |  |
|  | | *Ornithine carbamoyltransferase 1* | | *Amino acid transport and metabolism* |  |
|  | | *Prephenate dehydrogenase* | | *Amino acid synthesis* |  |
| 377 | | *Glycerol Kinase* | | *Glycerolipid Metabolism* |  |
|  | | *Ribokinase* | | *Feeder Pathways to Glycolysis* |  |
|  | | *Diaminopimelate epimerase* | | *Amino acid synthesis* |  |
| 378 | | *L-alanine dehydrogenase* | | *Amino acid synthesis* |  |
|  | | *Conjugated Bile Salt Hydrolase* | | *Exotic Metabolisms* |  |
|  | | *Pyruvate-Formate Lyase* | | *Respiration* |  |
| 379 | | *N-acetylmuramoyl-L-alanine amidase* | | *Glycan Biosynthesis and Metabolism* |  |
|  | | *3-demethylubiquinone-9 3-methyltransferase* | | *Cofactor Biosynthesis* |  |
|  | | *Beta-D-galactosidase* | | *Glycan structures - degradation;Complex Carbohydrates* |  |
| 380 | | *3-deoxy-7-phosphoheptulonate synthase* | | *Amino acid transport and metabolism* |  |
|  | | *Prephenate dehydrogenase* | | *Amino acid synthesis* |  |
|  | | *Dihydrodipicolinate reductase* | | *Amino acid transport and metabolism* |  |
| 381 | | *Pyruvate-Formate Lyase* | | *Respiration* |  |
|  | | *Methylmalonyl-CaA decarboxylase* | | *Organic Acids* |  |
|  | | *N-acetyl-D-galactosamine-4-sulfate 4-sulfohydrolase* | | *Glycosaminoglycan degradation* |  |
| 382 | | *Diaminopimelate epimerase* | | *Amino acid synthesis* |  |
|  | | *Pyruvate-Formate Lyase* | | *Respiration* |  |
|  | | *Alanine racemase* | | *Amino acid synthesis* |  |
| 383 | | *Acetyl-CoA acyltransferase anaerobic* | | *Fatty Acid Metabolism* |  |
|  | | *Dihydrodipicolinate synthase* | | *Amino acid synthesis* |  |
|  | | *Diaminopimelate epimerase* | | *Amino acid synthesis* |  |
| 384 | | *Prephenate dehydrogenase* | | *Amino acid synthesis* |  |
|  | | *UDP-N-acetylmuramateL-alanine ligase* | | *Glycan Biosynthesis and Metabolism* |  |
|  | | *Biotin Synthase* | | *Cofactor Biosynthesis* |  |
| 385 | | *N-acetylmuramoyl-L-alanine amidase* | | *Glycan Biosynthesis and Metabolism* |  |
|  | | *Anaerobic ribonucleoside-triphosphate reductase* | | *Pyrimidine metabolism* |  |
|  | | *IMP dehydrogenase* | | *Purine metabolism* |  |
| 386 | | *Uridine phosphorylase* | | *Pyrimidine metabolism* |  |
|  | | *Diaminopimelate epimerase* | | *Amino acid synthesis* |  |
|  | | *Glycerol Kinase* | | *Glycerolipid Metabolism* |  |
| 387 | | *Phosphoribosylglycinamide synthetase phosphoribosylamine-glycine ligase* | | *Purine metabolism* |  |
|  | | *N-acetylmuramoyl-L-alanine amidase* | | *Glycan Biosynthesis and Metabolism* |  |
|  | | *Anaerobic ribonucleoside-triphosphate reductase* | | *Pyrimidine metabolism* |  |
| 388 | | *Prephenate dehydrogenase* | | *Amino acid synthesis* |  |
|  | | *Biosynthetic arginine decarboxylase PLP-binding* | | *Amino acid transport and metabolism* |  |
|  | | *Formyltetrahydrofolate synthetase* | | *Organic Acids* |  |
| 389 | | *UDP-N-acetylmuramoyl-L-alanineD-glutamate ligase* | | *Glycan Biosynthesis and Metabolism* |  |
|  | | *Prephenate dehydrogenase* | | *Amino acid synthesis* |  |
|  | | *N-acetylmuramoyl-L-alanine amidase* | | *Glycan Biosynthesis and Metabolism* |  |
| 390 | | *Alanine racemase* | | *Amino acid synthesis* |  |
|  | | *N-acetyl-D-galactosamine-4-sulfate 4-sulfohydrolase* | | *Glycosaminoglycan degradation* |  |
|  | | *Prephenate dehydrogenase* | | *Amino acid synthesis* |  |
| 391 | | *Pyruvate-Formate Lyase* | | *Respiration* |  |
|  | | *Anthranilate synthase* | | *Amino acid transport and metabolism* |  |
|  | | *Alpha-Glucosidase* | | *Complex Carbohydrates* |  |
| 392 | | *Glycerol Kinase* | | *Glycerolipid Metabolism* |  |
|  | | *Beta-N-acetyl-D-hexosaminide N-acetylhexosaminohydrolase* | | *Glycosaminoglycan degradation* |  |
|  | | *Diaminopimelate epimerase* | | *Amino acid synthesis* |  |
| 393 | | *Butyrate Kinase* | | *Organic Acids* |  |
|  | | *Cytidylate kinase* | | *Pyrimidine metabolism* |  |
|  | | *Pyruvate-Formate Lyase* | | *Respiration* |  |
| 394 | | *N-acetylmuramoyl-L-alanine amidase* | | *Glycan Biosynthesis and Metabolism* |  |
|  | | *Butyrate Kinase* | | *Organic Acids* |  |
|  | | *Ornithine carbamoyltransferase 1* | | *Amino acid transport and metabolism* |  |
| 395 | | *Biosynthetic arginine decarboxylase PLP-binding* | | *Amino acid transport and metabolism* |  |
|  | | *Prephenate dehydrogenase* | | *Amino acid synthesis* |  |
|  | | *Ornithine carbamoyltransferase 1* | | *Amino acid transport and metabolism* |  |
| 396 | | *N-acetylmuramoyl-L-alanine amidase* | | *Glycan Biosynthesis and Metabolism* |  |
|  | | *Beta-ketoacyl-acyl-carrier-protein synthase III* | | *Fatty Acid Biosynthesis* |  |
|  | | *Formyltetrahydrofolate synthetase* | | *Organic Acids* |  |
| 397 | | *Acetate Kinase* | | *Organic Acids* |  |
|  | | *Diaminopimelate epimerase* | | *Amino acid synthesis* |  |
|  | | *Acetyl-CoA acyltransferase anaerobic* | | *Fatty Acid Metabolism* |  |
| 398 | | *Pyruvate-Formate Lyase* | | *Respiration* |  |
|  | | *Beta-ketoacyl-acyl-carrier-protein synthase III* | | *Fatty Acid Biosynthesis* |  |
|  | | *Acetylglutamate kinase* | | *Amino acid transport and metabolism* |  |
| 399 | | *Diaminopimelate epimerase* | | *Amino acid synthesis* |  |
|  | | *Methylmalonyl-CaA decarboxylase* | | *Organic Acids* |  |
|  | | *Alpha-Glucosidase* | | *Complex Carbohydrates* |  |
| 400 | | *Histidinol dehydrogenase* | | *Amino acid transport and metabolism* |  |
|  | | *N-acetylmuramoyl-L-alanine amidase* | | *Glycan Biosynthesis and Metabolism* |  |
|  | | *N-Acetylglucosamine-6-Phosphate Deacetylase* | | *Feeder Pathways to Glycolysis* |  |
| 401 | | *Prephenate dehydrogenase* | | *Amino acid synthesis* |  |
|  | | *N-acetyl-D-galactosamine-4-sulfate 4-sulfohydrolase* | | *Glycosaminoglycan degradation* |  |
|  | | *3-dehydroquinate dehydratase* | | *Amino acid transport and metabolism* |  |
| 402 | | *Aspartate kinase* | | *Amino acid synthesis* |  |
|  | | *3-deoxy-7-phosphoheptulonate synthase* | | *Amino acid transport and metabolism* |  |
|  | | *Pyruvate-Formate Lyase* | | *Respiration* |  |
| 403 | | *Cytidylate kinase* | | *Pyrimidine metabolism* |  |
|  | | *Thioredoxin reductase FAD-NADP-binding* | | *Pyrimidine metabolism* |  |
|  | | *Prephenate dehydrogenase* | | *Amino acid synthesis* |  |
| 404 | | *Argininosuccinate lyase* | | *Amino acid transport and metabolism* |  |
|  | | *Butyryl CoA Acetate CoA Transferase* | | *Organic Acids* |  |
|  | | *Diaminopimelate epimerase* | | *Amino acid synthesis* |  |
| 405 | | *Fucose Isomerase* | | *Feeder Pathways to Glycolysis* |  |
|  | | *UDP-N-acetylmuramateL-alanine ligase* | | *Glycan Biosynthesis and Metabolism* |  |
|  | | *Pyruvate-Formate Lyase* | | *Respiration* |  |
| 406 | | *D-alanineD-alanine ligase* | | *Glycan Biosynthesis and Metabolism* |  |
|  | | *N-acetyl-D-galactosamine-4-sulfate 4-sulfohydrolase* | | *Glycosaminoglycan degradation* |  |
|  | | *Pyruvate-Formate Lyase* | | *Respiration* |  |
| 407 | | *Prephenate dehydrogenase* | | *Amino acid synthesis* |  |
|  | | *Aspartate-ammonia ligase* | | *Amino acid synthesis* |  |
|  | | *Selenocysteine synthase* | | *Amino acid synthesis;Amino acid synthesis* |  |
| 408 | | *1-Phosphofructokinase* | | *Feeder Pathways to Glycolysis* |  |
|  | | *Pyruvate-Formate Lyase* | | *Respiration* |  |
|  | | *Cytosine deaminase* | | *Pyrimidine metabolism* |  |
| 409 | | *Beta-N-acetyl-D-hexosaminide N-acetylhexosaminohydrolase* | | *Glycosaminoglycan degradation* |  |
|  | | *Riboflavin Synthase α Subunit* | | *Cofactor Biosynthesis* |  |
|  | | *Diaminopimelate epimerase* | | *Amino acid synthesis* |  |
| 410 | | *Pyruvate-Formate Lyase* | | *Respiration* |  |
|  | | *Butyrate Kinase* | | *Organic Acids* |  |
|  | | *Formyltetrahydrofolate synthetase* | | *Organic Acids* |  |
| 411 | | *Ornithine carbamoyltransferase 1* | | *Amino acid transport and metabolism* |  |
|  | | *ADP-ribose pyrophosphatase* | | *Purine metabolism* |  |
|  | | *N-acetylmuramoyl-L-alanine amidase* | | *Glycan Biosynthesis and Metabolism* |  |
| 412 | | *Transketolase* | | *Central Carbon Metabolism Pathways* |  |
|  | | *Pyruvate-Formate Lyase* | | *Respiration* |  |
|  | | *Chorismate synthase* | | *Amino acid transport and metabolism* |  |
| 413 | | *N-acetylmuramoyl-L-alanine amidase* | | *Glycan Biosynthesis and Metabolism* |  |
|  | | *N-acetylmuramoyl-L-alanine amidase* | | *Glycan Biosynthesis and Metabolism* |  |
|  | | *Butyrate Kinase* | | *Organic Acids* |  |
| 414 | | *Pyruvate-Formate Lyase* | | *Respiration* |  |
|  | | *Glutamate synthase large and small subunit (NADPH)* | | *Amino acid synthesis* |  |
|  | | *Xylose Isomerase* | | *Feeder Pathways to Glycolysis* |  |
| 415 | | *Thioredoxin reductase FAD-NADP-binding* | | *Pyrimidine metabolism* |  |
|  | | *Carbon Monoxide Dehydrogenase* | | *Central Carbon Metabolism Pathways* |  |
|  | | *N-acetylmuramoyl-L-alanine amidase* | | *Glycan Biosynthesis and Metabolism* |  |
| 416 | | *Prephenate dehydrogenase* | | *Amino acid synthesis* |  |
|  | | *Pyridoxal Kinase* | | *Cofactor Biosynthesis* |  |
|  | | *Carbamoyl phosphate synthetase small subunit glutamine amidotransferase* | | *Pyrimidine metabolism* |  |
| 417 | | *Diaminopimelate epimerase* | | *Amino acid synthesis* |  |
|  | | *Glycerol Kinase* | | *Glycerolipid Metabolism* |  |
|  | | *Cysteine synthase A* | | *Amino acid synthesis* |  |
| 418 | | *3-isopropylmalate dehydrogenase* | | *Amino acid synthesis* |  |
|  | | *UDP-N-acetylmuramoyl-L-alanyl-D-glutamatemeso-diaminopimelate ligase* | | *Glycan Biosynthesis and Metabolism* |  |
|  | | *Diaminopimelate epimerase* | | *Amino acid synthesis* |  |
| 419 | | *Arginosuccinate synthase* | | *Amino acid synthesis* |  |
|  | | *N-acetyl-D-galactosamine-4-sulfate 4-sulfohydrolase* | | *Glycosaminoglycan degradation* |  |
|  | | *Prephenate dehydrogenase* | | *Amino acid synthesis* |  |
| 420 | | *N-acetylmuramoyl-L-alanine amidase* | | *Glycan Biosynthesis and Metabolism* |  |
|  | | *Beta-D-galactosidase* | | *Glycan structures - degradation;Complex Carbohydrates* |  |
|  | | *Diaminopimelate epimerase* | | *Amino acid synthesis* |  |
| 421 | | *N-acetyl-D-galactosamine-4-sulfate 4-sulfohydrolase* | | *Glycosaminoglycan degradation* |  |
|  | | *Beta-D-glucuronidase* | | *Glycan structures - degradation;Exotic Metabolisms* |  |
|  | | *Prephenate dehydrogenase* | | *Amino acid synthesis* |  |
| 422 | | *Beta-N-acetyl-D-hexosaminide N-acetylhexosaminohydrolase* | | *Glycosaminoglycan degradation* |  |
|  | | *L-glutamine synthase* | | *Amino acid synthesis* |  |
|  | | *Prephenate dehydrogenase* | | *Amino acid synthesis* |  |
| 423 | | *Pyruvate-Formate Lyase* | | *Respiration* |  |
|  | | *3-demethylubiquinone-9 3-methyltransferase* | | *Cofactor Biosynthesis* |  |
|  | | *UDP-N-acetylmuramoyl-L-alanyl-D-glutamatemeso-diaminopimelate ligase* | | *Glycan Biosynthesis and Metabolism* |  |
| 424 | | *Ribokinase* | | *Feeder Pathways to Glycolysis* |  |
|  | | *N-acetylmuramoyl-L-alanine amidase* | | *Glycan Biosynthesis and Metabolism* |  |
|  | | *Diaminopimelate epimerase* | | *Amino acid synthesis* |  |
| 425 | | *UDP-N-acetylglucosamine acyltransferase* | | *Glycan Biosynthesis and Metabolism* |  |
|  | | *Glutamate synthase large and small subunit (NADPH)* | | *Amino acid synthesis* |  |
|  | | *Pyruvate-Formate Lyase* | | *Respiration* |  |
| 426 | | *Cytosine deaminase* | | *Pyrimidine metabolism* |  |
|  | | *Alpha-Glucosidase* | | *Complex Carbohydrates* |  |
|  | | *Prephenate dehydrogenase* | | *Amino acid synthesis* |  |
| 427 | | *Agamintase* | | *Nitrogen Metabolism* |  |
|  | | *3-isopropylmalate dehydrogenase* | | *Amino acid synthesis* |  |
|  | | *Diaminopimelate epimerase* | | *Amino acid synthesis* |  |
| 428 | | *Fucose Isomerase* | | *Feeder Pathways to Glycolysis* |  |
|  | | *IMP dehydrogenase* | | *Purine metabolism* |  |
|  | | *Prephenate dehydrogenase* | | *Amino acid synthesis* |  |
| 429 | | *Cystathionine gamma-synthase* | | *Amino acid synthesis* |  |
|  | | *L-glutamine synthase* | | *Amino acid synthesis* |  |
|  | | *Pyruvate-Formate Lyase* | | *Respiration* |  |
| 430 | | *Prephenate dehydrogenase* | | *Amino acid synthesis* |  |
|  | | *UDP-N-acetylmuramoyl-L-alanineD-glutamate ligase* | | *Glycan Biosynthesis and Metabolism* |  |
|  | | *Xylose Isomerase* | | *Feeder Pathways to Glycolysis* |  |
| 431 | | *Uridine phosphorylase* | | *Pyrimidine metabolism* |  |
|  | | *N-acetyl-D-galactosamine-4-sulfate 4-sulfohydrolase* | | *Glycosaminoglycan degradation* |  |
|  | | *Prephenate dehydrogenase* | | *Amino acid synthesis* |  |
| 432 | | *Pyridoxal Kinase* | | *Cofactor Biosynthesis* |  |
|  | | *Beta-ketoacyl-acyl-carrier-protein synthase III* | | *Fatty Acid Biosynthesis* |  |
|  | | *Asparaginase* | | *Amino acid synthesis* |  |
| 433 | | *Uridine phosphorylase* | | *Pyrimidine metabolism* |  |
|  | | *N-acetyl-D-galactosamine-4-sulfate 4-sulfohydrolase* | | *Glycosaminoglycan degradation* |  |
|  | | *Pyruvate-Formate Lyase* | | *Respiration* |  |
| 434 | | *Prephenate dehydrogenase* | | *Amino acid synthesis* |  |
|  | | *Argininosuccinate lyase* | | *Amino acid transport and metabolism* |  |
|  | | *Beta-N-acetyl-D-hexosaminide N-acetylhexosaminohydrolase* | | *Glycosaminoglycan degradation* |  |
| 435 | | *Ornithine carbamoyltransferase 1* | | *Amino acid transport and metabolism* |  |
|  | | *Prephenate dehydrogenase* | | *Amino acid synthesis* |  |
|  | | *L-threonine aldolase* | | *Amino acid synthesis* |  |
| 436 | | *L-glutamine synthase* | | *Amino acid synthesis* |  |
|  | | *Indole-3-glycerol-phosphate synthase* | | *Amino acid synthesis* |  |
|  | | *Diaminopimelate epimerase* | | *Amino acid synthesis* |  |
| 437 | | *Uridine phosphorylase* | | *Pyrimidine metabolism* |  |
|  | | *L-alanine dehydrogenase* | | *Amino acid synthesis* |  |
|  | | *Prephenate dehydrogenase* | | *Amino acid synthesis* |  |
| 438 | | *Alpha-Glucosidase* | | *Complex Carbohydrates* |  |
|  | | *Prephenate dehydrogenase* | | *Amino acid synthesis* |  |
|  | | *Glutamate synthase large and small subunit (NADPH)* | | *Amino acid synthesis* |  |
| 439 | | *Diaminopimelate epimerase* | | *Amino acid synthesis* |  |
|  | | *Adenylosuccinate synthetase* | | *Purine metabolism* |  |
|  | | *Spermidine Synthase* | | *Nitrogen Metabolism;Amino acid transport and metabolism* |  |
| 440 | | *Arabinose Isomerase* | | *Feeder Pathways to Glycolysis* |  |
|  | | *Argininosuccinate lyase* | | *Amino acid transport and metabolism* |  |
|  | | *N-acetylmuramoyl-L-alanine amidase* | | *Glycan Biosynthesis and Metabolism* |  |
| 441 | | *Pyruvate-Formate Lyase* | | *Respiration* |  |
|  | | *N-Acetylglucosamine-6-Phosphate Deacetylase* | | *Feeder Pathways to Glycolysis* |  |
|  | | *Cytosine deaminase* | | *Pyrimidine metabolism* |  |
| 442 | | *UDP-N-acetylmuramateL-alanine ligase* | | *Glycan Biosynthesis and Metabolism* |  |
|  | | *Diaminopimelate epimerase* | | *Amino acid synthesis* |  |
|  | | *Cytidylate kinase* | | *Pyrimidine metabolism* |  |
| 443 | | *Cytidylate kinase* | | *Pyrimidine metabolism* |  |
|  | | *Prephenate dehydrogenase* | | *Amino acid synthesis* |  |
|  | | *Ribokinase* | | *Feeder Pathways to Glycolysis* |  |
| 444 | | *1-hydroxy-2-methyl-2-E-butenyl 4-diphosphate reductase 4Fe-4S protein* | | *Isoprenoid biosynthesis* |  |
|  | | *Diaminopimelate epimerase* | | *Amino acid synthesis* |  |
|  | | *Folylpolyglutamate Synthase* | | *Cofactor Biosynthesis* |  |
| 445 | | *Pyruvate-Formate Lyase* | | *Respiration* |  |
|  | | *Beta-D-galactosidase* | | *Glycan structures - degradation;Complex Carbohydrates* |  |
|  | | *L-alanine dehydrogenase* | | *Amino acid synthesis* |  |
| 446 | | *Ribokinase* | | *Feeder Pathways to Glycolysis* |  |
|  | | *Pyruvate-Formate Lyase* | | *Respiration* |  |
|  | | *Transketolase* | | *Central Carbon Metabolism Pathways* |  |
| 447 | | *Carbon Monoxide Dehydrogenase* | | *Central Carbon Metabolism Pathways* |  |
|  | | *N-acetylmuramoyl-L-alanine amidase* | | *Glycan Biosynthesis and Metabolism* |  |
|  | | *Folylpolyglutamate Synthase* | | *Cofactor Biosynthesis* |  |
| 448 | | *N-acetylmuramoyl-L-alanine amidase* | | *Glycan Biosynthesis and Metabolism* |  |
|  | | *Phosphoribosylglycinamide synthetase phosphoribosylamine-glycine ligase* | | *Purine metabolism* |  |
|  | | *Glutamate racemase* | | *Amino acid synthesis* |  |
| 449 | | *Ribokinase* | | *Feeder Pathways to Glycolysis* |  |
|  | | *Diaminopimelate epimerase* | | *Amino acid synthesis* |  |
|  | | *Carbamoyl phosphate synthetase small subunit glutamine amidotransferase* | | *Pyrimidine metabolism* |  |
| 450 | | *Beta-N-acetyl-D-hexosaminide N-acetylhexosaminohydrolase* | | *Glycosaminoglycan degradation* |  |
|  | | *N-acetyl-D-glucosamine-6-sulfate 6-sulfohydrolase* | | *Glycosaminoglycan degradation* |  |
|  | | *N-acetylmuramoyl-L-alanine amidase* | | *Glycan Biosynthesis and Metabolism* |  |
| 451 | | *Agamintase* | | *Nitrogen Metabolism* |  |
|  | | *Prephenate dehydrogenase* | | *Amino acid synthesis* |  |
|  | | *Aspartate-ammonia ligase* | | *Amino acid synthesis* |  |
| 452 | | *N-acetyl-D-galactosamine-4-sulfate 4-sulfohydrolase* | | *Glycosaminoglycan degradation* |  |
|  | | *Carbamoyl phosphate synthetase small subunit glutamine amidotransferase* | | *Pyrimidine metabolism* |  |
|  | | *Pyruvate-Formate Lyase* | | *Respiration* |  |
| 453 | | *Diaminopimelate epimerase* | | *Amino acid synthesis* |  |
|  | | *Geranyltranstransferase* | | *Isoprenoid biosynthesis* |  |
|  | | *Glycerol Kinase* | | *Glycerolipid Metabolism* |  |
| 454 | | *Glutamate synthase large and small subunit (NADPH)* | | *Amino acid synthesis* |  |
|  | | *Prephenate dehydrogenase* | | *Amino acid synthesis* |  |
|  | | *Aspartate-ammonia ligase* | | *Amino acid synthesis* |  |
| 455 | | *UDP-N-acetylmuramateL-alanine ligase* | | *Glycan Biosynthesis and Metabolism* |  |
|  | | *Asparaginase* | | *Amino acid synthesis* |  |
|  | | *Pyruvate-Formate Lyase* | | *Respiration* |  |
| 456 | | *L-threonine aldolase* | | *Amino acid synthesis* |  |
|  | | *Diaminopimelate epimerase* | | *Amino acid synthesis* |  |
|  | | *Aspartate-ammonia ligase* | | *Amino acid synthesis* |  |
| 457 | | *Diaminopimelate epimerase* | | *Amino acid synthesis* |  |
|  | | *Geranyltranstransferase* | | *Isoprenoid biosynthesis* |  |
|  | | *Alpha-Glucosidase* | | *Complex Carbohydrates* |  |
| 458 | | *Pyruvate-Formate Lyase* | | *Respiration* |  |
|  | | *4-diphosphocytidyl-2C-methyl-D-erythritol synthase* | | *Isoprenoid biosynthesis* |  |
|  | | *Alpha-Glucosidase* | | *Complex Carbohydrates* |  |
| 459 | | *Formyltetrahydrofolate synthetase* | | *Organic Acids* |  |
|  | | *Pyruvate-Formate Lyase* | | *Respiration* |  |
|  | | *Prephenate dehydrogenase* | | *Amino acid synthesis* |  |
| 460 | | *Ornithine carbamoyltransferase 1* | | *Amino acid transport and metabolism* |  |
|  | | *Pyruvate-Formate Lyase* | | *Respiration* |  |
|  | | *UDP-N-acetylglucosamine acyltransferase* | | *Glycan Biosynthesis and Metabolism* |  |
| 461 | | *N-Acetylglucosamine-6-Phosphate Deacetylase* | | *Feeder Pathways to Glycolysis* |  |
|  | | *Thioredoxin reductase FAD-NADP-binding* | | *Pyrimidine metabolism* |  |
|  | | *N-acetylmuramoyl-L-alanine amidase* | | *Glycan Biosynthesis and Metabolism* |  |
| 462 | | *UDP-N-acetylglucosamine acyltransferase* | | *Glycan Biosynthesis and Metabolism* |  |
|  | | *Pyruvate-Formate Lyase* | | *Respiration* |  |
|  | | *Aspartate-ammonia ligase* | | *Amino acid synthesis* |  |
| 463 | | *Alpha-Glucosidase* | | *Complex Carbohydrates* |  |
|  | | *N-acetylmuramoyl-L-alanine amidase* | | *Glycan Biosynthesis and Metabolism* |  |
|  | | *Alanine racemase* | | *Amino acid synthesis* |  |
| 464 | | *Dihydrodipicolinate synthase* | | *Amino acid synthesis* |  |
|  | | *Glycerol Kinase* | | *Glycerolipid Metabolism* |  |
|  | | *Diaminopimelate epimerase* | | *Amino acid synthesis* |  |
| 465 | | *Cytosine deaminase* | | *Pyrimidine metabolism* |  |
|  | | *Aspartate kinase* | | *Amino acid synthesis* |  |
|  | | *Prephenate dehydrogenase* | | *Amino acid synthesis* |  |
| 466 | | *Gamma-glutamyl kinase* | | *Amino acid transport and metabolism* |  |
|  | | *Pectinase (Pectinesterase)* | | *Complex Carbohydrates* |  |
|  | | *N-acetylmuramoyl-L-alanine amidase* | | *Glycan Biosynthesis and Metabolism* |  |
| 467 | | *Prephenate dehydrogenase* | | *Amino acid synthesis* |  |
|  | | *Folylpolyglutamate Synthase* | | *Cofactor Biosynthesis* |  |
|  | | *Alanine racemase* | | *Amino acid synthesis* |  |
| 468 | | *Cystathionine gamma-synthase* | | *Amino acid synthesis* |  |
|  | | *UDP-N-acetylglucosamine acyltransferase* | | *Glycan Biosynthesis and Metabolism* |  |
|  | | *Pyruvate-Formate Lyase* | | *Respiration* |  |
| 469 | | *IMP dehydrogenase* | | *Purine metabolism* |  |
|  | | *Thioredoxin reductase FAD-NADP-binding* | | *Pyrimidine metabolism* |  |
|  | | *Pyruvate-Formate Lyase* | | *Respiration* |  |
| 470 | | *Homoserine dehydrogenase* | | *Amino acid transport and metabolism* |  |
|  | | *Alanine racemase* | | *Amino acid synthesis* |  |
|  | | *Diaminopimelate epimerase* | | *Amino acid synthesis* |  |
| 471 | | *Carbamoyl phosphate synthetase small subunit glutamine amidotransferase* | | *Pyrimidine metabolism* |  |
|  | | *Pyruvate-Formate Lyase* | | *Respiration* |  |
|  | | *Glutamate synthase large and small subunit (NADPH)* | | *Amino acid synthesis* |  |
| 472 | | *Alanine racemase* | | *Amino acid synthesis* |  |
|  | | *Pyruvate-Formate Lyase* | | *Respiration* |  |
|  | | *UDP-N-acetylmuramateL-alanine ligase* | | *Glycan Biosynthesis and Metabolism* |  |
| 473 | | *Beta-D-glucuronidase* | | *Glycan structures - degradation;Exotic Metabolisms* |  |
|  | | *Prephenate dehydrogenase* | | *Amino acid synthesis* |  |
|  | | *Cytidylate kinase* | | *Pyrimidine metabolism* |  |
| 474 | | *Chorismate synthase* | | *Amino acid transport and metabolism* |  |
|  | | *Beta-ketoacyl-acyl-carrier-protein synthase III* | | *Fatty Acid Biosynthesis* |  |
|  | | *N-acetylmuramoyl-L-alanine amidase* | | *Glycan Biosynthesis and Metabolism* |  |
| 475 | | *Diaminopimelate epimerase* | | *Amino acid synthesis* |  |
|  | | *3-isopropylmalate dehydrogenase* | | *Amino acid synthesis* |  |
|  | | *Dihydrodipicolinate reductase* | | *Amino acid transport and metabolism* |  |
| 476 | | *Pyruvate-Formate Lyase* | | *Respiration* |  |
|  | | *Alanine racemase* | | *Amino acid synthesis* |  |
|  | | *Dihydrodipicolinate synthase* | | *Amino acid synthesis* |  |
| 477 | | *Pyruvate-Formate Lyase* | | *Respiration* |  |
|  | | *Glycerol Kinase* | | *Glycerolipid Metabolism* |  |
|  | | *Aspartate-ammonia ligase* | | *Amino acid synthesis* |  |
| 478 | | *IMP dehydrogenase* | | *Purine metabolism* |  |
|  | | *Dihydrodipicolinate reductase* | | *Amino acid transport and metabolism* |  |
|  | | *Pyruvate-Formate Lyase* | | *Respiration* |  |
| 479 | | *Beta-D-galactosidase* | | *Glycan structures - degradation;Complex Carbohydrates* |  |
|  | | *Diaminopimelate epimerase* | | *Amino acid synthesis* |  |
|  | | *UDP-N-acetylmuramoyl-L-alanyl-D-glutamatemeso-diaminopimelate ligase* | | *Glycan Biosynthesis and Metabolism* |  |
| 480 | | *Glycerol Kinase* | | *Glycerolipid Metabolism* |  |
|  | | *Diaminopimelate epimerase* | | *Amino acid synthesis* |  |
|  | | *1-hydroxy-2-methyl-2-E-butenyl 4-diphosphate reductase 4Fe-4S protein* | | *Isoprenoid biosynthesis* |  |
| 481 | | *Dihydrodipicolinate synthase* | | *Amino acid synthesis* |  |
|  | | *Pyruvate-Formate Lyase* | | *Respiration* |  |
|  | | *Beta-N-acetyl-D-hexosaminide N-acetylhexosaminohydrolase* | | *Glycosaminoglycan degradation* |  |
| 482 | | *N-Acetylglucosamine-6-Phosphate Deacetylase* | | *Feeder Pathways to Glycolysis* |  |
|  | | *IMP dehydrogenase* | | *Purine metabolism* |  |
|  | | *Prephenate dehydrogenase* | | *Amino acid synthesis* |  |
| 483 | | *Xylose Isomerase* | | *Feeder Pathways to Glycolysis* |  |
|  | | *Pyruvate-Formate Lyase* | | *Respiration* |  |
|  | | *Ornithine carbamoyltransferase 1* | | *Amino acid transport and metabolism* |  |
| 484 | | *L-glutamine synthase* | | *Amino acid synthesis* |  |
|  | | *Butyrate Kinase* | | *Organic Acids* |  |
|  | | *Prephenate dehydrogenase* | | *Amino acid synthesis* |  |
| 485 | | *Diaminopimelate epimerase* | | *Amino acid synthesis* |  |
|  | | *Pyridoxal Kinase* | | *Cofactor Biosynthesis* |  |
|  | | *Beta-D-galactosidase* | | *Glycan structures - degradation;Complex Carbohydrates* |  |
| 486 | | *Xylose Isomerase* | | *Feeder Pathways to Glycolysis* |  |
|  | | *Pyruvate-Formate Lyase* | | *Respiration* |  |
|  | | *Dihydrodipicolinate synthase* | | *Amino acid synthesis* |  |
| 487 | | *Butyrate Kinase* | | *Organic Acids* |  |
|  | | *UDP-N-acetylmuramateL-alanine ligase* | | *Glycan Biosynthesis and Metabolism* |  |
|  | | *N-acetylmuramoyl-L-alanine amidase* | | *Glycan Biosynthesis and Metabolism* |  |
| 488 | | *N-acetylmuramoyl-L-alanine amidase* | | *Glycan Biosynthesis and Metabolism* |  |
|  | | *Phosphoribosylglycinamide synthetase phosphoribosylamine-glycine ligase* | | *Purine metabolism* |  |
|  | | *N-Acetylglucosamine-6-Phosphate Deacetylase* | | *Feeder Pathways to Glycolysis* |  |
| 489 | | *Cysteine synthase A* | | *Amino acid synthesis* |  |
|  | | *Prephenate dehydrogenase* | | *Amino acid synthesis* |  |
|  | | *Acetylglutamate kinase* | | *Amino acid transport and metabolism* |  |
| 490 | | *Alpha-Glucosidase* | | *Complex Carbohydrates* |  |
|  | | *Prephenate dehydrogenase* | | *Amino acid synthesis* |  |
|  | | *3-deoxy-7-phosphoheptulonate synthase* | | *Amino acid transport and metabolism* |  |
| 491 | | *Prephenate dehydrogenase* | | *Amino acid synthesis* |  |
|  | | *Cytidylate kinase* | | *Pyrimidine metabolism* |  |
|  | | *Spermidine Synthase* | | *Nitrogen Metabolism;Amino acid transport and metabolism* |  |
| 492 | | *UDP-N-acetylmuramateL-alanine ligase* | | *Glycan Biosynthesis and Metabolism* |  |
|  | | *Pyruvate-Formate Lyase* | | *Respiration* |  |
|  | | *ADP-ribose pyrophosphatase* | | *Purine metabolism* |  |
| 493 | | *Prephenate dehydrogenase* | | *Amino acid synthesis* |  |
|  | | *Folylpolyglutamate Synthase* | | *Cofactor Biosynthesis* |  |
|  | | *Dihydroxy-acid dehydratase* | | *Amino acid transport and metabolism* |  |
| 494 | | *Biosynthetic arginine decarboxylase PLP-binding* | | *Amino acid transport and metabolism* |  |
|  | | *Prephenate dehydrogenase* | | *Amino acid synthesis* |  |
|  | | *Uridine phosphorylase* | | *Pyrimidine metabolism* |  |
| 495 | | *Cysteine synthase A* | | *Amino acid synthesis* |  |
|  | | *Adenylosuccinate synthetase* | | *Purine metabolism* |  |
|  | | *Prephenate dehydrogenase* | | *Amino acid synthesis* |  |
| 496 | | *Cytidylate kinase* | | *Pyrimidine metabolism* |  |
|  | | *Dihydrodipicolinate synthase* | | *Amino acid synthesis* |  |
|  | | *Prephenate dehydrogenase* | | *Amino acid synthesis* |  |
| 497 | | *UDP-N-acetylmuramateL-alanine ligase* | | *Glycan Biosynthesis and Metabolism* |  |
|  | | *N-acetylmuramoyl-L-alanine amidase* | | *Glycan Biosynthesis and Metabolism* |  |
|  | | *3-deoxy-7-phosphoheptulonate synthase* | | *Amino acid transport and metabolism* |  |
| 498 | | *Selenocysteine synthase* | | *Amino acid synthesis;Amino acid synthesis* |  |
|  | | *Ornithine carbamoyltransferase 1* | | *Amino acid transport and metabolism* |  |
|  | | *N-acetylmuramoyl-L-alanine amidase* | | *Glycan Biosynthesis and Metabolism* |  |
| 499 | | *Asparaginase* | | *Amino acid synthesis* |  |
|  | | *N-acetylmuramoyl-L-alanine amidase* | | *Glycan Biosynthesis and Metabolism* |  |
|  | | *L-Lactate Dehydrogenase* | | *Organic Acids* |  |
| 500 | | *Thioredoxin reductase FAD-NADP-binding* | | *Pyrimidine metabolism* |  |
|  | | *Diaminopimelate epimerase* | | *Amino acid synthesis* |  |
|  | | *Formyltetrahydrofolate synthetase* | | *Organic Acids* |  |
| 501 | | *Beta-D-galactosidase* | | *Glycan structures - degradation;Complex Carbohydrates* |  |
|  | | *Prephenate dehydrogenase* | | *Amino acid synthesis* |  |
|  | | *Quinolinate Synthase* | | *Cofactor Biosynthesis* |  |
| 502 | | *N-acetyl-D-galactosamine-4-sulfate 4-sulfohydrolase* | | *Glycosaminoglycan degradation* |  |
|  | | *Thioredoxin reductase FAD-NADP-binding* | | *Pyrimidine metabolism* |  |
|  | | *N-acetylmuramoyl-L-alanine amidase* | | *Glycan Biosynthesis and Metabolism* |  |
| 503 | | *Serine O-acetyltransferase* | | *Amino acid synthesis* |  |
|  | | *Diaminopimelate epimerase* | | *Amino acid synthesis* |  |
|  | | *Valine-pyruvate aminotransferase* | | *Amino acid synthesis* |  |
| 504 | | *Spermidine Synthase* | | *Nitrogen Metabolism;Amino acid transport and metabolism* |  |
|  | | *Transketolase* | | *Central Carbon Metabolism Pathways* |  |
|  | | *N-acetylmuramoyl-L-alanine amidase* | | *Glycan Biosynthesis and Metabolism* |  |
| 505 | | *Thymidine phosphorylase* | | *Pyrimidine metabolism* |  |
|  | | *N-acetylmuramoyl-L-alanine amidase* | | *Glycan Biosynthesis and Metabolism* |  |
|  | | *Cystathionine gamma-synthase* | | *Amino acid synthesis* |  |
| 506 | | *Prephenate dehydrogenase* | | *Amino acid synthesis* |  |
|  | | *3-demethylubiquinone-9 3-methyltransferase* | | *Cofactor Biosynthesis* |  |
|  | | *Selenocysteine synthase* | | *Amino acid synthesis;Amino acid synthesis* |  |
| 507 | | *Serine-tRNA ligase* | | *Amino acid synthesis* |  |
|  | | *Prephenate dehydrogenase* | | *Amino acid synthesis* |  |
|  | | *Aspartate-ammonia ligase* | | *Amino acid synthesis* |  |
| 508 | | *Glutamate racemase* | | *Amino acid synthesis* |  |
|  | | *Dihydrodipicolinate synthase* | | *Amino acid synthesis* |  |
|  | | *Prephenate dehydrogenase* | | *Amino acid synthesis* |  |
| 509 | | *Diaminopimelate epimerase* | | *Amino acid synthesis* |  |
|  | | *Transketolase* | | *Central Carbon Metabolism Pathways* |  |
|  | | *Carbon Monoxide Dehydrogenase* | | *Central Carbon Metabolism Pathways* |  |
| 510 | | *Prephenate dehydrogenase* | | *Amino acid synthesis* |  |
|  | | *UDP-N-acetylglucosamine acyltransferase* | | *Glycan Biosynthesis and Metabolism* |  |
|  | | *Ribokinase* | | *Feeder Pathways to Glycolysis* |  |
| 511 | | *Aspartate-ammonia ligase* | | *Amino acid synthesis* |  |
|  | | *L-glutamine synthase* | | *Amino acid synthesis* |  |
|  | | *Prephenate dehydrogenase* | | *Amino acid synthesis* |  |
| 512 | | *Asparaginase* | | *Amino acid synthesis* |  |
|  | | *N-acetylmuramoyl-L-alanine amidase* | | *Glycan Biosynthesis and Metabolism* |  |
|  | | *UDP-N-acetylmuramateL-alanine ligase* | | *Glycan Biosynthesis and Metabolism* |  |
| 513 | | *Aspartate kinase* | | *Amino acid synthesis* |  |
|  | | *N-acetylmuramoyl-L-alanine amidase* | | *Glycan Biosynthesis and Metabolism* |  |
|  | | *Mannose-6-Phosphate Isomerase* | | *Feeder Pathways to Glycolysis* |  |
| 514 | | *Diaminopimelate epimerase* | | *Amino acid synthesis* |  |
|  | | *IMP dehydrogenase* | | *Purine metabolism* |  |
|  | | *Butyrate Kinase* | | *Organic Acids* |  |
| 515 | | *Diaminopimelate epimerase* | | *Amino acid synthesis* |  |
|  | | *3-isopropylmalate dehydrogenase* | | *Amino acid synthesis* |  |
|  | | *Formyltetrahydrofolate synthetase* | | *Organic Acids* |  |
| 516 | | *Gamma-glutamyl kinase* | | *Amino acid transport and metabolism* |  |
|  | | *Homoserine dehydrogenase* | | *Amino acid transport and metabolism* |  |
|  | | *Prephenate dehydrogenase* | | *Amino acid synthesis* |  |
| 517 | | *Pyruvate-Formate Lyase* | | *Respiration* |  |
|  | | *Ornithine carbamoyltransferase 1* | | *Amino acid transport and metabolism* |  |
|  | | *N-acetylmuramoyl-L-alanine amidase* | | *Glycan Biosynthesis and Metabolism* |  |
| 518 | | *Glycogen Synthase* | | *Complex Carbohydrates* |  |
|  | | *Diaminopimelate epimerase* | | *Amino acid synthesis* |  |
|  | | *UDP-N-acetylmuramateL-alanine ligase* | | *Glycan Biosynthesis and Metabolism* |  |
| 519 | | *Beta-D-glucuronidase* | | *Glycan structures - degradation;Exotic Metabolisms* |  |
|  | | *UDP-N-acetylmuramoyl-L-alanineD-glutamate ligase* | | *Glycan Biosynthesis and Metabolism* |  |
|  | | *Pyruvate-Formate Lyase* | | *Respiration* |  |
| 520 | | *Thioredoxin reductase FAD-NADP-binding* | | *Pyrimidine metabolism* |  |
|  | | *Diaminopimelate epimerase* | | *Amino acid synthesis* |  |
|  | | *Uridine phosphorylase* | | *Pyrimidine metabolism* |  |
| 521 | | *Alpha-Glucosidase* | | *Complex Carbohydrates* |  |
|  | | *UDP-N-acetylmuramoyl-L-alanyl-D-glutamatemeso-diaminopimelate ligase* | | *Glycan Biosynthesis and Metabolism* |  |
|  | | *Diaminopimelate epimerase* | | *Amino acid synthesis* |  |
| 522 | | *Cystathionine gamma-synthase* | | *Amino acid synthesis* |  |
|  | | *Arabinose Isomerase* | | *Feeder Pathways to Glycolysis* |  |
|  | | *Diaminopimelate epimerase* | | *Amino acid synthesis* |  |
| 523 | | *Prephenate dehydrogenase* | | *Amino acid synthesis* |  |
|  | | *Cystathionine gamma-synthase* | | *Amino acid synthesis* |  |
|  | | *Cytidylate kinase* | | *Pyrimidine metabolism* |  |
| 524 | | *Pyruvate-Formate Lyase* | | *Respiration* |  |
|  | | *Dihydrodipicolinate synthase* | | *Amino acid synthesis* |  |
|  | | *Thioredoxin reductase FAD-NADP-binding* | | *Pyrimidine metabolism* |  |
| 525 | | *N-Acetylglucosamine-6-Phosphate Deacetylase* | | *Feeder Pathways to Glycolysis* |  |
|  | | *Diaminopimelate epimerase* | | *Amino acid synthesis* |  |
|  | | *Glutamate racemase* | | *Amino acid synthesis* |  |
| 526 | | *UDP-N-acetylmuramateL-alanine ligase* | | *Glycan Biosynthesis and Metabolism* |  |
|  | | *UDP-N-acetylglucosamine acyltransferase* | | *Glycan Biosynthesis and Metabolism* |  |
|  | | *Pyruvate-Formate Lyase* | | *Respiration* |  |
| 527 | | *Pyruvate-Formate Lyase* | | *Respiration* |  |
|  | | *Cytosine deaminase* | | *Pyrimidine metabolism* |  |
|  | | *Cytidylate kinase* | | *Pyrimidine metabolism* |  |
| 528 | | *Carbon Monoxide Dehydrogenase* | | *Central Carbon Metabolism Pathways* |  |
|  | | *Biotin Synthase* | | *Cofactor Biosynthesis* |  |
|  | | *N-acetylmuramoyl-L-alanine amidase* | | *Glycan Biosynthesis and Metabolism* |  |
| 529 | | *Prephenate dehydrogenase* | | *Amino acid synthesis* |  |
|  | | *Cobalt Chelatase* | | *Cofactor Biosynthesis* |  |
|  | | *Aspartate kinase* | | *Amino acid synthesis* |  |
| 530 | | *Diaminopimelate epimerase* | | *Amino acid synthesis* |  |
|  | | *Alpha-mannosidase* | | *N-Glycan degradation* |  |
|  | | *Glutamate synthase large and small subunit (NADPH)* | | *Amino acid synthesis* |  |
| 531 | | *Cystathionine gamma-synthase* | | *Amino acid synthesis* |  |
|  | | *N-acetyl-D-galactosamine-4-sulfate 4-sulfohydrolase* | | *Glycosaminoglycan degradation* |  |
|  | | *Diaminopimelate epimerase* | | *Amino acid synthesis* |  |
| 532 | | *Thioredoxin reductase FAD-NADP-binding* | | *Pyrimidine metabolism* |  |
|  | | *N-acetylmuramoyl-L-alanine amidase* | | *Glycan Biosynthesis and Metabolism* |  |
|  | | *Homoserine dehydrogenase* | | *Amino acid transport and metabolism* |  |
| 533 | | *Biotin Synthase* | | *Cofactor Biosynthesis* |  |
|  | | *Diaminopimelate epimerase* | | *Amino acid synthesis* |  |
|  | | *Ornithine carbamoyltransferase 1* | | *Amino acid transport and metabolism* |  |
| 534 | | *Pyruvate-Formate Lyase* | | *Respiration* |  |
|  | | *Alpha-Glucosidase* | | *Complex Carbohydrates* |  |
|  | | *3-deoxy-7-phosphoheptulonate synthase* | | *Amino acid transport and metabolism* |  |
| 535 | | *Prephenate dehydrogenase* | | *Amino acid synthesis* |  |
|  | | *Histidinol dehydrogenase* | | *Amino acid transport and metabolism* |  |
|  | | *Arginosuccinate synthase* | | *Amino acid synthesis* |  |
| 536 | | *Diaminopimelate epimerase* | | *Amino acid synthesis* |  |
|  | | *Formyltetrahydrofolate synthetase* | | *Organic Acids* |  |
|  | | *IMP dehydrogenase* | | *Purine metabolism* |  |
| 537 | | *Diaminopimelate epimerase* | | *Amino acid synthesis* |  |
|  | | *Arginosuccinate synthase* | | *Amino acid synthesis* |  |
|  | | *Pyridoxal Kinase* | | *Cofactor Biosynthesis* |  |
| 538 | | *Cystathionine gamma-synthase* | | *Amino acid synthesis* |  |
|  | | *N-Acetylglucosamine-6-Phosphate Deacetylase* | | *Feeder Pathways to Glycolysis* |  |
|  | | *Pyruvate-Formate Lyase* | | *Respiration* |  |
| 539 | | *Thymidine phosphorylase* | | *Pyrimidine metabolism* |  |
|  | | *Pyruvate-Formate Lyase* | | *Respiration* |  |
|  | | *Ribokinase* | | *Feeder Pathways to Glycolysis* |  |
| 540 | | *Glutamate synthase large and small subunit (NADPH)* | | *Amino acid synthesis* |  |
|  | | *Glycerol Kinase* | | *Glycerolipid Metabolism* |  |
|  | | *Prephenate dehydrogenase* | | *Amino acid synthesis* |  |
| 541 | | *3-deoxy-7-phosphoheptulonate synthase* | | *Amino acid transport and metabolism* |  |
|  | | *L-glutamine synthase* | | *Amino acid synthesis* |  |
|  | | *Prephenate dehydrogenase* | | *Amino acid synthesis* |  |
| 542 | | *Diaminopimelate epimerase* | | *Amino acid synthesis* |  |
|  | | *UDP-N-acetylmuramateL-alanine ligase* | | *Glycan Biosynthesis and Metabolism* |  |
|  | | *Pantothenate Synthetase* | | *Cofactor Biosynthesis* |  |
| 543 | | *Diaminopimelate epimerase* | | *Amino acid synthesis* |  |
|  | | *Glycerol Kinase* | | *Glycerolipid Metabolism* |  |
|  | | *Cytosine deaminase* | | *Pyrimidine metabolism* |  |
| 544 | | *Diaminopimelate epimerase* | | *Amino acid synthesis* |  |
|  | | *Arginosuccinate synthase* | | *Amino acid synthesis* |  |
|  | | *Ribokinase* | | *Feeder Pathways to Glycolysis* |  |
| 545 | | *Cytosine deaminase* | | *Pyrimidine metabolism* |  |
|  | | *Pyruvate-Formate Lyase* | | *Respiration* |  |
|  | | *UDP-N-acetylmuramateL-alanine ligase* | | *Glycan Biosynthesis and Metabolism* |  |
| 546 | | *D-alanineD-alanine ligase* | | *Glycan Biosynthesis and Metabolism* |  |
|  | | *Threonine ammonia-lyase* | | *Amino acid synthesis* |  |
|  | | *N-acetylmuramoyl-L-alanine amidase* | | *Glycan Biosynthesis and Metabolism* |  |
| 547 | | *Aspartate-ammonia ligase* | | *Amino acid synthesis* |  |
|  | | *N-acetylmuramoyl-L-alanine amidase* | | *Glycan Biosynthesis and Metabolism* |  |
|  | | *Beta-D-galactosidase* | | *Glycan structures - degradation;Complex Carbohydrates* |  |
| 548 | | *Diaminopimelate epimerase* | | *Amino acid synthesis* |  |
|  | | *Cystathionine gamma-synthase* | | *Amino acid synthesis* |  |
|  | | *Asparaginase* | | *Amino acid synthesis* |  |
| 549 | | *Beta-D-galactosidase* | | *Glycan structures - degradation;Complex Carbohydrates* |  |
|  | | *Diaminopimelate epimerase* | | *Amino acid synthesis* |  |
|  | | *Threonine ammonia-lyase* | | *Amino acid synthesis* |  |
| 550 | | *Geranyltranstransferase* | | *Isoprenoid biosynthesis* |  |
|  | | *Pyruvate-Formate Lyase* | | *Respiration* |  |
|  | | *Pyridoxal Kinase* | | *Cofactor Biosynthesis* |  |
| 551 | | *N-acetylmuramoyl-L-alanine amidase* | | *Glycan Biosynthesis and Metabolism* |  |
|  | | *Thioredoxin reductase FAD-NADP-binding* | | *Pyrimidine metabolism* |  |
|  | | *L-threonine aldolase* | | *Amino acid synthesis* |  |
| 552 | | *N-acetylmuramoyl-L-alanine amidase* | | *Glycan Biosynthesis and Metabolism* |  |
|  | | *L-threonine aldolase* | | *Amino acid synthesis* |  |
|  | | *Cytidylate kinase* | | *Pyrimidine metabolism* |  |
| 553 | | *Biotin Synthase* | | *Cofactor Biosynthesis* |  |
|  | | *Diaminopimelate epimerase* | | *Amino acid synthesis* |  |
|  | | *Gamma-glutamyl kinase* | | *Amino acid transport and metabolism* |  |
| 554 | | *Alanine racemase* | | *Amino acid synthesis* |  |
|  | | *IMP dehydrogenase* | | *Purine metabolism* |  |
|  | | *Prephenate dehydrogenase* | | *Amino acid synthesis* |  |
| 555 | | *Prephenate dehydrogenase* | | *Amino acid synthesis* |  |
|  | | *Quinolinate Synthase* | | *Cofactor Biosynthesis* |  |
|  | | *Acetylglutamate kinase* | | *Amino acid transport and metabolism* |  |
| 556 | | *Alpha-Glucosidase* | | *Complex Carbohydrates* |  |
|  | | *Alpha-Glucosidase* | | *Complex Carbohydrates* |  |
|  | | *Pyruvate-Formate Lyase* | | *Respiration* |  |
| 557 | | *UDP-N-acetylglucosamine acyltransferase* | | *Glycan Biosynthesis and Metabolism* |  |
|  | | *Carbamoyl phosphate synthetase small subunit glutamine amidotransferase* | | *Pyrimidine metabolism* |  |
|  | | *Pyruvate-Formate Lyase* | | *Respiration* |  |
| 558 | | *Diaminopimelate epimerase* | | *Amino acid synthesis* |  |
|  | | *Beta-N-acetyl-D-hexosaminide N-acetylhexosaminohydrolase* | | *Glycosaminoglycan degradation* |  |
|  | | *Galactokinase* | | *Feeder Pathways to Glycolysis* |  |
| 559 | | *Pyruvate-Formate Lyase* | | *Respiration* |  |
|  | | *Argininosuccinate lyase* | | *Amino acid transport and metabolism* |  |
|  | | *N-acetyl-D-galactosamine-4-sulfate 4-sulfohydrolase* | | *Glycosaminoglycan degradation* |  |
| 560 | | *Butyrate Kinase* | | *Organic Acids* |  |
|  | | *Beta-N-acetyl-D-hexosaminide N-acetylhexosaminohydrolase* | | *Glycosaminoglycan degradation* |  |
|  | | *Diaminopimelate epimerase* | | *Amino acid synthesis* |  |
| 561 | | *Beta-D-galactosidase* | | *Glycan structures - degradation;Complex Carbohydrates* |  |
|  | | *N-Acetylglucosamine-6-Phosphate Deacetylase* | | *Feeder Pathways to Glycolysis* |  |
|  | | *Diaminopimelate epimerase* | | *Amino acid synthesis* |  |
| 562 | | *Pyruvate-Formate Lyase* | | *Respiration* |  |
|  | | *UDP-N-acetylmuramateL-alanine ligase* | | *Glycan Biosynthesis and Metabolism* |  |
|  | | *Carbamoyl phosphate synthetase small subunit glutamine amidotransferase* | | *Pyrimidine metabolism* |  |
| 563 | | *4-diphosphocytidyl-2C-methyl-D-erythritol synthase* | | *Isoprenoid biosynthesis* |  |
|  | | *Methionyl-tRNA synthetase* | | *Amino acid synthesis* |  |
|  | | *N-acetylmuramoyl-L-alanine amidase* | | *Glycan Biosynthesis and Metabolism* |  |
| 564 | | *Beta-ketoacyl-acyl-carrier-protein synthase III* | | *Fatty Acid Biosynthesis* |  |
|  | | *Dihydrodipicolinate reductase* | | *Amino acid transport and metabolism* |  |
|  | | *Diaminopimelate epimerase* | | *Amino acid synthesis* |  |
| 565 | | *Cysteine synthase A* | | *Amino acid synthesis* |  |
|  | | *Pyruvate-Formate Lyase* | | *Respiration* |  |
|  | | *Anthranilate synthase* | | *Amino acid transport and metabolism* |  |
| 566 | | *Cytosine deaminase* | | *Pyrimidine metabolism* |  |
|  | | *N-acetylmuramoyl-L-alanine amidase* | | *Glycan Biosynthesis and Metabolism* |  |
|  | | *Alpha-Glucosidase* | | *Complex Carbohydrates* |  |
| 567 | | *D-alanineD-alanine ligase* | | *Glycan Biosynthesis and Metabolism* |  |
|  | | *Beta-D-galactosidase* | | *Glycan structures - degradation;Complex Carbohydrates* |  |
|  | | *N-acetylmuramoyl-L-alanine amidase* | | *Glycan Biosynthesis and Metabolism* |  |
| 568 | | *Diaminopimelate epimerase* | | *Amino acid synthesis* |  |
|  | | *Folylpolyglutamate Synthase* | | *Cofactor Biosynthesis* |  |
|  | | *Dihydroxy-acid dehydratase* | | *Amino acid transport and metabolism* |  |
| 569 | | *IMP dehydrogenase* | | *Purine metabolism* |  |
|  | | *Folylpolyglutamate Synthase* | | *Cofactor Biosynthesis* |  |
|  | | *N-acetylmuramoyl-L-alanine amidase* | | *Glycan Biosynthesis and Metabolism* |  |
| 570 | | *3-dehydroquinate dehydratase* | | *Amino acid transport and metabolism* |  |
|  | | *Pyruvate-Formate Lyase* | | *Respiration* |  |
|  | | *1-hydroxy-2-methyl-2-E-butenyl 4-diphosphate reductase 4Fe-4S protein* | | *Isoprenoid biosynthesis* |  |
| 571 | | *Threonine ammonia-lyase* | | *Amino acid synthesis* |  |
|  | | *Prephenate dehydrogenase* | | *Amino acid synthesis* |  |
|  | | *UDP-N-acetylmuramoyl-L-alanyl-D-glutamatemeso-diaminopimelate ligase* | | *Glycan Biosynthesis and Metabolism* |  |
| 572 | | *N-Acetylglucosamine-6-Phosphate Deacetylase* | | *Feeder Pathways to Glycolysis* |  |
|  | | *N-acetylmuramoyl-L-alanine amidase* | | *Glycan Biosynthesis and Metabolism* |  |
|  | | *N-Acetylglucosamine-6-Phosphate Deacetylase* | | *Feeder Pathways to Glycolysis* |  |
| 573 | | *Butyrate Kinase* | | *Organic Acids* |  |
|  | | *N-acetylmuramoyl-L-alanine amidase* | | *Glycan Biosynthesis and Metabolism* |  |
|  | | *L-glutamine synthase* | | *Amino acid synthesis* |  |
| 574 | | *Prephenate dehydrogenase* | | *Amino acid synthesis* |  |
|  | | *Alanine racemase* | | *Amino acid synthesis* |  |
|  | | *Cytidylate kinase* | | *Pyrimidine metabolism* |  |
| 575 | | *Chorismate synthase* | | *Amino acid transport and metabolism* |  |
|  | | *Cystathionine gamma-synthase* | | *Amino acid synthesis* |  |
|  | | *Pyruvate-Formate Lyase* | | *Respiration* |  |
| 576 | | *Threonine ammonia-lyase* | | *Amino acid synthesis* |  |
|  | | *Prephenate dehydrogenase* | | *Amino acid synthesis* |  |
|  | | *Conjugated Bile Salt Hydrolase* | | *Exotic Metabolisms* |  |
| 577 | | *Acetate Kinase* | | *Organic Acids* |  |
|  | | *Glycerol Kinase* | | *Glycerolipid Metabolism* |  |
|  | | *Diaminopimelate epimerase* | | *Amino acid synthesis* |  |
| 578 | | *Ribokinase* | | *Feeder Pathways to Glycolysis* |  |
|  | | *Diaminopimelate epimerase* | | *Amino acid synthesis* |  |
|  | | *Pyridoxal Kinase* | | *Cofactor Biosynthesis* |  |
| 579 | | *N-acetylmuramoyl-L-alanine amidase* | | *Glycan Biosynthesis and Metabolism* |  |
|  | | *Homoserine kinase* | | *Amino acid transport and metabolism* |  |
|  | | *Butyrate Kinase* | | *Organic Acids* |  |
| 580 | | *N-acetyl-D-galactosamine-4-sulfate 4-sulfohydrolase* | | *Glycosaminoglycan degradation* |  |
|  | | *N-acetylmuramoyl-L-alanine amidase* | | *Glycan Biosynthesis and Metabolism* |  |
|  | | *IMP dehydrogenase* | | *Purine metabolism* |  |
| 581 | | *Diaminopimelate epimerase* | | *Amino acid synthesis* |  |
|  | | *Cytosine deaminase* | | *Pyrimidine metabolism* |  |
|  | | *Pyridoxal Kinase* | | *Cofactor Biosynthesis* |  |
| 582 | | *N-acetylmuramoyl-L-alanine amidase* | | *Glycan Biosynthesis and Metabolism* |  |
|  | | *Alpha-mannosidase* | | *N-Glycan degradation* |  |
|  | | *Histidinol dehydrogenase* | | *Amino acid transport and metabolism* |  |
| 583 | | *Pyruvate-Formate Lyase* | | *Respiration* |  |
|  | | *Xylose Isomerase* | | *Feeder Pathways to Glycolysis* |  |
|  | | *Ornithine carbamoyltransferase 1* | | *Amino acid transport and metabolism* |  |
| 584 | | *Diaminopimelate epimerase* | | *Amino acid synthesis* |  |
|  | | *L-glutamine synthase* | | *Amino acid synthesis* |  |
|  | | *Cystathionine gamma-synthase* | | *Amino acid synthesis* |  |
| 585 | | *Purine-nucleoside phosphorylase* | | *Purine metabolism* |  |
|  | | *Spermidine Synthase* | | *Nitrogen Metabolism;Amino acid transport and metabolism* |  |
|  | | *Prephenate dehydrogenase* | | *Amino acid synthesis* |  |
| 586 | | *Orotidine-5-phosphate decarboxylase* | | *Pyrimidine metabolism* |  |
|  | | *N-acetylmuramoyl-L-alanine amidase* | | *Glycan Biosynthesis and Metabolism* |  |
|  | | *Thymidine phosphorylase* | | *Pyrimidine metabolism* |  |
| 587 | | *4-diphosphocytidyl-2C-methyl-D-erythritol synthase* | | *Isoprenoid biosynthesis* |  |
|  | | *Diaminopimelate epimerase* | | *Amino acid synthesis* |  |
|  | | *Alpha-Glucosidase* | | *Complex Carbohydrates* |  |
| 588 | | *Purine-nucleoside phosphorylase* | | *Purine metabolism* |  |
|  | | *Cystathionine gamma-synthase* | | *Amino acid synthesis* |  |
|  | | *Diaminopimelate epimerase* | | *Amino acid synthesis* |  |
| 589 | | *Prephenate dehydrogenase* | | *Amino acid synthesis* |  |
|  | | *Thymidine phosphorylase* | | *Pyrimidine metabolism* |  |
|  | | *Serine O-acetyltransferase* | | *Amino acid synthesis* |  |
| 590 | | *Beta-ketoacyl-acyl-carrier-protein synthase III* | | *Fatty Acid Biosynthesis* |  |
|  | | *N-acetylmuramoyl-L-alanine amidase* | | *Glycan Biosynthesis and Metabolism* |  |
|  | | *Beta-D-galactosidase* | | *Glycan structures - degradation;Complex Carbohydrates* |  |
| 591 | | *Pyridoxal Kinase* | | *Cofactor Biosynthesis* |  |
|  | | *Diaminopimelate epimerase* | | *Amino acid synthesis* |  |
|  | | *Aspartate-ammonia ligase* | | *Amino acid synthesis* |  |
| 592 | | *Beta-D-glucuronidase* | | *Glycan structures - degradation;Exotic Metabolisms* |  |
|  | | *Prephenate dehydrogenase* | | *Amino acid synthesis* |  |
|  | | *Folylpolyglutamate Synthase* | | *Cofactor Biosynthesis* |  |
| 593 | | *3-deoxy-7-phosphoheptulonate synthase* | | *Amino acid transport and metabolism* |  |
|  | | *N-acetylmuramoyl-L-alanine amidase* | | *Glycan Biosynthesis and Metabolism* |  |
|  | | *Cytosine deaminase* | | *Pyrimidine metabolism* |  |
| 594 | | *Gamma-glutamyl kinase* | | *Amino acid transport and metabolism* |  |
|  | | *Anthranilate synthase* | | *Amino acid transport and metabolism* |  |
|  | | *Diaminopimelate epimerase* | | *Amino acid synthesis* |  |
| 595 | | *Chorismate synthase* | | *Amino acid transport and metabolism* |  |
|  | | *Dihydrodipicolinate reductase* | | *Amino acid transport and metabolism* |  |
|  | | *Diaminopimelate epimerase* | | *Amino acid synthesis* |  |
| 596 | | *Histidinol dehydrogenase* | | *Amino acid transport and metabolism* |  |
|  | | *Prephenate dehydrogenase* | | *Amino acid synthesis* |  |
|  | | *N-acetyl-D-glucosamine-6-sulfate 6-sulfohydrolase* | | *Glycosaminoglycan degradation* |  |
| 597 | | *Alanine racemase* | | *Amino acid synthesis* |  |
|  | | *Prephenate dehydrogenase* | | *Amino acid synthesis* |  |
|  | | *Cysteine synthase A* | | *Amino acid synthesis* |  |
| 598 | | *Homoserine dehydrogenase* | | *Amino acid transport and metabolism* |  |
|  | | *Prephenate dehydrogenase* | | *Amino acid synthesis* |  |
|  | | *Dihydroxy-acid dehydratase* | | *Amino acid transport and metabolism* |  |
| 599 | | *Anthranilate synthase* | | *Amino acid transport and metabolism* |  |
|  | | *Diaminopimelate epimerase* | | *Amino acid synthesis* |  |
|  | | *Acetyl-CoA acyltransferase anaerobic* | | *Fatty Acid Metabolism* |  |
| 600 | | *Pyruvate-Formate Lyase* | | *Respiration* |  |
|  | | *Chorismate synthase* | | *Amino acid transport and metabolism* |  |
|  | | *UDP-N-acetylmuramoyl-L-alanineD-glutamate ligase* | | *Glycan Biosynthesis and Metabolism* |  |
| 601 | | *Si-Citrate Synthase* | | *Central Carbon Metabolism Pathways* |  |
|  | | *Diaminopimelate epimerase* | | *Amino acid synthesis* |  |
|  | | *Conjugated Bile Salt Hydrolase* | | *Exotic Metabolisms* |  |
| 602 | | *Pyruvate-Formate Lyase* | | *Respiration* |  |
|  | | *Dihydroxy-acid dehydratase* | | *Amino acid transport and metabolism* |  |
|  | | *Ferrochetalase* | | *Cofactor Biosynthesis* |  |
| 603 | | *Diaminopimelate epimerase* | | *Amino acid synthesis* |  |
|  | | *Carbamoyl phosphate synthetase small subunit glutamine amidotransferase* | | *Pyrimidine metabolism* |  |
|  | | *Dihydroxy-acid dehydratase* | | *Amino acid transport and metabolism* |  |
| 604 | | *Pyrroline-5-carboxylate reductase* | | *Amino acid transport and metabolism* |  |
|  | | *Diaminopimelate epimerase* | | *Amino acid synthesis* |  |
|  | | *L-glutamine synthase* | | *Amino acid synthesis* |  |
| 605 | | *Pyruvate-Formate Lyase* | | *Respiration* |  |
|  | | *Thioredoxin reductase FAD-NADP-binding* | | *Pyrimidine metabolism* |  |
|  | | *4-diphosphocytidyl-2C-methyl-D-erythritol synthase* | | *Isoprenoid biosynthesis* |  |
| 606 | | *Pyruvate-Formate Lyase* | | *Respiration* |  |
|  | | *N-acetyl-D-galactosamine-4-sulfate 4-sulfohydrolase* | | *Glycosaminoglycan degradation* |  |
|  | | *Transketolase* | | *Central Carbon Metabolism Pathways* |  |
| 607 | | *Prephenate dehydrogenase* | | *Amino acid synthesis* |  |
|  | | *Transketolase* | | *Central Carbon Metabolism Pathways* |  |
|  | | *Ornithine carbamoyltransferase 1* | | *Amino acid transport and metabolism* |  |
| 608 | | *Acetyl-CoA acyltransferase anaerobic* | | *Fatty Acid Metabolism* |  |
|  | | *Valine-pyruvate aminotransferase* | | *Amino acid synthesis* |  |
|  | | *Diaminopimelate epimerase* | | *Amino acid synthesis* |  |
| 609 | | *N-acetylmuramoyl-L-alanine amidase* | | *Glycan Biosynthesis and Metabolism* |  |
|  | | *Acetyl-CoA acyltransferase anaerobic* | | *Fatty Acid Metabolism* |  |
|  | | *1-hydroxy-2-methyl-2-E-butenyl 4-diphosphate reductase 4Fe-4S protein* | | *Isoprenoid biosynthesis* |  |
| 610 | | *Cytosine deaminase* | | *Pyrimidine metabolism* |  |
|  | | *Pyruvate-Formate Lyase* | | *Respiration* |  |
|  | | *UDP-N-acetylmuramateL-alanine ligase* | | *Glycan Biosynthesis and Metabolism* |  |
| 611 | | *N-Acetylglucosamine-6-Phosphate Deacetylase* | | *Feeder Pathways to Glycolysis* |  |
|  | | *Diaminopimelate epimerase* | | *Amino acid synthesis* |  |
|  | | *Cysteine synthase A* | | *Amino acid synthesis* |  |
| 612 | | *Alpha-Glucosidase* | | *Complex Carbohydrates* |  |
|  | | *Acetyl-CoA acyltransferase anaerobic* | | *Fatty Acid Metabolism* |  |
|  | | *Prephenate dehydrogenase* | | *Amino acid synthesis* |  |
| 613 | | *N-acetylmuramoyl-L-alanine amidase* | | *Glycan Biosynthesis and Metabolism* |  |
|  | | *3-deoxy-7-phosphoheptulonate synthase* | | *Amino acid transport and metabolism* |  |
|  | | *Glycerol Kinase* | | *Glycerolipid Metabolism* |  |
| 614 | | *L-glutamine synthase* | | *Amino acid synthesis* |  |
|  | | *N-acetylmuramoyl-L-alanine amidase* | | *Glycan Biosynthesis and Metabolism* |  |
|  | | *Carbon Monoxide Dehydrogenase* | | *Central Carbon Metabolism Pathways* |  |
| 615 | | *Diaminopimelate epimerase* | | *Amino acid synthesis* |  |
|  | | *Cysteine synthase A* | | *Amino acid synthesis* |  |
|  | | *N-acetyl-D-galactosamine-4-sulfate 4-sulfohydrolase* | | *Glycosaminoglycan degradation* |  |
| 616 | | *L-alanine dehydrogenase* | | *Amino acid synthesis* |  |
|  | | *Pyruvate-Formate Lyase* | | *Respiration* |  |
|  | | *Folylpolyglutamate Synthase* | | *Cofactor Biosynthesis* |  |
| 617 | | *Geranyltranstransferase* | | *Isoprenoid biosynthesis* |  |
|  | | *Argininosuccinate lyase* | | *Amino acid transport and metabolism* |  |
|  | | *Prephenate dehydrogenase* | | *Amino acid synthesis* |  |
| 618 | | *1-hydroxy-2-methyl-2-E-butenyl 4-diphosphate reductase 4Fe-4S protein* | | *Isoprenoid biosynthesis* |  |
|  | | *Arginosuccinate synthase* | | *Amino acid synthesis* |  |
|  | | *N-acetylmuramoyl-L-alanine amidase* | | *Glycan Biosynthesis and Metabolism* |  |
| 619 | | *Pyruvate-Formate Lyase* | | *Respiration* |  |
|  | | *Acetyl-CoA acyltransferase anaerobic* | | *Fatty Acid Metabolism* |  |
|  | | *Glutamate synthase large and small subunit (NADPH)* | | *Amino acid synthesis* |  |
| 620 | | *N-Acetylglucosamine-6-Phosphate Deacetylase* | | *Feeder Pathways to Glycolysis* |  |
|  | | *Beta-D-galactosidase* | | *Glycan structures - degradation;Complex Carbohydrates* |  |
|  | | *Diaminopimelate epimerase* | | *Amino acid synthesis* |  |
| 621 | | *Diaminopimelate epimerase* | | *Amino acid synthesis* |  |
|  | | *Cytosine deaminase* | | *Pyrimidine metabolism* |  |
|  | | *Spermidine Synthase* | | *Nitrogen Metabolism;Amino acid transport and metabolism* |  |
| 622 | | *Phosphogluconate dehydratase* | | *Central Carbon Metabolism Pathways* |  |
|  | | *Prephenate dehydrogenase* | | *Amino acid synthesis* |  |
|  | | *Thioredoxin reductase FAD-NADP-binding* | | *Pyrimidine metabolism* |  |
| 623 | | *N-acetylmuramoyl-L-alanine amidase* | | *Glycan Biosynthesis and Metabolism* |  |
|  | | *Beta-N-acetyl-D-hexosaminide N-acetylhexosaminohydrolase* | | *Glycosaminoglycan degradation* |  |
|  | | *Beta-ketoacyl-acyl-carrier-protein synthase III* | | *Fatty Acid Biosynthesis* |  |
| 624 | | *Diaminopimelate epimerase* | | *Amino acid synthesis* |  |
|  | | *Carbamoyl phosphate synthetase small subunit glutamine amidotransferase* | | *Pyrimidine metabolism* |  |
|  | | *3-deoxy-7-phosphoheptulonate synthase* | | *Amino acid transport and metabolism* |  |
| 625 | | *D-alanineD-alanine ligase* | | *Glycan Biosynthesis and Metabolism* |  |
|  | | *Alpha-mannosidase* | | *N-Glycan degradation* |  |
|  | | *Pyruvate-Formate Lyase* | | *Respiration* |  |
| 626 | | *Riboflavin Synthase α Subunit* | | *Cofactor Biosynthesis* |  |
|  | | *Cysteine synthase A* | | *Amino acid synthesis* |  |
|  | | *Diaminopimelate epimerase* | | *Amino acid synthesis* |  |
| 627 | | *Prephenate dehydrogenase* | | *Amino acid synthesis* |  |
|  | | *ADP-L-glycero-D-mannoheptose-6-epimerase NAD(P)-binding* | | *Glycan Biosynthesis and Metabolism* |  |
|  | | *Homoserine dehydrogenase* | | *Amino acid transport and metabolism* |  |
| 628 | | *Folylpolyglutamate Synthase* | | *Cofactor Biosynthesis* |  |
|  | | *Glutamate synthase large and small subunit (NADPH)* | | *Amino acid synthesis* |  |
|  | | *Pyruvate-Formate Lyase* | | *Respiration* |  |
| 629 | | *Prephenate dehydrogenase* | | *Amino acid synthesis* |  |
|  | | *UDP-N-acetylmuramoyl-L-alanyl-D-glutamatemeso-diaminopimelate ligase* | | *Glycan Biosynthesis and Metabolism* |  |
|  | | *Spermidine Synthase* | | *Nitrogen Metabolism;Amino acid transport and metabolism* |  |
| 630 | | *Beta-D-glucuronidase* | | *Glycan structures - degradation;Exotic Metabolisms* |  |
|  | | *N-acetylmuramoyl-L-alanine amidase* | | *Glycan Biosynthesis and Metabolism* |  |
|  | | *Anthranilate synthase* | | *Amino acid transport and metabolism* |  |
| 631 | | *Cystathionine gamma-synthase* | | *Amino acid synthesis* |  |
|  | | *Pyruvate-Formate Lyase* | | *Respiration* |  |
|  | | *Anthranilate synthase* | | *Amino acid transport and metabolism* |  |
| 632 | | *Xylose Isomerase* | | *Feeder Pathways to Glycolysis* |  |
|  | | *Anthranilate synthase* | | *Amino acid transport and metabolism* |  |
|  | | *Pyruvate-Formate Lyase* | | *Respiration* |  |
| 633 | | *Beta-ketoacyl-acyl-carrier-protein synthase III* | | *Fatty Acid Biosynthesis* |  |
|  | | *Pyruvate-Formate Lyase* | | *Respiration* |  |
|  | | *Formyltetrahydrofolate synthetase* | | *Organic Acids* |  |
| 634 | | *Prephenate dehydrogenase* | | *Amino acid synthesis* |  |
|  | | *Methionyl-tRNA synthetase* | | *Amino acid synthesis* |  |
|  | | *Beta-D-galactosidase* | | *Glycan structures - degradation;Complex Carbohydrates* |  |
| 635 | | *Threonine ammonia-lyase* | | *Amino acid synthesis* |  |
|  | | *Prephenate dehydrogenase* | | *Amino acid synthesis* |  |
|  | | *Folylpolyglutamate Synthase* | | *Cofactor Biosynthesis* |  |
| 636 | | *Gamma-glutamyl kinase* | | *Amino acid transport and metabolism* |  |
|  | | *Formyltetrahydrofolate synthetase* | | *Organic Acids* |  |
|  | | *Pyruvate-Formate Lyase* | | *Respiration* |  |
| 637 | | *4-diphosphocytidyl-2C-methyl-D-erythritol synthase* | | *Isoprenoid biosynthesis* |  |
|  | | *Folylpolyglutamate Synthase* | | *Cofactor Biosynthesis* |  |
|  | | *N-acetylmuramoyl-L-alanine amidase* | | *Glycan Biosynthesis and Metabolism* |  |
| 638 | | *Quinolinate Synthase* | | *Cofactor Biosynthesis* |  |
|  | | *Beta-D-galactosidase* | | *Glycan structures - degradation;Complex Carbohydrates* |  |
|  | | *Prephenate dehydrogenase* | | *Amino acid synthesis* |  |
| 639 | | *Prephenate dehydrogenase* | | *Amino acid synthesis* |  |
|  | | *Glucuronate Isomerase* | | *Feeder Pathways to Glycolysis* |  |
|  | | *Homoserine dehydrogenase* | | *Amino acid transport and metabolism* |  |
| 640 | | *Phosphogluconate dehydratase* | | *Central Carbon Metabolism Pathways* |  |
|  | | *Carbon Monoxide Dehydrogenase* | | *Central Carbon Metabolism Pathways* |  |
|  | | *N-acetylmuramoyl-L-alanine amidase* | | *Glycan Biosynthesis and Metabolism* |  |
| 641 | | *Prephenate dehydrogenase* | | *Amino acid synthesis* |  |
|  | | *Adenylosuccinate synthetase* | | *Purine metabolism* |  |
|  | | *Beta-D-galactosidase* | | *Glycan structures - degradation;Complex Carbohydrates* |  |
| 642 | | *Pyruvate-Formate Lyase* | | *Respiration* |  |
|  | | *Cystathionine gamma-synthase* | | *Amino acid synthesis* |  |
|  | | *Fucose Isomerase* | | *Feeder Pathways to Glycolysis* |  |
| 643 | | *Cysteine synthase A* | | *Amino acid synthesis* |  |
|  | | *Methylmalonyl-CaA decarboxylase* | | *Organic Acids* |  |
|  | | *Prephenate dehydrogenase* | | *Amino acid synthesis* |  |
| 644 | | *N-acetylmuramoyl-L-alanine amidase* | | *Glycan Biosynthesis and Metabolism* |  |
|  | | *Gamma-glutamyl kinase* | | *Amino acid transport and metabolism* |  |
|  | | *Anthranilate synthase* | | *Amino acid transport and metabolism* |  |
| 645 | | *N-acetylmuramoyl-L-alanine amidase* | | *Glycan Biosynthesis and Metabolism* |  |
|  | | *Thioredoxin reductase FAD-NADP-binding* | | *Pyrimidine metabolism* |  |
|  | | *Homoserine dehydrogenase* | | *Amino acid transport and metabolism* |  |
| 646 | | *1-hydroxy-2-methyl-2-E-butenyl 4-diphosphate reductase 4Fe-4S protein* | | *Isoprenoid biosynthesis* |  |
|  | | *Prephenate dehydrogenase* | | *Amino acid synthesis* |  |
|  | | *4-diphosphocytidyl-2C-methyl-D-erythritol synthase* | | *Isoprenoid biosynthesis* |  |
| 647 | | *Diaminopimelate epimerase* | | *Amino acid synthesis* |  |
|  | | *UDP-N-acetylmuramateL-alanine ligase* | | *Glycan Biosynthesis and Metabolism* |  |
|  | | *Ornithine carbamoyltransferase 1* | | *Amino acid transport and metabolism* |  |
| 648 | | *Methylmalonyl-CaA decarboxylase* | | *Organic Acids* |  |
|  | | *Aspartate kinase* | | *Amino acid synthesis* |  |
|  | | *Diaminopimelate epimerase* | | *Amino acid synthesis* |  |
| 649 | | *Uridine phosphorylase* | | *Pyrimidine metabolism* |  |
|  | | *N-acetylmuramoyl-L-alanine amidase* | | *Glycan Biosynthesis and Metabolism* |  |
|  | | *UDP-N-acetylglucosamine acyltransferase* | | *Glycan Biosynthesis and Metabolism* |  |
| 650 | | *Thymidine phosphorylase* | | *Pyrimidine metabolism* |  |
|  | | *Prephenate dehydrogenase* | | *Amino acid synthesis* |  |
|  | | *Glutamate racemase* | | *Amino acid synthesis* |  |
| 651 | | *UDP-N-acetylmuramateL-alanine ligase* | | *Glycan Biosynthesis and Metabolism* |  |
|  | | *Transketolase* | | *Central Carbon Metabolism Pathways* |  |
|  | | *Prephenate dehydrogenase* | | *Amino acid synthesis* |  |
| 652 | | *Cysteine synthase A* | | *Amino acid synthesis* |  |
|  | | *Glutamate synthase large and small subunit (NADPH)* | | *Amino acid synthesis* |  |
|  | | *Prephenate dehydrogenase* | | *Amino acid synthesis* |  |
| 653 | | *Beta-D-galactosidase* | | *Glycan structures - degradation;Complex Carbohydrates* |  |
|  | | *Diaminopimelate epimerase* | | *Amino acid synthesis* |  |
|  | | *Glycerol Kinase* | | *Glycerolipid Metabolism* |  |
| 654 | | *Biotin Synthase* | | *Cofactor Biosynthesis* |  |
|  | | *N-acetylmuramoyl-L-alanine amidase* | | *Glycan Biosynthesis and Metabolism* |  |
|  | | *Argininosuccinate lyase* | | *Amino acid transport and metabolism* |  |
| 655 | | *Glycerol Kinase* | | *Glycerolipid Metabolism* |  |
|  | | *Serine O-acetyltransferase* | | *Amino acid synthesis* |  |
|  | | *Prephenate dehydrogenase* | | *Amino acid synthesis* |  |
| 656 | | *Pyruvate-Formate Lyase* | | *Respiration* |  |
|  | | *Arginosuccinate synthase* | | *Amino acid synthesis* |  |
|  | | *Cytosine deaminase* | | *Pyrimidine metabolism* |  |
| 657 | | *Pyruvate-Formate Lyase* | | *Respiration* |  |
|  | | *Cytosine deaminase* | | *Pyrimidine metabolism* |  |
|  | | *Uridine phosphorylase* | | *Pyrimidine metabolism* |  |
| 658 | | *Pyruvate-Formate Lyase* | | *Respiration* |  |
|  | | *UDP-N-acetylmuramateL-alanine ligase* | | *Glycan Biosynthesis and Metabolism* |  |
|  | | *Homoserine dehydrogenase* | | *Amino acid transport and metabolism* |  |
| 659 | | *Pyruvate-Formate Lyase* | | *Respiration* |  |
|  | | *Thymidine phosphorylase* | | *Pyrimidine metabolism* |  |
|  | | *Threonine ammonia-lyase* | | *Amino acid synthesis* |  |
| 660 | | *Beta-N-acetyl-D-hexosaminide N-acetylhexosaminohydrolase* | | *Glycosaminoglycan degradation* |  |
|  | | *Anaerobic ribonucleoside-triphosphate reductase* | | *Pyrimidine metabolism* |  |
|  | | *N-acetylmuramoyl-L-alanine amidase* | | *Glycan Biosynthesis and Metabolism* |  |
| 661 | | *Cytidylate kinase* | | *Pyrimidine metabolism* |  |
|  | | *Histidinol dehydrogenase* | | *Amino acid transport and metabolism* |  |
|  | | *Pyruvate-Formate Lyase* | | *Respiration* |  |
| 662 | | *Pyruvate-Formate Lyase* | | *Respiration* |  |
|  | | *Glutamate synthase large and small subunit (NADPH)* | | *Amino acid synthesis* |  |
|  | | *Cysteine synthase A* | | *Amino acid synthesis* |  |
| 663 | | *Prephenate dehydrogenase* | | *Amino acid synthesis* |  |
|  | | *Beta-ketoacyl-acyl-carrier-protein synthase III* | | *Fatty Acid Biosynthesis* |  |
|  | | *N-acetyl-D-galactosamine-4-sulfate 4-sulfohydrolase* | | *Glycosaminoglycan degradation* |  |
| 664 | | *Beta-D-galactosidase* | | *Glycan structures - degradation;Complex Carbohydrates* |  |
|  | | *Biotin Synthase* | | *Cofactor Biosynthesis* |  |
|  | | *N-acetylmuramoyl-L-alanine amidase* | | *Glycan Biosynthesis and Metabolism* |  |
| 665 | | *Pyruvate-Formate Lyase* | | *Respiration* |  |
|  | | *Glycerol Kinase* | | *Glycerolipid Metabolism* |  |
|  | | *N-Acetylglucosamine-6-Phosphate Deacetylase* | | *Feeder Pathways to Glycolysis* |  |
| 666 | | *Histidinol dehydrogenase* | | *Amino acid transport and metabolism* |  |
|  | | *Alpha-Glucosidase* | | *Complex Carbohydrates* |  |
|  | | *Prephenate dehydrogenase* | | *Amino acid synthesis* |  |
| 667 | | *UDP-N-acetylmuramateL-alanine ligase* | | *Glycan Biosynthesis and Metabolism* |  |
|  | | *N-acetylmuramoyl-L-alanine amidase* | | *Glycan Biosynthesis and Metabolism* |  |
|  | | *Cysteine synthase A* | | *Amino acid synthesis* |  |
| 668 | | *Diaminopimelate epimerase* | | *Amino acid synthesis* |  |
|  | | *Dihydrodipicolinate synthase* | | *Amino acid synthesis* |  |
|  | | *Ribokinase* | | *Feeder Pathways to Glycolysis* |  |
| 669 | | *Diaminopimelate epimerase* | | *Amino acid synthesis* |  |
|  | | *Geranyltranstransferase* | | *Isoprenoid biosynthesis* |  |
|  | | *Glutamate racemase* | | *Amino acid synthesis* |  |
| 670 | | *Diaminopimelate epimerase* | | *Amino acid synthesis* |  |
|  | | *Dihydrodipicolinate synthase* | | *Amino acid synthesis* |  |
|  | | *Acetyl-CoA acyltransferase anaerobic* | | *Fatty Acid Metabolism* |  |
| 671 | | *Ribokinase* | | *Feeder Pathways to Glycolysis* |  |
|  | | *Anaerobic ribonucleoside-triphosphate reductase* | | *Pyrimidine metabolism* |  |
|  | | *N-acetylmuramoyl-L-alanine amidase* | | *Glycan Biosynthesis and Metabolism* |  |
| 672 | | *UDP-N-acetylmuramoyl-L-alanineD-glutamate ligase* | | *Glycan Biosynthesis and Metabolism* |  |
|  | | *Prephenate dehydrogenase* | | *Amino acid synthesis* |  |
|  | | *Adenylosuccinate synthetase* | | *Purine metabolism* |  |
| 673 | | *Prephenate dehydrogenase* | | *Amino acid synthesis* |  |
|  | | *L-alanine dehydrogenase* | | *Amino acid synthesis* |  |
|  | | *Cysteine synthase A* | | *Amino acid synthesis* |  |
| 674 | | *Phosphogluconate dehydratase* | | *Central Carbon Metabolism Pathways* |  |
|  | | *Diaminopimelate epimerase* | | *Amino acid synthesis* |  |
|  | | *Beta-D-galactosidase* | | *Glycan structures - degradation;Complex Carbohydrates* |  |
| 675 | | *Pyridoxal Kinase* | | *Cofactor Biosynthesis* |  |
|  | | *N-acetylmuramoyl-L-alanine amidase* | | *Glycan Biosynthesis and Metabolism* |  |
|  | | *Beta-ketoacyl-acyl-carrier-protein synthase III* | | *Fatty Acid Biosynthesis* |  |
| 676 | | *Pyruvate-Formate Lyase* | | *Respiration* |  |
|  | | *3-deoxy-7-phosphoheptulonate synthase* | | *Amino acid transport and metabolism* |  |
|  | | *Dihydrodipicolinate synthase* | | *Amino acid synthesis* |  |
| 677 | | *3-demethylubiquinone-9 3-methyltransferase* | | *Cofactor Biosynthesis* |  |
|  | | *N-acetylmuramoyl-L-alanine amidase* | | *Glycan Biosynthesis and Metabolism* |  |
|  | | *L-glutamine synthase* | | *Amino acid synthesis* |  |
| 678 | | *Beta-D-galactosidase* | | *Glycan structures - degradation;Complex Carbohydrates* |  |
|  | | *Diaminopimelate epimerase* | | *Amino acid synthesis* |  |
|  | | *Ribokinase* | | *Feeder Pathways to Glycolysis* |  |
| 679 | | *Prephenate dehydrogenase* | | *Amino acid synthesis* |  |
|  | | *Histidinol dehydrogenase* | | *Amino acid transport and metabolism* |  |
|  | | *Conjugated Bile Salt Hydrolase* | | *Exotic Metabolisms* |  |
| 680 | | *Phosphogluconate dehydratase* | | *Central Carbon Metabolism Pathways* |  |
|  | | *Biosynthetic arginine decarboxylase PLP-binding* | | *Amino acid transport and metabolism* |  |
|  | | *N-acetylmuramoyl-L-alanine amidase* | | *Glycan Biosynthesis and Metabolism* |  |
| 681 | | *Ornithine carbamoyltransferase 1* | | *Amino acid transport and metabolism* |  |
|  | | *L-threonine aldolase* | | *Amino acid synthesis* |  |
|  | | *Pyruvate-Formate Lyase* | | *Respiration* |  |
| 682 | | *Glutamate synthase large and small subunit (NADPH)* | | *Amino acid synthesis* |  |
|  | | *Aspartate-ammonia ligase* | | *Amino acid synthesis* |  |
|  | | *Diaminopimelate epimerase* | | *Amino acid synthesis* |  |
| 683 | | *Ornithine carbamoyltransferase 1* | | *Amino acid transport and metabolism* |  |
|  | | *L-threonine aldolase* | | *Amino acid synthesis* |  |
|  | | *Pyruvate-Formate Lyase* | | *Respiration* |  |
| 684 | | *Pyridoxal Kinase* | | *Cofactor Biosynthesis* |  |
|  | | *Cytosine deaminase* | | *Pyrimidine metabolism* |  |
|  | | *Pyruvate-Formate Lyase* | | *Respiration* |  |
| 685 | | *Galactokinase* | | *Feeder Pathways to Glycolysis* |  |
|  | | *Diaminopimelate epimerase* | | *Amino acid synthesis* |  |
|  | | *Alanine racemase* | | *Amino acid synthesis* |  |
| 686 | | *Carbon Monoxide Dehydrogenase* | | *Central Carbon Metabolism Pathways* |  |
|  | | *Homoserine kinase* | | *Amino acid transport and metabolism* |  |
|  | | *N-acetylmuramoyl-L-alanine amidase* | | *Glycan Biosynthesis and Metabolism* |  |
| 687 | | *Pyruvate-Formate Lyase* | | *Respiration* |  |
|  | | *4-diphosphocytidyl-2C-methyl-D-erythritol synthase* | | *Isoprenoid biosynthesis* |  |
|  | | *Beta-N-acetyl-D-hexosaminide N-acetylhexosaminohydrolase* | | *Glycosaminoglycan degradation* |  |
| 688 | | *N-acetylmuramoyl-L-alanine amidase* | | *Glycan Biosynthesis and Metabolism* |  |
|  | | *L-threonine synthase* | | *Amino acid synthesis* |  |
|  | | *L-glutamine synthase* | | *Amino acid synthesis* |  |
| 689 | | *Ornithine carbamoyltransferase 1* | | *Amino acid transport and metabolism* |  |
|  | | *Cytosine deaminase* | | *Pyrimidine metabolism* |  |
|  | | *Prephenate dehydrogenase* | | *Amino acid synthesis* |  |
| 690 | | *Xylose Isomerase* | | *Feeder Pathways to Glycolysis* |  |
|  | | *Glutamate decarboxylase A and B PLP-dependent* | | *Amino acid transport and metabolism* |  |
|  | | *Pyruvate-Formate Lyase* | | *Respiration* |  |
| 691 | | *Threonine ammonia-lyase* | | *Amino acid synthesis* |  |
|  | | *Pyruvate-Formate Lyase* | | *Respiration* |  |
|  | | *Pyruvate-Formate Lyase* | | *Respiration* |  |
| 692 | | *Beta-ketoacyl-acyl-carrier-protein synthase III* | | *Fatty Acid Biosynthesis* |  |
|  | | *Diaminopimelate epimerase* | | *Amino acid synthesis* |  |
|  | | *Acetyl-CoA acyltransferase anaerobic* | | *Fatty Acid Metabolism* |  |
| 693 | | *Cobalamin Synthase* | | *Cofactor Biosynthesis* |  |
|  | | *Diaminopimelate epimerase* | | *Amino acid synthesis* |  |
|  | | *3-isopropylmalate dehydrogenase* | | *Amino acid synthesis* |  |
| 694 | | *Glutamate synthase large and small subunit (NADPH)* | | *Amino acid synthesis* |  |
|  | | *Pyruvate-Formate Lyase* | | *Respiration* |  |
|  | | *Beta-D-glucuronidase* | | *Glycan structures - degradation;Exotic Metabolisms* |  |
| 695 | | *1-Phosphofructokinase* | | *Feeder Pathways to Glycolysis* |  |
|  | | *Alanine racemase* | | *Amino acid synthesis* |  |
|  | | *N-acetylmuramoyl-L-alanine amidase* | | *Glycan Biosynthesis and Metabolism* |  |
| 696 | | *Chorismate synthase* | | *Amino acid transport and metabolism* |  |
|  | | *Beta-N-acetyl-D-hexosaminide N-acetylhexosaminohydrolase* | | *Glycosaminoglycan degradation* |  |
|  | | *Prephenate dehydrogenase* | | *Amino acid synthesis* |  |
| 697 | | *Diaminopimelate epimerase* | | *Amino acid synthesis* |  |
|  | | *Agamintase* | | *Nitrogen Metabolism* |  |
|  | | *IMP dehydrogenase* | | *Purine metabolism* |  |
| 698 | | *Dihydrodipicolinate synthase* | | *Amino acid synthesis* |  |
|  | | *Cytosine deaminase* | | *Pyrimidine metabolism* |  |
|  | | *Diaminopimelate epimerase* | | *Amino acid synthesis* |  |
| 699 | | *Adenylosuccinate synthetase* | | *Purine metabolism* |  |
|  | | *Prephenate dehydrogenase* | | *Amino acid synthesis* |  |
|  | | *UDP-N-acetylmuramateL-alanine ligase* | | *Glycan Biosynthesis and Metabolism* |  |
| 700 | | *Mannose-6-Phosphate Isomerase* | | *Feeder Pathways to Glycolysis* |  |
|  | | *Prephenate dehydrogenase* | | *Amino acid synthesis* |  |
|  | | *Beta-D-galactosidase* | | *Glycan structures - degradation;Complex Carbohydrates* |  |
| 701 | | *Cytidylate kinase* | | *Pyrimidine metabolism* |  |
|  | | *Cytosine deaminase* | | *Pyrimidine metabolism* |  |
|  | | *Diaminopimelate epimerase* | | *Amino acid synthesis* |  |
| 702 | | *Pyridoxal Kinase* | | *Cofactor Biosynthesis* |  |
|  | | *L-alanine dehydrogenase* | | *Amino acid synthesis* |  |
|  | | *Prephenate dehydrogenase* | | *Amino acid synthesis* |  |
| 703 | | *Glycerol Kinase* | | *Glycerolipid Metabolism* |  |
|  | | *UDP-N-acetylmuramateL-alanine ligase* | | *Glycan Biosynthesis and Metabolism* |  |
|  | | *N-acetylmuramoyl-L-alanine amidase* | | *Glycan Biosynthesis and Metabolism* |  |
| 704 | | *Prephenate dehydrogenase* | | *Amino acid synthesis* |  |
|  | | *Ornithine carbamoyltransferase 1* | | *Amino acid transport and metabolism* |  |
|  | | *Threonine ammonia-lyase* | | *Amino acid synthesis* |  |
| 705 | | *L-Lactate Dehydrogenase* | | *Organic Acids* |  |
|  | | *N-acetylmuramoyl-L-alanine amidase* | | *Glycan Biosynthesis and Metabolism* |  |
|  | | *Carbon Monoxide Dehydrogenase* | | *Central Carbon Metabolism Pathways* |  |
| 706 | | *Cystathionine gamma-synthase* | | *Amino acid synthesis* |  |
|  | | *Ornithine carbamoyltransferase 1* | | *Amino acid transport and metabolism* |  |
|  | | *Prephenate dehydrogenase* | | *Amino acid synthesis* |  |
| 707 | | *Biosynthetic arginine decarboxylase PLP-binding* | | *Amino acid transport and metabolism* |  |
|  | | *3-dehydroquinate dehydratase* | | *Amino acid transport and metabolism* |  |
|  | | *N-acetylmuramoyl-L-alanine amidase* | | *Glycan Biosynthesis and Metabolism* |  |
| 708 | | *Spermidine Synthase* | | *Nitrogen Metabolism;Amino acid transport and metabolism* |  |
|  | | *Folylpolyglutamate Synthase* | | *Cofactor Biosynthesis* |  |
|  | | *Prephenate dehydrogenase* | | *Amino acid synthesis* |  |
| 709 | | *Glutamate synthase large and small subunit (NADPH)* | | *Amino acid synthesis* |  |
|  | | *Prephenate dehydrogenase* | | *Amino acid synthesis* |  |
|  | | *L-glutamine synthase* | | *Amino acid synthesis* |  |
| 710 | | *Alpha-Glucosidase* | | *Complex Carbohydrates* |  |
|  | | *Prephenate dehydrogenase* | | *Amino acid synthesis* |  |
|  | | *D-alanineD-alanine ligase* | | *Glycan Biosynthesis and Metabolism* |  |
| 711 | | *Prephenate dehydrogenase* | | *Amino acid synthesis* |  |
|  | | *Folylpolyglutamate Synthase* | | *Cofactor Biosynthesis* |  |
|  | | *L-threonine aldolase* | | *Amino acid synthesis* |  |
| 712 | | *Homoserine dehydrogenase* | | *Amino acid transport and metabolism* |  |
|  | | *Prephenate dehydrogenase* | | *Amino acid synthesis* |  |
|  | | *Cystathionine gamma-synthase* | | *Amino acid synthesis* |  |
| 713 | | *Dihydrodipicolinate synthase* | | *Amino acid synthesis* |  |
|  | | *Prephenate dehydrogenase* | | *Amino acid synthesis* |  |
|  | | *Arginosuccinate synthase* | | *Amino acid synthesis* |  |
| 714 | | *Formyltetrahydrofolate synthetase* | | *Organic Acids* |  |
|  | | *Anthranilate synthase* | | *Amino acid transport and metabolism* |  |
|  | | *Prephenate dehydrogenase* | | *Amino acid synthesis* |  |
| 715 | | *Prephenate dehydrogenase* | | *Amino acid synthesis* |  |
|  | | *N-acetyl-D-galactosamine-4-sulfate 4-sulfohydrolase* | | *Glycosaminoglycan degradation* |  |
|  | | *4-diphosphocytidyl-2C-methyl-D-erythritol synthase* | | *Isoprenoid biosynthesis* |  |
| 716 | | *Acetate Kinase* | | *Organic Acids* |  |
|  | | *Diaminopimelate epimerase* | | *Amino acid synthesis* |  |
|  | | *Glutamate synthase large and small subunit (NADPH)* | | *Amino acid synthesis* |  |
| 717 | | *N-acetylmuramoyl-L-alanine amidase* | | *Glycan Biosynthesis and Metabolism* |  |
|  | | *Cysteine synthase A* | | *Amino acid synthesis* |  |
|  | | *Alanine racemase* | | *Amino acid synthesis* |  |
| 718 | | *Alanine racemase* | | *Amino acid synthesis* |  |
|  | | *Orotidine-5-phosphate decarboxylase* | | *Pyrimidine metabolism* |  |
|  | | *N-acetylmuramoyl-L-alanine amidase* | | *Glycan Biosynthesis and Metabolism* |  |
| 719 | | *Beta-D-galactosidase* | | *Glycan structures - degradation;Complex Carbohydrates* |  |
|  | | *Thioredoxin reductase FAD-NADP-binding* | | *Pyrimidine metabolism* |  |
|  | | *Pyruvate-Formate Lyase* | | *Respiration* |  |
| 720 | | *Cytosine deaminase* | | *Pyrimidine metabolism* |  |
|  | | *Glutamate synthase large and small subunit (NADPH)* | | *Amino acid synthesis* |  |
|  | | *Pyruvate-Formate Lyase* | | *Respiration* |  |
| 721 | | *Histidinol dehydrogenase* | | *Amino acid transport and metabolism* |  |
|  | | *Carbamoyl phosphate synthetase small subunit glutamine amidotransferase* | | *Pyrimidine metabolism* |  |
|  | | *N-acetylmuramoyl-L-alanine amidase* | | *Glycan Biosynthesis and Metabolism* |  |
| 722 | | *Beta-D-glucuronidase* | | *Glycan structures - degradation;Exotic Metabolisms* |  |
|  | | *Prephenate dehydrogenase* | | *Amino acid synthesis* |  |
|  | | *Ornithine carbamoyltransferase 1* | | *Amino acid transport and metabolism* |  |
| 723 | | *Carbamoyl phosphate synthetase small subunit glutamine amidotransferase* | | *Pyrimidine metabolism* |  |
|  | | *Dihydrodipicolinate synthase* | | *Amino acid synthesis* |  |
|  | | *Diaminopimelate epimerase* | | *Amino acid synthesis* |  |
| 724 | | *Prephenate dehydrogenase* | | *Amino acid synthesis* |  |
|  | | *Histidinol dehydrogenase* | | *Amino acid transport and metabolism* |  |
|  | | *Beta-D-glucuronidase* | | *Glycan structures - degradation;Exotic Metabolisms* |  |
| 725 | | *Methylmalonyl-CaA decarboxylase* | | *Organic Acids* |  |
|  | | *Diaminopimelate epimerase* | | *Amino acid synthesis* |  |
|  | | *Cytosine deaminase* | | *Pyrimidine metabolism* |  |
| 726 | | *Phosphogluconate dehydratase* | | *Central Carbon Metabolism Pathways* |  |
|  | | *Prephenate dehydrogenase* | | *Amino acid synthesis* |  |
|  | | *Conjugated Bile Salt Hydrolase* | | *Exotic Metabolisms* |  |
| 727 | | *Alanine racemase* | | *Amino acid synthesis* |  |
|  | | *Prephenate dehydrogenase* | | *Amino acid synthesis* |  |
|  | | *Beta-D-glucuronidase* | | *Glycan structures - degradation;Exotic Metabolisms* |  |
| 728 | | *Pyridoxal Kinase* | | *Cofactor Biosynthesis* |  |
|  | | *N-acetyl-D-galactosamine-4-sulfate 4-sulfohydrolase* | | *Glycosaminoglycan degradation* |  |
|  | | *N-acetylmuramoyl-L-alanine amidase* | | *Glycan Biosynthesis and Metabolism* |  |
| 729 | | *N-acetylmuramoyl-L-alanine amidase* | | *Glycan Biosynthesis and Metabolism* |  |
|  | | *UDP-N-acetylglucosamine acyltransferase* | | *Glycan Biosynthesis and Metabolism* |  |
|  | | *Glucuronate Isomerase* | | *Feeder Pathways to Glycolysis* |  |
| 730 | | *N-acetylmuramoyl-L-alanine amidase* | | *Glycan Biosynthesis and Metabolism* |  |
|  | | *L-Lactate Dehydrogenase* | | *Organic Acids* |  |
|  | | *Pantothenate Synthetase* | | *Cofactor Biosynthesis* |  |
| 731 | | *Orotidine-5-phosphate decarboxylase* | | *Pyrimidine metabolism* |  |
|  | | *Prephenate dehydrogenase* | | *Amino acid synthesis* |  |
|  | | *UDP-N-acetylmuramateL-alanine ligase* | | *Glycan Biosynthesis and Metabolism* |  |
| 732 | | *Pyruvate-Formate Lyase* | | *Respiration* |  |
|  | | *Pyruvate-Formate Lyase* | | *Respiration* |  |
|  | | *IMP dehydrogenase* | | *Purine metabolism* |  |
| 733 | | *N-Acetylglucosamine-6-Phosphate Deacetylase* | | *Feeder Pathways to Glycolysis* |  |
|  | | *Prephenate dehydrogenase* | | *Amino acid synthesis* |  |
|  | | *Alpha-Glucosidase* | | *Complex Carbohydrates* |  |
| 734 | | *Pyrroline-5-carboxylate reductase* | | *Amino acid transport and metabolism* |  |
|  | | *N-acetylmuramoyl-L-alanine amidase* | | *Glycan Biosynthesis and Metabolism* |  |
|  | | *Homoserine dehydrogenase* | | *Amino acid transport and metabolism* |  |
| 735 | | *N-acetylmuramoyl-L-alanine amidase* | | *Glycan Biosynthesis and Metabolism* |  |
|  | | *Alpha-Glucosidase* | | *Complex Carbohydrates* |  |
|  | | *Alpha-mannosidase* | | *N-Glycan degradation* |  |
| 736 | | *Pyridoxal Kinase* | | *Cofactor Biosynthesis* |  |
|  | | *Biotin Synthase* | | *Cofactor Biosynthesis* |  |
|  | | *Diaminopimelate epimerase* | | *Amino acid synthesis* |  |
| 737 | | *Anthranilate synthase* | | *Amino acid transport and metabolism* |  |
|  | | *Prephenate dehydrogenase* | | *Amino acid synthesis* |  |
|  | | *Beta-ketoacyl-acyl-carrier-protein synthase III* | | *Fatty Acid Biosynthesis* |  |
| 738 | | *Ribokinase* | | *Feeder Pathways to Glycolysis* |  |
|  | | *Ribokinase* | | *Feeder Pathways to Glycolysis* |  |
|  | | *N-acetylmuramoyl-L-alanine amidase* | | *Glycan Biosynthesis and Metabolism* |  |
| 739 | | *Beta-D-galactosidase* | | *Glycan structures - degradation;Complex Carbohydrates* |  |
|  | | *Pyruvate-Formate Lyase* | | *Respiration* |  |
|  | | *IMP dehydrogenase* | | *Purine metabolism* |  |
| 740 | | *Adenylosuccinate synthetase* | | *Purine metabolism* |  |
|  | | *Acetyl-CoA acyltransferase anaerobic* | | *Fatty Acid Metabolism* |  |
|  | | *Prephenate dehydrogenase* | | *Amino acid synthesis* |  |
| 741 | | *Beta-D-glucuronidase* | | *Glycan structures - degradation;Exotic Metabolisms* |  |
|  | | *Pyruvate-Formate Lyase* | | *Respiration* |  |
|  | | *Shikimate kinase I II* | | *Amino acid transport and metabolism* |  |
| 742 | | *Alpha-Glucosidase* | | *Complex Carbohydrates* |  |
|  | | *Pyruvate-Formate Lyase* | | *Respiration* |  |
|  | | *Xylose Isomerase* | | *Feeder Pathways to Glycolysis* |  |
| 743 | | *Thioredoxin reductase FAD-NADP-binding* | | *Pyrimidine metabolism* |  |
|  | | *Diaminopimelate epimerase* | | *Amino acid synthesis* |  |
|  | | *Cysteine synthase A* | | *Amino acid synthesis* |  |
| 744 | | *Diaminopimelate epimerase* | | *Amino acid synthesis* |  |
|  | | *Methylmalonyl-CaA decarboxylase* | | *Organic Acids* |  |
|  | | *Alpha-Glucosidase* | | *Complex Carbohydrates* |  |
| 745 | | *Pyruvate-Formate Lyase* | | *Respiration* |  |
|  | | *Ribokinase* | | *Feeder Pathways to Glycolysis* |  |
|  | | *4-diphosphocytidyl-2C-methyl-D-erythritol synthase* | | *Isoprenoid biosynthesis* |  |
| 746 | | *Beta-D-glucuronidase* | | *Glycan structures - degradation;Exotic Metabolisms* |  |
|  | | *Diaminopimelate epimerase* | | *Amino acid synthesis* |  |
|  | | *Cytosine deaminase* | | *Pyrimidine metabolism* |  |
| 747 | | *N-acetylmuramoyl-L-alanine amidase* | | *Glycan Biosynthesis and Metabolism* |  |
|  | | *N-acetylmuramoyl-L-alanine amidase* | | *Glycan Biosynthesis and Metabolism* |  |
|  | | *N-acetylmuramoyl-L-alanine amidase* | | *Glycan Biosynthesis and Metabolism* |  |
| 748 | | *Cysteine synthase A* | | *Amino acid synthesis* |  |
|  | | *Anthranilate synthase* | | *Amino acid transport and metabolism* |  |
|  | | *N-acetylmuramoyl-L-alanine amidase* | | *Glycan Biosynthesis and Metabolism* |  |
| 749 | | *Ribokinase* | | *Feeder Pathways to Glycolysis* |  |
|  | | *Aspartate-ammonia ligase* | | *Amino acid synthesis* |  |
|  | | *Diaminopimelate epimerase* | | *Amino acid synthesis* |  |
| 750 | | *Conjugated Bile Salt Hydrolase* | | *Exotic Metabolisms* |  |
|  | | *Beta-D-galactosidase* | | *Glycan structures - degradation;Complex Carbohydrates* |  |
|  | | *Pyruvate-Formate Lyase* | | *Respiration* |  |
| 751 | | *Xylose Isomerase* | | *Feeder Pathways to Glycolysis* |  |
|  | | *Pyridoxal Kinase* | | *Cofactor Biosynthesis* |  |
|  | | *Pyruvate-Formate Lyase* | | *Respiration* |  |
| 752 | | *Ribokinase* | | *Feeder Pathways to Glycolysis* |  |
|  | | *Beta-D-galactosidase* | | *Glycan structures - degradation;Complex Carbohydrates* |  |
|  | | *Diaminopimelate epimerase* | | *Amino acid synthesis* |  |
| 753 | | *Xylose Isomerase* | | *Feeder Pathways to Glycolysis* |  |
|  | | *ketol-acid reductoisomerase* | | *Amino acid synthesis* |  |
|  | | *Pyruvate-Formate Lyase* | | *Respiration* |  |
| 754 | | *Diaminopimelate epimerase* | | *Amino acid synthesis* |  |
|  | | *Formyltetrahydrofolate synthetase* | | *Organic Acids* |  |
|  | | *Alanine racemase* | | *Amino acid synthesis* |  |
| 755 | | *L-threonine aldolase* | | *Amino acid synthesis* |  |
|  | | *Transketolase* | | *Central Carbon Metabolism Pathways* |  |
|  | | *Prephenate dehydrogenase* | | *Amino acid synthesis* |  |
| 756 | | *N-acetylmuramoyl-L-alanine amidase* | | *Glycan Biosynthesis and Metabolism* |  |
|  | | *KDPG Aldolase* | | *Central Carbon Metabolism Pathways* |  |
|  | | *Acetyl-CoA acyltransferase anaerobic* | | *Fatty Acid Metabolism* |  |
| 757 | | *Selenocysteine synthase* | | *Amino acid synthesis;Amino acid synthesis* |  |
|  | | *Prephenate dehydrogenase* | | *Amino acid synthesis* |  |
|  | | *3-deoxy-7-phosphoheptulonate synthase* | | *Amino acid transport and metabolism* |  |
| 758 | | *Threonine ammonia-lyase* | | *Amino acid synthesis* |  |
|  | | *N-acetyl-D-glucosamine-6-sulfate 6-sulfohydrolase* | | *Glycosaminoglycan degradation* |  |
|  | | *Pyruvate-Formate Lyase* | | *Respiration* |  |
| 759 | | *3-isopropylmalate dehydrogenase* | | *Amino acid synthesis* |  |
|  | | *Uridine phosphorylase* | | *Pyrimidine metabolism* |  |
|  | | *Diaminopimelate epimerase* | | *Amino acid synthesis* |  |
| 760 | | *Histidinol dehydrogenase* | | *Amino acid transport and metabolism* |  |
|  | | *Transaldolase* | | *Central Carbon Metabolism Pathways* |  |
|  | | *Diaminopimelate epimerase* | | *Amino acid synthesis* |  |
| 761 | | *Glucuronate Isomerase* | | *Feeder Pathways to Glycolysis* |  |
|  | | *N-acetylglutamate synthase* | | *Amino acid transport and metabolism* |  |
|  | | *Pyruvate-Formate Lyase* | | *Respiration* |  |
| 762 | | *Prephenate dehydrogenase* | | *Amino acid synthesis* |  |
|  | | *Beta-ketoacyl-acyl-carrier-protein synthase III* | | *Fatty Acid Biosynthesis* |  |
|  | | *Glutamate racemase* | | *Amino acid synthesis* |  |
| 763 | | *Pyruvate-Formate Lyase* | | *Respiration* |  |
|  | | *N-acetyl-D-galactosamine-4-sulfate 4-sulfohydrolase* | | *Glycosaminoglycan degradation* |  |
|  | | *Biotin Synthase* | | *Cofactor Biosynthesis* |  |
| 764 | | *Cysteine synthase A* | | *Amino acid synthesis* |  |
|  | | *Pyruvate-Formate Lyase* | | *Respiration* |  |
|  | | *N-acetyl-D-galactosamine-4-sulfate 4-sulfohydrolase* | | *Glycosaminoglycan degradation* |  |
| 765 | | *Cytosine deaminase* | | *Pyrimidine metabolism* |  |
|  | | *Pyridoxal Kinase* | | *Cofactor Biosynthesis* |  |
|  | | *Pyruvate-Formate Lyase* | | *Respiration* |  |
| 766 | | *Beta-D-glucuronidase* | | *Glycan structures - degradation;Exotic Metabolisms* |  |
|  | | *Prephenate dehydrogenase* | | *Amino acid synthesis* |  |
|  | | *L-glutamine synthase* | | *Amino acid synthesis* |  |
| 767 | | *Beta-D-galactosidase* | | *Glycan structures - degradation;Complex Carbohydrates* |  |
|  | | *Pyruvate-Formate Lyase* | | *Respiration* |  |
|  | | *Orotidine-5-phosphate decarboxylase* | | *Pyrimidine metabolism* |  |
| 768 | | *Pantothenate Synthetase* | | *Cofactor Biosynthesis* |  |
|  | | *3-deoxy-7-phosphoheptulonate synthase* | | *Amino acid transport and metabolism* |  |
|  | | *Prephenate dehydrogenase* | | *Amino acid synthesis* |  |
| 769 | | *Thioredoxin reductase FAD-NADP-binding* | | *Pyrimidine metabolism* |  |
|  | | *Prephenate dehydrogenase* | | *Amino acid synthesis* |  |
|  | | *Adenylosuccinate synthetase* | | *Purine metabolism* |  |
| 770 | | *Ribokinase* | | *Feeder Pathways to Glycolysis* |  |
|  | | *Formyltetrahydrofolate synthetase* | | *Organic Acids* |  |
|  | | *Diaminopimelate epimerase* | | *Amino acid synthesis* |  |
| 771 | | *D-alanineD-alanine ligase* | | *Glycan Biosynthesis and Metabolism* |  |
|  | | *Prephenate dehydrogenase* | | *Amino acid synthesis* |  |
|  | | *Beta-D-glucuronidase* | | *Glycan structures - degradation;Exotic Metabolisms* |  |
| 772 | | *UDP-N-acetylmuramateL-alanine ligase* | | *Glycan Biosynthesis and Metabolism* |  |
|  | | *Prephenate dehydrogenase* | | *Amino acid synthesis* |  |
|  | | *Spermidine Synthase* | | *Nitrogen Metabolism;Amino acid transport and metabolism* |  |
| 773 | | *L-alanine dehydrogenase* | | *Amino acid synthesis* |  |
|  | | *Pyruvate-Formate Lyase* | | *Respiration* |  |
|  | | *Adenylosuccinate synthetase* | | *Purine metabolism* |  |
| 774 | | *N-acetylmuramoyl-L-alanine amidase* | | *Glycan Biosynthesis and Metabolism* |  |
|  | | *Butyrate Kinase* | | *Organic Acids* |  |
|  | | *Cobalamin Synthase* | | *Cofactor Biosynthesis* |  |
| 775 | | *Diaminopimelate epimerase* | | *Amino acid synthesis* |  |
|  | | *N-acetylmuramoyl-L-alanine amidase* | | *Glycan Biosynthesis and Metabolism* |  |
|  | | *Branched-chain-amino-acid transaminase* | | *Amino acid transport and metabolism* |  |
| 776 | | *Acetyl-CoA acyltransferase anaerobic* | | *Fatty Acid Metabolism* |  |
|  | | *Prephenate dehydrogenase* | | *Amino acid synthesis* |  |
|  | | *UDP-N-acetylglucosamine acyltransferase* | | *Glycan Biosynthesis and Metabolism* |  |
| 777 | | *Pyruvate-Formate Lyase* | | *Respiration* |  |
|  | | *Cytosine deaminase* | | *Pyrimidine metabolism* |  |
|  | | *Acetyl-CoA acyltransferase anaerobic* | | *Fatty Acid Metabolism* |  |
| 778 | | *Acetyl-CoA acyltransferase anaerobic* | | *Fatty Acid Metabolism* |  |
|  | | *Diaminopimelate epimerase* | | *Amino acid synthesis* |  |
|  | | *N-acetyl-D-galactosamine-4-sulfate 4-sulfohydrolase* | | *Glycosaminoglycan degradation* |  |
| 779 | | *Diaminopimelate epimerase* | | *Amino acid synthesis* |  |
|  | | *3-demethylubiquinone-9 3-methyltransferase* | | *Cofactor Biosynthesis* |  |
|  | | *Galactokinase* | | *Feeder Pathways to Glycolysis* |  |
| 780 | | *Adenylosuccinate synthetase* | | *Purine metabolism* |  |
|  | | *Fucose Isomerase* | | *Feeder Pathways to Glycolysis* |  |
|  | | *Diaminopimelate epimerase* | | *Amino acid synthesis* |  |
| 781 | | *Chorismate synthase* | | *Amino acid transport and metabolism* |  |
|  | | *N-acetylmuramoyl-L-alanine amidase* | | *Glycan Biosynthesis and Metabolism* |  |
|  | | *UDP-N-acetylglucosamine acyltransferase* | | *Glycan Biosynthesis and Metabolism* |  |
| 782 | | *Glutamate racemase* | | *Amino acid synthesis* |  |
|  | | *Ornithine carbamoyltransferase 1* | | *Amino acid transport and metabolism* |  |
|  | | *Prephenate dehydrogenase* | | *Amino acid synthesis* |  |
| 783 | | *Prephenate dehydrogenase* | | *Amino acid synthesis* |  |
|  | | *Carbamoyl phosphate synthetase small subunit glutamine amidotransferase* | | *Pyrimidine metabolism* |  |
|  | | *L-threonine synthase* | | *Amino acid synthesis* |  |
| 784 | | *Prephenate dehydrogenase* | | *Amino acid synthesis* |  |
|  | | *Branched-chain-amino-acid transaminase* | | *Amino acid transport and metabolism* |  |
|  | | *Glutamate racemase* | | *Amino acid synthesis* |  |
| 785 | | *Thioredoxin reductase FAD-NADP-binding* | | *Pyrimidine metabolism* |  |
|  | | *N-acetyl-D-galactosamine-4-sulfate 4-sulfohydrolase* | | *Glycosaminoglycan degradation* |  |
|  | | *Prephenate dehydrogenase* | | *Amino acid synthesis* |  |
| 786 | | *Transketolase* | | *Central Carbon Metabolism Pathways* |  |
|  | | *N-Acetylglucosamine-6-Phosphate Deacetylase* | | *Feeder Pathways to Glycolysis* |  |
|  | | *Prephenate dehydrogenase* | | *Amino acid synthesis* |  |
| 787 | | *Beta-D-galactosidase* | | *Glycan structures - degradation;Complex Carbohydrates* |  |
|  | | *Shikimate kinase I II* | | *Amino acid transport and metabolism* |  |
|  | | *Prephenate dehydrogenase* | | *Amino acid synthesis* |  |
| 788 | | *KDPG Aldolase* | | *Central Carbon Metabolism Pathways* |  |
|  | | *Beta-D-galactosidase* | | *Glycan structures - degradation;Complex Carbohydrates* |  |
|  | | *Prephenate dehydrogenase* | | *Amino acid synthesis* |  |
| 789 | | *Alpha-Glucosidase* | | *Complex Carbohydrates* |  |
|  | | *Acetyl-CoA acyltransferase anaerobic* | | *Fatty Acid Metabolism* |  |
|  | | *Pyruvate-Formate Lyase* | | *Respiration* |  |
| 790 | | *Cytidylate kinase* | | *Pyrimidine metabolism* |  |
|  | | *Prephenate dehydrogenase* | | *Amino acid synthesis* |  |
|  | | *Asparaginase* | | *Amino acid synthesis* |  |
| 791 | | *Histidinol dehydrogenase* | | *Amino acid transport and metabolism* |  |
|  | | *Ribokinase* | | *Feeder Pathways to Glycolysis* |  |
|  | | *Beta-D-galactosidase* | | *Glycan structures - degradation;Complex Carbohydrates* |  |
| 792 | | *UDP-N-acetylmuramateL-alanine ligase* | | *Glycan Biosynthesis and Metabolism* |  |
|  | | *N-acetylmuramoyl-L-alanine amidase* | | *Glycan Biosynthesis and Metabolism* |  |
|  | | *Cytidylate kinase* | | *Pyrimidine metabolism* |  |
| 793 | | *UDP-N-acetylmuramoyl-L-alanineD-glutamate ligase* | | *Glycan Biosynthesis and Metabolism* |  |
|  | | *Adenylosuccinate synthetase* | | *Purine metabolism* |  |
|  | | *Pyruvate-Formate Lyase* | | *Respiration* |  |
| 794 | | *Conjugated Bile Salt Hydrolase* | | *Exotic Metabolisms* |  |
|  | | *ADP-ribose pyrophosphatase* | | *Purine metabolism* |  |
|  | | *N-acetylmuramoyl-L-alanine amidase* | | *Glycan Biosynthesis and Metabolism* |  |
| 795 | | *N-Acetylglucosamine-6-Phosphate Deacetylase* | | *Feeder Pathways to Glycolysis* |  |
|  | | *N-acetylmuramoyl-L-alanine amidase* | | *Glycan Biosynthesis and Metabolism* |  |
|  | | *4-diphosphocytidyl-2C-methyl-D-erythritol synthase* | | *Isoprenoid biosynthesis* |  |
| 796 | | *L-glutamine synthase* | | *Amino acid synthesis* |  |
|  | | *N-acetylmuramoyl-L-alanine amidase* | | *Glycan Biosynthesis and Metabolism* |  |
|  | | *N-acetylmuramoyl-L-alanine amidase* | | *Glycan Biosynthesis and Metabolism* |  |
| 797 | | *ketol-acid reductoisomerase* | | *Amino acid synthesis* |  |
|  | | *Prephenate dehydrogenase* | | *Amino acid synthesis* |  |
|  | | *N-acetyl-D-galactosamine-4-sulfate 4-sulfohydrolase* | | *Glycosaminoglycan degradation* |  |
| 798 | | *Adenylosuccinate synthetase* | | *Purine metabolism* |  |
|  | | *Serine O-acetyltransferase* | | *Amino acid synthesis* |  |
|  | | *N-acetylmuramoyl-L-alanine amidase* | | *Glycan Biosynthesis and Metabolism* |  |
| 799 | | *Acetyl-CoA acyltransferase anaerobic* | | *Fatty Acid Metabolism* |  |
|  | | *Thioredoxin reductase FAD-NADP-binding* | | *Pyrimidine metabolism* |  |
|  | | *Diaminopimelate epimerase* | | *Amino acid synthesis* |  |
| 800 | | *IMP dehydrogenase* | | *Purine metabolism* |  |
|  | | *Thioredoxin reductase FAD-NADP-binding* | | *Pyrimidine metabolism* |  |
|  | | *Prephenate dehydrogenase* | | *Amino acid synthesis* |  |
| 801 | | *N-acetylglutamate synthase* | | *Amino acid transport and metabolism* |  |
|  | | *Pyruvate-Formate Lyase* | | *Respiration* |  |
|  | | *Biosynthetic arginine decarboxylase PLP-binding* | | *Amino acid transport and metabolism* |  |
| 802 | | *Argininosuccinate lyase* | | *Amino acid transport and metabolism* |  |
|  | | *Alanine racemase* | | *Amino acid synthesis* |  |
|  | | *Diaminopimelate epimerase* | | *Amino acid synthesis* |  |
| 803 | | *Spermidine Synthase* | | *Nitrogen Metabolism;Amino acid transport and metabolism* |  |
|  | | *Dihydrodipicolinate synthase* | | *Amino acid synthesis* |  |
|  | | *Prephenate dehydrogenase* | | *Amino acid synthesis* |  |
| 804 | | *IMP dehydrogenase* | | *Purine metabolism* |  |
|  | | *Branched-chain-amino-acid transaminase* | | *Amino acid transport and metabolism* |  |
|  | | *N-acetylmuramoyl-L-alanine amidase* | | *Glycan Biosynthesis and Metabolism* |  |
| 805 | | *Glycerol Kinase* | | *Glycerolipid Metabolism* |  |
|  | | *Diaminopimelate epimerase* | | *Amino acid synthesis* |  |
|  | | *L-alanine dehydrogenase* | | *Amino acid synthesis* |  |
| 806 | | *N-acetylmuramoyl-L-alanine amidase* | | *Glycan Biosynthesis and Metabolism* |  |
|  | | *3-dehydroquinate dehydratase* | | *Amino acid transport and metabolism* |  |
|  | | *Orotidine-5-phosphate decarboxylase* | | *Pyrimidine metabolism* |  |
| 807 | | *Dihydroxy-acid dehydratase* | | *Amino acid transport and metabolism* |  |
|  | | *UDP-N-acetylmuramateL-alanine ligase* | | *Glycan Biosynthesis and Metabolism* |  |
|  | | *N-acetylmuramoyl-L-alanine amidase* | | *Glycan Biosynthesis and Metabolism* |  |
| 808 | | *N-acetylmuramoyl-L-alanine amidase* | | *Glycan Biosynthesis and Metabolism* |  |
|  | | *Glycerol Kinase* | | *Glycerolipid Metabolism* |  |
|  | | *Beta-D-galactosidase* | | *Glycan structures - degradation;Complex Carbohydrates* |  |
| 809 | | *Methionyl-tRNA synthetase* | | *Amino acid synthesis* |  |
|  | | *Pyruvate-Formate Lyase* | | *Respiration* |  |
|  | | *Cysteine synthase A* | | *Amino acid synthesis* |  |
| 810 | | *N-acetylmuramoyl-L-alanine amidase* | | *Glycan Biosynthesis and Metabolism* |  |
|  | | *Homoserine dehydrogenase* | | *Amino acid transport and metabolism* |  |
|  | | *Carbon Monoxide Dehydrogenase* | | *Central Carbon Metabolism Pathways* |  |
| 811 | | *Diaminopimelate epimerase* | | *Amino acid synthesis* |  |
|  | | *Acetylglutamate kinase* | | *Amino acid transport and metabolism* |  |
|  | | *Pyridoxal Kinase* | | *Cofactor Biosynthesis* |  |
| 812 | | *Beta-D-galactosidase* | | *Glycan structures - degradation;Complex Carbohydrates* |  |
|  | | *Pyruvate-Formate Lyase* | | *Respiration* |  |
|  | | *Cystathionine gamma-synthase* | | *Amino acid synthesis* |  |
| 813 | | *Dihydroxy-acid dehydratase* | | *Amino acid transport and metabolism* |  |
|  | | *Adenylosuccinate synthetase* | | *Purine metabolism* |  |
|  | | *Diaminopimelate epimerase* | | *Amino acid synthesis* |  |
| 814 | | *N-Acetylglucosamine-6-Phosphate Deacetylase* | | *Feeder Pathways to Glycolysis* |  |
|  | | *Diaminopimelate epimerase* | | *Amino acid synthesis* |  |
|  | | *IMP dehydrogenase* | | *Purine metabolism* |  |
| 815 | | *Pyruvate-Formate Lyase* | | *Respiration* |  |
|  | | *Phosphogluconate dehydratase* | | *Central Carbon Metabolism Pathways* |  |
|  | | *Diaminopimelate epimerase* | | *Amino acid synthesis* |  |
| 816 | | *Uridine phosphorylase* | | *Pyrimidine metabolism* |  |
|  | | *N-acetylmuramoyl-L-alanine amidase* | | *Glycan Biosynthesis and Metabolism* |  |
|  | | *Formyltetrahydrofolate synthetase* | | *Organic Acids* |  |
| 817 | | *Threonine ammonia-lyase* | | *Amino acid synthesis* |  |
|  | | *Diaminopimelate epimerase* | | *Amino acid synthesis* |  |
|  | | *Prephenate dehydrogenase* | | *Amino acid synthesis* |  |
| 818 | | *Beta-D-galactosidase* | | *Glycan structures - degradation;Complex Carbohydrates* |  |
|  | | *N-acetylmuramoyl-L-alanine amidase* | | *Glycan Biosynthesis and Metabolism* |  |
|  | | *Agamintase* | | *Nitrogen Metabolism* |  |
| 819 | | *Prephenate dehydrogenase* | | *Amino acid synthesis* |  |
|  | | *Cytidylate kinase* | | *Pyrimidine metabolism* |  |
|  | | *Homoserine dehydrogenase* | | *Amino acid transport and metabolism* |  |
| 820 | | *Folylpolyglutamate Synthase* | | *Cofactor Biosynthesis* |  |
|  | | *N-acetylmuramoyl-L-alanine amidase* | | *Glycan Biosynthesis and Metabolism* |  |
|  | | *Beta-D-glucuronidase* | | *Glycan structures - degradation;Exotic Metabolisms* |  |
| 821 | | *Prephenate dehydrogenase* | | *Amino acid synthesis* |  |
|  | | *1-Phosphofructokinase* | | *Feeder Pathways to Glycolysis* |  |
|  | | *Ribokinase* | | *Feeder Pathways to Glycolysis* |  |
| 822 | | *Homoserine dehydrogenase* | | *Amino acid transport and metabolism* |  |
|  | | *Diaminopimelate epimerase* | | *Amino acid synthesis* |  |
|  | | *Beta-D-galactosidase* | | *Glycan structures - degradation;Complex Carbohydrates* |  |
| 823 | | *Galactokinase* | | *Feeder Pathways to Glycolysis* |  |
|  | | *Diaminopimelate epimerase* | | *Amino acid synthesis* |  |
|  | | *Valine-pyruvate aminotransferase* | | *Amino acid synthesis* |  |
| 824 | | *UDP-N-acetylmuramateL-alanine ligase* | | *Glycan Biosynthesis and Metabolism* |  |
|  | | *4-diphosphocytidyl-2C-methyl-D-erythritol synthase* | | *Isoprenoid biosynthesis* |  |
|  | | *Prephenate dehydrogenase* | | *Amino acid synthesis* |  |
| 825 | | *Diaminopimelate epimerase* | | *Amino acid synthesis* |  |
|  | | *sulfate adenylyltransferase subunit 2* | | *Purine metabolism* |  |
|  | | *Histidinol dehydrogenase* | | *Amino acid transport and metabolism* |  |
| 826 | | *N-acetylmuramoyl-L-alanine amidase* | | *Glycan Biosynthesis and Metabolism* |  |
|  | | *Arginosuccinate synthase* | | *Amino acid synthesis* |  |
|  | | *Glycerol Kinase* | | *Glycerolipid Metabolism* |  |
| 827 | | *Alanine racemase* | | *Amino acid synthesis* |  |
|  | | *IMP dehydrogenase* | | *Purine metabolism* |  |
|  | | *Prephenate dehydrogenase* | | *Amino acid synthesis* |  |
| 828 | | *Pyridoxal Kinase* | | *Cofactor Biosynthesis* |  |
|  | | *Pyruvate-Formate Lyase* | | *Respiration* |  |
|  | | *Beta-D-glucuronidase* | | *Glycan structures - degradation;Exotic Metabolisms* |  |
| 829 | | *Cystathionine gamma-synthase* | | *Amino acid synthesis* |  |
|  | | *Diaminopimelate epimerase* | | *Amino acid synthesis* |  |
|  | | *Dihydroxy-acid dehydratase* | | *Amino acid transport and metabolism* |  |
| 830 | | *D-alanineD-alanine ligase* | | *Glycan Biosynthesis and Metabolism* |  |
|  | | *Threonine ammonia-lyase* | | *Amino acid synthesis* |  |
|  | | *Diaminopimelate epimerase* | | *Amino acid synthesis* |  |
| 831 | | *Ornithine carbamoyltransferase 1* | | *Amino acid transport and metabolism* |  |
|  | | *Cytosine deaminase* | | *Pyrimidine metabolism* |  |
|  | | *N-acetylmuramoyl-L-alanine amidase* | | *Glycan Biosynthesis and Metabolism* |  |
| 832 | | *Pyruvate-Formate Lyase* | | *Respiration* |  |
|  | | *Geranyltranstransferase* | | *Isoprenoid biosynthesis* |  |
|  | | *L-glutamine synthase* | | *Amino acid synthesis* |  |
| 833 | | *Carbon Monoxide Dehydrogenase* | | *Central Carbon Metabolism Pathways* |  |
|  | | *Glutamate synthase large and small subunit (NADPH)* | | *Amino acid synthesis* |  |
|  | | *Pyruvate-Formate Lyase* | | *Respiration* |  |
| 834 | | *Carbon Monoxide Dehydrogenase* | | *Central Carbon Metabolism Pathways* |  |
|  | | *N-acetylmuramoyl-L-alanine amidase* | | *Glycan Biosynthesis and Metabolism* |  |
|  | | *3-deoxy-7-phosphoheptulonate synthase* | | *Amino acid transport and metabolism* |  |
| 835 | | *Diaminopimelate epimerase* | | *Amino acid synthesis* |  |
|  | | *Gamma-glutamyl kinase* | | *Amino acid transport and metabolism* |  |
|  | | *Pyruvate-Formate Lyase* | | *Respiration* |  |
| 836 | | *Diaminopimelate epimerase* | | *Amino acid synthesis* |  |
|  | | *sulfate adenylyltransferase subunit 2* | | *Purine metabolism* |  |
|  | | *Beta-ketoacyl-acyl-carrier-protein synthase III* | | *Fatty Acid Biosynthesis* |  |
| 837 | | *N-acetylmuramoyl-L-alanine amidase* | | *Glycan Biosynthesis and Metabolism* |  |
|  | | *Quinolinate Synthase* | | *Cofactor Biosynthesis* |  |
|  | | *Pyruvate-Formate Lyase* | | *Respiration* |  |
| 838 | | *Anthranilate synthase* | | *Amino acid transport and metabolism* |  |
|  | | *Prephenate dehydrogenase* | | *Amino acid synthesis* |  |
|  | | *Ornithine carbamoyltransferase 1* | | *Amino acid transport and metabolism* |  |
| 839 | | *L-glutamine synthase* | | *Amino acid synthesis* |  |
|  | | *Butyrate Kinase* | | *Organic Acids* |  |
|  | | *Diaminopimelate epimerase* | | *Amino acid synthesis* |  |
| 840 | | *Folylpolyglutamate Synthase* | | *Cofactor Biosynthesis* |  |
|  | | *Dihydrodipicolinate synthase* | | *Amino acid synthesis* |  |
|  | | *Diaminopimelate epimerase* | | *Amino acid synthesis* |  |
| 841 | | *Pyruvate-Formate Lyase* | | *Respiration* |  |
|  | | *Phosphoribosylglycinamide synthetase phosphoribosylamine-glycine ligase* | | *Purine metabolism* |  |
|  | | *Diaminopimelate epimerase* | | *Amino acid synthesis* |  |
| 842 | | *N-acetylmuramoyl-L-alanine amidase* | | *Glycan Biosynthesis and Metabolism* |  |
|  | | *Prephenate dehydrogenase* | | *Amino acid synthesis* |  |
|  | | *Cytosine deaminase* | | *Pyrimidine metabolism* |  |
| 843 | | *UDP-N-acetylglucosamine acyltransferase* | | *Glycan Biosynthesis and Metabolism* |  |
|  | | *Carbamoyl phosphate synthetase small subunit glutamine amidotransferase* | | *Pyrimidine metabolism* |  |
|  | | *Prephenate dehydrogenase* | | *Amino acid synthesis* |  |
| 844 | | *Diaminopimelate epimerase* | | *Amino acid synthesis* |  |
|  | | *Spermidine Synthase* | | *Nitrogen Metabolism;Amino acid transport and metabolism* |  |
|  | | *UDP-N-acetylmuramateL-alanine ligase* | | *Glycan Biosynthesis and Metabolism* |  |
| 845 | | *Alanine racemase* | | *Amino acid synthesis* |  |
|  | | *Prephenate dehydrogenase* | | *Amino acid synthesis* |  |
|  | | *Alpha-mannosidase* | | *N-Glycan degradation* |  |
| 846 | | *Pyruvate-Formate Lyase* | | *Respiration* |  |
|  | | *N-Acetylglucosamine-6-Phosphate Deacetylase* | | *Feeder Pathways to Glycolysis* |  |
|  | | *Acetate Kinase* | | *Organic Acids* |  |
| 847 | | *L-threonine aldolase* | | *Amino acid synthesis* |  |
|  | | *Carbamoyl phosphate synthetase small subunit glutamine amidotransferase* | | *Pyrimidine metabolism* |  |
|  | | *Pyruvate-Formate Lyase* | | *Respiration* |  |
| 848 | | *Alpha-Glucosidase* | | *Complex Carbohydrates* |  |
|  | | *Acetyl-CoA acyltransferase anaerobic* | | *Fatty Acid Metabolism* |  |
|  | | *Prephenate dehydrogenase* | | *Amino acid synthesis* |  |
| 849 | | *Ribokinase* | | *Feeder Pathways to Glycolysis* |  |
|  | | *4-diphosphocytidyl-2C-methyl-D-erythritol synthase* | | *Isoprenoid biosynthesis* |  |
|  | | *Diaminopimelate epimerase* | | *Amino acid synthesis* |  |
| 850 | | *Alpha-mannosidase* | | *N-Glycan degradation* |  |
|  | | *Diaminopimelate epimerase* | | *Amino acid synthesis* |  |
|  | | *Ribokinase* | | *Feeder Pathways to Glycolysis* |  |
| 851 | | *Branched-chain-amino-acid transaminase* | | *Amino acid transport and metabolism* |  |
|  | | *N-acetylmuramoyl-L-alanine amidase* | | *Glycan Biosynthesis and Metabolism* |  |
|  | | *UDP-N-acetylmuramoyl-L-alanyl-D-glutamatemeso-diaminopimelate ligase* | | *Glycan Biosynthesis and Metabolism* |  |
| 852 | | *Beta-N-acetyl-D-hexosaminide N-acetylhexosaminohydrolase* | | *Glycosaminoglycan degradation* |  |
|  | | *Diaminopimelate epimerase* | | *Amino acid synthesis* |  |
|  | | *Methylmalonyl-CaA decarboxylase* | | *Organic Acids* |  |
| 853 | | *Cystathionine gamma-synthase* | | *Amino acid synthesis* |  |
|  | | *N-acetylmuramoyl-L-alanine amidase* | | *Glycan Biosynthesis and Metabolism* |  |
|  | | *Riboflavin Synthase α Subunit* | | *Cofactor Biosynthesis* |  |
| 854 | | *Cytidylate kinase* | | *Pyrimidine metabolism* |  |
|  | | *Pyruvate-Formate Lyase* | | *Respiration* |  |
|  | | *Transketolase* | | *Central Carbon Metabolism Pathways* |  |
| 855 | | *Cytosine deaminase* | | *Pyrimidine metabolism* |  |
|  | | *Cytidylate kinase* | | *Pyrimidine metabolism* |  |
|  | | *N-acetylmuramoyl-L-alanine amidase* | | *Glycan Biosynthesis and Metabolism* |  |
| 856 | | *Glutamate synthase large and small subunit (NADPH)* | | *Amino acid synthesis* |  |
|  | | *Butyrate Kinase* | | *Organic Acids* |  |
|  | | *Pyruvate-Formate Lyase* | | *Respiration* |  |
| 857 | | *Mannose-6-Phosphate Isomerase* | | *Feeder Pathways to Glycolysis* |  |
|  | | *Pyruvate-Formate Lyase* | | *Respiration* |  |
|  | | *N-acetyl-D-galactosamine-4-sulfate 4-sulfohydrolase* | | *Glycosaminoglycan degradation* |  |
| 858 | | *Uridine phosphorylase* | | *Pyrimidine metabolism* |  |
|  | | *Pyruvate-Formate Lyase* | | *Respiration* |  |
|  | | *UDP-N-acetylmuramoyl-L-alanyl-D-glutamatemeso-diaminopimelate ligase* | | *Glycan Biosynthesis and Metabolism* |  |
| 859 | | *Cystathionine gamma-synthase* | | *Amino acid synthesis* |  |
|  | | *Prephenate dehydrogenase* | | *Amino acid synthesis* |  |
|  | | *N-acetyl-D-galactosamine-4-sulfate 4-sulfohydrolase* | | *Glycosaminoglycan degradation* |  |
| 860 | | *Fucose Isomerase* | | *Feeder Pathways to Glycolysis* |  |
|  | | *Pyruvate-Formate Lyase* | | *Respiration* |  |
|  | | *Cytosine deaminase* | | *Pyrimidine metabolism* |  |
| 861 | | *Cytidylate kinase* | | *Pyrimidine metabolism* |  |
|  | | *Prephenate dehydrogenase* | | *Amino acid synthesis* |  |
|  | | *L-glutamine synthase* | | *Amino acid synthesis* |  |
| 862 | | *Pyruvate-Formate Lyase* | | *Respiration* |  |
|  | | *Acetyl-CoA acyltransferase anaerobic* | | *Fatty Acid Metabolism* |  |
|  | | *Ornithine carbamoyltransferase 1* | | *Amino acid transport and metabolism* |  |
| 863 | | *Beta-D-galactosidase* | | *Glycan structures - degradation;Complex Carbohydrates* |  |
|  | | *D-alanineD-alanine ligase* | | *Glycan Biosynthesis and Metabolism* |  |
|  | | *Pyruvate-Formate Lyase* | | *Respiration* |  |
| 864 | | *Glutamate synthase large and small subunit (NADPH)* | | *Amino acid synthesis* |  |
|  | | *3-deoxy-7-phosphoheptulonate synthase* | | *Amino acid transport and metabolism* |  |
|  | | *Diaminopimelate epimerase* | | *Amino acid synthesis* |  |
| 865 | | *Galactokinase* | | *Feeder Pathways to Glycolysis* |  |
|  | | *Diaminopimelate epimerase* | | *Amino acid synthesis* |  |
|  | | *Acetyl-CoA acyltransferase anaerobic* | | *Fatty Acid Metabolism* |  |
| 866 | | *Diaminopimelate epimerase* | | *Amino acid synthesis* |  |
|  | | *Agamintase* | | *Nitrogen Metabolism* |  |
|  | | *Cytosine deaminase* | | *Pyrimidine metabolism* |  |
| 867 | | *UDP-N-acetylmuramoyl-L-alanineD-glutamate ligase* | | *Glycan Biosynthesis and Metabolism* |  |
|  | | *Glutamate decarboxylase A and B PLP-dependent* | | *Amino acid transport and metabolism* |  |
|  | | *Prephenate dehydrogenase* | | *Amino acid synthesis* |  |
| 868 | | *Pyruvate-Formate Lyase* | | *Respiration* |  |
|  | | *Cytosine deaminase* | | *Pyrimidine metabolism* |  |
|  | | *Acetyl-CoA acyltransferase anaerobic* | | *Fatty Acid Metabolism* |  |
| 869 | | *Cysteine synthase A* | | *Amino acid synthesis* |  |
|  | | *Prephenate dehydrogenase* | | *Amino acid synthesis* |  |
|  | | *L-glutamine synthase* | | *Amino acid synthesis* |  |
| 870 | | *Pyridoxal Kinase* | | *Cofactor Biosynthesis* |  |
|  | | *Spermidine Synthase* | | *Nitrogen Metabolism;Amino acid transport and metabolism* |  |
|  | | *Prephenate dehydrogenase* | | *Amino acid synthesis* |  |
| 871 | | *Purine-nucleoside phosphorylase* | | *Purine metabolism* |  |
|  | | *Alanine racemase* | | *Amino acid synthesis* |  |
|  | | *Pyruvate-Formate Lyase* | | *Respiration* |  |
| 872 | | *Asparaginase* | | *Amino acid synthesis* |  |
|  | | *Shikimate kinase I II* | | *Amino acid transport and metabolism* |  |
|  | | *Pyruvate-Formate Lyase* | | *Respiration* |  |
| 873 | | *L-alanine dehydrogenase* | | *Amino acid synthesis* |  |
|  | | *Si-Citrate Synthase* | | *Central Carbon Metabolism Pathways* |  |
|  | | *Diaminopimelate epimerase* | | *Amino acid synthesis* |  |
| 874 | | *Beta-ketoacyl-acyl-carrier-protein synthase III* | | *Fatty Acid Biosynthesis* |  |
|  | | *Prephenate dehydrogenase* | | *Amino acid synthesis* |  |
|  | | *Cysteine synthase A* | | *Amino acid synthesis* |  |
| 875 | | *2-isopropylmalate synthase* | | *Amino acid synthesis* |  |
|  | | *3-deoxy-7-phosphoheptulonate synthase* | | *Amino acid transport and metabolism* |  |
|  | | *Prephenate dehydrogenase* | | *Amino acid synthesis* |  |
| 876 | | *Alpha-Glucosidase* | | *Complex Carbohydrates* |  |
|  | | *Diaminopimelate epimerase* | | *Amino acid synthesis* |  |
|  | | *Dihydrodipicolinate synthase* | | *Amino acid synthesis* |  |
| 877 | | *Cysteine synthase A* | | *Amino acid synthesis* |  |
|  | | *Pyruvate-Formate Lyase* | | *Respiration* |  |
|  | | *Beta-N-acetyl-D-hexosaminide N-acetylhexosaminohydrolase* | | *Glycosaminoglycan degradation* |  |
| 878 | | *Alanine racemase* | | *Amino acid synthesis* |  |
|  | | *Prephenate dehydrogenase* | | *Amino acid synthesis* |  |
|  | | *Pyrroline-5-carboxylate reductase* | | *Amino acid transport and metabolism* |  |
| 879 | | *Cytidylate kinase* | | *Pyrimidine metabolism* |  |
|  | | *N-acetylmuramoyl-L-alanine amidase* | | *Glycan Biosynthesis and Metabolism* |  |
|  | | *Glutamate decarboxylase A and B PLP-dependent* | | *Amino acid transport and metabolism* |  |
| 880 | | *N-acetylmuramoyl-L-alanine amidase* | | *Glycan Biosynthesis and Metabolism* |  |
|  | | *Carbon Monoxide Dehydrogenase* | | *Central Carbon Metabolism Pathways* |  |
|  | | *N-Acetylglucosamine-6-Phosphate Deacetylase* | | *Feeder Pathways to Glycolysis* |  |
| 881 | | *Pyridoxal Kinase* | | *Cofactor Biosynthesis* |  |
|  | | *N-acetylmuramoyl-L-alanine amidase* | | *Glycan Biosynthesis and Metabolism* |  |
|  | | *Alanine racemase* | | *Amino acid synthesis* |  |
| 882 | | *Pyruvate-Formate Lyase* | | *Respiration* |  |
|  | | *Beta-N-acetyl-D-hexosaminide N-acetylhexosaminohydrolase* | | *Glycosaminoglycan degradation* |  |
|  | | *4-diphosphocytidyl-2C-methyl-D-erythritol synthase* | | *Isoprenoid biosynthesis* |  |
| 883 | | *Asparaginase* | | *Amino acid synthesis* |  |
|  | | *Ribokinase* | | *Feeder Pathways to Glycolysis* |  |
|  | | *Pyruvate-Formate Lyase* | | *Respiration* |  |
| 884 | | *3-deoxy-7-phosphoheptulonate synthase* | | *Amino acid transport and metabolism* |  |
|  | | *Pyruvate-Formate Lyase* | | *Respiration* |  |
|  | | *UDP-N-acetylmuramateL-alanine ligase* | | *Glycan Biosynthesis and Metabolism* |  |
| 885 | | *Uridine phosphorylase* | | *Pyrimidine metabolism* |  |
|  | | *N-acetyl-D-galactosamine-4-sulfate 4-sulfohydrolase* | | *Glycosaminoglycan degradation* |  |
|  | | *Pyruvate-Formate Lyase* | | *Respiration* |  |
| 886 | | *Diaminopimelate epimerase* | | *Amino acid synthesis* |  |
|  | | *Alpha-Glucosidase* | | *Complex Carbohydrates* |  |
|  | | *Dihydrodipicolinate synthase* | | *Amino acid synthesis* |  |
| 887 | | *Ribokinase* | | *Feeder Pathways to Glycolysis* |  |
|  | | *Prephenate dehydrogenase* | | *Amino acid synthesis* |  |
|  | | *Si-Citrate Synthase* | | *Central Carbon Metabolism Pathways* |  |
| 888 | | *Geranyltranstransferase* | | *Isoprenoid biosynthesis* |  |
|  | | *Pyruvate-Formate Lyase* | | *Respiration* |  |
|  | | *Thymidine phosphorylase* | | *Pyrimidine metabolism* |  |
| 889 | | *Alanine racemase* | | *Amino acid synthesis* |  |
|  | | *Asparaginase* | | *Amino acid synthesis* |  |
|  | | *Pyruvate-Formate Lyase* | | *Respiration* |  |
| 890 | | *Cystathionine gamma-synthase* | | *Amino acid synthesis* |  |
|  | | *Prephenate dehydrogenase* | | *Amino acid synthesis* |  |
|  | | *Cytosine deaminase* | | *Pyrimidine metabolism* |  |
| 891 | | *Cystathionine gamma-synthase* | | *Amino acid synthesis* |  |
|  | | *L-alanine dehydrogenase* | | *Amino acid synthesis* |  |
|  | | *Pyruvate-Formate Lyase* | | *Respiration* |  |
| 892 | | *Ribokinase* | | *Feeder Pathways to Glycolysis* |  |
|  | | *Anthranilate synthase* | | *Amino acid transport and metabolism* |  |
|  | | *Pyruvate-Formate Lyase* | | *Respiration* |  |
| 893 | | *Prephenate dehydrogenase* | | *Amino acid synthesis* |  |
|  | | *Anaerobic ribonucleoside-triphosphate reductase* | | *Pyrimidine metabolism* |  |
|  | | *N-acetylmuramoyl-L-alanine amidase* | | *Glycan Biosynthesis and Metabolism* |  |
| 894 | | *Cysteine synthase A* | | *Amino acid synthesis* |  |
|  | | *Alpha-Glucosidase* | | *Complex Carbohydrates* |  |
|  | | *Prephenate dehydrogenase* | | *Amino acid synthesis* |  |
| 895 | | *IMP dehydrogenase* | | *Purine metabolism* |  |
|  | | *Diaminopimelate epimerase* | | *Amino acid synthesis* |  |
|  | | *N-acetylmuramoyl-L-alanine amidase* | | *Glycan Biosynthesis and Metabolism* |  |
| 896 | | *Diaminopimelate epimerase* | | *Amino acid synthesis* |  |
|  | | *Cysteine synthase A* | | *Amino acid synthesis* |  |
|  | | *Thioredoxin reductase FAD-NADP-binding* | | *Pyrimidine metabolism* |  |
| 897 | | *Acetylglutamate kinase* | | *Amino acid transport and metabolism* |  |
|  | | *Aspartate kinase* | | *Amino acid synthesis* |  |
|  | | *Diaminopimelate epimerase* | | *Amino acid synthesis* |  |
| 898 | | *Beta-D-galactosidase* | | *Glycan structures - degradation;Complex Carbohydrates* |  |
|  | | *Diaminopimelate epimerase* | | *Amino acid synthesis* |  |
|  | | *Dihydrodipicolinate synthase* | | *Amino acid synthesis* |  |
| 899 | | *N-acetylmuramoyl-L-alanine amidase* | | *Glycan Biosynthesis and Metabolism* |  |
|  | | *Beta-D-galactosidase* | | *Glycan structures - degradation;Complex Carbohydrates* |  |
|  | | *Ribokinase* | | *Feeder Pathways to Glycolysis* |  |
| 900 | | *Pyruvate-Formate Lyase* | | *Respiration* |  |
|  | | *IMP dehydrogenase* | | *Purine metabolism* |  |
|  | | *N-acetyl-D-galactosamine-4-sulfate 4-sulfohydrolase* | | *Glycosaminoglycan degradation* |  |
| 901 | | *Diaminopimelate epimerase* | | *Amino acid synthesis* |  |
|  | | *Pyruvate-Formate Lyase* | | *Respiration* |  |
|  | | *Beta-N-acetyl-D-hexosaminide N-acetylhexosaminohydrolase* | | *Glycosaminoglycan degradation* |  |
| 902 | | *Alpha-Glucosidase* | | *Complex Carbohydrates* |  |
|  | | *Diaminopimelate epimerase* | | *Amino acid synthesis* |  |
|  | | *Cytosine deaminase* | | *Pyrimidine metabolism* |  |
| 903 | | *N-Acetylglucosamine-6-Phosphate Deacetylase* | | *Feeder Pathways to Glycolysis* |  |
|  | | *Prephenate dehydrogenase* | | *Amino acid synthesis* |  |
|  | | *Ribokinase* | | *Feeder Pathways to Glycolysis* |  |
| 904 | | *N-acetyl-D-galactosamine-4-sulfate 4-sulfohydrolase* | | *Glycosaminoglycan degradation* |  |
|  | | *Pyruvate-Formate Lyase* | | *Respiration* |  |
|  | | *Beta-ketoacyl-acyl-carrier-protein synthase III* | | *Fatty Acid Biosynthesis* |  |
| 905 | | *Cytosine deaminase* | | *Pyrimidine metabolism* |  |
|  | | *UDP-N-acetylglucosamine acyltransferase* | | *Glycan Biosynthesis and Metabolism* |  |
|  | | *Pyruvate-Formate Lyase* | | *Respiration* |  |
| 906 | | *Methionyl-tRNA synthetase* | | *Amino acid synthesis* |  |
|  | | *Diaminopimelate epimerase* | | *Amino acid synthesis* |  |
|  | | *2-isopropylmalate synthase* | | *Amino acid synthesis* |  |
| 907 | | *Thioredoxin reductase FAD-NADP-binding* | | *Pyrimidine metabolism* |  |
|  | | *3-deoxy-7-phosphoheptulonate synthase* | | *Amino acid transport and metabolism* |  |
|  | | *Prephenate dehydrogenase* | | *Amino acid synthesis* |  |
| 908 | | *Cytosine deaminase* | | *Pyrimidine metabolism* |  |
|  | | *Pyruvate-Formate Lyase* | | *Respiration* |  |
|  | | *Ribokinase* | | *Feeder Pathways to Glycolysis* |  |
| 909 | | *Methionyl-tRNA synthetase* | | *Amino acid synthesis* |  |
|  | | *3-deoxy-7-phosphoheptulonate synthase* | | *Amino acid transport and metabolism* |  |
|  | | *Diaminopimelate epimerase* | | *Amino acid synthesis* |  |
| 910 | | *Thymidine phosphorylase* | | *Pyrimidine metabolism* |  |
|  | | *Pyruvate-Formate Lyase* | | *Respiration* |  |
|  | | *IMP dehydrogenase* | | *Purine metabolism* |  |
| 911 | | *phospho-N-acetylmuramoyl-pentapeptide transferase* | | *Glycan Biosynthesis and Metabolism* |  |
|  | | *Pyruvate-Formate Lyase* | | *Respiration* |  |
|  | | *Anthranilate synthase* | | *Amino acid transport and metabolism* |  |
| 912 | | *N-acetyl-D-galactosamine-4-sulfate 4-sulfohydrolase* | | *Glycosaminoglycan degradation* |  |
|  | | *Diaminopimelate epimerase* | | *Amino acid synthesis* |  |
|  | | *N-acetylglutamate synthase* | | *Amino acid transport and metabolism* |  |
| 913 | | *KDPG Aldolase* | | *Central Carbon Metabolism Pathways* |  |
|  | | *Pyruvate-Formate Lyase* | | *Respiration* |  |
|  | | *Carbon Monoxide Dehydrogenase* | | *Central Carbon Metabolism Pathways* |  |
| 914 | | *4-diphosphocytidyl-2C-methyl-D-erythritol synthase* | | *Isoprenoid biosynthesis* |  |
|  | | *Diaminopimelate epimerase* | | *Amino acid synthesis* |  |
|  | | *L-threonine aldolase* | | *Amino acid synthesis* |  |
| 915 | | *Beta-N-acetyl-D-hexosaminide N-acetylhexosaminohydrolase* | | *Glycosaminoglycan degradation* |  |
|  | | *Pyruvate-Formate Lyase* | | *Respiration* |  |
|  | | *Dihydroxy-acid dehydratase* | | *Amino acid transport and metabolism* |  |
| 916 | | *N-acetylmuramoyl-L-alanine amidase* | | *Glycan Biosynthesis and Metabolism* |  |
|  | | *N-acetylmuramoyl-L-alanine amidase* | | *Glycan Biosynthesis and Metabolism* |  |
|  | | *UDP-N-acetylmuramoyl-L-alanyl-D-glutamatemeso-diaminopimelate ligase* | | *Glycan Biosynthesis and Metabolism* |  |
| 917 | | *Quinolinate Synthase* | | *Cofactor Biosynthesis* |  |
|  | | *Carbon Monoxide Dehydrogenase* | | *Central Carbon Metabolism Pathways* |  |
|  | | *N-acetylmuramoyl-L-alanine amidase* | | *Glycan Biosynthesis and Metabolism* |  |
| 918 | | *Pyruvate-Formate Lyase* | | *Respiration* |  |
|  | | *Anthranilate synthase* | | *Amino acid transport and metabolism* |  |
|  | | *Si-Citrate Synthase* | | *Central Carbon Metabolism Pathways* |  |
| 919 | | *Folylpolyglutamate Synthase* | | *Cofactor Biosynthesis* |  |
|  | | *Conjugated Bile Salt Hydrolase* | | *Exotic Metabolisms* |  |
|  | | *Pyruvate-Formate Lyase* | | *Respiration* |  |
| 920 | | *Alpha-Glucosidase* | | *Complex Carbohydrates* |  |
|  | | *Ribokinase* | | *Feeder Pathways to Glycolysis* |  |
|  | | *Diaminopimelate epimerase* | | *Amino acid synthesis* |  |
| 921 | | *Prephenate dehydrogenase* | | *Amino acid synthesis* |  |
|  | | *Thymidine phosphorylase* | | *Pyrimidine metabolism* |  |
|  | | *Beta-D-galactosidase* | | *Glycan structures - degradation;Complex Carbohydrates* |  |
| 922 | | *Homoserine kinase* | | *Amino acid transport and metabolism* |  |
|  | | *Pyruvate-Formate Lyase* | | *Respiration* |  |
|  | | *N-Acetylglucosamine-6-Phosphate Deacetylase* | | *Feeder Pathways to Glycolysis* |  |
| 923 | | *Methylmalonyl-CaA decarboxylase* | | *Organic Acids* |  |
|  | | *Pyruvate-Formate Lyase* | | *Respiration* |  |
|  | | *3-deoxy-7-phosphoheptulonate synthase* | | *Amino acid transport and metabolism* |  |
| 924 | | *Prephenate dehydrogenase* | | *Amino acid synthesis* |  |
|  | | *L-glutamine synthase* | | *Amino acid synthesis* |  |
|  | | *Adenylosuccinate synthetase* | | *Purine metabolism* |  |
| 925 | | *N-acetylmuramoyl-L-alanine amidase* | | *Glycan Biosynthesis and Metabolism* |  |
|  | | *Glucuronate Isomerase* | | *Feeder Pathways to Glycolysis* |  |
|  | | *Pyruvate-Formate Lyase* | | *Respiration* |  |
| 926 | | *Thymidine phosphorylase* | | *Pyrimidine metabolism* |  |
|  | | *Alanine racemase* | | *Amino acid synthesis* |  |
|  | | *Diaminopimelate epimerase* | | *Amino acid synthesis* |  |
| 927 | | *Prephenate dehydrogenase* | | *Amino acid synthesis* |  |
|  | | *Cysteine synthase A* | | *Amino acid synthesis* |  |
|  | | *Ornithine carbamoyltransferase 1* | | *Amino acid transport and metabolism* |  |
| 928 | | *Beta-N-acetyl-D-hexosaminide N-acetylhexosaminohydrolase* | | *Glycosaminoglycan degradation* |  |
|  | | *4-diphosphocytidyl-2C-methyl-D-erythritol synthase* | | *Isoprenoid biosynthesis* |  |
|  | | *Prephenate dehydrogenase* | | *Amino acid synthesis* |  |
| 929 | | *N-acetylglutamate synthase* | | *Amino acid transport and metabolism* |  |
|  | | *Cytidylate kinase* | | *Pyrimidine metabolism* |  |
|  | | *Prephenate dehydrogenase* | | *Amino acid synthesis* |  |
| 930 | | *Biotin Synthase* | | *Cofactor Biosynthesis* |  |
|  | | *Phosphogluconate dehydratase* | | *Central Carbon Metabolism Pathways* |  |
|  | | *Pyruvate-Formate Lyase* | | *Respiration* |  |
| 931 | | *Prephenate dehydrogenase* | | *Amino acid synthesis* |  |
|  | | *N-Acetylglucosamine-6-Phosphate Deacetylase* | | *Feeder Pathways to Glycolysis* |  |
|  | | *Methionyl-tRNA synthetase* | | *Amino acid synthesis* |  |
| 932 | | *Prephenate dehydrogenase* | | *Amino acid synthesis* |  |
|  | | *Thioredoxin reductase FAD-NADP-binding* | | *Pyrimidine metabolism* |  |
|  | | *Alpha-Glucosidase* | | *Complex Carbohydrates* |  |
| 933 | | *Diaminopimelate epimerase* | | *Amino acid synthesis* |  |
|  | | *Galactokinase* | | *Feeder Pathways to Glycolysis* |  |
|  | | *Beta-D-galactosidase* | | *Glycan structures - degradation;Complex Carbohydrates* |  |
| 934 | | *Pyruvate-Formate Lyase* | | *Respiration* |  |
|  | | *Beta-D-galactosidase* | | *Glycan structures - degradation;Complex Carbohydrates* |  |
|  | | *ATP phosphoribosyltransferase* | | *Amino acid transport and metabolism* |  |
| 935 | | *Prephenate dehydrogenase* | | *Amino acid synthesis* |  |
|  | | *Glycerol Kinase* | | *Glycerolipid Metabolism* |  |
|  | | *Anthranilate synthase* | | *Amino acid transport and metabolism* |  |
| 936 | | *Anthranilate synthase* | | *Amino acid transport and metabolism* |  |
|  | | *Cystathionine gamma-synthase* | | *Amino acid synthesis* |  |
|  | | *Diaminopimelate epimerase* | | *Amino acid synthesis* |  |
| 937 | | *Glutamate synthase large and small subunit (NADPH)* | | *Amino acid synthesis* |  |
|  | | *Biotin Synthase* | | *Cofactor Biosynthesis* |  |
|  | | *Pyruvate-Formate Lyase* | | *Respiration* |  |
| 938 | | *Xylose Isomerase* | | *Feeder Pathways to Glycolysis* |  |
|  | | *Cytidylate kinase* | | *Pyrimidine metabolism* |  |
|  | | *Pyruvate-Formate Lyase* | | *Respiration* |  |
| 939 | | *N-acetylmuramoyl-L-alanine amidase* | | *Glycan Biosynthesis and Metabolism* |  |
|  | | *UDP-N-acetylmuramoyl-L-alanineD-glutamate ligase* | | *Glycan Biosynthesis and Metabolism* |  |
|  | | *Thioredoxin reductase FAD-NADP-binding* | | *Pyrimidine metabolism* |  |
| 940 | | *Prephenate dehydrogenase* | | *Amino acid synthesis* |  |
|  | | *Beta-D-galactosidase* | | *Glycan structures - degradation;Complex Carbohydrates* |  |
|  | | *Chorismate synthase* | | *Amino acid transport and metabolism* |  |
| 941 | | *Dihydrodipicolinate synthase* | | *Amino acid synthesis* |  |
|  | | *Glutamate racemase* | | *Amino acid synthesis* |  |
|  | | *Diaminopimelate epimerase* | | *Amino acid synthesis* |  |
| 942 | | *N-Acetylglucosamine-6-Phosphate Deacetylase* | | *Feeder Pathways to Glycolysis* |  |
|  | | *Diaminopimelate epimerase* | | *Amino acid synthesis* |  |
|  | | *Cysteine synthase A* | | *Amino acid synthesis* |  |
| 943 | | *Pyruvate-Formate Lyase* | | *Respiration* |  |
|  | | *ADP-ribose pyrophosphatase* | | *Purine metabolism* |  |
|  | | *Uridine phosphorylase* | | *Pyrimidine metabolism* |  |
| 944 | | *Butyrate Kinase* | | *Organic Acids* |  |
|  | | *N-acetylmuramoyl-L-alanine amidase* | | *Glycan Biosynthesis and Metabolism* |  |
|  | | *UDP-N-acetylmuramateL-alanine ligase* | | *Glycan Biosynthesis and Metabolism* |  |
| 945 | | *Serine O-acetyltransferase* | | *Amino acid synthesis* |  |
|  | | *Diaminopimelate epimerase* | | *Amino acid synthesis* |  |
|  | | *Alpha-Glucosidase* | | *Complex Carbohydrates* |  |
| 946 | | *Pyridoxal Kinase* | | *Cofactor Biosynthesis* |  |
|  | | *Serine hydroxymethyltransferase* | | *Amino acid synthesis* |  |
|  | | *Prephenate dehydrogenase* | | *Amino acid synthesis* |  |
| 947 | | *Prephenate dehydrogenase* | | *Amino acid synthesis* |  |
|  | | *Acetyl-CoA acyltransferase anaerobic* | | *Fatty Acid Metabolism* |  |
|  | | *Ornithine carbamoyltransferase 1* | | *Amino acid transport and metabolism* |  |
| 948 | | *Pyruvate-Formate Lyase* | | *Respiration* |  |
|  | | *3-deoxy-7-phosphoheptulonate synthase* | | *Amino acid transport and metabolism* |  |
|  | | *Anthranilate synthase* | | *Amino acid transport and metabolism* |  |
| 949 | | *3-deoxy-7-phosphoheptulonate synthase* | | *Amino acid transport and metabolism* |  |
|  | | *N-acetylmuramoyl-L-alanine amidase* | | *Glycan Biosynthesis and Metabolism* |  |
|  | | *Beta-ketoacyl-acyl-carrier-protein synthase III* | | *Fatty Acid Biosynthesis* |  |
| 950 | | *Cytosine deaminase* | | *Pyrimidine metabolism* |  |
|  | | *Dihydrodipicolinate synthase* | | *Amino acid synthesis* |  |
|  | | *Diaminopimelate epimerase* | | *Amino acid synthesis* |  |
| 951 | | *Pyridoxal Kinase* | | *Cofactor Biosynthesis* |  |
|  | | *Pyruvate-Formate Lyase* | | *Respiration* |  |
|  | | *Uridine phosphorylase* | | *Pyrimidine metabolism* |  |
| 952 | | *Pyruvate-Formate Lyase* | | *Respiration* |  |
|  | | *Xylose Isomerase* | | *Feeder Pathways to Glycolysis* |  |
|  | | *N-acetyl-D-galactosamine-4-sulfate 4-sulfohydrolase* | | *Glycosaminoglycan degradation* |  |
| 953 | | *Dihydrodipicolinate synthase* | | *Amino acid synthesis* |  |
|  | | *Carbon Monoxide Dehydrogenase* | | *Central Carbon Metabolism Pathways* |  |
|  | | *Prephenate dehydrogenase* | | *Amino acid synthesis* |  |
| 954 | | *N-acetyl-D-galactosamine-4-sulfate 4-sulfohydrolase* | | *Glycosaminoglycan degradation* |  |
|  | | *Beta-D-galactosidase* | | *Glycan structures - degradation;Complex Carbohydrates* |  |
|  | | *Prephenate dehydrogenase* | | *Amino acid synthesis* |  |
| 955 | | *Beta-N-acetyl-D-hexosaminide N-acetylhexosaminohydrolase* | | *Glycosaminoglycan degradation* |  |
|  | | *Dihydrodipicolinate synthase* | | *Amino acid synthesis* |  |
|  | | *N-acetylmuramoyl-L-alanine amidase* | | *Glycan Biosynthesis and Metabolism* |  |
| 956 | | *Threonine ammonia-lyase* | | *Amino acid synthesis* |  |
|  | | *N-acetylmuramoyl-L-alanine amidase* | | *Glycan Biosynthesis and Metabolism* |  |
|  | | *UDP-N-acetylmuramoyl-L-alanyl-D-glutamatemeso-diaminopimelate ligase* | | *Glycan Biosynthesis and Metabolism* |  |
| 957 | | *Carbon Monoxide Dehydrogenase* | | *Central Carbon Metabolism Pathways* |  |
|  | | *Uridine phosphorylase* | | *Pyrimidine metabolism* |  |
|  | | *N-acetylmuramoyl-L-alanine amidase* | | *Glycan Biosynthesis and Metabolism* |  |
| 958 | | *Beta-D-galactosidase* | | *Glycan structures - degradation;Complex Carbohydrates* |  |
|  | | *Thymidine phosphorylase* | | *Pyrimidine metabolism* |  |
|  | | *Prephenate dehydrogenase* | | *Amino acid synthesis* |  |
| 959 | | *Prephenate dehydrogenase* | | *Amino acid synthesis* |  |
|  | | *Beta-D-galactosidase* | | *Glycan structures - degradation;Complex Carbohydrates* |  |
|  | | *Dihydrodipicolinate synthase* | | *Amino acid synthesis* |  |
| 960 | | *Acetyl-CoA acyltransferase anaerobic* | | *Fatty Acid Metabolism* |  |
|  | | *Cytosine deaminase* | | *Pyrimidine metabolism* |  |
|  | | *Pyruvate-Formate Lyase* | | *Respiration* |  |
| 961 | | *Prephenate dehydrogenase* | | *Amino acid synthesis* |  |
|  | | *Beta-ketoacyl-acyl-carrier-protein synthase III* | | *Fatty Acid Biosynthesis* |  |
|  | | *ATP phosphoribosyltransferase* | | *Amino acid transport and metabolism* |  |
| 962 | | *Cysteine synthase A* | | *Amino acid synthesis* |  |
|  | | *Branched-chain-amino-acid transaminase* | | *Amino acid transport and metabolism* |  |
|  | | *Prephenate dehydrogenase* | | *Amino acid synthesis* |  |
| 963 | | *Prephenate dehydrogenase* | | *Amino acid synthesis* |  |
|  | | *Pyruvate-Formate Lyase* | | *Respiration* |  |
|  | | *Cytidylate kinase* | | *Pyrimidine metabolism* |  |
| 964 | | *Glutamate decarboxylase A and B PLP-dependent* | | *Amino acid transport and metabolism* |  |
|  | | *Diaminopimelate epimerase* | | *Amino acid synthesis* |  |
|  | | *3-isopropylmalate dehydrogenase* | | *Amino acid synthesis* |  |
| 965 | | *Butyrate Kinase* | | *Organic Acids* |  |
|  | | *Pyruvate-Formate Lyase* | | *Respiration* |  |
|  | | *Ornithine carbamoyltransferase 1* | | *Amino acid transport and metabolism* |  |
| 966 | | *Anthranilate synthase* | | *Amino acid transport and metabolism* |  |
|  | | *N-acetyl-D-galactosamine-4-sulfate 4-sulfohydrolase* | | *Glycosaminoglycan degradation* |  |
|  | | *Prephenate dehydrogenase* | | *Amino acid synthesis* |  |
| 967 | | *Pyruvate-Formate Lyase* | | *Respiration* |  |
|  | | *Carbon Monoxide Dehydrogenase* | | *Central Carbon Metabolism Pathways* |  |
|  | | *Gamma-glutamyl kinase* | | *Amino acid transport and metabolism* |  |
| 968 | | *Beta-D-galactosidase* | | *Glycan structures - degradation;Complex Carbohydrates* |  |
|  | | *UDP-N-acetylmuramoyl-L-alanineD-glutamate ligase* | | *Glycan Biosynthesis and Metabolism* |  |
|  | | *N-acetylmuramoyl-L-alanine amidase* | | *Glycan Biosynthesis and Metabolism* |  |
| 969 | | *UDP-N-acetylmuramateL-alanine ligase* | | *Glycan Biosynthesis and Metabolism* |  |
|  | | *Mannose-6-Phosphate Isomerase* | | *Feeder Pathways to Glycolysis* |  |
|  | | *Diaminopimelate epimerase* | | *Amino acid synthesis* |  |
| 970 | | *Alpha-Glucosidase* | | *Complex Carbohydrates* |  |
|  | | *N-acetylmuramoyl-L-alanine amidase* | | *Glycan Biosynthesis and Metabolism* |  |
|  | | *D-alanineD-alanine ligase* | | *Glycan Biosynthesis and Metabolism* |  |
| 971 | | *UDP-N-acetylmuramateL-alanine ligase* | | *Glycan Biosynthesis and Metabolism* |  |
|  | | *Prephenate dehydrogenase* | | *Amino acid synthesis* |  |
|  | | *Chorismate synthase* | | *Amino acid transport and metabolism* |  |
| 972 | | *N-acetylmuramoyl-L-alanine amidase* | | *Glycan Biosynthesis and Metabolism* |  |
|  | | *N-acetyl-D-galactosamine-4-sulfate 4-sulfohydrolase* | | *Glycosaminoglycan degradation* |  |
|  | | *Anaerobic ribonucleoside-triphosphate reductase* | | *Pyrimidine metabolism* |  |
| 973 | | *Carbon Monoxide Dehydrogenase* | | *Central Carbon Metabolism Pathways* |  |
|  | | *Pyruvate-Formate Lyase* | | *Respiration* |  |
|  | | *Histidinol dehydrogenase* | | *Amino acid transport and metabolism* |  |
| 974 | | *Cysteine synthase A* | | *Amino acid synthesis* |  |
|  | | *Valine-pyruvate aminotransferase* | | *Amino acid synthesis* |  |
|  | | *Prephenate dehydrogenase* | | *Amino acid synthesis* |  |
| 975 | | *Acetylglutamate kinase* | | *Amino acid transport and metabolism* |  |
|  | | *Glutamate racemase* | | *Amino acid synthesis* |  |
|  | | *Prephenate dehydrogenase* | | *Amino acid synthesis* |  |
| 976 | | *Ribokinase* | | *Feeder Pathways to Glycolysis* |  |
|  | | *3-demethylubiquinone-9 3-methyltransferase* | | *Cofactor Biosynthesis* |  |
|  | | *Diaminopimelate epimerase* | | *Amino acid synthesis* |  |
| 977 | | *Cytosine deaminase* | | *Pyrimidine metabolism* |  |
|  | | *L-alanine dehydrogenase* | | *Amino acid synthesis* |  |
|  | | *Diaminopimelate epimerase* | | *Amino acid synthesis* |  |
| 978 | | *Pyruvate-Formate Lyase* | | *Respiration* |  |
|  | | *Cytosine deaminase* | | *Pyrimidine metabolism* |  |
|  | | *Pyruvate-Formate Lyase* | | *Respiration* |  |
| 979 | | *Prephenate dehydrogenase* | | *Amino acid synthesis* |  |
|  | | *Formyltetrahydrofolate synthetase* | | *Organic Acids* |  |
|  | | *Cysteine synthase A* | | *Amino acid synthesis* |  |
| 980 | | *3-deoxy-7-phosphoheptulonate synthase* | | *Amino acid transport and metabolism* |  |
|  | | *Carbon Monoxide Dehydrogenase* | | *Central Carbon Metabolism Pathways* |  |
|  | | *Prephenate dehydrogenase* | | *Amino acid synthesis* |  |
| 981 | | *Diaminopimelate epimerase* | | *Amino acid synthesis* |  |
|  | | *N-Acetylglucosamine-6-Phosphate Deacetylase* | | *Feeder Pathways to Glycolysis* |  |
|  | | *Ribokinase* | | *Feeder Pathways to Glycolysis* |  |
| 982 | | *Pantothenate Synthetase* | | *Cofactor Biosynthesis* |  |
|  | | *Cystathionine gamma-synthase* | | *Amino acid synthesis* |  |
|  | | *Diaminopimelate epimerase* | | *Amino acid synthesis* |  |
| 983 | | *Geranyltranstransferase* | | *Isoprenoid biosynthesis* |  |
|  | | *Prephenate dehydrogenase* | | *Amino acid synthesis* |  |
|  | | *N-Acetylglucosamine-6-Phosphate Deacetylase* | | *Feeder Pathways to Glycolysis* |  |
| 984 | | *Alanine racemase* | | *Amino acid synthesis* |  |
|  | | *Diaminopimelate epimerase* | | *Amino acid synthesis* |  |
|  | | *Butyrate Kinase* | | *Organic Acids* |  |
| 985 | | *Galactokinase* | | *Feeder Pathways to Glycolysis* |  |
|  | | *Diaminopimelate epimerase* | | *Amino acid synthesis* |  |
|  | | *Dihydrodipicolinate synthase* | | *Amino acid synthesis* |  |
| 986 | | *Butyrate Kinase* | | *Organic Acids* |  |
|  | | *Diaminopimelate epimerase* | | *Amino acid synthesis* |  |
|  | | *Methylmalonyl-CaA decarboxylase* | | *Organic Acids* |  |
| 987 | | *Methylmalonyl-CaA decarboxylase* | | *Organic Acids* |  |
|  | | *Argininosuccinate lyase* | | *Amino acid transport and metabolism* |  |
|  | | *Diaminopimelate epimerase* | | *Amino acid synthesis* |  |
| 988 | | *Argininosuccinate lyase* | | *Amino acid transport and metabolism* |  |
|  | | *Ribokinase* | | *Feeder Pathways to Glycolysis* |  |
|  | | *Diaminopimelate epimerase* | | *Amino acid synthesis* |  |
| 989 | | *N-acetyl-D-galactosamine-4-sulfate 4-sulfohydrolase* | | *Glycosaminoglycan degradation* |  |
|  | | *Butyrate Kinase* | | *Organic Acids* |  |
|  | | *N-acetylmuramoyl-L-alanine amidase* | | *Glycan Biosynthesis and Metabolism* |  |
| 990 | | *Diaminopimelate epimerase* | | *Amino acid synthesis* |  |
|  | | *UDP-N-acetylmuramateL-alanine ligase* | | *Glycan Biosynthesis and Metabolism* |  |
|  | | *Acetyl-CoA acyltransferase anaerobic* | | *Fatty Acid Metabolism* |  |
| 991 | | *Galactokinase* | | *Feeder Pathways to Glycolysis* |  |
|  | | *Cystathionine gamma-synthase* | | *Amino acid synthesis* |  |
|  | | *Diaminopimelate epimerase* | | *Amino acid synthesis* |  |
| 992 | | *Beta-N-acetyl-D-hexosaminide N-acetylhexosaminohydrolase* | | *Glycosaminoglycan degradation* |  |
|  | | *N-acetylmuramoyl-L-alanine amidase* | | *Glycan Biosynthesis and Metabolism* |  |
|  | | *Fucose Isomerase* | | *Feeder Pathways to Glycolysis* |  |
| 993 | | *Pyruvate-Formate Lyase* | | *Respiration* |  |
|  | | *Thymidine phosphorylase* | | *Pyrimidine metabolism* |  |
|  | | *Glutamate synthase large and small subunit (NADPH)* | | *Amino acid synthesis* |  |
| 994 | | *Diaminopimelate epimerase* | | *Amino acid synthesis* |  |
|  | | *Si-Citrate Synthase* | | *Central Carbon Metabolism Pathways* |  |
|  | | *Agamintase* | | *Nitrogen Metabolism* |  |
| 995 | | *Alpha-Glucosidase* | | *Complex Carbohydrates* |  |
|  | | *Cysteine synthase A* | | *Amino acid synthesis* |  |
|  | | *Diaminopimelate epimerase* | | *Amino acid synthesis* |  |
| 996 | | *Alpha-mannosidase* | | *N-Glycan degradation* |  |
|  | | *Pyruvate-Formate Lyase* | | *Respiration* |  |
|  | | *Anaerobic ribonucleoside-triphosphate reductase* | | *Pyrimidine metabolism* |  |
| 997 | | *N-Acetylglucosamine-6-Phosphate Deacetylase* | | *Feeder Pathways to Glycolysis* |  |
|  | | *Diaminopimelate epimerase* | | *Amino acid synthesis* |  |
|  | | *Ornithine carbamoyltransferase 1* | | *Amino acid transport and metabolism* |  |
| 998 | | *Cystathionine gamma-synthase* | | *Amino acid synthesis* |  |
|  | | *Beta-D-glucuronidase* | | *Glycan structures - degradation;Exotic Metabolisms* |  |
|  | | *Diaminopimelate epimerase* | | *Amino acid synthesis* |  |
| 999 | | *Ribokinase* | | *Feeder Pathways to Glycolysis* |  |
|  | | *Pyruvate-Formate Lyase* | | *Respiration* |  |
|  | | *Cytosine deaminase* | | *Pyrimidine metabolism* |  |
| 1000 | | *L-alanine dehydrogenase* | | *Amino acid synthesis* |  |
|  | | *Alpha-mannosidase* | | *N-Glycan degradation* |  |
|  | | *Pyruvate-Formate Lyase* | | *Respiration* |  |
| 1001 | | *Glycerol Kinase* | | *Glycerolipid Metabolism* |  |
|  | | *Prephenate dehydrogenase* | | *Amino acid synthesis* |  |
|  | | *Fucose Isomerase* | | *Feeder Pathways to Glycolysis* |  |
| 1002 | | *Diaminopimelate epimerase* | | *Amino acid synthesis* |  |
|  | | *Formyltetrahydrofolate synthetase* | | *Organic Acids* |  |
|  | | *Pyrroline-5-carboxylate reductase* | | *Amino acid transport and metabolism* |  |
| 1003 | | *Dihydrodipicolinate synthase* | | *Amino acid synthesis* |  |
|  | | *Cystathionine gamma-synthase* | | *Amino acid synthesis* |  |
|  | | *Uridine phosphorylase* | | *Pyrimidine metabolism* |  |
| 1004 | | *IMP dehydrogenase* | | *Purine metabolism* |  |
|  | | *Pyruvate-Formate Lyase* | | *Respiration* |  |
|  | | *Cysteine synthase A* | | *Amino acid synthesis* |  |
| 1005 | | *Alpha-Glucosidase* | | *Complex Carbohydrates* |  |
|  | | *Pyruvate-Formate Lyase* | | *Respiration* |  |
|  | | *Alanine racemase* | | *Amino acid synthesis* |  |
| 1006 | | *Diaminopimelate epimerase* | | *Amino acid synthesis* |  |
|  | | *Beta-D-glucuronidase* | | *Glycan structures - degradation;Exotic Metabolisms* |  |
|  | | *N-acetylmuramoyl-L-alanine amidase* | | *Glycan Biosynthesis and Metabolism* |  |
| 1007 | | *Diaminopimelate epimerase* | | *Amino acid synthesis* |  |
|  | | *Ribokinase* | | *Feeder Pathways to Glycolysis* |  |
|  | | *Thioredoxin reductase FAD-NADP-binding* | | *Pyrimidine metabolism* |  |
| 1008 | | *L-Lactate Dehydrogenase* | | *Organic Acids* |  |
|  | | *Diaminopimelate epimerase* | | *Amino acid synthesis* |  |
|  | | *Histidinol dehydrogenase* | | *Amino acid transport and metabolism* |  |
| 1009 | | *Pyruvate-Formate Lyase* | | *Respiration* |  |
|  | | *phospho-N-acetylmuramoyl-pentapeptide transferase* | | *Glycan Biosynthesis and Metabolism* |  |
|  | | *Beta-D-galactosidase* | | *Glycan structures - degradation;Complex Carbohydrates* |  |
| 1010 | | *Pyruvate-Formate Lyase* | | *Respiration* |  |
|  | | *Thymidine phosphorylase* | | *Pyrimidine metabolism* |  |
|  | | *Dihydroxy-acid dehydratase* | | *Amino acid transport and metabolism* |  |
| 1011 | | *UDP-N-acetylmuramateL-alanine ligase* | | *Glycan Biosynthesis and Metabolism* |  |
|  | | *Agamintase* | | *Nitrogen Metabolism* |  |
|  | | *Pyruvate-Formate Lyase* | | *Respiration* |  |
| 1012 | | *Glucuronate Isomerase* | | *Feeder Pathways to Glycolysis* |  |
|  | | *Prephenate dehydrogenase* | | *Amino acid synthesis* |  |
|  | | *Aspartate-ammonia ligase* | | *Amino acid synthesis* |  |
| 1013 | | *Folylpolyglutamate Synthase* | | *Cofactor Biosynthesis* |  |
|  | | *Diaminopimelate epimerase* | | *Amino acid synthesis* |  |
|  | | *Thymidine phosphorylase* | | *Pyrimidine metabolism* |  |
| 1014 | | *Histidinol dehydrogenase* | | *Amino acid transport and metabolism* |  |
|  | | *Pyridoxal Kinase* | | *Cofactor Biosynthesis* |  |
|  | | *Diaminopimelate epimerase* | | *Amino acid synthesis* |  |
| 1015 | | *Thioredoxin reductase FAD-NADP-binding* | | *Pyrimidine metabolism* |  |
|  | | *N-acetylmuramoyl-L-alanine amidase* | | *Glycan Biosynthesis and Metabolism* |  |
|  | | *Beta-ketoacyl-acyl-carrier-protein synthase III* | | *Fatty Acid Biosynthesis* |  |
| 1016 | | *Glycerol Kinase* | | *Glycerolipid Metabolism* |  |
|  | | *Diaminopimelate epimerase* | | *Amino acid synthesis* |  |
|  | | *Conjugated Bile Salt Hydrolase* | | *Exotic Metabolisms* |  |
| 1017 | | *Methionyl-tRNA synthetase* | | *Amino acid synthesis* |  |
|  | | *Cysteine synthase A* | | *Amino acid synthesis* |  |
|  | | *N-acetylmuramoyl-L-alanine amidase* | | *Glycan Biosynthesis and Metabolism* |  |
| 1018 | | *Cysteine synthase A* | | *Amino acid synthesis* |  |
|  | | *Prephenate dehydrogenase* | | *Amino acid synthesis* |  |
|  | | *Beta-N-acetyl-D-hexosaminide N-acetylhexosaminohydrolase* | | *Glycosaminoglycan degradation* |  |
| 1019 | | *Pyruvate-Formate Lyase* | | *Respiration* |  |
|  | | *Chorismate synthase* | | *Amino acid transport and metabolism* |  |
|  | | *Pyruvate-Formate Lyase* | | *Respiration* |  |
| 1020 | | *Pyruvate-Formate Lyase* | | *Respiration* |  |
|  | | *Butyrate Kinase* | | *Organic Acids* |  |
|  | | *Mannose-6-Phosphate Isomerase* | | *Feeder Pathways to Glycolysis* |  |
| 1021 | | *Acetyl-CoA acyltransferase anaerobic* | | *Fatty Acid Metabolism* |  |
|  | | *N-acetyl-D-glucosamine-6-sulfate 6-sulfohydrolase* | | *Glycosaminoglycan degradation* |  |
|  | | *Prephenate dehydrogenase* | | *Amino acid synthesis* |  |
| 1022 | | *1-hydroxy-2-methyl-2-E-butenyl 4-diphosphate reductase 4Fe-4S protein* | | *Isoprenoid biosynthesis* |  |
|  | | *N-acetylmuramoyl-L-alanine amidase* | | *Glycan Biosynthesis and Metabolism* |  |
|  | | *L-Lactate Dehydrogenase* | | *Organic Acids* |  |
| 1023 | | *Pyruvate-Formate Lyase* | | *Respiration* |  |
|  | | *Xylose Isomerase* | | *Feeder Pathways to Glycolysis* |  |
|  | | *Uridine phosphorylase* | | *Pyrimidine metabolism* |  |
| 1024 | | *Beta-D-galactosidase* | | *Glycan structures - degradation;Complex Carbohydrates* |  |
|  | | *Pyruvate-Formate Lyase* | | *Respiration* |  |
|  | | *Quinolinate Synthase* | | *Cofactor Biosynthesis* |  |
| 1025 | | *N-acetylmuramoyl-L-alanine amidase* | | *Glycan Biosynthesis and Metabolism* |  |
|  | | *Ornithine carbamoyltransferase 1* | | *Amino acid transport and metabolism* |  |
|  | | *Uridine phosphorylase* | | *Pyrimidine metabolism* |  |
| 1026 | | *Biosynthetic arginine decarboxylase PLP-binding* | | *Amino acid transport and metabolism* |  |
|  | | *Cytidylate kinase* | | *Pyrimidine metabolism* |  |
|  | | *Prephenate dehydrogenase* | | *Amino acid synthesis* |  |
| 1027 | | *Diaminopimelate epimerase* | | *Amino acid synthesis* |  |
|  | | *Acetyl-CoA acyltransferase anaerobic* | | *Fatty Acid Metabolism* |  |
|  | | *N-Acetylglucosamine-6-Phosphate Deacetylase* | | *Feeder Pathways to Glycolysis* |  |
| 1028 | | *Lysine decarboxylase 1* | | *Amino acid transport and metabolism* |  |
|  | | *Anthranilate synthase* | | *Amino acid transport and metabolism* |  |
|  | | *Diaminopimelate epimerase* | | *Amino acid synthesis* |  |
| 1029 | | *Butyrate Kinase* | | *Organic Acids* |  |
|  | | *N-acetylmuramoyl-L-alanine amidase* | | *Glycan Biosynthesis and Metabolism* |  |
|  | | *Beta-ketoacyl-acyl-carrier-protein synthase III* | | *Fatty Acid Biosynthesis* |  |
| 1030 | | *Prephenate dehydrogenase* | | *Amino acid synthesis* |  |
|  | | *Argininosuccinate lyase* | | *Amino acid transport and metabolism* |  |
|  | | *Folylpolyglutamate Synthase* | | *Cofactor Biosynthesis* |  |
| 1031 | | *Riboflavin Synthase α Subunit* | | *Cofactor Biosynthesis* |  |
|  | | *Diaminopimelate epimerase* | | *Amino acid synthesis* |  |
|  | | *Serine O-acetyltransferase* | | *Amino acid synthesis* |  |
| 1032 | | *Aspartate kinase* | | *Amino acid synthesis* |  |
|  | | *Diaminopimelate epimerase* | | *Amino acid synthesis* |  |
|  | | *Adenylosuccinate synthetase* | | *Purine metabolism* |  |
| 1033 | | *Prephenate dehydrogenase* | | *Amino acid synthesis* |  |
|  | | *Carbamoyl phosphate synthetase small subunit glutamine amidotransferase* | | *Pyrimidine metabolism* |  |
|  | | *Selenocysteine synthase* | | *Amino acid synthesis;Amino acid synthesis* |  |
| 1034 | | *Glutamate racemase* | | *Amino acid synthesis* |  |
|  | | *Prephenate dehydrogenase* | | *Amino acid synthesis* |  |
|  | | *L-Lactate Dehydrogenase* | | *Organic Acids* |  |
| 1035 | | *Dihydrodipicolinate synthase* | | *Amino acid synthesis* |  |
|  | | *1-Phosphofructokinase* | | *Feeder Pathways to Glycolysis* |  |
|  | | *Diaminopimelate epimerase* | | *Amino acid synthesis* |  |
| 1036 | | *Folylpolyglutamate Synthase* | | *Cofactor Biosynthesis* |  |
|  | | *Alpha-Glucosidase* | | *Complex Carbohydrates* |  |
|  | | *Pyruvate-Formate Lyase* | | *Respiration* |  |
| 1037 | | *Diaminopimelate epimerase* | | *Amino acid synthesis* |  |
|  | | *phospho-N-acetylmuramoyl-pentapeptide transferase* | | *Glycan Biosynthesis and Metabolism* |  |
|  | | *Asparaginase* | | *Amino acid synthesis* |  |
| 1038 | | *Prephenate dehydrogenase* | | *Amino acid synthesis* |  |
|  | | *Glutamate racemase* | | *Amino acid synthesis* |  |
|  | | *N-acetyl-D-glucosamine-6-sulfate 6-sulfohydrolase* | | *Glycosaminoglycan degradation* |  |
| 1039 | | *Pyruvate-Formate Lyase* | | *Respiration* |  |
|  | | *Ribokinase* | | *Feeder Pathways to Glycolysis* |  |
|  | | *Selenocysteine synthase* | | *Amino acid synthesis;Amino acid synthesis* |  |
| 1040 | | *Diaminopimelate epimerase* | | *Amino acid synthesis* |  |
|  | | *4-diphosphocytidyl-2C-methyl-D-erythritol synthase* | | *Isoprenoid biosynthesis* |  |
|  | | *UDP-N-acetylmuramateL-alanine ligase* | | *Glycan Biosynthesis and Metabolism* |  |
| 1041 | | *IMP dehydrogenase* | | *Purine metabolism* |  |
|  | | *Shikimate kinase I II* | | *Amino acid transport and metabolism* |  |
|  | | *Pyruvate-Formate Lyase* | | *Respiration* |  |
| 1042 | | *Ribokinase* | | *Feeder Pathways to Glycolysis* |  |
|  | | *3-isopropylmalate dehydrogenase* | | *Amino acid synthesis* |  |
|  | | *Prephenate dehydrogenase* | | *Amino acid synthesis* |  |
| 1043 | | *Prephenate dehydrogenase* | | *Amino acid synthesis* |  |
|  | | *Anthranilate synthase* | | *Amino acid transport and metabolism* |  |
|  | | *Beta-D-galactosidase* | | *Glycan structures - degradation;Complex Carbohydrates* |  |
| 1044 | | *Dihydroxy-acid dehydratase* | | *Amino acid transport and metabolism* |  |
|  | | *Galactokinase* | | *Feeder Pathways to Glycolysis* |  |
|  | | *Diaminopimelate epimerase* | | *Amino acid synthesis* |  |
| 1045 | | *Alanine racemase* | | *Amino acid synthesis* |  |
|  | | *Uridine phosphorylase* | | *Pyrimidine metabolism* |  |
|  | | *N-acetylmuramoyl-L-alanine amidase* | | *Glycan Biosynthesis and Metabolism* |  |
| 1046 | | *Diaminopimelate epimerase* | | *Amino acid synthesis* |  |
|  | | *Alpha-Glucosidase* | | *Complex Carbohydrates* |  |
|  | | *Acetyl-CoA acyltransferase anaerobic* | | *Fatty Acid Metabolism* |  |
| 1047 | | *Dihydroxy-acid dehydratase* | | *Amino acid transport and metabolism* |  |
|  | | *Diaminopimelate epimerase* | | *Amino acid synthesis* |  |
|  | | *Histidinol dehydrogenase* | | *Amino acid transport and metabolism* |  |
| 1048 | | *Diaminopimelate epimerase* | | *Amino acid synthesis* |  |
|  | | *Dihydrodipicolinate synthase* | | *Amino acid synthesis* |  |
|  | | *IMP dehydrogenase* | | *Purine metabolism* |  |
| 1049 | | *Alpha-Glucosidase* | | *Complex Carbohydrates* |  |
|  | | *Acetyl-CoA acyltransferase anaerobic* | | *Fatty Acid Metabolism* |  |
|  | | *Diaminopimelate epimerase* | | *Amino acid synthesis* |  |
| 1050 | | *D-alanineD-alanine ligase* | | *Glycan Biosynthesis and Metabolism* |  |
|  | | *Diaminopimelate epimerase* | | *Amino acid synthesis* |  |
|  | | *Arginosuccinate synthase* | | *Amino acid synthesis* |  |
| 1051 | | *Pantothenate Synthetase* | | *Cofactor Biosynthesis* |  |
|  | | *Folylpolyglutamate Synthase* | | *Cofactor Biosynthesis* |  |
|  | | *Diaminopimelate epimerase* | | *Amino acid synthesis* |  |
| 1052 | | *Acetyl-CoA acyltransferase anaerobic* | | *Fatty Acid Metabolism* |  |
|  | | *Diaminopimelate epimerase* | | *Amino acid synthesis* |  |
|  | | *Beta-ketoacyl-acyl-carrier-protein synthase III* | | *Fatty Acid Biosynthesis* |  |
| 1053 | | *N-acetylmuramoyl-L-alanine amidase* | | *Glycan Biosynthesis and Metabolism* |  |
|  | | *L-glutaminase* | | *Amino acid synthesis* |  |
|  | | *Pyruvate-Formate Lyase* | | *Respiration* |  |
| 1054 | | *Pyruvate-Formate Lyase* | | *Respiration* |  |
|  | | *Thymidine phosphorylase* | | *Pyrimidine metabolism* |  |
|  | | *Formyltetrahydrofolate synthetase* | | *Organic Acids* |  |
| 1055 | | *Beta-N-acetyl-D-hexosaminide N-acetylhexosaminohydrolase* | | *Glycosaminoglycan degradation* |  |
|  | | *Diaminopimelate epimerase* | | *Amino acid synthesis* |  |
|  | | *1-Phosphofructokinase* | | *Feeder Pathways to Glycolysis* |  |
| 1056 | | *Pyruvate-Formate Lyase* | | *Respiration* |  |
|  | | *Dihydrodipicolinate synthase* | | *Amino acid synthesis* |  |
|  | | *KDPG Aldolase* | | *Central Carbon Metabolism Pathways* |  |
| 1057 | | *N-acetylmuramoyl-L-alanine amidase* | | *Glycan Biosynthesis and Metabolism* |  |
|  | | *Beta-ketoacyl-acyl-carrier-protein synthase III* | | *Fatty Acid Biosynthesis* |  |
|  | | *Formyltetrahydrofolate synthetase* | | *Organic Acids* |  |
| 1058 | | *Diaminopimelate epimerase* | | *Amino acid synthesis* |  |
|  | | *Galactokinase* | | *Feeder Pathways to Glycolysis* |  |
|  | | *Si-Citrate Synthase* | | *Central Carbon Metabolism Pathways* |  |
| 1059 | | *Histidinol dehydrogenase* | | *Amino acid transport and metabolism* |  |
|  | | *N-acetylmuramoyl-L-alanine amidase* | | *Glycan Biosynthesis and Metabolism* |  |
|  | | *Histidinol dehydrogenase* | | *Amino acid transport and metabolism* |  |
| 1060 | | *Diaminopimelate epimerase* | | *Amino acid synthesis* |  |
|  | | *Glutamate synthase large and small subunit (NADPH)* | | *Amino acid synthesis* |  |
|  | | *Folylpolyglutamate Synthase* | | *Cofactor Biosynthesis* |  |
| 1061 | | *Alpha-mannosidase* | | *N-Glycan degradation* |  |
|  | | *Prephenate dehydrogenase* | | *Amino acid synthesis* |  |
|  | | *N-acetyl-D-glucosamine-6-sulfate 6-sulfohydrolase* | | *Glycosaminoglycan degradation* |  |
| 1062 | | *Dihydrodipicolinate synthase* | | *Amino acid synthesis* |  |
|  | | *Diaminopimelate epimerase* | | *Amino acid synthesis* |  |
|  | | *Acetyl-CoA acyltransferase anaerobic* | | *Fatty Acid Metabolism* |  |
| 1063 | | *Geranyltranstransferase* | | *Isoprenoid biosynthesis* |  |
|  | | *IMP dehydrogenase* | | *Purine metabolism* |  |
|  | | *Diaminopimelate epimerase* | | *Amino acid synthesis* |  |
| 1064 | | *N-acetylmuramoyl-L-alanine amidase* | | *Glycan Biosynthesis and Metabolism* |  |
|  | | *Quinolinate Synthase* | | *Cofactor Biosynthesis* |  |
|  | | *Selenocysteine synthase* | | *Amino acid synthesis;Amino acid synthesis* |  |
| 1065 | | *3-deoxy-7-phosphoheptulonate synthase* | | *Amino acid transport and metabolism* |  |
|  | | *Shikimate kinase I II* | | *Amino acid transport and metabolism* |  |
|  | | *Diaminopimelate epimerase* | | *Amino acid synthesis* |  |
| 1066 | | *Ribokinase* | | *Feeder Pathways to Glycolysis* |  |
|  | | *Cysteine synthase A* | | *Amino acid synthesis* |  |
|  | | *Diaminopimelate epimerase* | | *Amino acid synthesis* |  |
| 1067 | | *Prephenate dehydrogenase* | | *Amino acid synthesis* |  |
|  | | *ADP-ribose pyrophosphatase* | | *Purine metabolism* |  |
|  | | *Chorismate synthase* | | *Amino acid transport and metabolism* |  |
| 1068 | | *Cytosine deaminase* | | *Pyrimidine metabolism* |  |
|  | | *L-threonine aldolase* | | *Amino acid synthesis* |  |
|  | | *Pyruvate-Formate Lyase* | | *Respiration* |  |
| 1069 | | *N-acetylmuramoyl-L-alanine amidase* | | *Glycan Biosynthesis and Metabolism* |  |
|  | | *Anthranilate synthase* | | *Amino acid transport and metabolism* |  |
|  | | *Beta-D-galactosidase* | | *Glycan structures - degradation;Complex Carbohydrates* |  |
| 1070 | | *Cysteine synthase A* | | *Amino acid synthesis* |  |
|  | | *Prephenate dehydrogenase* | | *Amino acid synthesis* |  |
|  | | *Ribokinase* | | *Feeder Pathways to Glycolysis* |  |
| 1071 | | *Thioredoxin reductase FAD-NADP-binding* | | *Pyrimidine metabolism* |  |
|  | | *L-glutamine synthase* | | *Amino acid synthesis* |  |
|  | | *Prephenate dehydrogenase* | | *Amino acid synthesis* |  |
| 1072 | | *Glycerol Kinase* | | *Glycerolipid Metabolism* |  |
|  | | *Pyruvate-Formate Lyase* | | *Respiration* |  |
|  | | *Anthranilate synthase* | | *Amino acid transport and metabolism* |  |
| 1073 | | *Ornithine carbamoyltransferase 1* | | *Amino acid transport and metabolism* |  |
|  | | *Pyruvate-Formate Lyase* | | *Respiration* |  |
|  | | *Cystathionine gamma-synthase* | | *Amino acid synthesis* |  |
| 1074 | | *Thioredoxin reductase FAD-NADP-binding* | | *Pyrimidine metabolism* |  |
|  | | *Quinolinate Synthase* | | *Cofactor Biosynthesis* |  |
|  | | *Prephenate dehydrogenase* | | *Amino acid synthesis* |  |
| 1075 | | *UDP-N-acetylmuramateL-alanine ligase* | | *Glycan Biosynthesis and Metabolism* |  |
|  | | *Selenocysteine synthase* | | *Amino acid synthesis;Amino acid synthesis* |  |
|  | | *N-acetylmuramoyl-L-alanine amidase* | | *Glycan Biosynthesis and Metabolism* |  |
| 1076 | | *N-acetylmuramoyl-L-alanine amidase* | | *Glycan Biosynthesis and Metabolism* |  |
|  | | *sulfate adenylyltransferase subunit 2* | | *Purine metabolism* |  |
|  | | *Ribokinase* | | *Feeder Pathways to Glycolysis* |  |
| 1077 | | *Pyruvate-Formate Lyase* | | *Respiration* |  |
|  | | *N-acetylmuramoyl-L-alanine amidase* | | *Glycan Biosynthesis and Metabolism* |  |
|  | | *Thioredoxin reductase FAD-NADP-binding* | | *Pyrimidine metabolism* |  |
| 1078 | | *N-Acetylglucosamine-6-Phosphate Deacetylase* | | *Feeder Pathways to Glycolysis* |  |
|  | | *2-isopropylmalate synthase* | | *Amino acid synthesis* |  |
|  | | *N-acetylmuramoyl-L-alanine amidase* | | *Glycan Biosynthesis and Metabolism* |  |
| 1079 | | *Prephenate dehydrogenase* | | *Amino acid synthesis* |  |
|  | | *Beta-ketoacyl-acyl-carrier-protein synthase III* | | *Fatty Acid Biosynthesis* |  |
|  | | *N-Acetylglucosamine-6-Phosphate Deacetylase* | | *Feeder Pathways to Glycolysis* |  |
| 1080 | | *Beta-N-acetyl-D-hexosaminide N-acetylhexosaminohydrolase* | | *Glycosaminoglycan degradation* |  |
|  | | *Prephenate dehydrogenase* | | *Amino acid synthesis* |  |
|  | | *N-acetyl-D-galactosamine-4-sulfate 4-sulfohydrolase* | | *Glycosaminoglycan degradation* |  |
| 1081 | | *Gamma-glutamyl kinase* | | *Amino acid transport and metabolism* |  |
|  | | *Glutamate synthase large and small subunit (NADPH)* | | *Amino acid synthesis* |  |
|  | | *Prephenate dehydrogenase* | | *Amino acid synthesis* |  |
| 1082 | | *L-alanine dehydrogenase* | | *Amino acid synthesis* |  |
|  | | *2-isopropylmalate synthase* | | *Amino acid synthesis* |  |
|  | | *Diaminopimelate epimerase* | | *Amino acid synthesis* |  |
| 1083 | | *D-alanineD-alanine ligase* | | *Glycan Biosynthesis and Metabolism* |  |
|  | | *Alpha-Glucosidase* | | *Complex Carbohydrates* |  |
|  | | *Prephenate dehydrogenase* | | *Amino acid synthesis* |  |
| 1084 | | *Diaminopimelate epimerase* | | *Amino acid synthesis* |  |
|  | | *Alpha-Glucosidase* | | *Complex Carbohydrates* |  |
|  | | *N-Acetylglucosamine-6-Phosphate Deacetylase* | | *Feeder Pathways to Glycolysis* |  |
| 1085 | | *Phosphoribosylglycinamide synthetase phosphoribosylamine-glycine ligase* | | *Purine metabolism* |  |
|  | | *Pyruvate-Formate Lyase* | | *Respiration* |  |
|  | | *Pyridoxal Kinase* | | *Cofactor Biosynthesis* |  |
| 1086 | | *Pyruvate-Formate Lyase* | | *Respiration* |  |
|  | | *Phosphoribosylglycinamide synthetase phosphoribosylamine-glycine ligase* | | *Purine metabolism* |  |
|  | | *Glutamate synthase large and small subunit (NADPH)* | | *Amino acid synthesis* |  |
| 1087 | | *Gamma-glutamyl kinase* | | *Amino acid transport and metabolism* |  |
|  | | *L-alanine dehydrogenase* | | *Amino acid synthesis* |  |
|  | | *Diaminopimelate epimerase* | | *Amino acid synthesis* |  |
| 1088 | | *Glutamate racemase* | | *Amino acid synthesis* |  |
|  | | *Prephenate dehydrogenase* | | *Amino acid synthesis* |  |
|  | | *D-alanineD-alanine ligase* | | *Glycan Biosynthesis and Metabolism* |  |
| 1089 | | *Diaminopimelate epimerase* | | *Amino acid synthesis* |  |
|  | | *Carbamoyl phosphate synthetase small subunit glutamine amidotransferase* | | *Pyrimidine metabolism* |  |
|  | | *Pantothenate Synthetase* | | *Cofactor Biosynthesis* |  |
| 1090 | | *N-Acetylglucosamine-6-Phosphate Deacetylase* | | *Feeder Pathways to Glycolysis* |  |
|  | | *Anaerobic ribonucleoside-triphosphate reductase* | | *Pyrimidine metabolism* |  |
|  | | *N-acetylmuramoyl-L-alanine amidase* | | *Glycan Biosynthesis and Metabolism* |  |
| 1091 | | *Cytidylate kinase* | | *Pyrimidine metabolism* |  |
|  | | *Prephenate dehydrogenase* | | *Amino acid synthesis* |  |
|  | | *Ribokinase* | | *Feeder Pathways to Glycolysis* |  |
| 1092 | | *Riboflavin Synthase α Subunit* | | *Cofactor Biosynthesis* |  |
|  | | *N-acetylmuramoyl-L-alanine amidase* | | *Glycan Biosynthesis and Metabolism* |  |
|  | | *4-diphosphocytidyl-2C-methyl-D-erythritol synthase* | | *Isoprenoid biosynthesis* |  |
| 1093 | | *Cysteine synthase A* | | *Amino acid synthesis* |  |
|  | | *Acetyl-CoA acyltransferase anaerobic* | | *Fatty Acid Metabolism* |  |
|  | | *Prephenate dehydrogenase* | | *Amino acid synthesis* |  |
| 1094 | | *Pantothenate Synthetase* | | *Cofactor Biosynthesis* |  |
|  | | *Pyruvate-Formate Lyase* | | *Respiration* |  |
|  | | *L-alanine dehydrogenase* | | *Amino acid synthesis* |  |
| 1095 | | *ADP-ribose pyrophosphatase* | | *Purine metabolism* |  |
|  | | *Prephenate dehydrogenase* | | *Amino acid synthesis* |  |
|  | | *Cysteine synthase A* | | *Amino acid synthesis* |  |
| 1096 | | *Glutamate racemase* | | *Amino acid synthesis* |  |
|  | | *Alpha-Glucosidase* | | *Complex Carbohydrates* |  |
|  | | *Pyruvate-Formate Lyase* | | *Respiration* |  |
| 1097 | | *N-acetylmuramoyl-L-alanine amidase* | | *Glycan Biosynthesis and Metabolism* |  |
|  | | *Formyltetrahydrofolate synthetase* | | *Organic Acids* |  |
|  | | *L-alanine dehydrogenase* | | *Amino acid synthesis* |  |
| 1098 | | *Prephenate dehydrogenase* | | *Amino acid synthesis* |  |
|  | | *Cytidylate kinase* | | *Pyrimidine metabolism* |  |
|  | | *Phosphoribosylglycinamide synthetase phosphoribosylamine-glycine ligase* | | *Purine metabolism* |  |
| 1099 | | *Pyruvate-Formate Lyase* | | *Respiration* |  |
|  | | *Beta-ketoacyl-acyl-carrier-protein synthase III* | | *Fatty Acid Biosynthesis* |  |
|  | | *N-acetyl-D-galactosamine-4-sulfate 4-sulfohydrolase* | | *Glycosaminoglycan degradation* |  |
| 1100 | | *Pyruvate-Formate Lyase* | | *Respiration* |  |
|  | | *Alpha-mannosidase* | | *N-Glycan degradation* |  |
|  | | *Purine-nucleoside phosphorylase* | | *Purine metabolism* |  |
| 1101 | | *UDP-N-acetylmuramateL-alanine ligase* | | *Glycan Biosynthesis and Metabolism* |  |
|  | | *Acetyl-CoA acyltransferase anaerobic* | | *Fatty Acid Metabolism* |  |
|  | | *Pyruvate-Formate Lyase* | | *Respiration* |  |
| 1102 | | *Threonine ammonia-lyase* | | *Amino acid synthesis* |  |
|  | | *N-acetylmuramoyl-L-alanine amidase* | | *Glycan Biosynthesis and Metabolism* |  |
|  | | *Thioredoxin reductase FAD-NADP-binding* | | *Pyrimidine metabolism* |  |
| 1103 | | *Pyruvate-Formate Lyase* | | *Respiration* |  |
|  | | *Alpha-Glucosidase* | | *Complex Carbohydrates* |  |
|  | | *Cytosine deaminase* | | *Pyrimidine metabolism* |  |
| 1104 | | *3-demethylubiquinone-9 3-methyltransferase* | | *Cofactor Biosynthesis* |  |
|  | | *Diaminopimelate epimerase* | | *Amino acid synthesis* |  |
|  | | *3-deoxy-7-phosphoheptulonate synthase* | | *Amino acid transport and metabolism* |  |
| 1105 | | *Diaminopimelate epimerase* | | *Amino acid synthesis* |  |
|  | | *N-acetylglutamate synthase* | | *Amino acid transport and metabolism* |  |
|  | | *N-Acetylglucosamine-6-Phosphate Deacetylase* | | *Feeder Pathways to Glycolysis* |  |
| 1106 | | *2-isopropylmalate synthase* | | *Amino acid synthesis* |  |
|  | | *Glycogen Synthase* | | *Complex Carbohydrates* |  |
|  | | *N-acetylmuramoyl-L-alanine amidase* | | *Glycan Biosynthesis and Metabolism* |  |
| 1107 | | *N-acetylmuramoyl-L-alanine amidase* | | *Glycan Biosynthesis and Metabolism* |  |
|  | | *Diaminopimelate epimerase* | | *Amino acid synthesis* |  |
|  | | *Glucuronate Isomerase* | | *Feeder Pathways to Glycolysis* |  |
| 1108 | | *Ribokinase* | | *Feeder Pathways to Glycolysis* |  |
|  | | *Prephenate dehydrogenase* | | *Amino acid synthesis* |  |
|  | | *Glutamate racemase* | | *Amino acid synthesis* |  |
| 1109 | | *Pyruvate-Formate Lyase* | | *Respiration* |  |
|  | | *Beta-ketoacyl-acyl-carrier-protein synthase III* | | *Fatty Acid Biosynthesis* |  |
|  | | *Glucuronate Isomerase* | | *Feeder Pathways to Glycolysis* |  |
| 1110 | | *L-threonine synthase* | | *Amino acid synthesis* |  |
|  | | *Glycerol Kinase* | | *Glycerolipid Metabolism* |  |
|  | | *Prephenate dehydrogenase* | | *Amino acid synthesis* |  |
| 1111 | | *Ribokinase* | | *Feeder Pathways to Glycolysis* |  |
|  | | *N-acetyl-D-glucosamine-6-sulfate 6-sulfohydrolase* | | *Glycosaminoglycan degradation* |  |
|  | | *Pyruvate-Formate Lyase* | | *Respiration* |  |
| 1112 | | *UDP-N-acetylmuramateL-alanine ligase* | | *Glycan Biosynthesis and Metabolism* |  |
|  | | *Adenylosuccinate synthetase* | | *Purine metabolism* |  |
|  | | *N-acetylmuramoyl-L-alanine amidase* | | *Glycan Biosynthesis and Metabolism* |  |
| 1113 | | *N-acetylmuramoyl-L-alanine amidase* | | *Glycan Biosynthesis and Metabolism* |  |
|  | | *Asparaginase* | | *Amino acid synthesis* |  |
|  | | *Anthranilate synthase* | | *Amino acid transport and metabolism* |  |
| 1114 | | *Acetyl-CoA acyltransferase anaerobic* | | *Fatty Acid Metabolism* |  |
|  | | *Purine-nucleoside phosphorylase* | | *Purine metabolism* |  |
|  | | *Diaminopimelate epimerase* | | *Amino acid synthesis* |  |
| 1115 | | *UDP-N-acetylglucosamine acyltransferase* | | *Glycan Biosynthesis and Metabolism* |  |
|  | | *Prephenate dehydrogenase* | | *Amino acid synthesis* |  |
|  | | *Aspartate-ammonia ligase* | | *Amino acid synthesis* |  |
| 1116 | | *Folylpolyglutamate Synthase* | | *Cofactor Biosynthesis* |  |
|  | | *Pyruvate-Formate Lyase* | | *Respiration* |  |
|  | | *UDP-N-acetylmuramateL-alanine ligase* | | *Glycan Biosynthesis and Metabolism* |  |
| 1117 | | *Beta-D-galactosidase* | | *Glycan structures - degradation;Complex Carbohydrates* |  |
|  | | *Aspartate-ammonia ligase* | | *Amino acid synthesis* |  |
|  | | *Pyruvate-Formate Lyase* | | *Respiration* |  |
| 1118 | | *N-acetylmuramoyl-L-alanine amidase* | | *Glycan Biosynthesis and Metabolism* |  |
|  | | *Ribokinase* | | *Feeder Pathways to Glycolysis* |  |
|  | | *Folylpolyglutamate Synthase* | | *Cofactor Biosynthesis* |  |
| 1119 | | *Prephenate dehydrogenase* | | *Amino acid synthesis* |  |
|  | | *Anthranilate synthase* | | *Amino acid transport and metabolism* |  |
|  | | *3-dehydroquinate dehydratase* | | *Amino acid transport and metabolism* |  |
| 1120 | | *Beta-D-galactosidase* | | *Glycan structures - degradation;Complex Carbohydrates* |  |
|  | | *N-acetyl-D-glucosamine-6-sulfate 6-sulfohydrolase* | | *Glycosaminoglycan degradation* |  |
|  | | *Pyruvate-Formate Lyase* | | *Respiration* |  |
| 1121 | | *Methylmalonyl-CaA decarboxylase* | | *Organic Acids* |  |
|  | | *Beta-N-acetyl-D-hexosaminide N-acetylhexosaminohydrolase* | | *Glycosaminoglycan degradation* |  |
|  | | *Prephenate dehydrogenase* | | *Amino acid synthesis* |  |
| 1122 | | *Carbon Monoxide Dehydrogenase* | | *Central Carbon Metabolism Pathways* |  |
|  | | *N-acetylmuramoyl-L-alanine amidase* | | *Glycan Biosynthesis and Metabolism* |  |
|  | | *Pantothenate Synthetase* | | *Cofactor Biosynthesis* |  |
| 1123 | | *UDP-N-acetylmuramateL-alanine ligase* | | *Glycan Biosynthesis and Metabolism* |  |
|  | | *Ribokinase* | | *Feeder Pathways to Glycolysis* |  |
|  | | *N-acetylmuramoyl-L-alanine amidase* | | *Glycan Biosynthesis and Metabolism* |  |
| 1124 | | *Cystathionine gamma-synthase* | | *Amino acid synthesis* |  |
|  | | *Biotin Synthase* | | *Cofactor Biosynthesis* |  |
|  | | *Diaminopimelate epimerase* | | *Amino acid synthesis* |  |
| 1125 | | *Beta-D-galactosidase* | | *Glycan structures - degradation;Complex Carbohydrates* |  |
|  | | *Pyruvate-Formate Lyase* | | *Respiration* |  |
|  | | *Butyrate Kinase* | | *Organic Acids* |  |
| 1126 | | *Beta-N-acetyl-D-hexosaminide N-acetylhexosaminohydrolase* | | *Glycosaminoglycan degradation* |  |
|  | | *Beta-D-galactosidase* | | *Glycan structures - degradation;Complex Carbohydrates* |  |
|  | | *Prephenate dehydrogenase* | | *Amino acid synthesis* |  |
| 1127 | | *Pyruvate-Formate Lyase* | | *Respiration* |  |
|  | | *ketol-acid reductoisomerase* | | *Amino acid synthesis* |  |
|  | | *Asparaginase* | | *Amino acid synthesis* |  |
| 1128 | | *N-acetylmuramoyl-L-alanine amidase* | | *Glycan Biosynthesis and Metabolism* |  |
|  | | *ketol-acid reductoisomerase* | | *Amino acid synthesis* |  |
|  | | *Cytidylate kinase* | | *Pyrimidine metabolism* |  |
| 1129 | | *Diaminopimelate epimerase* | | *Amino acid synthesis* |  |
|  | | *Cystathionine gamma-synthase* | | *Amino acid synthesis* |  |
|  | | *L-alanine dehydrogenase* | | *Amino acid synthesis* |  |
| 1130 | | *L-glutaminase* | | *Amino acid synthesis* |  |
|  | | *Dihydrodipicolinate synthase* | | *Amino acid synthesis* |  |
|  | | *N-acetylmuramoyl-L-alanine amidase* | | *Glycan Biosynthesis and Metabolism* |  |
| 1131 | | *Diaminopimelate epimerase* | | *Amino acid synthesis* |  |
|  | | *Beta-N-acetyl-D-hexosaminide N-acetylhexosaminohydrolase* | | *Glycosaminoglycan degradation* |  |
|  | | *Fucose Isomerase* | | *Feeder Pathways to Glycolysis* |  |
| 1132 | | *Diaminopimelate epimerase* | | *Amino acid synthesis* |  |
|  | | *ketol-acid reductoisomerase* | | *Amino acid synthesis* |  |
|  | | *Glycerol Kinase* | | *Glycerolipid Metabolism* |  |
| 1133 | | *Dihydroxy-acid dehydratase* | | *Amino acid transport and metabolism* |  |
|  | | *N-acetylmuramoyl-L-alanine amidase* | | *Glycan Biosynthesis and Metabolism* |  |
|  | | *Conjugated Bile Salt Hydrolase* | | *Exotic Metabolisms* |  |
| 1134 | | *Thioredoxin reductase FAD-NADP-binding* | | *Pyrimidine metabolism* |  |
|  | | *Pyruvate-Formate Lyase* | | *Respiration* |  |
|  | | *4-diphosphocytidyl-2C-methyl-D-erythritol synthase* | | *Isoprenoid biosynthesis* |  |
| 1135 | | *Shikimate kinase I II* | | *Amino acid transport and metabolism* |  |
|  | | *Valine-pyruvate aminotransferase* | | *Amino acid synthesis* |  |
|  | | *Prephenate dehydrogenase* | | *Amino acid synthesis* |  |
| 1136 | | *L-glutamine synthase* | | *Amino acid synthesis* |  |
|  | | *Alanine racemase* | | *Amino acid synthesis* |  |
|  | | *Diaminopimelate epimerase* | | *Amino acid synthesis* |  |
| 1137 | | *Beta-ketoacyl-acyl-carrier-protein synthase III* | | *Fatty Acid Biosynthesis* |  |
|  | | *Pyruvate-Formate Lyase* | | *Respiration* |  |
|  | | *4-diphosphocytidyl-2C-methyl-D-erythritol synthase* | | *Isoprenoid biosynthesis* |  |
| 1138 | | *Prephenate dehydrogenase* | | *Amino acid synthesis* |  |
|  | | *Ornithine carbamoyltransferase 1* | | *Amino acid transport and metabolism* |  |
|  | | *Spermidine Synthase* | | *Nitrogen Metabolism;Amino acid transport and metabolism* |  |
| 1139 | | *Diaminopimelate epimerase* | | *Amino acid synthesis* |  |
|  | | *Mannose-6-Phosphate Isomerase* | | *Feeder Pathways to Glycolysis* |  |
|  | | *Transketolase* | | *Central Carbon Metabolism Pathways* |  |
| 1140 | | *N-acetylmuramoyl-L-alanine amidase* | | *Glycan Biosynthesis and Metabolism* |  |
|  | | *Pyridoxal Kinase* | | *Cofactor Biosynthesis* |  |
|  | | *Uridine phosphorylase* | | *Pyrimidine metabolism* |  |
| 1141 | | *Prephenate dehydrogenase* | | *Amino acid synthesis* |  |
|  | | *UDP-N-acetylmuramateL-alanine ligase* | | *Glycan Biosynthesis and Metabolism* |  |
|  | | *Cytidylate kinase* | | *Pyrimidine metabolism* |  |
| 1142 | | *Phosphoribosylglycinamide synthetase phosphoribosylamine-glycine ligase* | | *Purine metabolism* |  |
|  | | *Acetyl-CoA acyltransferase anaerobic* | | *Fatty Acid Metabolism* |  |
|  | | *Diaminopimelate epimerase* | | *Amino acid synthesis* |  |
| 1143 | | *Pyruvate-Formate Lyase* | | *Respiration* |  |
|  | | *Geranyltranstransferase* | | *Isoprenoid biosynthesis* |  |
|  | | *Cytosine deaminase* | | *Pyrimidine metabolism* |  |
| 1144 | | *3-dehydroquinate dehydratase* | | *Amino acid transport and metabolism* |  |
|  | | *D-alanineD-alanine ligase* | | *Glycan Biosynthesis and Metabolism* |  |
|  | | *Diaminopimelate epimerase* | | *Amino acid synthesis* |  |
| 1145 | | *Diaminopimelate epimerase* | | *Amino acid synthesis* |  |
|  | | *Alpha-Glucosidase* | | *Complex Carbohydrates* |  |
|  | | *Aspartate-ammonia ligase* | | *Amino acid synthesis* |  |
| 1146 | | *Pyruvate-Formate Lyase* | | *Respiration* |  |
|  | | *3-deoxy-7-phosphoheptulonate synthase* | | *Amino acid transport and metabolism* |  |
|  | | *Aspartate kinase* | | *Amino acid synthesis* |  |
| 1147 | | *Diaminopimelate epimerase* | | *Amino acid synthesis* |  |
|  | | *Phosphoribosylglycinamide synthetase phosphoribosylamine-glycine ligase* | | *Purine metabolism* |  |
|  | | *Formyltetrahydrofolate synthetase* | | *Organic Acids* |  |
| 1148 | | *Dihydroxy-acid dehydratase* | | *Amino acid transport and metabolism* |  |
|  | | *Diaminopimelate epimerase* | | *Amino acid synthesis* |  |
|  | | *Cysteine synthase A* | | *Amino acid synthesis* |  |
| 1149 | | *Riboflavin Synthase α Subunit* | | *Cofactor Biosynthesis* |  |
|  | | *Diaminopimelate epimerase* | | *Amino acid synthesis* |  |
|  | | *Thioredoxin reductase FAD-NADP-binding* | | *Pyrimidine metabolism* |  |
| 1150 | | *4-diphosphocytidyl-2C-methyl-D-erythritol synthase* | | *Isoprenoid biosynthesis* |  |
|  | | *Pyruvate-Formate Lyase* | | *Respiration* |  |
|  | | *Thioredoxin reductase FAD-NADP-binding* | | *Pyrimidine metabolism* |  |
| 1151 | | *Butyrate Kinase* | | *Organic Acids* |  |
|  | | *Prephenate dehydrogenase* | | *Amino acid synthesis* |  |
|  | | *N-acetylmuramoyl-L-alanine amidase* | | *Glycan Biosynthesis and Metabolism* |  |
| 1152 | | *Thioredoxin reductase FAD-NADP-binding* | | *Pyrimidine metabolism* |  |
|  | | *N-acetyl-D-galactosamine-4-sulfate 4-sulfohydrolase* | | *Glycosaminoglycan degradation* |  |
|  | | *Prephenate dehydrogenase* | | *Amino acid synthesis* |  |
| 1153 | | *Pyruvate-Formate Lyase* | | *Respiration* |  |
|  | | *Mannose-6-Phosphate Isomerase* | | *Feeder Pathways to Glycolysis* |  |
|  | | *Valine-pyruvate aminotransferase* | | *Amino acid synthesis* |  |
| 1154 | | *Transketolase* | | *Central Carbon Metabolism Pathways* |  |
|  | | *Prephenate dehydrogenase* | | *Amino acid synthesis* |  |
|  | | *Alpha-mannosidase* | | *N-Glycan degradation* |  |
| 1155 | | *Ribokinase* | | *Feeder Pathways to Glycolysis* |  |
|  | | *Folylpolyglutamate Synthase* | | *Cofactor Biosynthesis* |  |
|  | | *N-acetylmuramoyl-L-alanine amidase* | | *Glycan Biosynthesis and Metabolism* |  |
| 1156 | | *Anthranilate synthase* | | *Amino acid transport and metabolism* |  |
|  | | *Carbon Monoxide Dehydrogenase* | | *Central Carbon Metabolism Pathways* |  |
|  | | *Pyruvate-Formate Lyase* | | *Respiration* |  |
| 1157 | | *Formyltetrahydrofolate synthetase* | | *Organic Acids* |  |
|  | | *Prephenate dehydrogenase* | | *Amino acid synthesis* |  |
|  | | *UDP-N-acetylmuramoyl-L-alanineD-glutamate ligase* | | *Glycan Biosynthesis and Metabolism* |  |
| 1158 | | *Conjugated Bile Salt Hydrolase* | | *Exotic Metabolisms* |  |
|  | | *Pyruvate-Formate Lyase* | | *Respiration* |  |
|  | | *UDP-N-acetylmuramateL-alanine ligase* | | *Glycan Biosynthesis and Metabolism* |  |
| 1159 | | *Beta-D-glucuronidase* | | *Glycan structures - degradation;Exotic Metabolisms* |  |
|  | | *Pyruvate-Formate Lyase* | | *Respiration* |  |
|  | | *Phosphoribosylglycinamide synthetase phosphoribosylamine-glycine ligase* | | *Purine metabolism* |  |
| 1160 | | *Spermidine Synthase* | | *Nitrogen Metabolism;Amino acid transport and metabolism* |  |
|  | | *Carbon Monoxide Dehydrogenase* | | *Central Carbon Metabolism Pathways* |  |
|  | | *Pyruvate-Formate Lyase* | | *Respiration* |  |
| 1161 | | *Prephenate dehydrogenase* | | *Amino acid synthesis* |  |
|  | | *Prephenate dehydrogenase* | | *Amino acid synthesis* |  |
|  | | *Cobalamin Synthase* | | *Cofactor Biosynthesis* |  |
| 1162 | | *Anthranilate synthase* | | *Amino acid transport and metabolism* |  |
|  | | *Acetyl-CoA acyltransferase anaerobic* | | *Fatty Acid Metabolism* |  |
|  | | *Diaminopimelate epimerase* | | *Amino acid synthesis* |  |
| 1163 | | *Beta-ketoacyl-acyl-carrier-protein synthase III* | | *Fatty Acid Biosynthesis* |  |
|  | | *3-dehydroquinate dehydratase* | | *Amino acid transport and metabolism* |  |
|  | | *Prephenate dehydrogenase* | | *Amino acid synthesis* |  |
| 1164 | | *Prephenate dehydrogenase* | | *Amino acid synthesis* |  |
|  | | *Carbamoyl phosphate synthetase small subunit glutamine amidotransferase* | | *Pyrimidine metabolism* |  |
|  | | *Thioredoxin reductase FAD-NADP-binding* | | *Pyrimidine metabolism* |  |
| 1165 | | *Diaminopimelate epimerase* | | *Amino acid synthesis* |  |
|  | | *Chorismate synthase* | | *Amino acid transport and metabolism* |  |
|  | | *N-Acetylglucosamine-6-Phosphate Deacetylase* | | *Feeder Pathways to Glycolysis* |  |
| 1166 | | *Diaminopimelate epimerase* | | *Amino acid synthesis* |  |
|  | | *Cytosine deaminase* | | *Pyrimidine metabolism* |  |
|  | | *Pyruvate-Formate Lyase* | | *Respiration* |  |
| 1167 | | *Diaminopimelate epimerase* | | *Amino acid synthesis* |  |
|  | | *3-dehydroquinate dehydratase* | | *Amino acid transport and metabolism* |  |
|  | | *Dihydrodipicolinate synthase* | | *Amino acid synthesis* |  |
| 1168 | | *Cysteine synthase A* | | *Amino acid synthesis* |  |
|  | | *Glutamate racemase* | | *Amino acid synthesis* |  |
|  | | *Prephenate dehydrogenase* | | *Amino acid synthesis* |  |
| 1169 | | *N-acetylmuramoyl-L-alanine amidase* | | *Glycan Biosynthesis and Metabolism* |  |
|  | | *Acetyl-CoA acyltransferase anaerobic* | | *Fatty Acid Metabolism* |  |
|  | | *Transketolase* | | *Central Carbon Metabolism Pathways* |  |
| 1170 | | *1-Phosphofructokinase* | | *Feeder Pathways to Glycolysis* |  |
|  | | *KDPG Aldolase* | | *Central Carbon Metabolism Pathways* |  |
|  | | *Diaminopimelate epimerase* | | *Amino acid synthesis* |  |
| 1171 | | *Cobalamin Synthase* | | *Cofactor Biosynthesis* |  |
|  | | *Diaminopimelate epimerase* | | *Amino acid synthesis* |  |
|  | | *Alanine racemase* | | *Amino acid synthesis* |  |
| 1172 | | *N-acetylmuramoyl-L-alanine amidase* | | *Glycan Biosynthesis and Metabolism* |  |
|  | | *2-isopropylmalate synthase* | | *Amino acid synthesis* |  |
|  | | *Carbamoyl phosphate synthetase small subunit glutamine amidotransferase* | | *Pyrimidine metabolism* |  |
| 1173 | | *Pyruvate-Formate Lyase* | | *Respiration* |  |
|  | | *Glucuronate Isomerase* | | *Feeder Pathways to Glycolysis* |  |
|  | | *3-deoxy-7-phosphoheptulonate synthase* | | *Amino acid transport and metabolism* |  |
| 1174 | | *UDP-N-acetylmuramateL-alanine ligase* | | *Glycan Biosynthesis and Metabolism* |  |
|  | | *Beta-N-acetyl-D-hexosaminide N-acetylhexosaminohydrolase* | | *Glycosaminoglycan degradation* |  |
|  | | *Diaminopimelate epimerase* | | *Amino acid synthesis* |  |
| 1175 | | *Anthranilate synthase* | | *Amino acid transport and metabolism* |  |
|  | | *Carbon Monoxide Dehydrogenase* | | *Central Carbon Metabolism Pathways* |  |
|  | | *Pyruvate-Formate Lyase* | | *Respiration* |  |
| 1176 | | *Pyrroline-5-carboxylate reductase* | | *Amino acid transport and metabolism* |  |
|  | | *Beta-N-acetyl-D-hexosaminide N-acetylhexosaminohydrolase* | | *Glycosaminoglycan degradation* |  |
|  | | *Diaminopimelate epimerase* | | *Amino acid synthesis* |  |
| 1177 | | *Alpha-Glucosidase* | | *Complex Carbohydrates* |  |
|  | | *UDP-N-acetylglucosamine acyltransferase* | | *Glycan Biosynthesis and Metabolism* |  |
|  | | *Pyruvate-Formate Lyase* | | *Respiration* |  |
| 1178 | | *Serine-tRNA ligase* | | *Amino acid synthesis* |  |
|  | | *Cysteine synthase A* | | *Amino acid synthesis* |  |
|  | | *Prephenate dehydrogenase* | | *Amino acid synthesis* |  |
| 1179 | | *Pyruvate-Formate Lyase* | | *Respiration* |  |
|  | | *Ornithine carbamoyltransferase 1* | | *Amino acid transport and metabolism* |  |
|  | | *Cytidylate kinase* | | *Pyrimidine metabolism* |  |
| 1180 | | *Pyruvate-Formate Lyase* | | *Respiration* |  |
|  | | *Transketolase* | | *Central Carbon Metabolism Pathways* |  |
|  | | *Fucose Isomerase* | | *Feeder Pathways to Glycolysis* |  |
| 1181 | | *2-isopropylmalate synthase* | | *Amino acid synthesis* |  |
|  | | *Diaminopimelate epimerase* | | *Amino acid synthesis* |  |
|  | | *Lysine decarboxylase 1* | | *Amino acid transport and metabolism* |  |
| 1182 | | *Ribokinase* | | *Feeder Pathways to Glycolysis* |  |
|  | | *Prephenate dehydrogenase* | | *Amino acid synthesis* |  |
|  | | *Beta-D-glucuronidase* | | *Glycan structures - degradation;Exotic Metabolisms* |  |
| 1183 | | *Prephenate dehydrogenase* | | *Amino acid synthesis* |  |
|  | | *Anaerobic ribonucleoside-triphosphate reductase* | | *Pyrimidine metabolism* |  |
|  | | *3-dehydroquinate dehydratase* | | *Amino acid transport and metabolism* |  |
| 1184 | | *Anthranilate synthase* | | *Amino acid transport and metabolism* |  |
|  | | *N-acetylmuramoyl-L-alanine amidase* | | *Glycan Biosynthesis and Metabolism* |  |
|  | | *Cysteine synthase A* | | *Amino acid synthesis* |  |
| 1185 | | *Diaminopimelate epimerase* | | *Amino acid synthesis* |  |
|  | | *Glucuronate Isomerase* | | *Feeder Pathways to Glycolysis* |  |
|  | | *Diaminopimelate epimerase* | | *Amino acid synthesis* |  |
| 1186 | | *Histidinol dehydrogenase* | | *Amino acid transport and metabolism* |  |
|  | | *Serine-tRNA ligase* | | *Amino acid synthesis* |  |
|  | | *Prephenate dehydrogenase* | | *Amino acid synthesis* |  |
| 1187 | | *3-deoxy-7-phosphoheptulonate synthase* | | *Amino acid transport and metabolism* |  |
|  | | *Cystathionine gamma-synthase* | | *Amino acid synthesis* |  |
|  | | *Pyruvate-Formate Lyase* | | *Respiration* |  |
| 1188 | | *Cystathionine gamma-synthase* | | *Amino acid synthesis* |  |
|  | | *N-acetyl-D-glucosamine-6-sulfate 6-sulfohydrolase* | | *Glycosaminoglycan degradation* |  |
|  | | *Pyruvate-Formate Lyase* | | *Respiration* |  |
| 1189 | | *Pyridoxal Kinase* | | *Cofactor Biosynthesis* |  |
|  | | *Cytosine deaminase* | | *Pyrimidine metabolism* |  |
|  | | *Pyruvate-Formate Lyase* | | *Respiration* |  |
| 1190 | | *Aspartate-ammonia ligase* | | *Amino acid synthesis* |  |
|  | | *Diaminopimelate epimerase* | | *Amino acid synthesis* |  |
|  | | *Pyruvate-Formate Lyase* | | *Respiration* |  |
| 1191 | | *Alanine racemase* | | *Amino acid synthesis* |  |
|  | | *Prephenate dehydrogenase* | | *Amino acid synthesis* |  |
|  | | *Pyridoxal Kinase* | | *Cofactor Biosynthesis* |  |
| 1192 | | *Glucuronate Isomerase* | | *Feeder Pathways to Glycolysis* |  |
|  | | *Thymidine phosphorylase* | | *Pyrimidine metabolism* |  |
|  | | *Pyruvate-Formate Lyase* | | *Respiration* |  |
| 1193 | | *UDP-N-acetylglucosamine acyltransferase* | | *Glycan Biosynthesis and Metabolism* |  |
|  | | *Pyruvate-Formate Lyase* | | *Respiration* |  |
|  | | *Mannose-6-Phosphate Isomerase* | | *Feeder Pathways to Glycolysis* |  |
| 1194 | | *Pyruvate-Formate Lyase* | | *Respiration* |  |
|  | | *Anthranilate synthase* | | *Amino acid transport and metabolism* |  |
|  | | *N-acetylmuramoyl-L-alanine amidase* | | *Glycan Biosynthesis and Metabolism* |  |
| 1195 | | *Acetyl-CoA acyltransferase anaerobic* | | *Fatty Acid Metabolism* |  |
|  | | *Pyruvate-Formate Lyase* | | *Respiration* |  |
|  | | *N-acetyl-D-galactosamine-4-sulfate 4-sulfohydrolase* | | *Glycosaminoglycan degradation* |  |
| 1196 | | *L-glutamine synthase* | | *Amino acid synthesis* |  |
|  | | *Diaminopimelate epimerase* | | *Amino acid synthesis* |  |
|  | | *Folylpolyglutamate Synthase* | | *Cofactor Biosynthesis* |  |
| 1197 | | *Pyruvate-Formate Lyase* | | *Respiration* |  |
|  | | *N-acetyl-D-glucosamine-6-sulfate 6-sulfohydrolase* | | *Glycosaminoglycan degradation* |  |
|  | | *Acetyl-CoA acyltransferase anaerobic* | | *Fatty Acid Metabolism* |  |
| 1198 | | *Cysteine synthase A* | | *Amino acid synthesis* |  |
|  | | *Prephenate dehydrogenase* | | *Amino acid synthesis* |  |
|  | | *Formyltetrahydrofolate synthetase* | | *Organic Acids* |  |
| 1199 | | *Ornithine carbamoyltransferase 1* | | *Amino acid transport and metabolism* |  |
|  | | *Prephenate dehydrogenase* | | *Amino acid synthesis* |  |
|  | | *Adenylosuccinate synthetase* | | *Purine metabolism* |  |
| 1200 | | *Diaminopimelate epimerase* | | *Amino acid synthesis* |  |
|  | | *Ornithine carbamoyltransferase 1* | | *Amino acid transport and metabolism* |  |
|  | | *Dihydrodipicolinate synthase* | | *Amino acid synthesis* |  |
| 1201 | | *Ornithine carbamoyltransferase 1* | | *Amino acid transport and metabolism* |  |
|  | | *Diaminopimelate epimerase* | | *Amino acid synthesis* |  |
|  | | *IMP dehydrogenase* | | *Purine metabolism* |  |
| 1202 | | *UDP-N-acetylmuramoyl-L-alanineD-glutamate ligase* | | *Glycan Biosynthesis and Metabolism* |  |
|  | | *Glutamate racemase* | | *Amino acid synthesis* |  |
|  | | *Prephenate dehydrogenase* | | *Amino acid synthesis* |  |
| 1203 | | *N-acetylmuramoyl-L-alanine amidase* | | *Glycan Biosynthesis and Metabolism* |  |
|  | | *Carbon Monoxide Dehydrogenase* | | *Central Carbon Metabolism Pathways* |  |
|  | | *Aspartate kinase* | | *Amino acid synthesis* |  |
| 1204 | | *Diaminopimelate epimerase* | | *Amino acid synthesis* |  |
|  | | *Beta-D-galactosidase* | | *Glycan structures - degradation;Complex Carbohydrates* |  |
|  | | *Carbamoyl phosphate synthetase small subunit glutamine amidotransferase* | | *Pyrimidine metabolism* |  |
| 1205 | | *Thioredoxin reductase FAD-NADP-binding* | | *Pyrimidine metabolism* |  |
|  | | *Pyruvate-Formate Lyase* | | *Respiration* |  |
|  | | *Glucuronate Isomerase* | | *Feeder Pathways to Glycolysis* |  |
| 1206 | | *D-alanineD-alanine ligase* | | *Glycan Biosynthesis and Metabolism* |  |
|  | | *Pyruvate-Formate Lyase* | | *Respiration* |  |
|  | | *Thymidine phosphorylase* | | *Pyrimidine metabolism* |  |
| 1207 | | *Dihydrodipicolinate synthase* | | *Amino acid synthesis* |  |
|  | | *Dihydrodipicolinate synthase* | | *Amino acid synthesis* |  |
|  | | *N-acetylmuramoyl-L-alanine amidase* | | *Glycan Biosynthesis and Metabolism* |  |
| 1208 | | *Thymidine phosphorylase* | | *Pyrimidine metabolism* |  |
|  | | *Pyruvate-Formate Lyase* | | *Respiration* |  |
|  | | *L-glutamine synthase* | | *Amino acid synthesis* |  |
| 1209 | | *Diaminopimelate epimerase* | | *Amino acid synthesis* |  |
|  | | *Biosynthetic arginine decarboxylase PLP-binding* | | *Amino acid transport and metabolism* |  |
|  | | *Conjugated Bile Salt Hydrolase* | | *Exotic Metabolisms* |  |
| 1210 | | *Asparaginase* | | *Amino acid synthesis* |  |
|  | | *Pyruvate-Formate Lyase* | | *Respiration* |  |
|  | | *N-acetyl-D-galactosamine-4-sulfate 4-sulfohydrolase* | | *Glycosaminoglycan degradation* |  |
| 1211 | | *Conjugated Bile Salt Hydrolase* | | *Exotic Metabolisms* |  |
|  | | *Formyltetrahydrofolate synthetase* | | *Organic Acids* |  |
|  | | *N-acetylmuramoyl-L-alanine amidase* | | *Glycan Biosynthesis and Metabolism* |  |
| 1212 | | *Pyridoxal Kinase* | | *Cofactor Biosynthesis* |  |
|  | | *N-acetylmuramoyl-L-alanine amidase* | | *Glycan Biosynthesis and Metabolism* |  |
|  | | *Biosynthetic arginine decarboxylase PLP-binding* | | *Amino acid transport and metabolism* |  |
| 1213 | | *N-acetylmuramoyl-L-alanine amidase* | | *Glycan Biosynthesis and Metabolism* |  |
|  | | *Beta-N-acetyl-D-hexosaminide N-acetylhexosaminohydrolase* | | *Glycosaminoglycan degradation* |  |
|  | | *Alanine racemase* | | *Amino acid synthesis* |  |
| 1214 | | *N-acetylmuramoyl-L-alanine amidase* | | *Glycan Biosynthesis and Metabolism* |  |
|  | | *Diaminopimelate epimerase* | | *Amino acid synthesis* |  |
|  | | *Cytosine deaminase* | | *Pyrimidine metabolism* |  |
| 1215 | | *Thioredoxin reductase FAD-NADP-binding* | | *Pyrimidine metabolism* |  |
|  | | *Beta-N-acetyl-D-hexosaminide N-acetylhexosaminohydrolase* | | *Glycosaminoglycan degradation* |  |
|  | | *Prephenate dehydrogenase* | | *Amino acid synthesis* |  |
| 1216 | | *Methylmalonyl-CaA decarboxylase* | | *Organic Acids* |  |
|  | | *L-glutamine synthase* | | *Amino acid synthesis* |  |
|  | | *Prephenate dehydrogenase* | | *Amino acid synthesis* |  |
| 1217 | | *Pyruvate-Formate Lyase* | | *Respiration* |  |
|  | | *D-alanineD-alanine ligase* | | *Glycan Biosynthesis and Metabolism* |  |
|  | | *UDP-N-acetylmuramateL-alanine ligase* | | *Glycan Biosynthesis and Metabolism* |  |
| 1218 | | *UDP-N-acetylmuramateL-alanine ligase* | | *Glycan Biosynthesis and Metabolism* |  |
|  | | *Glutamate decarboxylase A and B PLP-dependent* | | *Amino acid transport and metabolism* |  |
|  | | *Diaminopimelate epimerase* | | *Amino acid synthesis* |  |
| 1219 | | *Pyruvate-Formate Lyase* | | *Respiration* |  |
|  | | *Carbon Monoxide Dehydrogenase* | | *Central Carbon Metabolism Pathways* |  |
|  | | *Cytosine deaminase* | | *Pyrimidine metabolism* |  |
| 1220 | | *Acetyl-CoA acyltransferase anaerobic* | | *Fatty Acid Metabolism* |  |
|  | | *Diaminopimelate epimerase* | | *Amino acid synthesis* |  |
|  | | *L-glutamine synthase* | | *Amino acid synthesis* |  |
| 1221 | | *N-acetyl-D-glucosamine-6-sulfate 6-sulfohydrolase* | | *Glycosaminoglycan degradation* |  |
|  | | *Prephenate dehydrogenase* | | *Amino acid synthesis* |  |
|  | | *Homoserine dehydrogenase* | | *Amino acid transport and metabolism* |  |
| 1222 | | *L-alanine dehydrogenase* | | *Amino acid synthesis* |  |
|  | | *Arginosuccinate synthase* | | *Amino acid synthesis* |  |
|  | | *Diaminopimelate epimerase* | | *Amino acid synthesis* |  |
| 1223 | | *Acetyl-CoA acyltransferase anaerobic* | | *Fatty Acid Metabolism* |  |
|  | | *N-acetylmuramoyl-L-alanine amidase* | | *Glycan Biosynthesis and Metabolism* |  |
|  | | *Pectinase (Pectinesterase)* | | *Complex Carbohydrates* |  |
| 1224 | | *Uridine phosphorylase* | | *Pyrimidine metabolism* |  |
|  | | *Ribokinase* | | *Feeder Pathways to Glycolysis* |  |
|  | | *Diaminopimelate epimerase* | | *Amino acid synthesis* |  |
| 1225 | | *Pyruvate-Formate Lyase* | | *Respiration* |  |
|  | | *Folylpolyglutamate Synthase* | | *Cofactor Biosynthesis* |  |
|  | | *UDP-N-acetylmuramoyl-L-alanyl-D-glutamatemeso-diaminopimelate ligase* | | *Glycan Biosynthesis and Metabolism* |  |
| 1226 | | *Prephenate dehydrogenase* | | *Amino acid synthesis* |  |
|  | | *Alpha-Glucosidase* | | *Complex Carbohydrates* |  |
|  | | *UDP-N-acetylmuramateL-alanine ligase* | | *Glycan Biosynthesis and Metabolism* |  |
| 1227 | | *Cystathionine gamma-synthase* | | *Amino acid synthesis* |  |
|  | | *Diaminopimelate epimerase* | | *Amino acid synthesis* |  |
|  | | *Anthranilate synthase* | | *Amino acid transport and metabolism* |  |
| 1228 | | *UDP-N-acetylmuramateL-alanine ligase* | | *Glycan Biosynthesis and Metabolism* |  |
|  | | *Pyruvate-Formate Lyase* | | *Respiration* |  |
|  | | *Beta-D-galactosidase* | | *Glycan structures - degradation;Complex Carbohydrates* |  |
| 1229 | | *Valine-pyruvate aminotransferase* | | *Amino acid synthesis* |  |
|  | | *Diaminopimelate epimerase* | | *Amino acid synthesis* |  |
|  | | *Uridine phosphorylase* | | *Pyrimidine metabolism* |  |
| 1230 | | *L-alanine dehydrogenase* | | *Amino acid synthesis* |  |
|  | | *Acetyl-CoA acyltransferase anaerobic* | | *Fatty Acid Metabolism* |  |
|  | | *Diaminopimelate epimerase* | | *Amino acid synthesis* |  |
| 1231 | | *N-acetylmuramoyl-L-alanine amidase* | | *Glycan Biosynthesis and Metabolism* |  |
|  | | *Pyridoxal Kinase* | | *Cofactor Biosynthesis* |  |
|  | | *Aspartate-ammonia ligase* | | *Amino acid synthesis* |  |
| 1232 | | *L-glutamine synthase* | | *Amino acid synthesis* |  |
|  | | *Quinolinate Synthase* | | *Cofactor Biosynthesis* |  |
|  | | *N-acetylmuramoyl-L-alanine amidase* | | *Glycan Biosynthesis and Metabolism* |  |
| 1233 | | *N-acetylmuramoyl-L-alanine amidase* | | *Glycan Biosynthesis and Metabolism* |  |
|  | | *UDP-N-acetylmuramateL-alanine ligase* | | *Glycan Biosynthesis and Metabolism* |  |
|  | | *Homoserine dehydrogenase* | | *Amino acid transport and metabolism* |  |
| 1234 | | *N-acetylmuramoyl-L-alanine amidase* | | *Glycan Biosynthesis and Metabolism* |  |
|  | | *Purine-nucleoside phosphorylase* | | *Purine metabolism* |  |
|  | | *Beta-N-acetyl-D-hexosaminide N-acetylhexosaminohydrolase* | | *Glycosaminoglycan degradation* |  |
| 1235 | | *Beta-ketoacyl-acyl-carrier-protein synthase III* | | *Fatty Acid Biosynthesis* |  |
|  | | *Carbamoyl phosphate synthetase small subunit glutamine amidotransferase* | | *Pyrimidine metabolism* |  |
|  | | *N-acetylmuramoyl-L-alanine amidase* | | *Glycan Biosynthesis and Metabolism* |  |
| 1236 | | *Diaminopimelate epimerase* | | *Amino acid synthesis* |  |
|  | | *Carbamoyl phosphate synthetase small subunit glutamine amidotransferase* | | *Pyrimidine metabolism* |  |
|  | | *Butyrate Kinase* | | *Organic Acids* |  |
| 1237 | | *Anthranilate synthase* | | *Amino acid transport and metabolism* |  |
|  | | *1-Phosphofructokinase* | | *Feeder Pathways to Glycolysis* |  |
|  | | *Pyruvate-Formate Lyase* | | *Respiration* |  |
| 1238 | | *N-acetylmuramoyl-L-alanine amidase* | | *Glycan Biosynthesis and Metabolism* |  |
|  | | *Serine-tRNA ligase* | | *Amino acid synthesis* |  |
|  | | *UDP-N-acetylmuramateL-alanine ligase* | | *Glycan Biosynthesis and Metabolism* |  |
| 1239 | | *Ribokinase* | | *Feeder Pathways to Glycolysis* |  |
|  | | *Homoserine dehydrogenase* | | *Amino acid transport and metabolism* |  |
|  | | *Pyruvate-Formate Lyase* | | *Respiration* |  |
| 1240 | | *Diaminopimelate epimerase* | | *Amino acid synthesis* |  |
|  | | *Uridine phosphorylase* | | *Pyrimidine metabolism* |  |
|  | | *Alanine racemase* | | *Amino acid synthesis* |  |
| 1241 | | *Gamma-glutamyl kinase* | | *Amino acid transport and metabolism* |  |
|  | | *Phosphogluconate dehydratase* | | *Central Carbon Metabolism Pathways* |  |
|  | | *Prephenate dehydrogenase* | | *Amino acid synthesis* |  |
| 1242 | | *Cystathionine gamma-synthase* | | *Amino acid synthesis* |  |
|  | | *Acetyl-CoA acyltransferase anaerobic* | | *Fatty Acid Metabolism* |  |
|  | | *Diaminopimelate epimerase* | | *Amino acid synthesis* |  |
| 1243 | | *3-deoxy-7-phosphoheptulonate synthase* | | *Amino acid transport and metabolism* |  |
|  | | *Diaminopimelate epimerase* | | *Amino acid synthesis* |  |
|  | | *Alanine racemase* | | *Amino acid synthesis* |  |
| 1244 | | *N-acetylmuramoyl-L-alanine amidase* | | *Glycan Biosynthesis and Metabolism* |  |
|  | | *Butyrate Kinase* | | *Organic Acids* |  |
|  | | *Homoserine dehydrogenase* | | *Amino acid transport and metabolism* |  |
| 1245 | | *Asparaginase* | | *Amino acid synthesis* |  |
|  | | *Pyruvate-Formate Lyase* | | *Respiration* |  |
|  | | *Acetyl-CoA acyltransferase anaerobic* | | *Fatty Acid Metabolism* |  |
| 1246 | | *Prephenate dehydrogenase* | | *Amino acid synthesis* |  |
|  | | *Biotin Synthase* | | *Cofactor Biosynthesis* |  |
|  | | *D-alanineD-alanine ligase* | | *Glycan Biosynthesis and Metabolism* |  |
| 1247 | | *Ornithine carbamoyltransferase 1* | | *Amino acid transport and metabolism* |  |
|  | | *N-acetylmuramoyl-L-alanine amidase* | | *Glycan Biosynthesis and Metabolism* |  |
|  | | *Carbon Monoxide Dehydrogenase* | | *Central Carbon Metabolism Pathways* |  |
