# Supplementary material for: Saliva Microbiota Carry Caries-Specific Functional Gene Signatures
Source: PLoS One. 2014 Feb 12;9(2):e76458. doi: 10.1371/journal.pone.0076458 (PMC3922703; doi:10.1371/journal.pone.0076458)
Supplement: Materials S1 — Supplementary materials. (DOC) [file pone.0076458.s008.doc]

**Supplementary Materials for the manuscript “Yang, Ning, et al, Saliva microbiota carry caries-specific functional gene signatures”**

**Materials and Methods**

***Study design*** All human hosts (nearly 700 individuals) were volunteers from an oral health census of the undergraduate students from the east campus of Sun Yat-sen University, Guangzhou, China in September, 2009. All were made aware of the nature of the experiment and provided written informed consent in accordance with the sampling protocol with approval of the ethical committee of the Guanghua Stomatological Hospital, Sun Yat-sen University. They were randomly selected individuals of both genders, aged between 18~23 years and shared a relatively homogeneous college-campus living environment. All reported no antibiotics intake for the preceding at least 6 months and no smoking or tobacco used. All were asked to avoid eating or drinking for one hour prior to oral sampling. Those with other oral (e.g. periodontitis or halitosis) or systematic diseases were excluded.

Dental examinations were performed by five professional dentists who were previously trained and calibrated for the evaluation and sampling procedures, according to the criteria defined by NIDCR (USA) for caries recording and diagnosis [1]. The DMFT index measures the number of decayed, missing and filled teeth in epidemiologic surveys of dental caries [2,3,4]. It was adopted in our study to measure each individual’s caries status and thus to define and distinguish the caries-active subjects and healthy subjects. The intra-examiner reproducibility in both the pilot phase and the main survey was assessed by kappa statistics, which was higher than 0.91. “Healthy” hosts (DMFT=0) and “caries-active” subjects (DMFT≧6) were chosen for saliva collection. Saliva from a total of 45 human hosts that include 19 caries-active and 26 healthy human were analyzed for organismal structure of microbiota by 16S-based amplicon sequencing [5]. In order to decipher the functional landscape of saliva microbiota, 20 saliva samples (including ten from the “healthy” group and ten from the “caries-active” group) were randomly selected for HuMiChip analysis (**Table 1**).

***HuMiChip analysis*** A functional gene microarray (HuMiChip1.0) was developed to interrogate microbial metabolism in human and mouse microbiota. The development of HuMiChip employed a modified pipeline as that in the well validated GeoChip3.0 [6], including six major steps: gene selection, sequence retrieval, sequence verification, probe design, probe check, and microarray construction. A total of 139 functional genes that play important metabolic roles were selected from KEGG pathway database [7]. Protein sequences corresponding to the selected genes were extracted from KEGG database and aligned for HMM model building. 322 draft/finished bacterial genomes and 27 shotgun metagenome datasets from various human body sites were downloaded from multiple public databases, including NCBI, HOMD (Human Oral Microbiome Database, http://www.homd.org), Oralgen (<http://www.oralgen.lanl.gov/>), and MGRAST server [8]. Predicted protein sequences were searched against prebuilt HMM models and sequences aligned to HMM databases with e value cutoff of 1e-3 were retrieved. Corresponding nucleotide sequences were used for probe designing by CommOligo2.0[9]. Candidate probes were searched against the above reference genomes and metagenomes as well as NCBI nt/env_nt databases for specificity. Microarrays were finally synthesized and manufactured by NimbleGen.

In total, 36,056 probes targeting 139 functional genes families were included in HuMiChip, covering 50,007 coding sequences. Among them, 24,188 (67.1%) are sequence-specific probes, and 11,868 (32.9%) are group-specific probes (**Table S1**). Specifically, 14,783 probes (41.0%) targeted 83 genes involved in amino acid metabolism, 6,539 probes (18.1%) targeted 36 genes related with carbohydrate metabolism, 3,292 probes (9.1%) targeted 14 energy metabolism genes, 4,682 probes (13.0%) targeted 15 genes involved glycan biosynthesis and metabolism, 2,680 probes (7.4%) targeted 17 genes related with cofactors and vitamins metabolism, covering 59.7%, 25.1%, 12.7%, 16.7% and 10.0% coding sequences, respectively. In addition, HuMiChip also contains 8 degenerate probes (80 replicates) targeting 16S rRNA sequences as positive controls, 563 probes (3 replicates) targeting seven sequenced hyperthermophile genomes as negative control. In addition, 3,000 replicates of a 50-mer common oligonucleotide reference standard were included as a common reference standard for data normalization and comparison [10]. Such design makes HuMiChip a comprehensive functional gene array for confident and accurate detection, identification, analyzing and comparative studies of human microbiome.

Ten “healthy” (4 males and 6 females) and ten “caries-active” (8 males and 2 females) were randomly chosen from the 45 saliva samples [5] for HuMiChip analysis (**Table S1**). Briefly, saliva DNA was extracted and fluorescently labeled with Cy5. Hybridizations were performed using a MAUI hybridization station (BioMicro, Salt Lake City, UT, USA) overnight at 42°C. HuMiChip microarrays were scanned using a NimbleGen MS200 scanner (Madison, WI, USA) at a laser power of 100%. Scanned images were analyzed using ImaGene 6.0 software (BioDiscovery, El Segundo, CA, USA).

Raw data obtained from microarray image analysis were uploaded to a microarray data manager (http://ieg2.ou.edu/NimbleGen) and preprocessed using the data analysis pipeline. Functional gene diversity (e.g., Shannon-Weaver index), detrended correspondence analysis (DCA), and permutation *t* tests were performed using R (version 2.9.1, http://www.r-project.org/). All statistical tests were two-sided. Permutation *t* tests were performed based on host dental health-state. Asterisks were used to denote statistical significance (NS: not significant; *: *p* <0.1; **: *p* <0.05; ***: *p* <0.01). Array data were deposited at the Gene Expression Omnibus with accession numbers GSE49875.

***Network reconstruction*** The presence of a protein can be represented as a normalized number representing its abundance (not expression) in all genomes in the dataset, or as either 0 (not present) or 1 (present). The 3,656 functional genes with hybridization signals on HuMiChip were grouped into “complete-presence genes” (“core”, i.e., those present in all the 20 saliva microbiota) and “partial-presence genes” (“non-core”, i.e., those missing in at least one saliva microbiota). The “complete-presence genes” were represented as normalized values according to their signal intensity, while the “partial-presence genes” as binary values (either 1 or 0).

Then the “co-presence” of two genes can be defined as the pair of normalized abundance values or binary values for the two genes: the presence of the two genes under the same condition suggests a potential functional link between the two genes. Based on this definition, the co-presence network is defined as a network in which each node is a protein, and each weighed edge between two nodes presents the Pearson co-presence relationship of the two genes, based on the co-presence profiles of the saliva microbiota. Based on this co-presence network, MCODE [11] is used to cluster the genes into densely connected clusters, in which the importance of clusters can be measured by their density centrality and transitive centrality based on a permutation model.

Exploiting the sample-size of ten healthy microbiota and ten caries-active microbiota, such co-presence analyses unveiled the interacting networks linking genes and pathways in saliva microbiota. The networks of core functional genes were based on normalized values of the “complete-presence genes” in H and C samples respectively. The networks of non-core functional genes were based on the binary value of the “partial-presence genes” on specific metabolic pathways that include: Carbon-associated pathway (including “*Complex carbohydrates*” and “*Feeder pathways to glycolysis” and “Respiration*”), AA-associated pathway (including “*Amino acid transport and metabolism*” and “*Amino acid synthesis*”) and Nitrogen-associated pathway (“*Nitrogen Metabolism”*).

.

***Biomarker selection*** To identify the features on HuMiChip 1.0 that could reliably distinguish caries-active saliva microbiota from healthy ones, we separated the ten healthy microbiota into seven sets for training and three samples by exhaustive permutation for testing (thus totally 120 different separations). The ten caries-active microbiotas were grouped under a similar strategy (120 different separations). Thus, there were 120*120=14,400 different configurations of training and testing data in total. Each saliva microbiota was represented as a binary presence profile of the non-core functional genes as defined by hybridization results of HuMiChip. We then used two steps, "feature selection" and "classification", to select the biomarkers that are highly sensitive to the disease state (**Figure S1**).

At the "feature selection" step, (i) for each of these 14,400 configurations, the top 50 features with the highest confidence level were selected by mRMR [12]; (ii) the top three features (triplet-features) from these top 50 were selected that exhibit the highest discrimination power on training data. The selection of triplet-features was the compromise between speed and discrimination power. This step resulted in a set of “candidate triplet-features” with high discrimination power. At the "classification" step, these candidate triplet-features were employed to “predict” the presence profiles of all 14,400 configurations of testing data (consisting of three healthy and three caries samples as testing data), and select the final list of triplet-features with the highest prediction power. Both the candidate triplet-features and the triplet-features after classification were subject to manual inspection to retrieve the final list of biomarkers.

**References**

1. Kaste LM, Selwitz RH, Oldakowski RJ, Brunelle JA, Winn DM, et al. (1996) Coronal caries in the primary and permanent dentition of children and adolescents 1-17 years of age: United States, 1988-1991. J Dent Res 75 Spec No: 631-641.

2. Boffano P, Roccia F, Pittoni D, Di Dio D, Forni P, et al. (2011) Management of 112 hospitalized patients with spreading odontogenic infections: correlation with DMFT and Oral Health Impact Profile 14 indexes. Oral Surg Oral Med Oral Pathol Oral Radiol Endod.

3. Eslamipour F, Borzabadi-Farahani A, Asgari I (2010) The relationship between aging and oral health inequalities assessed by the DMFT index. Eur J Paediatr Dent 11: 193-199.

4. Anaise JZ (1984) Measurement of dental caries experience--modification of the DMFT index. Community Dent Oral Epidemiol 12: 43-46.

5. Yang F, Zeng X, Ning K, Liu KL, Lo CC, et al. (2012) Saliva microbiomes distinguish caries-active from healthy human populations. ISME J 6: 1-10.

6. He Z, Deng Y, Van Nostrand JD, Tu Q, Xu M, et al. (2010) GeoChip 3.0 as a high-throughput tool for analyzing microbial community composition, structure and functional activity. ISME J 4: 1167-1179.

7. Kanehisa M, Araki M, Goto S, Hattori M, Hirakawa M, et al. (2008) KEGG for linking genomes to life and the environment. Nucleic Acids Research 36: D480-D484.

8. Meyer F, Paarmann D, D'Souza M, Olson R, Glass E, et al. (2008) The metagenomics RAST server - a public resource for the automatic phylogenetic and functional analysis of metagenomes. BMC Bioinformatics 9: 386.

9. Nasidze I, Quinque D, Li J, Li M, Tang K, et al. (2009) Comparative analysis of human saliva microbiome diversity by barcoded pyrosequencing and cloning approaches. Anal Biochem 391: 64-68.

10. Liang Y, He Z, Wu L, Deng Y, Li G, et al. (2010) Development of a Common Oligonucleotide Reference Standard for Microarray Data Normalization and Comparison across Different Microbial Communities. Applied and Environmental Microbiology 76: 1088-1094.

11. Bader GD, Hogue CW (2003) An automated method for finding molecular complexes in large protein interaction networks. BMC Bioinformatics 4: 2.

12. Ding C, Peng H (2005) Minimum redundancy feature selection from microarray gene expression data. J Bioinform Comput Biol 3: 185-205.
